# A Precise Synthetic Toolbox: H-Bond-Assisted Quadruple Reactivity of *o*-Dimethylaminoaryloximes

**DOI:** 10.1021/acs.joc.5c00207

**Published:** 2025-03-13

**Authors:** Semyon V. Tsybulin, Stepan A. Meshalkin, Daria I. Tonkoglazova, Victor G. Bardakov, Alexander F. Pozharskii, Alexander S. Antonov

**Affiliations:** † Institute of Chemistry, St. Petersburg State University, St. Petersburg 198504, Russian Federation; ‡ Department of Organic Chemistry, Southern Federal University, Rostov-on-Don 344090, Russian Federation; § Institute of Organic Chemistry, University of Regensburg, D-93053 Regensburg, Germany

## Abstract

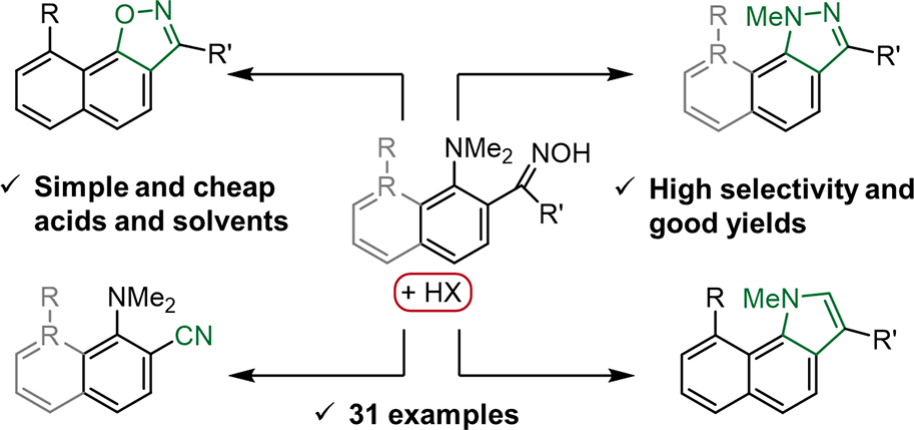

Non-covalent interactions are a highly promising tool for the development of transition-metal-free chemospecific synthetic transformations. Here, we demonstrate the implementation of non-covalent interactions as a simple, precise, and flexible synthetic toolbox, allowing the controlled transformation of *o*-dimethylaminoaryloximes into nitriles and hard-to-reach nitrogen heterocycles under mild conditions. This diverse reactivity is activated via hydrogen bonding and facilitated via the “buttressing effect” of the substituents next to the NMe_2_ group. All transformations require only simple and easily available acids and solvents, which generally provide precise control over the direction of the reaction, allowing the selective synthesis of nitriles, fused pyrazoles, isoxazoles, and pyrroles.

## Introduction

A simple, efficient, and flexible approach to the construction of complex molecules has long been a central objective in chemical synthesis due to the high demand of the pharmaceutical industry. Therefore, the development of universal synthetic instruments that enable transformations in a chemospecific manner has become an unceasing quest for synthetic chemists. Regardless of the considerable progress, the ability to achieve switchable reactivity with a common reagent remains a challenge.

**Scheme 1:**
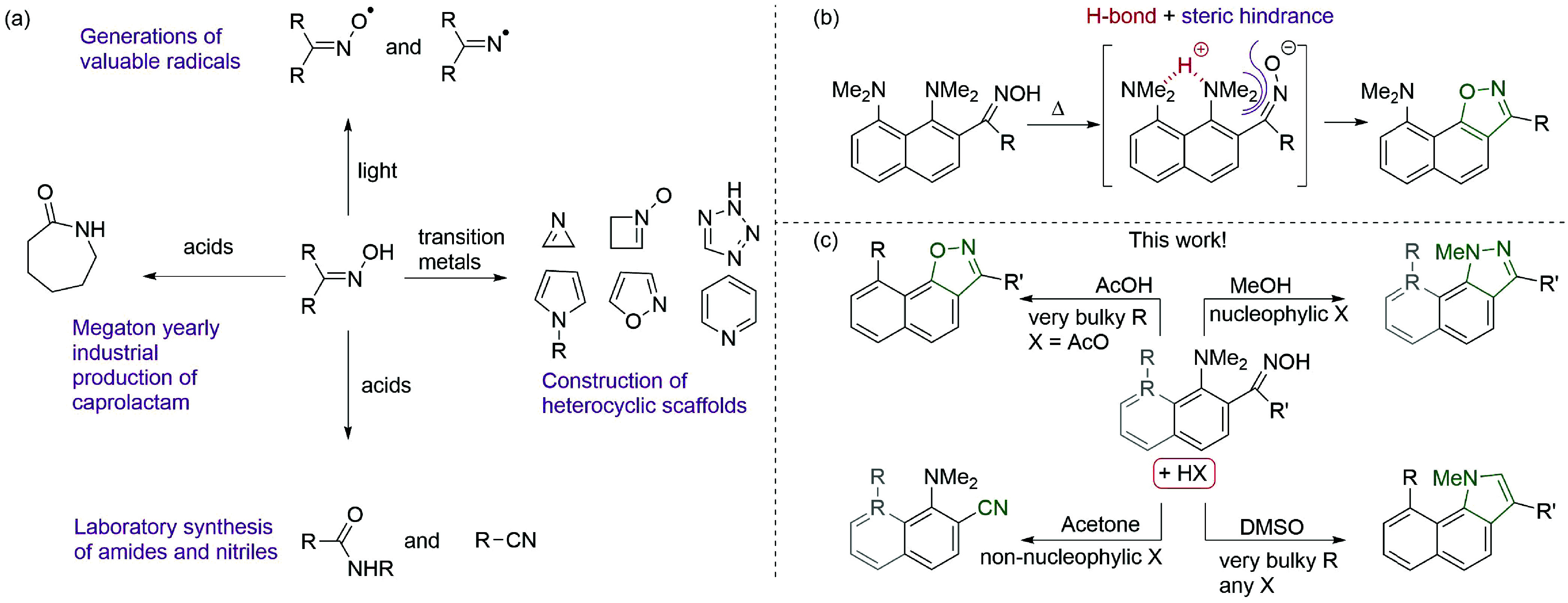
Reactivity of Oximes (a) Industrial and laboratory applications for the preparation of valuable chemicals. (b) Example of effective implementation of non-covalent interactions for enabling unusual oxime transformations: the H-bond-assisted, sterically facilitated intramolecular nucleophilic substitution of the NMe_2_ group leads to the formation of naphtho[2,1-*d*]isoxazoles. (c) Our tunable approach, relying on hydrogen bonding and the steric hindrance of the substrate, allows the selective quadruple transformation of *o*-dimethylaminoaryloximes in acidic media: a highly efficient dehydration to nitriles under mild conditions, demethylation-assisted cyclization in indazoles, the NMe_2_ group substitution with the formation of isoxazoles, and HAT-induced pyrrole ring construction.

Due to the presence of two heteroatoms, oximes are excellent and versatile reagents for the laboratory construction of valuable chemicals. Oximes not only find important application in the Beckmann rearrangement^1−4^— the key step in the yearly industrial production of megatons of caprolactam, a precursor of nylon-6 — but also in various metal-promoted,^5−8^ radical,^9−11^ and bioconjugated^12,13^ transformations, especially for the construction of heterocyclic scaffolds^14−17^ (Scheme 1a). Recent advances in oxime-based heterocyclizations have focused on transition metal catalysis, with a few exceptions, such as the Trofimov reaction.^18^ Despite the grand success of transition metal catalysis, the high cost of metals like palladium, platinum, and rhodium, along with the need for complex and expensive ligands, as well as challenges in purifying the final products from toxic transition metals, continue to limit its application. Moreover, the construction of a specific heterocycle (be it pyrroles, oxazoles, or pyridines) requires different strategies and substrate-specific reagents, thus lacking a universal key to a chemical space that is difficult to access. Therefore, we envisioned that the ability to achieve chemodivergent transformations of oximes into valuable heterocycles with a common reagent would accelerate industrially applied research.

The implementation of non-covalent interactions, such as hydrogen bonding, appears to be a promising transition-metal-free approach to achieve diverse reactivity. For instance, we have previously reported the efficient synthesis of naphtho[2,1-*d*]isoxazoles via a simple thermolysis of 1,8-bis(dimethylamino)naphthoketoximes (Scheme 1b).^19^ Later, by using quantum chemical calculations, we revealed that this unusual reactivity originates from the simultaneous action of hydrogen bonding, which activates the leaving group, and steric interactions, which provide proximity of the reacting centers and the general strain of the substrate.^20^ Based on the above-mentioned and keeping the high synthetic potential of oximes in mind, the “dream” approach for both laboratory and industrial applications would be a selective, controlled transformation of one oxime substrate into various useful chemicals. It would be especially beneficial if this process was transition-metal-free, relied on tunable non-covalent interactions, and involved the use of only simple and cheap reagents, such as acids. To answer this challenge, here we present an efficient toolbox for the selective quadruple transformation of *o*-dimethylaminoaryloximes in acidic media into nitriles, pyrazoles, isoxazoles, and pyrroles (Scheme 1c). All of the studied reactions are activated via hydrogen bonding, facilitated by steric interactions, and controlled by the nature of acid.

## Results and Discussion

Steric hindrance of the substrate is key to the implementation of our synthetic toolbox. For convenience, further discussion is constructed in a way that allows one to see how the increase of steric hindrance enables new reaction routes. That is why we start with unsubstituted, benzene-based oximes, proceed to *ortho*-substituted ones, and finish with naphthalene-based compounds. The synthesis of the tested aldoximes and ketoximes was performed by treatment of the corresponding aldehydes^19,21^ or imines^19,22^ with hydroxylamine (or methoxyamine). The experimental results are followed by mechanistic considerations.

### Steric Control of the Reactivity of Benzene-Based Aldoximes

All of our experiments with benzene-based aldoximes are summarized in Tables 1 and S1. It is reasonable to start the discussion with the most representative cases of oxime **1a**, containing no substituent at position 6, and oxime **1b**, containing the largest tested TMS substituent. Refluxing the mixture of **1a** with aqueous HBF_4_, HCl, or HI in methanol for 12 h or heating **1a** in acetic acid at 65 °C leaves the starting material unchanged (runs 1–4). Only heating in DMSO at 100 °C for 12 h in the presence of aqueous HCl allowed a complete transformation of **1a** into a 1:0.5 mixture of nitrile **2a** and indazole **3a** (run 5). In contrast, heating the sterically strained **1b** in acetone with HBF_4_ or in methanol with HI for 3 h results in the complete selective transformation into nitrile **2b** and indazole **3b**, respectively (runs 6, 7). A similar outcome is achieved for oximes **1c** (*R* = Br) and **1d** (*R* = SMe); however, full conversion into nitrile requires a longer reaction time (runs 9, 10, 12, 13). Transition to **1e**, containing a methyl substituent at position 6, leads to a significant decrease of reactivity (run 16).

**Table 1:**
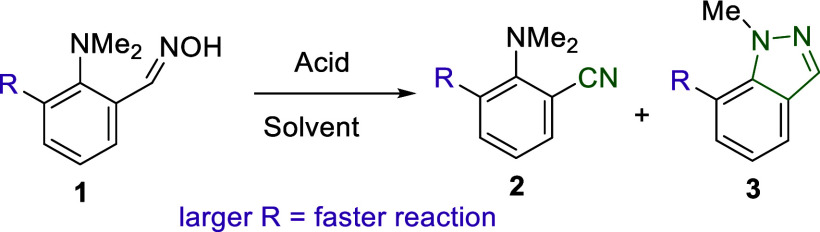
Transformation of *o*-Dimethylaminobenzaldoximes in Acidic Media^a^

							products ratio		
run	oxime	*R*	acid	solvent	*T* (°C)	time (h)	**1**	**2**	**3**
1	**1a**	H	HBF_4_	MeOH	65	12	1	–	–
2	**1a**	H	HCl	MeOH	65	12	1	–	–
3	**1a**	H	HI	MeOH	65	12	1	–	–
4	**1a**	H	AcOH	AcOH	65	12	1	–	–
5	**1a**	H	HCl	DMSO	100	12	–	1	0.5
6	**1b**	TMS	HBF_4_	acetone	65	3	–	1	–
7	**1b**	TMS	HI	MeOH	65	3	–	–	1
8	**1b**	TMS	AcOH	AcOH	65	3	1	–	–
9	**1c**	Br	HBF_4_	acetone	65	12	–	1	–
10	**1c**	Br	HI	MeOH	65	3	–	–	1
11	**1c**	Br	AcOH	AcOH	65	3	8.5	1	–
12	**1d**	SMe	HBF_4_	acetone	65	48	–	1	–
13	**1d**	SMe	HI	MeOH	65	3	–	–	1
14	**1d**	SMe	AcOH	AcOH	65	3	4	1	–
15	**1e**	Me	HBF_4_	acetone	65	3	1	–	–
16	**1e**	Me	HI	MeOH	65	12	–	0.1	1
17	**1e**	Me	AcOH	AcOH	65	3	5.5	1	–

a Steric hindrance enables both reaction routes, while the nature of the acid determines the selectivity. See all tested conditions in Table S1.

Overall, on the one hand, the implementation of the “buttressing effect”^21^ via the introduction of substituent *R* dramatically facilitates the reactivity of **1**, and, on the other hand, the choice of the nucleophilicity of the reaction media allows precise control of the direction of the transformation: non-nucleophilic media provide a selective formation of nitriles, while nucleophilic media solely give indazoles. It should be noted that while the behavior of **1** in MeOH and acetone follows the principle “the larger the substituent *R*, the better the reactivity”, the transformation of **1** in AcOH demonstrates a nonlinear dependence: small (H) and very large (TMS) substituents provide no reactivity, which leaves the starting material unchanged after 3 h of heating at 65 °C, while medium-sized substituents (Me, Br, SMe) allow the selective, slow formation of nitriles **2** (runs 8, 11, 14, 17).

### Steric Control of the Reactivity of Naphthalene-Based Aldoximes

All of our experiments with naphthalene-based aldoximes are summarized in Tables 2 and S2. The annulation of the benzene ring upon transition from benzaldoximes **1** to naphthaldoximes **4** dramatically affects the reactivity of the latter. Thus, unsubstituted in position 8, aldoxime **4a** demonstrated a reactivity similar to that of **1e**. For instance, after 12 h of heating in acetone with aqueous HBF_4_ or 3 h in methanol with aqueous HI, **4a** undergoes a complete selective transformation into nitrile **5a** and indazole **6a**, respectively (Table 2, runs 1, 2). Prolonged heating in acetic acid enables the transformation into isoxazole **7a**, as it was predicted by means of quantum chemistry in our previous work;^20^ however, with poor selectivity: after 12 h, the reaction mixture consists of a 1:0.8 mixture of **5a** and **7a,** together with a small amount of the starting material (run 3). The transition to the most sterically hindered aldoxime **4b**, containing the TMS group, dramatically changes the reaction output. Thus, heating of **4b** in acetone with aqueous HBF_4_ solely provides indole **8b**, with no traces of the expected nitrile **5b** (run 4).

**Table 2:**
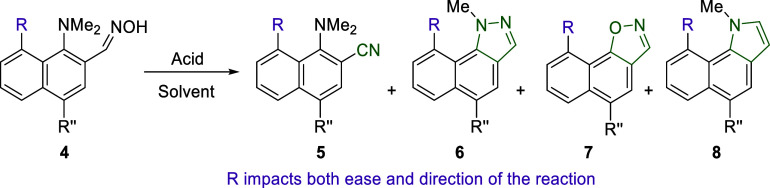
Transformation of *o*-Dimethylaminonaphthaloximes in Acidic Media^a^

								products ratio				
run	oxime	*R*	*R*″	acid	solvent	*T* (°C)	time (h)	**4**	**5**	**6**	**7**	**8**
1	**4a**	H	Me	HBF_4_	acetone	65	12	–	1	–	–	–
2	**4a**	H	Me	HI	MeOH	65	3	–	–	1	–	–
3	**4a**	H	Me	AcOH	AcOH	65	12	0.1	1	–	0.8	–
4	**4b**	TMS	Me	HBF_4_	acetone	65	9	–	–	–	–	1^d^
5	**4b**	TMS	Me	HI	MeOH	65	9	–	–	–	0.9	1^d^
6	**4b**	TMS	Me	HCl	MeOH	65	9	–	–	–	1.9	1^d^
7	**4b**	TMS	Me	HBF_4_	MeOH	65	9	–	–	–	1.6	1^d^
8	**4b**	TMS	Me	AcOH	AcOH	65	9	0.5	–	–	1	–
9	**4c**	NMe_2_	H	HBF_4_	acetone	65	3	1	–	–	–	–
10	**4c**	NMe_2_	H	HI	MeOH	65	3	1	–	–	–	–
11	**4c**	NMe_2_	H	AcOH	AcOH	65	3	1	–	–	–	–
12^b^	**4c**	NMe_2_	H	HI	*n*-BuOH	100	81	–	–	–	–	1
13^b^	**4c**	NMe_2_	H	HI	DMSO	100	22	–	–	–	–	1
14	**4c**	NMe_2_	H	HCl	DMSO	100	72	–	1	–	–	–
15	**4d**	SMe	Me	HBF_4_	acetone	65	3	–	1	–	–	–
16	**4e**	Me	Me	HBF_4_	acetone	65	3	–	1	–	–	–
17	**4d**	SMe	Me	HI	MeOH	65	3	–	–	1	–	–
18	**4e**	Me	Me	HI	MeOH	65	3	–	–	1	0.1	–
19	**4d**	SMe	Me	AcOH	AcOH	65	12^c^	0.25	–	–	1	–
20	**4e**	Me	Me	AcOH	AcOH	65	3	–	–	–	1	–

a Steric hindrance enables four reaction routes, while the nature of the acid and substituent *R* determine selectivity. See all tested conditions in Table S2.

b Pure **4c·HI** was heated in neat solvent.

c Longer reaction time leads to significant tarring.

d In the non-aromatic form **21b**.

Switching to MeOH/HI media provides a 0.9:1 mixture of isoxazole **7b** with indole **8b**, while no traces of the expected indazole **6b** were detected (run 5). This ratio can be significantly improved in favor of **7b** by using HCl or HBF_4_ in MeOH (runs 6, 7). The utilization of AcOH as a reaction medium allows us to achieve selectivity of **7b** formation; however, prolonged heating leads to significant tarring, and the short reaction time does not provide a full conversion (run 8).

Even more peculiar is the behavior of oxime **4c**, containing the NMe_2_ group at position 8. Due to the strong basicity of the naphthalene “proton sponges”,^23^ this compound binds acid, which switches off the desired “buttressing effect” (due to the removal of electrostatic repulsion) and thus suppresses reactivity. For instance, heating **4c** in methanol with HI, in acetone with HBF_4_, or in neat AcOH leaves the starting material unchanged (runs 9–11). At the same time, heating of separately prepared and isolated **4c**·HI in *n*-BuOH at 100 °C for 81 h allows the activation of the substrate, which, similarly to **4b**, exclusively gives the corresponding indole **8c** (run 12). Switching the solvent to DMSO facilitates reactivity; thus, the full conversion of **4c·**HI into **8c** is achieved after 22 h at 100 °C (run 13). Heating of **4c** with two equivalents of HCl in DMSO leads to the selective formation of nitrile **5c** (run 14). None of the tested reaction conditions allowed the transformation of **4c** into **7c** (see Table S2).

The behavior of aldoximes **4d** and **4e**, containing SMe and Me substituents, is more straightforward and better tunable. For instance, heating in acetone/HBF_4_ media selectively gives nitriles **5d** and **5e** (runs 15, 16). Switching to MeOH/HI media allows selective preparation of indazoles **6d** and **6e** (runs 17, 18). Utilization of AcOH as reaction media provides access to isoxazoles **7d** and **7e** (runs 19, 20).

### Steric Control of the Reactivity of Ketoximes

Notably, our attempt to transfer our findings to ketoximes was rather challenging, since the “buttressing effect” provided by substituent *R* hampers the reaction of organolithium precursors^21^ with nitriles, as well as the reaction of imines with hydroxylamine (or methoxyamine). Thus, it was possible to obtain only a few ketoximes, which were tested in acid-promoted reactions (Tables 3 and S3).

**Table 3:**
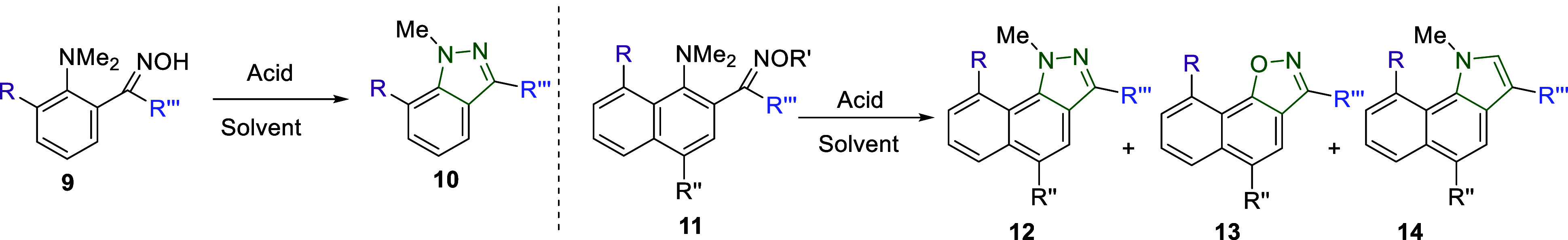
Transformation of *o*-Dimethylaminoarylketoximes in Acidic Media^a^

										products ratio			
run	oxime	*R*	*R*′	*R*″	*R*‴	acid	solvent	*T* (°C)	time (h)	**9** (**11**)	**10** (**12**)	**13**	**14**
1	**9a**	H	–	–	*p*-Tol	HI	MeOH	65	36	1	–	–	–
2	**9a**	H	–	–	*p*-Tol	HI	DMSO	100	48	1	–	–	–
3	**9b**	TMS	–	–	*p*-Tol	HI	MeOH	65	24	1.4^b^	1	–	–
4	**9b**	TMS	–	–	*p*-Tol	HCl	DMSO	100	70	1^c^	1	–	–
5	**11a**	H	H	Me	*p*-Tol	HI	MeOH	65	72	–	0.6	1	–
6	**11a**	H	H	Me	*p*-Tol	AcOH	AcOH	65	12	0.2	–	1	–
7	**11a**	H	H	Me	*p*-Tol	HCl	DMSO	100	12	3	–	1	–
8	**11b**	H	H	Me	*n*-Bu	HI	MeOH	65	48	–	1	–	–
9	**11c**	TMS	H	Me	*p*-Tol	HI	MeOH	65	20	–	–	1	–
10^d^	**11d**	NMe_2_	H	H	Ph	HCl	DMSO	100	72	–	–	1	–
11^e^	**11e**	Me	H	Me	*p*-Tol	HCl	EtOH	85	24	–	–	1	–
12^f^	**11f**	H	Me	Me	*p*-Tol	HCl	EtOH	85	24	–	–	–	1
13^d^	**11g**	NMe_2_	Me	H	Ph	HI	DMSO	100	21	–	–	–	1
14^d^	**11h**	NMe_2_	Me	H	*p*-Tol	HI	DMSO	100	21	–	–	–	1

a Introduction of substituent *R*’’’ decreases the overall reactivity; however, significantly facilitates the formation of isoxazoles.

b 0.4 of **9b** + 1.0 of **9a** due to the desilylation.

c In a form of **9a** due to the desilylation.

d Pure **11**·HX was heated in the solvent.

e Oxime **11e** was not isolated; the reaction of the corresponding imine in the provided conditions with hydroxylamine hydrochloride gives **13e** as the only product.

f *o*-Me oxime **11f** was not isolated; the reaction of the corresponding imine in the provided conditions with methoxyamine gives **14f** as the only product.

Similarly to the corresponding aldoxime, ketoxime **9a** demonstrates no reactivity under the tested conditions (Table 3, runs 1, 2). The transition to oxime **9b**, containing the TMS substituent in position 6, activates the reactivity, which, however, is much inferior in comparison with that of the corresponding aldoxime **1b**. Thus, prolonged heating of **9b** in MeOH with HI or in DMSO with HCl leaves a significant amount of starting material unreacted (runs 3, 4). This is partially related to the protodesilylation of **9b**, leading to the formation of unreactive **9a**. We believe that the steric pressure of the ketoxime substituent facilitates the nucleophile-induced (I^–^ or Cl^–^) desilylation, which, due to the overall reduced reaction rates, effectively competes with the desired transformation into indazole **13b**.

The transition to naphthoketoximes **11** demonstrates that the introduction of substituent *R*’’’ significantly facilitates the formation of isoxazoles **13**, hampering the formation of indazoles. Thus, prolonged heating of **11a** in acidic media gives **13a** as a major product (Table 3, runs 5–7). Reducing the size of *R*’’’ via the use of *n*-Bu instead of *p*-Tol provides a clear transformation of **11b** into indazole **12b** (run 8). However, the introduction of the TMS group in the case of **11c** again leads to the selective formation of isoxazoles (run 9). A similar effect was achieved for **11d** and **11e**, containing NMe_2_ and Me groups at position 8, respectively (runs 10, 11). Altogether, increasing the bulkiness of the surrounding of the 1-NMe_2_ group strongly facilitated its substitution with the formation of the corresponding isoxazole. Our attempt to suppress this isoxazole formation via the use of *O*-methyl oximes surprisingly led to the selective formation of indoles **14**. For instance, during the preparation of **11f** from the corresponding imine and methoxyamine, indole **14a** was formed as the only product (run 12). The synthesis of **11g** and **11h** via the same method was successful, and their prolonged heating in DMSO with HI also led to the selective preparation of the corresponding indoles **14g** and **14h** (runs 13, 14). Overall, the formation of indoles obeys the same principle as other types of investigated heterocyclizations: the bigger *R* is, the faster the reaction is (if the formation of oxime is possible at all due to steric reasons).

### Mechanistic Considerations

**Scheme 2:**
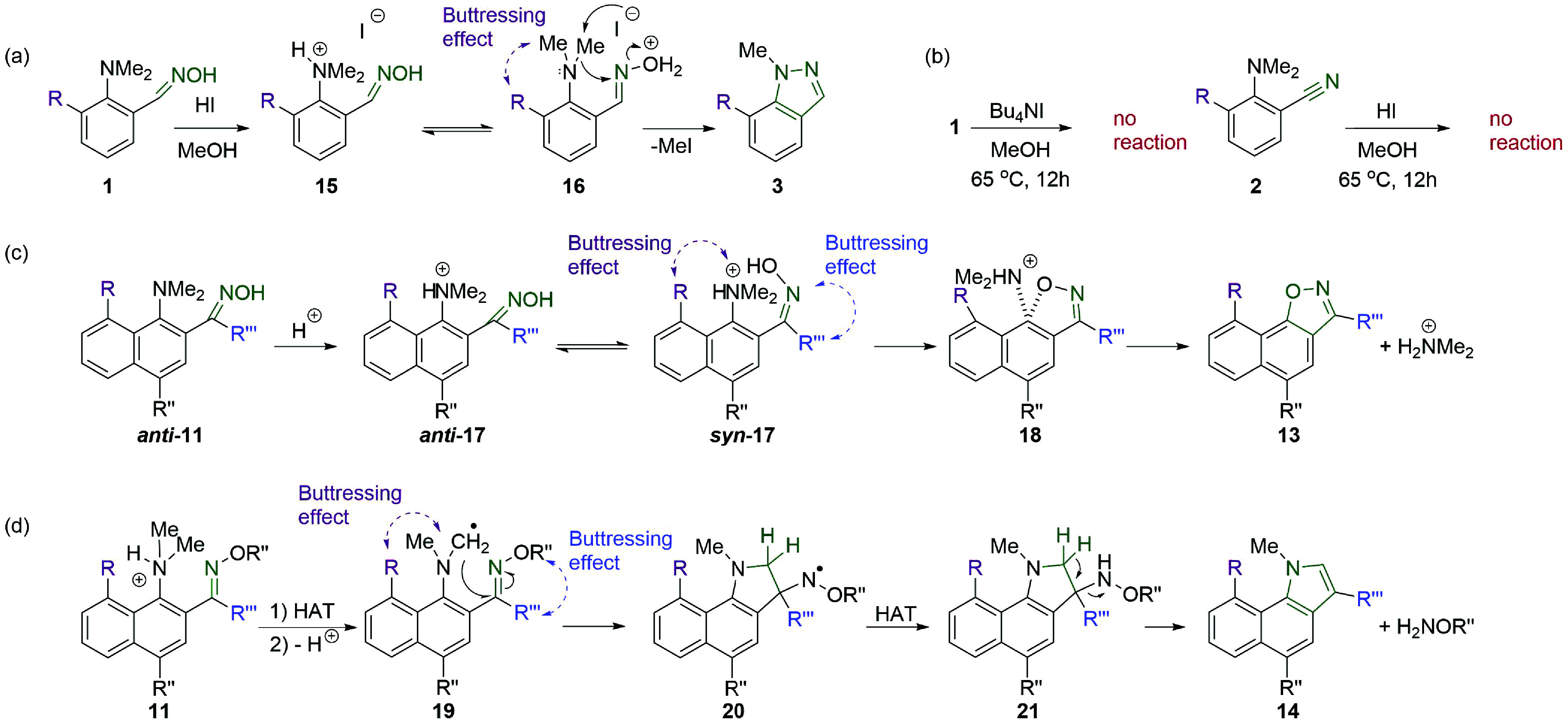
Proposed Mechanisms of Discovered Heterocyclizations (a) Indazoles formation via nucleophile-intercepted Beckmann rearrangement and (b) supporting additional reactivity tests. (c) Isoxazoles formation via intramolecular nucleophilic substitution of NMe_2_ group. (d) Indoles formation via hydrogen atom transfer process followed by intramolecular radical trapping (cyclization).

While the formation of nitriles **2** and **5** upon heating of oximes **1** and **4** in the presence of acid is obviously an acid-promoted thermal Beckmann rearrangement, the formation of indazoles **3**, **6,** and **12** is rather unexpected. Our experiments clearly demonstrate that the formation of **3** requires the presence of a nucleophile, while non-nucleophilic acids only promote the formation of nitriles **2**. At the same time, simple heating of the most reactive **1b** with tetrabutylammonium iodide in methanol left the starting material unchanged. Thus, the simultaneous presence of acid and the nucleophile is detrimental to the discovered heterocyclization. Based on the above-mentioned, we believe that the reaction mechanism can be described as a nucleophile-intercepted Beckmann rearrangement (Scheme 2a). First, the protonation of **1** leads to the formation of the ion pair **15**. Second, the proton transfer to the oxygen atom leads to the formation of an equilibrating amount of ion pair **16**. The presence of the bulky substituent *R* forces the proximity of the NMe_2_ and NOH groups — the “buttressing effect” — creating steric strain and facilitating further transformations. If no nucleophile is present in the reaction mixture, **16** loses a water molecule and gives nitrile **2**. In the presence of the nucleophile, its interaction with the NMe group of **16** initiates a chain of intramolecular nucleophilic attacks, leading to the formation of a pyrazole cycle. The possibility of the formation of **5** via the interaction of **2** with nucleophilic acid was excluded based on the treatment of nitrile **2b** with HI in boiling MeOH, leaving the starting material unchanged (Scheme 2b).

The formation of isoxazoles **7** and **13** is more straightforward and occurs via intramolecular nucleophilic substitution of the NMe_2_ group (Scheme 2c). In our previous paper, we predicted this reaction outcome by means of quantum chemical calculations and demonstrated that the increase in the size of substituent *R* facilitates the transformation by decreasing the activation barrier of the substitution stage *syn***–17** → **18**.^20^ Noticeably, both substituents *R* and *R*’’’ provide the “buttressing effect” and assist in the substitution of the NMe_2_ group. With this mechanism in mind, it is not surprising that less aromatic naphthalene substrates easily undergo displacement of the NMe_2_ group, while benzene-based oximes avoid this transformation even in the presence of the TMS group in position 6.

Surprisingly, the formation of indoles effectively competes with the formation of isoxazoles and indazoles in the case of naphthalene-based oximes. Keeping in mind that the transition from HBF_4_/DMSO to HI/DMSO systems significantly facilitates indole formation (see Table 2, run 13, and Table S2, run 33) and taking into account the one-electron-reducing nature of the iodide anion^24^ combined with the ability of DMSO to form radical species, we assumed that the radical mechanism in the indole-type cyclization can be operative (Scheme 2d). We believe that the reaction starts with the hydrogen atom abstraction process. Since HAT processes in tertiary amines could be facilitated by hydrogen bonding with an attacking radical,^25^ we assume that the protonation of the dimethylamino group moiety by external acid (HI) could play a similar role. The hydrogen atom abstraction from **11**, followed by deprotonation, leads to the so-called “nucleophilic radical” **19**, which is additionally stabilized by the nitrogen lone pair via a 2c,3e-bond.^26^ The radical **19** then attacks the most electrophilic reactive site, forming the heterocyclic radical **20**. Further quenching of radical **20** leads to the formation of dihydroindoles **21**, which, under the reaction conditions, undergo spontaneous aromatization via the elimination of a hydroxylamine (or methoxyamine) molecule. The above-mentioned mechanistic assumptions are in agreement with additional experiments. First, in the absence of oxygen (degassed *n*-BuOH), **4c·HI** gives no indole **8c**. This indicates the importance of O_2_ in initiating or propagating the process. Second, the addition of 2 equivalents of KI to the reaction mixture (in the presence of oxygen) resulted in a higher conversion of **4c·HI** into **8c**. Third, the addition of TEMPO dramatically facilitates the formation of **8c**. Since TEMPO catalyzes the hydrogen atom transfer,^27^ it can promote the formation of the radicals and therefore facilitate the cyclization into **8c**. It should be noted that no TEMPO adducts were found in the reaction mixture according to ESI-HRMS. Though unexpected, this demonstrates that intramolecular radical trapping (cyclization) is faster than intermolecular interception by TEMPO.^28^ We believe that this fast intramolecular trapping is a key step in the discovered transformation, and it is enabled by the “buttressing effect” forcing the radical center and oxime moiety into proximity.

## Conclusion

**Scheme 3:**
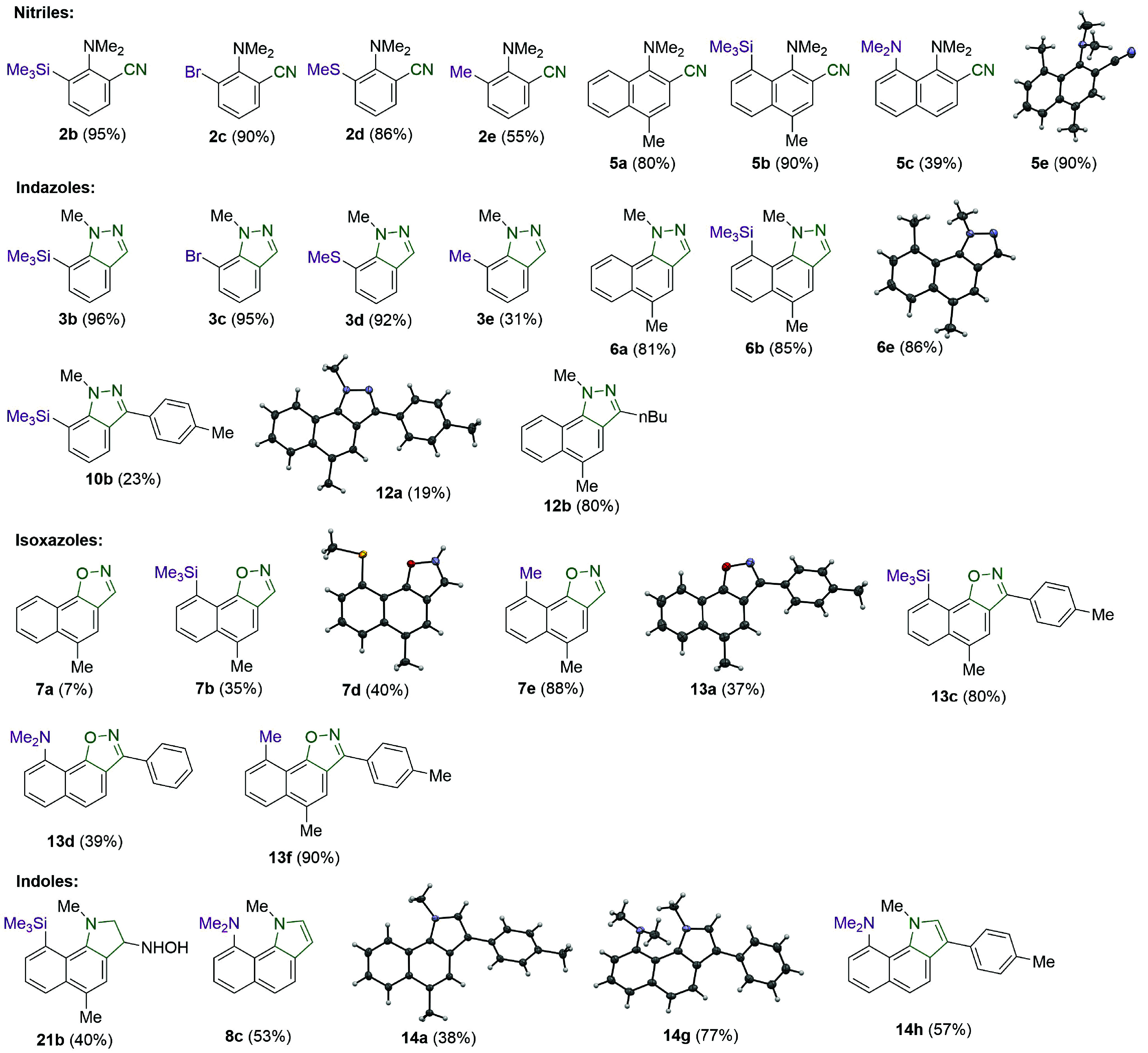
Overview of the Nitriles, Indazoles, Isoxazoles, and Indoles Obtained via the Reaction of *o*-Dimethylaminoaryloximes in Acidic Media

In summary, we have demonstrated a simple, precise, and flexible synthetic toolbox, allowing for the controlled transformation of *o*-dimethylaminoaryloximes into nitriles and hard-to-reach nitrogen heterocycles under mild conditions (Scheme 3). All discovered transformations are activated via hydrogen bonding with acids and facilitated via the “buttressing effect” of the substituents next to the NMe_2_ group. The choice of acid and solvent allows precise control over the direction of the reaction. Utilization of non-nucleophilic media consisting of HBF_4_ in acetone allows the selective, high-yielding preparation of nitriles without the usage of additional dehydrating agents. Performing the reaction in nucleophilic media, such as HI in MeOH, provides the effective preparation of various pyrazoles. The presence of the nucleophile intercepts the thermal Beckmann rearrangement of oximes via demethylation of the NMe_2_ group, which is followed by an intramolecular nucleophilic attack on the oxime nitrogen, furnished with the displacement of water. Switching to acetic acid as a reaction medium allows the selective synthesis of naphtho[*2,1-d*]isoxazoles. Here, hydrogen bonding with AcOH activates the intramolecular displacement of the NMe_2_ group, facilitated by the “buttressing effect” of the substituent in position 8, forcing the proximity of interacting groups and providing a distortion of the naphthalene core. More aromatic benzene-based oximes show inertness toward this transformation. Finally, in some cases, it is possible to selectively transform naphthalene-based oximes into benzo[*g*]indoles via a hydrogen atom transfer from the NMe group. The effective realization of this reaction direction requires a strong fixation of the conformation of the NMe_2_ group (either by a very bulky TMS substituent or strong hydrogen bonding with the second NMe_2_ group) and the source of radicals (iodine ions, oxygen, and TEMPO). The formation of indoles competes with the formation of isoxazoles; thus, better results are achieved for *O*-Me oximes, which are not able to transform into isoxazoles.

## Experimental Procedures

### General

Solvents used in organometallic reactions were dried over sodium-benzophenone. Unless otherwise stated, all other solvents and commercial reagents were used without additional purification. An oil bath was used as a heat source. Reaction temperatures were reported as the temperatures of the bath surrounding the flasks or vials.

Liquid-state NMR experiments were performed using a Bruker Avance III NMR spectrometer (400 MHz for ^1^H and 100 MHz for ^13^C) at the Center for Magnetic Resonance, St. Petersburg State University Research Park. Chemical shifts are referenced to TMS for ^1^H and ^13^C.

Single crystals of **4d**, **13a,** and **14g** were grown by slow evaporation of Et_2_O solution at +25 °C; **5e**, **6e**, **12a**, **7d,** and **14a** by slow evaporation of Et_2_O solution at −25 °C. The single-crystal X-ray diffraction data were collected using the SuperNova diffractometer equipped with a HyPix-3000 detector and a microfocus Cu Kα radiation source (λ = 1.54184 Å) at temperatures *T* = 100 (2) or 120 K at the Centre for X-ray Diffraction Studies, St. Petersburg State University Research Park. Using Olex216, the structure was solved with the SHELXT^29^ structure solution program using Intrinsic Phasing and refined with the SHELXL^30^ refinement package using Least Squares minimization.

HR-ESI mass spectra were obtained on a Bruker maXis spectrometer equipped with an electrospray ionization (ESI) source; methanol was used as the solvent at the Chemical Analysis and Materials Research Centre, St. Petersburg State University Research Park. The instrument was operated in positive mode using an *m*/*z* range of 50–1200. The capillary voltage of the ion source was set at 4000 V. The nebulizer gas pressure was 1.0 bar, and the drying gas flow was set to 4.0 L/min.

### Examples of Synthetic Procedures

#### 1-(Dimethylamino)-4-Methyl-2-Naphthonitrile **5a**

A solution of the oxime **4a** (0.25 mmol, 57 mg), HBF_4_ (50% aqueous solution, 0.12 mL, 0.50 mmol, 2 equiv) in acetone (10 mL) was stirred for 3 h at 65 °C, then treated with aqueous ammonia and extracted with CH_2_Cl_2_ (3 × 10 mL). The solvent was evaporated to dryness, and the residue was purified by column chromatography on Al_2_O_3_ (1 × 10 cm) with *n*-hexane/Et_2_O (5:1, v/v) as the eluent, collecting a colorless fraction with *R_f_* = 0.7–0.8 with blue fluorescence. Colorless oil, yield: 42 mg (80%). ^1^H NMR (400 MHz, CDCl_3_): δ = 2.61 (s, 3 H), 3.16 (s, 6 H), 7.27 (s, 1 H), 7.58 (ddd, *J* = 8.2, 6.8, 1.4 Hz, 1 H), 7.64 (ddd, *J* = 8.3, 6.8, 1.5 Hz, 1 H), 7.95 (dd, *J* = 8.4, 1.6 Hz, 1 H), 8.32 (dd, *J* = 8.3, 1.5 Hz, 1 H) ppm. ^13^C{^1^H} NMR (100 MHz, CDCl_3_): δ = 19.0, 44.6, 103.1, 119.7, 124.8, 125.9, 126.5, 128.1, 128.7, 130.8, 131.1, 135.6, 154.5 ppm. HRMS (ESI): *m*/*z* calcd. for C_14_H_15_N_2_^+^ [M + H^+^]: 211.1230, found 211.1228.

#### 1,5-Dimethyl-1H-Benzo[*g*]indazole **6a**

A solution of the corresponding oxime **4a** (0.25 mmol, 57 mg), HI (55% aqueous solution, 0.08 mL, 0.50 mmol, 2 equiv) in methanol (10 mL) was stirred for 3 h at 65 °C, then treated with aqueous ammonia and extracted with CH_2_Cl_2_ (3 × 10 mL). The solvent was evaporated to dryness, and the residue was purified by column chromatography on Al_2_O_3_ (1 × 10 cm) with *n*-hexane/Et_2_O (1:1, v/v) as the eluent, collecting a colorless fraction with *R_f_* = 0.7–0.8 with blue fluorescence. Colorless crystals with mp = 74–75 °C (Et_2_O), yield: 40 mg (81%). ^1^H NMR (400 MHz, CDCl_3_): δ = 2.68 (s, 3 H), 4.51 (s, 3 H), 7.50 (s, 1 H), 7.58–7.67 (m, 2 H), 7.92 (s, 1 H), 8.02–8.13 (m, 1 H), 8.42–8.50 (m, 1 H) ppm. ^13^C{^1^H} NMR (100 MHz, CDCl_3_): δ = 20.2, 40.9, 119.2, 121.3, 121.7, 122.2, 125.6, 125.8, 126.0, 127.8, 132.4, 132.8, 135.3 ppm. HRMS (ESI): *m*/*z* calcd. for C_13_H_13_N_2_^+^ [M + H^+^]: 197.1074, found 197.1071.

#### 5-Methylnaphtho[2,1-*d*]isoxazole **7a**

A solution of **4a** (57 mg, 0.25 mmol) in glacial acetic acid (10 mL) was stirred overnight at 65 °C, then treated with aqueous ammonia and extracted with CH_2_Cl_2_ (3 × 10 mL). The solvent was evaporated to dryness, and the residue was purified by column chromatography on Al_2_O_3_ (1 × 10 cm) with *n*-hexane/Et_2_O (5:1, v/v) as the eluent. The first colorless fraction with *R_f_* = 0.7–0.8 with blue fluorescence gave **5a** (28 mg, 53%). The second colorless fraction with *R_f_* = 0.5–0.6 with blue fluorescence gave **7a** (3 mg, 7%) as a colorless oil. ^1^H NMR (400 MHz, CDCl_3_): δ = 2.76 (d, *J* = 1.1 Hz, 3 H), 7.52 (d, *J* = 1.1 Hz, 1 H), 7.71–7.78 (m, 2 H), 8.09–8.14 (m, 1 H), 8.45–8.53 (m, 1 H), 8.75 (s, 1 H) ppm. ^13^C{^1^H} NMR (100 MHz, CDCl_3_): δ = 20.0, 116.4, 117.8, 119.4, 122.5, 125.3, 127.0, 128.2, 131.4, 133.5, 146.6, 160.4 ppm. HRMS (ESI): *m*/*z* calcd. for C_12_H_10_NO^+^ [M + H^+^]: 184.0757, found 184.0759.

#### *N,N*,1-Trimethyl-1H-Benzo[*g*]indol-9-Amine **8c**

The salt **4f**·HI (26 mg, 0.065 mmol) was dissolved in DMSO (650 μL) and heated at 100 °C for 19 h. Then, the resulting mixture was treated with KOH 5% aqueous solution (50 mL), then 100 mL of H_2_O, and extracted with AcOEt (3 × 10 mL). The organic extracts were combined and evaporated in vacuo. The crude product was purified by thin-layer chromatography on Al_2_O_3_ with *n*-hexane as the eluent. The fraction with *R_f_* = 0.8 and violet fluorescence yielded indole **8c** in 53% (8 mg). The spectroscopic data correspond to that of the previously reported.^31^^1^H NMR (400 MHz, CD_3_CN): δ = 2.67 (s, 6H), 4.04 (s, 3H), 6.60 (d, *J* = 3.0 Hz, 1H), 7.16 (dd, *J* = 7.6, 0.9 Hz, 1H), 7.21 (d, *J* = 3.0 Hz, 1H), 7.28–7.34 (m, 1H), 7.40 (d, *J* = 8.4 Hz, 1H), 7.52 (dd, *J* = 7.9, 0.9 Hz, 1H), 7.60 (d, *J* = 3.0 Hz, 1H) ppm.

## Data Availability

The data underlying this study are available in the published article and its Supporting Information.

## Supporting Information Available

Detailed experimental procedures, NMR spectra, and X-ray data for obtained compounds (PDF)
