# Supplementary material for: A Precise Synthetic Toolbox: H-Bond-Assisted Quadruple Reactivity of o-Dimethylaminoaryloximes
Source: J Org Chem. 2025 Mar 13;90(12):4374–81. doi: 10.1021/acs.joc.5c00207 (PMC11959518; doi:10.1021/acs.joc.5c00207)
Supplement: Supplementary file 1 — jo5c00207_si_001.pdf [file jo5c00207_si_001.pdf]

Supporting information for

A Precise Synthetic Toolbox: H-Bond-Assisted Quadruple Reactivity of o-Dimethylaminoaryloximes

Semyon V. Tsybulin[b], Stepan A. Meshalkin,[b] Daria I. Tonkoglazova,[b] Victor G. Bardakov,[b] Alexander F. Pozharskii,[c] and Alexander S. Antonov*[a]

[a] Institute of Organic Chemistry, University of Regensburg, D-93053 Regensburg, Germany
E-mail: Alexander.Antonov@chemie.uni-regensburg.de
[b] Institute of Chemistry, St. Petersburg State University, 198504 St. Petersburg, Russian Federation
[c] Department of Organic Chemistry, Southern Federal University, 344090 Rostov-on-Don, Russian Federation

Table of Contents

General S2
Transformation of *ortho*-dimethylaminoaryldoximes in acidic media (reaction conditions tests) S3
Synthetic procedures S9
Copies of ^{1}H and ^{13}C NMR spectra of newly obtained compounds S20
X-ray studies S81
References S88

General

Solvents used in organometallic reactions were dried over sodium-benzophenone. Unless otherwise stated, all the other solvents and commercial reagents were used without additional purification. An oil bath was used as the heat source. Reaction temperatures were reported as the temperatures of the bather surrounding the flasks or vials.

Liquid-state NMR experiments were performed using a Bruker Avance iii NMR spectrometer (400 MHz for ^{1}H and 100 MHz for ^{13}C) at the Center for Magnetic Resonance, St. Petersburg State University Research Park. Chemical shifts are referenced to TMS for ^{1}H and ^{13}C.

Single crystals of **4d**, **13a** and **14g** were grown by slow evaporation of Et_2O solution at +25 °C; **5e**, **6e**, **12a**, **7d** and **14a** – by slow evaporation of Et_2O solution at −25 °C. The single crystal X-ray diffraction data were collected using the SuperNova diffractometer equipped with a HyPix-3000 detector and a micro-focus Cu Ka radiation source (λ = 1.54184 Å) at temperature T = 100 (2) K or 120 K at the Centre for X-ray Diffraction Studies, St. Petersburg State University Research Park. Using Olex216, the structure was solved with the SHELXT structure solution program using Intrinsic Phasing and refined with the SHELXL refinement package using Least Squares minimization.

HR-ESI mass-spectra were obtained on a BRUKER maXis spectrometer equipped with an electrospray ionization (ESI) source; methanol was used as the solvent at the Chemical Analysis and Materials Research Centre, St. Petersburg State University Research Park. The instrument was operated in positive mode using an m/z range of 50–1200. The capillary voltage of the ion source was set at 4000 V. The nebulizer gas pressure was 1.0 bar, and the drying gas flow was set to 4.0 L/min.

Transformation of *ortho*-dimethylaminoaryldoximes in acidic media (reaction conditions tests)

Table S1. Transformation of *ortho*-dimethylaminobensaldoximes in acidic media.

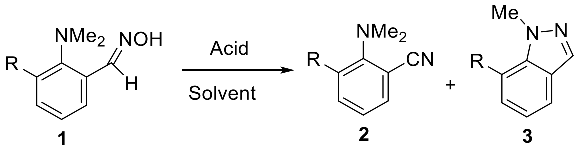

Run	Oxime	R	Acid	Solvent	T, °C	Time, h	Ratio **1**:**2**:**3**
1	**1a**	H	HBF_4	MeOH	65	3	1 : 0 : 0
2	**1a**	H	HBF_4	MeOH	65	12	1 : 0 : 0
3	**1a**	H	HCl	MeOH	65	12	1 : 0 : 0
4	**1a**	H	HI	MeOH	65	3	1 : 0 : 0
5	**1a**	H	HI	MeOH	65	12	1 : 0 : 0
6	**1a**	H	AcOH	AcOH	65	3	1 : 0 : 0[a]
7	**1a**	H	AcOH	AcOH	65	12	1 : 0 : 0
8	**1a**	H	HCl	DMSO	100	12	0 : 1 : 0.5
9	**1a**	H	HI	DMSO	100	12	0 : 1 : 7.7[b]
10	**1b**	TMS	HBF_4	MeOH	65	3	0.6 : 1 : 0
11	**1b**	TMS	HBF_4	acetone	65	3	0 : 1 : 0
12	**1b**	TMS	HCl	MeOH	65	3	0 : 0.14 : 1[c]
13	**1b**	TMS	HI	MeOH	65	3	0 : 0 : 1
14	**1b**	TMS	AcOH	AcOH	65	3	1 : 0 : 0
15	**1b**	TMS	HBF_4	DMSO	100	12	0 : 1 : 0[d]
16	**1b**	TMS	HCl	DMSO	100	12	0 : 0 : 0[e]
17	**1b**	TMS	HI	DMSO	100	12	0 : 0 : 1[f]
18	**1c**	Br	HBF_4	MeOH	65	3	0 : 1 : 0.5
19	**1c**	Br	HBF_4	acetone	65	12	0 : 1 : 0
20	**1c**	Br	HI	MeOH	65	3	0 : 0 : 1
21	**1c**	Br	AcOH	AcOH	65	3	8.5 : 1 : 0
22	**1c**	Br	AcOH	AcOH	65	12	2.2 : 1 : 0
23	**1c**	Br	AcOH	AcOH	90	3	1 : 1 : 0
24	**1c**	Br	AcOH	acetone	65	3	1 : 0 : 0
25	**1c**	Br	HCl	DMSO	100	12	0 : 1 : 6
26	**1c**	Br	$FeCl_3$	MeOH	65	3	0 : 1 : 1.1
27	**1d**	Me	HBF_4	acetone	65	3	1 : 0 : 0
28	**1d**	Me	HBF_4	MeOH	65	3	0.5 : 1 : 0
29	**1d**	Me	HBF_4	MeOH	65	48	0 : 0 : 0[g]
30	**1d**	Me	HI	MeOH	65	3	1 : 1 : 3.3
31	**1d**	Me	AcOH	AcOH	65	3	5.5 : 1 : 0

32	**1d**	Me	HCl	DMSO	100	12	0 : 1 : 3.3
33	**1e**	SMe	HBF_4	acetone	65	3	1 : 1 : 0
34	**1e**	SMe	HBF_4	acetone	65	6	0.4 : 1 : 0[h]
35	**1e**	SMe	HBF_4	acetone	65	48	0 : 1 : 0
36	**1e**	SMe	HBF_4	MeOH	65	3	0 : 1 : 0.3
37	**1e**	SMe	HI	MeOH	65	3	0 : 0 : 1
38	**1e**	SMe	AcOH	AcOH	65	3	4 : 1 : 0
39	**1e**	SMe	AcOH	AcOH	65	24	0.3 : 1 : 0
40	**1e**	SMe	HBF_4	DMSO	100	12	0 : 1 : 0.3[i]
41	**1e**	SMe	HCl	DMSO	100	12	0 : 1 : 3.3[i]
42	**1e**	SMe	HI	DMSO	100	12	0 : 0 : 1[i]
43	**1e**	SMe	$FeCl_3$	MeOH	65	3	0 : 1 : 1.1

[a] Additional minor products were also detected. [b] The reaction mixture is very complex, but **2a** and **3a** are clearly detected. [c] Under these conditions TMS group is partially removed resulting in the formation of products **3b**, **3a** and in a ratio 1 : 0.7. [d] Under these conditions TMS group is partially removed resulting in the formation of products **2b**, **2a** and **3a** in a ratio 1 : 1.3 : 1. [e] Under these conditions TMS group is removed resulting in the formation of products **2a** and **3a** in a ratio 1 : 1.25. [f] Under these conditions TMS group is partially removed resulting in the formation of products **3b** and **3a** in a ratio 1 : 0.6, additional minor products were also detected. [g] The reaction mixture is very complex, neither **2d** nor **3d** are detected. [h] Corresponding **aldehyde**, **1e** and **2e** were detected in a ratio 0.01 : 0.4 : 1. [i] Additional minor products were also detected

Table S2. Transformation of *ortho*-dimethylaminonaphthaloximes in acidic media.

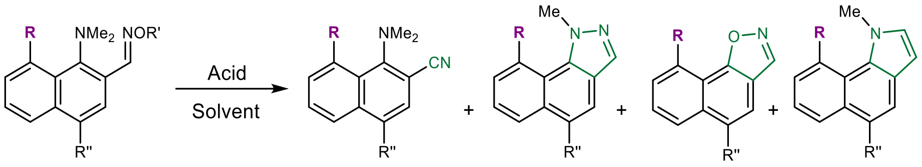

4 5 6 7 8

Run	Oxime	R	R’	R’’	Acid	Solvent	T, °C	Time, h	Products ratio 4	5	6	7	8
1	**4a**	H	H	Me	HBF_4	acetone	65	12	-	1	-	-	-
2	**4a**	H	H	Me	HBF_4	acetone	65	3	0.4	1	-	-	-
3	**4a**	H	H	Me	HI	MeOH	65	3	-	-	1	-	-
4	**4a**	H	H	Me	HBF_4	MeOH	65	3	0.2	1	0.1	-	-
5	**4a**	H	H	Me	AcOH	AcOH	65	12	0.1	1	-	0.8	-
6	**4a**	H	H	Me	AcOH	AcOH	65	3	2.2	1	-	0.9	-
7	**4a**	H	H	Me	AcOH	acetone	65	3	15	1	-	0.6	-
8	**4a**	H	H	Me	$FeCl_3$	MeOH	65	3	0.1	-	0.4	1	-
9	**4a**	H	H	Me	$FeCl_3$	acetone	65	3	0.2	1	0.1	0.1	-
10	**4a**	H	H	Me	$AlCl_3$	MeOH	65	12	-	0.1	1	-	-
11	**4a**	H	H	Me	$AlCl_3$	acetone	65	3	-	1	0.6	-	-
12	**4a**	H	H	Me	HBF_4	THF	65	12	0.2	1	0.2	0.1	-
13	**4a**	H	H	Me	HBF_4	benzene	65	3	1.4[a]	0.1	-	0.1	-
14	**4a**	H	H	Me	HBF_4	DMSO	65	3	0.7	0.6	1	-	-
15	**4a**	H	H	Me	HI	DMSO	65	3	-	0.4	1	-	-
16	**4a**	H	H	Me	HCl	DMSO	65	12	-	-	1	-	-
17[b]	**4a**	H	H	Me	-	-	115	1	1	-	-	-	-
18	**4b**	TMS	H	Me	HBF_4	acetone	65	9	-	-	-	-	1
19	**4b**	TMS	H	Me	HI	MeOH	65	9	-	-	-	0.9	1
20	**4b**	TMS	H	Me	HCl	MeOH	65	9	-	-	-	1.9	1
21	**4b**	TMS	H	Me	HBF_4	MeOH	65	9	-	-	-	1.6	1
22	**4b**	TMS	H	Me	AcOH	AcOH	65	9	0.5	-	-	1	-
23	**4b**	TMS	H	Me	AcOH	acetone	65	3	1	-	-	-	-
24[c]	**4b**	TMS	H	Me	HCl	DMSO	100	12	-	-	-	-	-

25[d]	**4c**	NMe_2	H	H	HBF_4	acetone	65	12	1	-	-	-	-
26	**4c**	NMe_2	H	H	AcOH	AcOH	65	3	1	-	-	-	-
27	**4c**	NMe_2	H	H	AcOH	AcOH	65	18	1	-	-	-	-
28	**4c**	NMe_2	H	H	AcOH	AcOH	90	4	1	-	-	-	-
29[d]	**4c**	NMe_2	H	H	HI	MeOH	65	3	1	-	-	-	-
30[d]	**4c**	NMe_2	H	H	HI	EtOH	85	61	1	-	-	-	0.05
31[d]	**4c**	NMe_2	H	H	HI	*n*-BuOH	100	81	-	-	-	-	1
32 [d]	**4c**	NMe_2	H	H	HI	DMSO	100	22	-	-	-	-	1
33 [d]	**4c**	NMe_2	H	H	HBF_4	DMSO	100	22	1	-	-	-	0.35
34	**4c**	NMe_2	H	H	HCl	DMSO	100	72	-	1	-	-	-
35	**4d**	SMe	H	Me	HBF_4	acetone	65	3	-	1	-	-	-
36	**4d**	SMe	H	Me	HBF_4	MeOH	65	3	-	0.2	0.2	1	-
37	**4d**	SMe	H	Me	HI	MeOH	65	3	-	-	1	-	-
38[c]	**4d**	SMe	H	Me	AcOH	AcOH	65	12	0.25	-	-	1	-
39	**4d**	SMe	H	Me	AcOH	AcOH	65	6	1.6	-	-	1	-
40	**4d**	SMe	H	Me	AcOH	AcOH	65	3	1	-	-	1	-
41	**4d**	SMe	H	Me	AcOH	acetone	65	3	1	-	-	-	-
42[c]	**4d**	SMe	H	Me	HCl	DMSO	100	12	-	1	-	-	-
43	**4e**	Me	H	Me	HBF_4	acetone	65	3	-	1	-	-	-
44	**4e**	Me	H	Me	HI	MeOH	65	3	-	-	1	0.1	-
45	**4e**	Me	H	Me	HBF_4	MeOH	65	3	-	1	0.5	0.4	-
46	**4e**	Me	H	Me	AcOH	AcOH	65	3	-	-	-	1	-
47	**4e**	Me	H	Me	AcOH	acetone	65	3	1	-	-	-	-
48	**4e**	Me	H	Me	HCl	DMSO	100	14	-	0.6	1	-	-
49	**4e**	Me	H	Me	$FeCl_3$	MeOH	65	3	0.5	-	-	1	-

[a] Total amount of **4a** and a form of a corresponding aldehyde. [b] Pure compound **4a** was melted. [c] Tarring. [d] Salt **4c·HI** was isolated before use in the reaction.

Table S3. Transformation of *ortho*-dimethylaminoarylketoximes in acidic media.

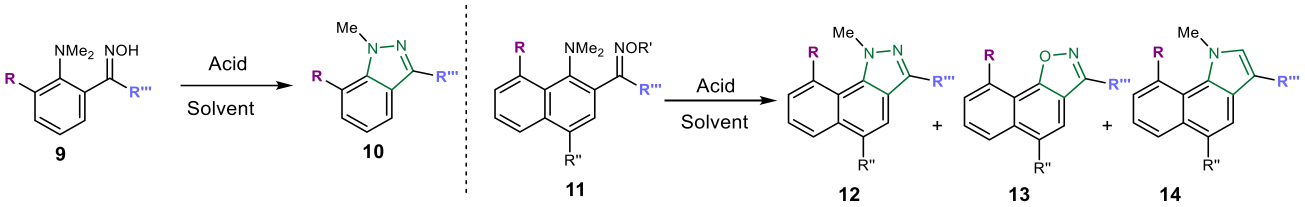

Run	Oxime	R	R'	R''	R'''	Acid	Solvent	T, °C	Time, h	Products ratio			
										**9(11)**	**10(12)**	**13**	**14**
1	**9a**	H	-	-	p-Tol	HI	MeOH	65	36	1	-	-	-
2	**9a**	H	-	-	p-Tol	HI	MeOH	65	12	1	-	-	-
3	**9a**	H	-	-	p-Tol	HI	n-BuOH	100	24	1	-	-	-
4	**9a**	H	-	-	p-Tol	HI	n-BuOH	100	48	1	0.1	-	-
5	**9a**	H	-	-	p-Tol	HI	DMSO	100	24	1[c]	-	-	-
6	**9b**	TMS	-	-	p-Tol	HI	MeOH	65	24	1.4[a]	1	-	-
7	**9b**	TMS	-	-	p-Tol	HI	MeOH	65	12	4[b]	1	-	-
8	**9b**	TMS	-	-	p-Tol	HI	MeOH	65	3	10.2[a]	1	-	-
9	**9b**	TMS	-	-	p-Tol	HCl	DMSO	100	70	1[b]	1	-	-
10	**9b**	TMS	-	-	p-Tol	AcOH	AcOH	65	3	1[a]	-	-	-
11	**9b**	TMS	-	-	p-Tol	AcOH	AcOH	65	12	1[b]	-	-	-
12	**11a**	H	H	Me	p-Tol	HI	MeOH	65	72	-	0.6	1	-
13	**11a**	H	H	Me	p-Tol	HI	MeOH	65	48	1	0.6	1	-
14	**11a**	H	H	Me	p-Tol	HI	MeOH	65	12	6.3	0.6	1	-
15	**11a**	H	H	Me	p-Tol	HI	MeOH	65	3	8	0.6	1	-
16[c]	**11a**	H	H	Me	p-Tol	AcOH	AcOH	65	12	0.2	-	1	-
17[c]	**11a**	H	H	Me	p-Tol	AcOH	AcOH	65	3	2	-	1	-
18[c]	**11a**	H	H	Me	p-Tol	HCl	DMSO	100	12	3	-	1	-
19	**11b**	H	H	Me	n-Bu	HI	MeOH	65	48	-	1	-	-
20	**11c**	TMS	H	Me	p-Tol	HI	MeOH	65	20	-	-	1	-
21[d]	**11d**	NMe_2	H	H	Ph	HCl	DMSO	100	72	-	-	1	-
22[d]	**11g**	NMe_2	Me	H	Ph	HI	DMSO	100	21	-	-	-	1
23[d]	**11g**	NMe_2	Me	H	Ph	HI	n-BuOH	100	70	-	-	-	1
24[d]	**11h**	NMe_2	Me	H	p-Tol	HI	DMSO	100	21	-	-	-	1

25[d]	**11h**	NMe_2	Me	H	p-Tol	HI	n-BuOH	100	70	-	-	-	1
26[e]	**11e**	Me	H	Me	p-Tol	HCl	EtOH	85	24	-	-	1	-
27[f]	**11f**	H	Me	Me	p-Tol	HCl	EtOH	85	24	-	-	-	1

[a] Total amount of **9b** and **9a** due to the desilylation. [b] In a form of **9a** due to the desilylation. [c] Tarring. [d] Pure **11**·HX was heated in the solvent; [e] oxime **11e** was not isolated, the reaction of the corresponding imine in the provided conditions with hydroxylamine hydrochloride gives **13e** as the only product; [f] O-Me oxime **11f** was not isolated, the reaction of the corresponding imine in the provided conditions with methoxyamine hydrochloride gives **14a** as the only product.

Table S4. Mechanistic studies.

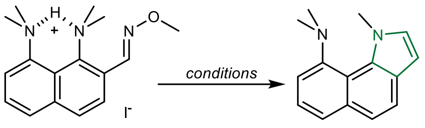

4c·HI **8c**

Run		Solvent	T, °C	Additive	Other	Time, h	Products ratio	
							**4c**	**8c**
1	Experiment	n-BuOH	100	–	argon	81	1	0–0.15
	Control	n-BuOH	100	–	air	81	0	1
2	Experiment	n-BuOH	100	TEMPO	air	18	0	1
	Control	n-BuOH	100	–	air	18	1	0.29
3	Experiment	n-BuOH	65	TEMPO	air	18	1	0.04
	Control	n-BuOH	65	-	air	18	1	0
4	Experiment	n-BuOH	100	KI	air		1	0.57-0.59
	Control	n-BuOH	100	–	air		1	0.43

Synthetic procedures

2-(Dimethylamino)benzaldehyde oxime (*general procedure*): A solution of corresponding benzaldehyde (1 mmol: 126 mg of **24b**[1], 242 mg of **24c**[1], 210 mg of **24d**[1]), $NH_2OH{\cdot}HCl$ (209 mg, 3 mmol, 3 equiv.), and Et_3N (0.7 mL) in methanol was stirred for 24 h at 65 °C. The solvent was evaporated to dryness, the residue was treated with water and extracted with CH_2Cl_2 (3×20 mL). The combined organic extracts were dried over Na_2SO_4 and filtered off. The solvent was evaporated to dryness.

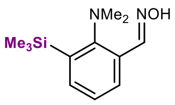

1b

2-(Dimethylamino)-3-(trimethylsilyl)benzaldehyde oxime 1b: Brownish solid, yield: 200 mg (88%). ^1H NMR (400 MHz, $CDCl_3$): *δ*= 0.30 (s, 9H), 2.86 (s, 6H), 7.27–7.21 (m, 1H), 7.55 (dd, *J* = 7.3, 1.7 Hz, 1H), 7.80 (dd, *J* = 7.7, 1.7, 1H), 8.37 (s, 1H), 8.51 (s, 1H) ppm. $^{13}C\{^1H\}$ NMR (100 MHz, $CDCl_3$): *δ*= −0.04, 45.6, 126.1, 128.9, 131.4, 137.4, 142.4, 148.6, 157.4 ppm. HRMS (ESI): *m/z* calcd. for $C_{12}H_{21}N_2OSi^+$ [M+H$^+$]: 237.1418, found 237.1419.

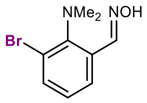

1c

3-Bromo-2-(dimethylamino)benzaldehyde oxime 1c: Yellowish solid, yield: 150 mg (62%). ^1H NMR (400 MHz, $CDCl_3$): *δ*= 2.91 (s, 6H), 6.99–7.08 (m, 1H), 7.57 (dd, *J* = 7.9, 1.5 Hz, 1H), 7.71 (dd, *J*=7.8, 1.5 Hz, 1H), 8.36 (s, 1H), 8.55 (s, 1H) ppm. $^{13}C\{^1H\}$ NMR (100 MHz, $CDCl_3$): *δ* = 42.9, 123.8, 125.8, 126.7, 133.8, 135.7, 148.8, 149.6 ppm. HRMS (ESI): *m/z* calcd. for $C_9H_{12}{}^{79}BrN_2O^+$ [M+H$^+$]: 243.0128, found 243.0130, *m/z* calcd. for $C_9H_{12}{}^{81}BrN_2O^+$ [M+H$^+$]: 245.0108, found 245.0116.

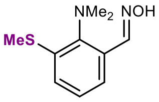

1d

2-(Dimethylamino)-3-(methylthio)benzaldehyde oxime 1d: The product was purified by recrystallisation from *n*-heptane. The powder was dissolved in Et_2O (5 mL) and solvent was slowly evaporated (rt). The compound **1d** was obtained as orange solid, yield: 130 mg (62%). ^1H NMR (400 MHz, $CDCl_3$): *δ*= 2.41 (s, 3H), 2.90 (s, 6H), 7.09–7.23 (m, 2H), 7.52 (dd, *J* = 7.5, 1.8 Hz, 1H), 8.09 (s, 1H), 8.45 (s, 1H) ppm. $^{13}C\{^1H\}$ NMR (100 MHz, $CDCl_3$): *δ* = 14.9, 43.1, 122.8, 125.9, 126.2, 131.5, 141.2, 148.0, 148.6 ppm. HRMS (ESI): *m/z* calcd. for $C_{10}H_{15}N_2OS^+$ [M+H$^+$]: 211.0900, found 211.0901.

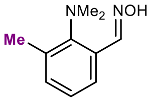

1e

2-(Dimethylamino)-3-methylbenzaldehyde oxime 1e: A solution of **24e**[1] (163 mg, 1 mmol), $NH_2OH{\cdot}HCl$ (209 mg, 3 mmol, 3 equiv.) in pyridine (20 mL) was stirred for 24 h at 65 °C. The solvent was evaporated to dryness, the residue was treated with water and extracted with CH_2Cl_2 (3×20 mL). The combined organic extracts were dried over Na_2SO_4 and filtered off. The solvent was evaporated to dryness. Compound **1e** was obtained as a pale-yellow oil. Yield: 156 mg (88 %). ^1H NMR (400 MHz, $CDCl_3$): *δ*= 2.35 (s, 3H), 2.89 (s, 6H), 7.02–7.12 (m, 1H), 7.19 (dd, *J* = 7.5, 1.7 Hz, 1H), 7.59 (dd, *J* = 7.7, 1.7 Hz, 1H), 8.56 (s, 1H), 8.16–9.15 (s, 1H) ppm. $^{13}C\{^1H\}$ NMR (100 MHz, $CDCl_3$): *δ* = 19.2, 43.5, 124.5, 125.2, 130.8, 133.4, 137.3, 149.6, 150.2 ppm. HRMS (ESI): *m/z* calcd. for $C_{10}H_{15}N_2O^+$ [M+H$^+$]: 179.1179, found 179.1180.

2-(Dimethylamino)benzonitriles (*general procedure*): A solution of corresponding oxime (0.5 mmol: 118 mg of **1b**, 121 mg of **1c**, 105 mg of **1d**), HBF_4 (50%, 63 µL, 1 mmol, 2 equiv.) in acetone was stirred for 3, 12 or 48 h at 65 °C. The solvent was evaporated to dryness, the residue was treated with water and extracted with CH_2Cl_2 (3×20 mL). The combined organic extracts were dried over Na_2SO_4 and filtered off. The solvent was evaporated to dryness.

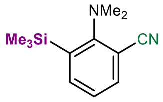

2b

2-(Dimethylamino)-3-(trimethylsilyl)benzonitrile 2b: A solution was stirred for 3 h at 65 °C. A compound **2b** was obtained as brown oil. Yield: 103 mg (95%). ^1H NMR (400 MHz, $CDCl_3$): *δ*= 0.30 (s, 9H), 2.90 (s, 6H), 7.24–7.33 (m, 1H), 7.63 (dd, *J* = 7.6, 1.7 Hz, 1H), 7.71 (dd, *J* = 7.5, 1.7 Hz, 1H) ppm. $^{13}C\{^1H\}$ NMR (100 MHz, $CDCl_3$): *δ* = −0.4, 44.1, 110.3, 118.6, 123.8, 136.0, 140.1, 143.3, 161.9 ppm. HRMS (ESI): *m/z* calcd. for $C_{12}H_{19}N_2Si^+$ [M+H$^+$]: 219.1312, found 219.1313.

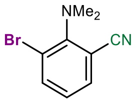

2c

3-Bromo-2-(dimethylamino)benzonitrile 2c: A solution was stirred for 12 h at 65 °C. A compound **2c** was obtained as brown oil. Yield: 101 mg (90%). ^1H NMR (400 MHz, $CDCl_3$): *δ*= 3.06 (s, 3H), 6.96–7.03 (m, 1H), 7.53 (dd, *J* = 7.7, 1.6 Hz, 1H), 7.76 (dd, *J* = 8.0, 1.6 Hz, 1H) ppm. $^{13}C\{^1H\}$ NMR (100 MHz, $CDCl_3$): *δ*= 43.1, 111.3, 117.7, 122.9, 124.8, 133.7, 138.6, 153.9 ppm. HRMS (ESI): *m/z* calcd. for $C_9H_{10}{}^{79}BrN_2^+$ [M+H$^+$]: 225.0022, found 225.0022, *m/z* calcd. for $C_9H_{10}{}^{81}BrN_2^+$ [M+H$^+$]: 227.0002, found 226.9991.

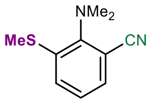

2d

2-(Dimethylamino)-3-(methylthio)benzonitrile 2d: A solution was stirred for 48 h at 65 °C. A compound **2d** was obtained as brown oil. Yield: 83 mg (86%). ^1H NMR (400 MHz, $CDCl_3$): *δ*= 2.40 (s, 3H), 2.98 (s, 6H), 7.12–7.24 (m, 1H), 7.24–7.31 (m, 1H), 7.34 (dd, *J* = 7.6, 1.6 Hz, 1H) ppm. $^{13}C\{^1H\}$ NMR (100 MHz, $CDCl_3$): *δ*= 29.7, 42.7, 109.5, 118.2, 125.4, 128.1, 129.7, 141.3, 152.0 ppm. HRMS (ESI): *m/z* calcd. for $C_{10}H_{13}N_2S^+$ [M+H$^+$]: 193.0794, found 193.0791

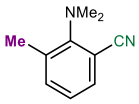

2e

2-(Dimethylamino)-3-methylbenzonitrile 2e: A solution of oxime **1e** (139 mg, 0.5 mmol), HBF_4 (50%, 63 µL, 1 mmol, 2 equiv.) in methanol was stirred for 12 h at 65 °C. The solvent was evaporated to dryness, the residue was treated with water and extracted with CH_2Cl_2 (3×20 mL). The combined organic extracts were dried over Na_2SO_4 and filtered off. The solvent was evaporated to dryness. The product was purified by a thin layer chromatography on silica gel with *n*-hexane/CH_2Cl_2 (1:1, v/v) as the eluent. The yellow fraction with R_f = 0.7 gave compound **2e** as brown oil. Yield: 44 mg (55%). ^1H NMR (400 MHz, $CDCl_3$): *δ*= 2.33 (s, 3H), 2.99 (s, 6H), 7.01–7.09 (m, 1H), 7.36 (dd, *J* = 7.5, 1.6 Hz, 1H), 7.41 (dd, *J* = 7.7, 1.7 Hz, 1H) ppm. $^{13}C\{^1H\}$ NMR (100 MHz, $CDCl_3$): δ= 18.8, 43.1, 109.2, 119.1, 123.9, 132.2, 135.7, 136.8, 154.7 ppm. HRMS (ESI): *m/z* calcd. for $C_{10}H_{13}N_2^+$ [M+H$^+$]: 161.1073, found 161.1079

1-Methyl-1*H*-indazoles (*general procedure*): A solution of corresponding oxime (0.5 mmol: 118 mg of **1b**, 121 mg of **1c**, 105 mg of **1d**, 139 mg of **1e**), HI (55% aqueous solution, 137 µL, 1 mmol, 2 equiv.) in methanol was stirred for 3 h at 65 °C. The solvent was evaporated to dryness, the residue was treated with water and extracted with CH_2Cl_2 (3×20 mL). The combined organic extracts were dried over Na_2SO_4 and filtered off. The solvent was evaporated to dryness.

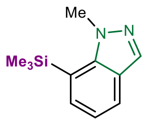

3b

1-Methyl-7-(trimethylsilyl)-1*H*-indazole 3b: Brown oil. Yield: 98 mg (96%). ^{1}H NMR (400 MHz, $CDCl_3$): *δ*= 0.52 (s, 9H), 4.29 (s, 3H), 7.14 (dd, *J* = 8.0, 6.9 Hz, 1H), 7.59 (dd, *J* = 6.9, 1.2 Hz, 1H), 7.78 (dd, *J* = 8.1, 1.2 Hz, 1H), 8.04 (s, 1H) ppm. ^{13}C{^{1}H} NMR (100 MHz, $CDCl_3$): *δ*= 1.7, 39.3, 120.0, 120.7, 122.7, 123.9, 133.6, 134.3, 144.0 ppm. HRMS (ESI): *m/z* calcd. for $C_{11}H_{17}N_2Si^+$ [M+H$^+$]: 205.1156, found 205.1153.

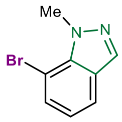

3c

7-Bromo-1-methyl-1*H*-indazole 3c: Brown oil. Yield: 100 mg (95%). ^{1}H NMR (400 MHz, $CDCl_3$): *δ*= 4.44 (s, 3H), 6.93–7.02 (m, 1H), 7.55 (d, *J* = 7.3 Hz, 1H), 7.67 (d, *J* = 8.0 Hz, 1H), 7.98 (s, 1H) ppm. ^{13}C{^{1}H} NMR (100 MHz, $CDCl_3$): *δ*= 39.1, 120.5, 121.6, 126.6, 129.7, 130.8, 132.7, 137.3 ppm. HRMS (ESI): *m/z* calcd. for $C_8H_8{}^{79}BrN_2^+$ [M+H$^+$]: 210.9866, found 210.9847, *m/z* calcd. for $C_8H_8{}^{81}BrN_2^+$ [M+H$^+$]: 212.9845, found 212.9867.

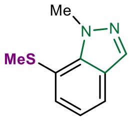

3d

1-Methyl-7-(methylthio)-1*H*-indazole 3d: Brown oil. Yield: 83 mg (92%). ^{1}H NMR (400 MHz, $CDCl_3$): *δ*= 2.55 (s, 3H), 4.50 (s, 3H), 7.05–7.16 (m, 1H), 7.26–7.34 (m, 1H), 7.57 (dd, *J* = 8.0, 1 Hz, 1H), 7.96 (s, 1H) ppm. ^{13}C{^{1}H} NMR (100 MHz, $CDCl_3$): *δ*= 18.3, 39.4, 119.3, 120.4, 121.2, 125.2, 127.3, 132.9, 138.6 ppm. HRMS (ESI): *m/z* calcd. for $C_9H_{11}N_2S^+$[M+H$^+$]:179.0638, found 179.0637.

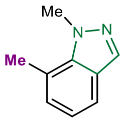

3e

1,7-Dimethyl-1*H*-indazole 3e: The product was purified by a thin layer chromatography on silica gel with *n*-hexane/ CH_2Cl_2 (1:4, v/v) as the eluent. The colorless fraction with R_f = 0.1 gave compound **3e** as brown oil. Yield: 36 mg (31%). ^{1}H NMR (400 MHz, $CDCl_3$): *δ*= 2.80 (s, 3H), 4.35 (s, 3H), 7.02 (dd, *J* = 8.0, 6.9 Hz, 1H), 7.08–7.12 (m, 1H), 7.57 (d, *J* = 8.0 Hz, 1H), 7.94 (s, 1H) ppm. ^{13}C{^{1}H} NMR (100 MHz, $CDCl_3$): *δ* = 19.3, 39.1, 119.0, 120.3, 120.8, 124.9, 127.9, 132.7, 139.4 ppm. HRMS (ESI): *m/z* calcd. for $C_9H_{11}N_2^+$ [M+H$^+$]: 147.0917, found 147.0926

1-(Dimethylamino)-4-methyl-2-naphthaldehyde oximes (*general procedure*): A solution of corresponding naphthaldehyde (1 mmol: 213 mg of **26a**, 285 mg of **26b**, 259 mg of **26d**, 227 mg of **26e**), $NH_2OH·HCl$ (139 mg, 2 mmol, 2 equiv.) and Et_3N (0.5 mL) in methanol (15 mL) was stirred for 24 h at 65 °C. The solvent was evaporated to dryness, and the residue was purified by a thin layer chromatography on Al_2O_3. The colourless fraction with blue fluorescence gave the corresponding product.

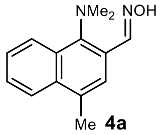

4a

1-(Dimethylamino)-4-methyl-2-naphthaldehyde oxime 4a: Colourless crystals with mp = 113−115 °C (Et_2O), R_f = 0.6 (*n*-hexane), yield: 194 mg, (85%). ^{1}H NMR (400 MHz, $CDCl_3$): *δ*= 2.66 (d, *J* = 1.1 Hz, 3 H), 3.10 (s, 6 H), 7.49–7.59 (m, 2 H), 7.73 (d, *J* = 1.1 Hz, 1 H), 7.93–8.03 (m, 1 H), 8.12–8.19 (m, 1 H), 8.66 (s, 1 H), 8.72 (s, 1 H) ppm. ^{13}C{^{1}H} NMR (100 MHz, $CDCl_3$): *δ*= 19.4, 45.1, 123.7, 125.2, 125.5, 125.8, 126.6, 127.4, 132.4, 132.7, 135.1, 148.0, 149.4 ppm. HRMS (ESI): *m/z* calcd. for $C_{14}H_{17}N_2O^+$ [M+H$^+$]: 229.1336, found 229.1341.

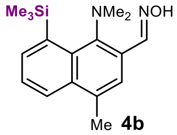

4b

1-(Dimethylamino)-4-methyl-8-(trimethylsilyl)-2-naphthaldehyde oxime 4b: Pale yellow crystals with mp = 142−143 °C ($CHCl_3$), R_f = 0.3 (*n*-hexane/Et_2O (3:1, v/v)), yield: 207 mg, (69%). ^{1}H NMR (400 MHz, $CDCl_3$): *δ*= 0.29 (s, 9 H), 2.64 (s, 3 H), 2.83 (s, 6 H), 7.48 (dd, *J* = 8.2, 6.8 Hz, 1 H), 7.59 (s, 1 H), 7.86–7.95 (m, 2 H), 8.38 (s, 1 H), 8.79 (s, 1 H) ppm. ^{13}C{^{1}H} NMR (100 MHz, $CDCl_3$): *δ*= 2.6, 19.6, 48.2, 125.5, 125.8, 125.9, 126.0, 132.4, 135.0, 136.5, 136.9, 138.6, 149.6, 150.0 ppm. HRMS (ESI): *m/z* calcd. for $C_{17}H_{25}N_2OSi^+$ [M+H$^+$]: 301.1731, found 301.1730.

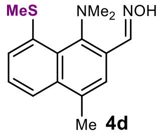

4d

1-(Dimethylamino)-4-methyl-8-(methylthio)-2-naphthaldehyde oxime 4d: Pale yellow crystals with mp = 151−153 °C (CH_2Cl_2), R_f = 0.7 (*n*-hexane/Et_2O (1:1, v/v)), yield: 227 mg, (83%). ^{1}H NMR (400 MHz, $CDCl_3$): *δ*= 2.45 (s, 3 H), 2.67 (s, 3 H), 2.87 (s, 6 H), 7.28 (d, *J* = 7.1 Hz, 1 H), 7.45–7. 52 (m, 1 H), 7.70 (dd, *J* = 8.3, 1.1 Hz, 1 H), 7.80 (s, 1 H), 8.57 (s, 1 H), 8.93 (s, 1 H) ppm. ^{13}C{^{1}H} NMR (100 MHz, $CDCl_3$): *δ*= 17.8, 20.3, 44.9, 120.2, 121.2, 124.4, 126.7, 129.4, 131.0, 133.4, 135.9, 139.3, 147.2, 148.4 ppm. HRMS (ESI): *m/z* calcd. for $C_{15}H_{19}N_2OS^+$ [M+H$^+$]: 275.1213, found 275.1213.

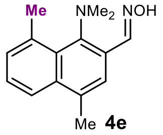

4e

1-(Dimethylamino)-4,8-dimethyl-2-naphthaldehyde oxime 4e: Pale yellow crystals with mp = 111−113 °C (Et_2O), R_f = 0.3 (*n*-hexane/Et_2O (5:1, v/v)), yield: 174 mg, (72%). ^{1}H NMR (400 MHz, $CDCl_3$): *δ*= 2.65 (s, 3H), 2.87 (s, 3 H), 2.91 (s, 6 H), 7.33 (d, *J* = 7.0 Hz, 1 H), 7.40 (dd, *J* = 8.3, 7.0 Hz, 1 H), 7.75 (s, 1 H), 7.83 (d, *J* = 8.3 Hz, 1 H), 8.47 (s, 1 H), 8.59 (s, 1 H) ppm. ^{13}C{^{1}H} NMR (100 MHz, $CDCl_3$): *δ*= 20.4, 23.9, 44.9, 123.4, 124.1, 126.3, 130.4, 130.6, 133.0, 133.3, 136.2, 148.4, 149.2 ppm. HRMS (ESI): *m/z* calcd. for $C_{15}H_{19}N_2O^+$ [M+H$^+$]: 243.1492, found 243.1491.

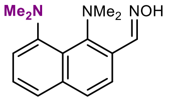

4c

1,8-Bis(dimethylamino)-2-naphthaldehyde oxime 4c: Compound **4c** was obtained similarly to a previously described procedure with slight modifications.[2] A mixture of 1,8-bis(dimethylamino)-2-naphthaldehyde **26c** (442 mg,1.83 mmol) and $NH_2OH·HCl$ (254 mg, 3.66 mmol, 2 equiv.) in 20 mL of EtOH was stirred for 6 h and then the resulting mixture was evaporated to dryness. The residue was treated with 1 % KOH aq. solution (50 mL), extracted with CH_2Cl_2 (3×15 mL). The extract was dried over Na_2SO_4 and evaporated. Compound **4c** was purified by washing the rection mixture with *n*-hexane. The yellow solid was then filtered off giving **4c** in 76% yield (355 mg). The spectroscopic ^{1}H NMR data corresponds to that of the previously reported.[2] ^{1}H NMR (400 MHz, DMSO-*d6*): δ= 2.70 (s, 6H), 2.98 (s, 6H), 7.10 (d, *J* = 7.5 Hz, 1H), 7.30–7.35 (m, 1H), 7.41 (d, *J* = 8.0 Hz, 1H), 7.47 (d, *J* = 8.6 Hz, 1H), 7.65 (d, *J* = 8.6 Hz, 1H), 8.28 (s, 1H), 11.12 (s, 1H) ppm.

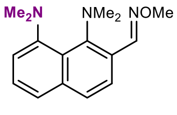

4f

1,8-Bis(dimethylamino)-2-naphthaldehyde *O*-methyl oxime 4f: A mixture of 1,8-bis(dimethylamino)-2-naphthaldehyde **26c** (128 mg, 0.53 mmol) and $NH_2OMe{\cdot}HCl$ (88 mg, 1.06 mmol, 2 equiv.) in 20 mL of EtOH was stirred for 6 h and then the resulting mixture was evaporated to dryness. The residue was treated with 1 % KOH aq. solution (50 mL), extracted with CH_2Cl_2 (3×15 mL). The extract was dried over Na_2SO_4 and evaporated. Compound **4f** was purified by a thin layer chromatography on Al_2O_3 with *n*-hexane/AcOEt (50 : 1, v/v) as the eluent. The yellow fraction with R_f = 0.8–0.9 yielded 84 % of **4f** as yellow oil. ^{1}H NMR (400 MHz, $CDCl_3$): δ= 2.77 (s, 6H), 3.08 (s, 6H), 4.04 (s, 3H), 7.09 (dd, *J* = 7.4, 1.1, 1H), 7.30–7.36 (m, 1H), 7.40 (dd, *J* = 7.9, 1.1, 1H), 7.46 (d, *J* = 8.6, 1H), 7.79 (d, *J* = 8.6, 1H), 8.40 (s, 1 H) ppm. ^{13}C{^{1}H} NMR (100 MHz, $CDCl_3$): δ= 45.5, 45.8, 61.9, 115.1, 123.1, 124.5, 124.8, 124.9, 126.3, 126.5, 138.5, 149.3, 149.5, 152.3 ppm. HRMS (ESI): *m/z* calcd. for $C_{16}H_{22}N_3O^+$ [M+H$^+$]: 272.1757, found 272.1763.

1-(Dimethylamino)-4-methyl-2-naphthonitriles (*general procedure*): A solution of corresponding naphthaldehyde oxime (0.25 mmol: 57 mg of **4a**, 68 mg of **4d**, 60 mg of **4e**), HBF_4 (50% aqueous solution, 0.12 mL, 0.50 mmol, 2 equiv.) in acetone (10 mL) was stirred for 3 h (overnight for **4a**) at 65 °C, then treated with aqueous ammonia and extracted with CH_2Cl_2 3×10 mL. The solvent was evaporated to dryness, and the residue was purified by a thin layer chromatography on Al_2O_3 with *n*-hexane/Et_2O (5:1, v/v) as the eluent. The colourless fraction with R_f = 0.7−0.8 and blue fluorescence gave the corresponding products.

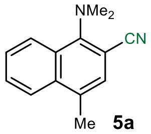

5a

1-(Dimethylamino)-4-methyl-2-naphthonitrile 5a: Colourless oil, yield: 42 mg (80%). ^{1}H NMR (400 MHz, $CDCl_3$): *δ*= 2.61 (s, 3 H), 3.16 (s, 6 H), 7.27 (s, 1 H), 7.58 (ddd, *J* = 8.2, 6.8, 1.4 Hz, 1 H), 7.64 (ddd, *J* = 8.3, 6.8, 1.5 Hz, 1 H), 7.95 (dd, *J* = 8.4, 1.6 Hz, 1 H), 8.32 (dd, *J* = 8.3, 1.5 Hz, 1 H) ppm. ^{13}C{^{1}H} NMR (100 MHz, $CDCl_3$): *δ*= 19.0, 44.6, 103.1, 119.7, 124.8, 125.9, 126.5, 128.1, 128.7, 130.8, 131.1, 135.6, 154.5 ppm. HRMS (ESI): *m/z* calcd. for $C_{14}H_{15}N_2^+$ [M+H$^+$]: 211.1230, found 211.1228.

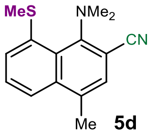

5d

1-(Dimethylamino)-4-methyl-8-(methylthio)-2-naphthonitrile 5d: Pale yellow oil, yield: 58 mg (90%). ^{1}H NMR (400 MHz, $CDCl_3$): *δ*= 2.41 (s, 3 H), 2.62 (s, 3 H), 2.90 (s, 6 H), 7.29 (d, *J* = 7.6 Hz, 1 H), 7.34 (s, 1 H), 7.52–7.57 (m, 1 H), 7.67 (dd, *J* = 8.3, 1.1 Hz, 1 H) ppm. ^{13}C{^{1}H} NMR (100 MHz, $CDCl_3$): *δ*= 17.7, 20.1, 43.0, 107.7, 119.0, 120.0, 122.0, 128.6, 128.7, 130.0, 133.6, 137.2, 140.6, 154.0 ppm. HRMS (ESI): *m/z* calcd. for $C_{15}H_{17}N_2S^+$ [M+H$^+$]: 257.1107, found 257.1110.

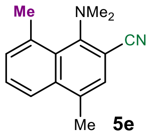

5e

1-(Dimethylamino)-4,8-dimethyl-2-naphthonitrile 5e: Colourless crystals with mp = 93−95 °C (Et_2O), yield: 50 mg (90%). ^{1}H NMR (400 MHz, $CDCl_3$): *δ*= 2.62 (s, 3 H), 2.90 (s, 3 H), 2.98 (s, 6 H), 7.31 (s, 1 H), 7.37 (d, *J* = 7.1 Hz, 1 H), 7.50 (dd, *J* = 8.4, 7.1 Hz, 1 H), 7.83 (d, *J* = 8.4 Hz, 1 H) ppm. ^{13}C{^{1}H} NMR (100 MHz, $CDCl_3$): *δ*= 20.1, 23.9, 43.5, 108.1, 119.6, 123.2, 128.2(6), 128.2(8), 131.1, 131.6, 133.1, 137.0, 137.3, 155.2 ppm. HRMS (ESI): *m/z* calcd. for $C_{15}H_{17}N_2^+$ [M+H$^+$]: 225.1387, found 225.1388.

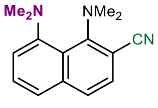

5c

1,8-Bis(dimethylamino)-2-naphthonitrile 5c:
Method A: the mixture of aldoxime **4c** (39 mg, 0.15 mmol) and HCl (37% aqueous solution, 25 μL, 0.3 mmol, 2 equiv.) in 650 μL of DMSO-*d6* was heated at 100 °C for 86 h. Then the reaction mixture was treated with aqueous ammonia (50 mL) and water (200 mL) and extracted with Et_2O (3×10 mL). The extract was dried over Na_2SO_4 and evaporated. The crude product was purified by thin layer chromatography on Al_2O_3 with *n*-hexane/EtOAc (1:10, v/v) as the eluent. The yellowish fraction with R_f = 0.8 gave product **5c** in 8 % yield (3 mg).
Method B: the mixture of aldehyde **26c** (472 mg, 1.95 mmol) and $NH_2OH{\cdot}HCl$ (271 mg, 3.9 mmol, 2 equiv.) in 2 mL of DMSO was heated at 100 °C for 24 h. Then the reaction mixture was treated with aqueous ammonia (75 mL) and water (150 mL) and extracted with Et_2O (3×25 mL). The extract was dried over Na_2SO_4 and evaporated. The crude product was purified by thin layer chromatography on Al_2O_3 with *n*-hexane/EtOAc (1:10, v/v) as the eluent. The yellowish fraction with R_f = 0.8 gave product **5c** in 39 % yield (182 mg).
The spectroscopic ^{1}H NMR data corresponds to that of the previously reported.[2] ^{1}H NMR (400 MHz, $CDCl_3$): *δ*= 2.78 (s, 6H), 3.17 (s, 6H), 6.98 (d, *J* = 7.8 Hz, 1H), 7.25–7.33 (m, 3H), 7.36−7.41 (m, 1H) ppm.

1,5-Dimethyl-1*H*-benzo[*g*]indazoles (*general procedure*): A solution of corresponding naphthaldehyde oxime (0.25 mmol: 57 mg of **4a**, 68 mg of **4d**, 60 mg of **4e**), HI (55% aqueous solution, 0.08 mL, 0.50 mmol, 2 equiv.) in methanol (10 mL) was stirred for 3 h at 65 °C, then treated with aqueous ammonia and extracted with CH_2Cl_2 (3×10 mL). The solvent was evaporated to dryness, and the residue was purified by thin layer chromatography on Al_2O_3 with *n*-hexane/Et_2O (1:1, v/v) as the eluent. The colourless fraction with R_f = 0.7−0.8 with blue fluorescence gave the corresponding indazole.

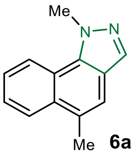

6a

1,5-Dimethyl-1*H*-benzo[*g*]indazole 6a: Colourless crystals with mp = 74−75 °C (Et_2O), yield: 40 mg (81%). ^{1}H NMR (400 MHz, $CDCl_3$): *δ*= 2.68 (s, 3 H), 4.51 (s, 3 H), 7.50 (s, 1 H), 7.58–7.67 (m, 2 H), 7.92 (s, 1 H), 8.02–8.13 (m, 1 H), 8.42–8.50 (m, 1 H) ppm. ^{13}C{^{1}H} NMR (100 MHz, $CDCl_3$): *δ*= 20.2, 40.9, 119.2, 121.3, 121.7, 122.2, 125.6, 125.8, 126.0, 127.8, 132.4, 132.8, 135.3 ppm. HRMS (ESI): *m/z* calcd. for $C_{13}H_{13}N_2^+$ [M+H$^+$]: 197.1074, found 197.1071.

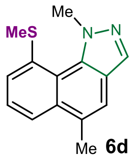

6d

1,5-Dimethyl-9-(methylthio)-1*H*-benzo[*g*]indazole 6d: Colourless oil, yield: 51 mg (85%). ^{1}H NMR (400 MHz, $CDCl_3$): *δ*= 2.40 (s, 3 H), 2.65 (s, 3 H), 4.30 (s, 3 H), 7.49 (s, 1 H), 7.53–7.58 (m, 1 H), 7.68 (dd, *J* = 7.4, 1.2 Hz, 1 H), 7.90 (dd, *J* = 8.1, 1.2 Hz, 1 H), 8.06 (s, 1 H) ppm. ^{13}C{^{1}H} NMR (100 MHz, $CDCl_3$): *δ*= 19.9, 20.5, 43.8, 119.8, 122.3, 122.4, 123.4, 126.2, 128.3, 128.5, 133.6, 134.4, 134.8, 138.2 ppm. HRMS (ESI): *m/z* calcd. for $C_{14}H_{15}N_2S^+$ [M+H$^+$]: 243.0951, found 243.0949.

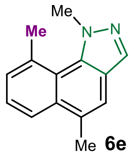
6e

1,5,9-Trimethyl-1*H*-benzo[*g*]indazole 6e: Pale yellow crystals with mp = 83−85 °C (Et_2O), yield: 45 mg (86%). ^{1}H NMR (400 MHz, $CDCl_3$): *δ*= 2.65 (s, 3 H), 2.92 (s, 3 H), 4.17 (s, 3 H), 7.45–7.50 (m, 2 H), 7.52–7.57 (m, 1 H), 7.90 (d, *J* = 8.1 Hz, 1 H), 8.05 (s, 1 H) ppm. ^{13}C{^{1}H} NMR (100 MHz, $CDCl_3$): *δ*= 20.5, 24.2, 42.6, 119.0, 121.9, 122.2, 122.8, 125.9, 128.5, 129.0, 131.9, 134.2, 135.0, 139.0 ppm. HRMS (ESI): *m/z* calcd. for $C_{14}H_{15}N_2^+$ [M+H$^+$]: 211.1230, found 211.1232.

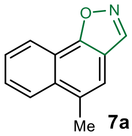
7a

5-Methylnaphtho[2,1-*d*]isoxazole 7a: A solution of **4a** (57 mg, 0.25 mmol) in glacial acetic acid (10 mL) was stirred overnight at 65 °C, then treated with aqueous ammonia and extracted with CH_2Cl_2 (3×10 mL). The solvent was evaporated to dryness, and the residue was purified by thin layer chromatography on Al_2O_3 with *n*-hexane/Et_2O (5:1, v/v) as the eluent. The first colourless fraction with R_f = 0.7−0.8 with blue fluorescence gave **5a** (28 mg, 53%). The second colourless fraction with R_f = 0.5−0.6 with blue fluorescence gave **7a** (3 mg, 7%) as a colourless oil. ^{1}H NMR (400 MHz, $CDCl_3$): *δ*= 2.76 (d, *J* = 1.1 Hz, 3 H), 7.52 (d, *J* = 1.1 Hz, 1 H), 7.71–7.78 (m, 2 H), 8.09–8.14 (m, 1 H), 8.45–8.53 (m, 1 H), 8.75 (s, 1 H) ppm. ^{13}C{^{1}H} NMR (100 MHz, $CDCl_3$): *δ*= 20.0, 116.4, 117.8, 119.4, 122.5, 125.3, 127.0, 128.2, 131.4, 133.5, 146.6, 160.4 ppm. HRMS (ESI): *m/z* calcd. for $C_{12}H_{10}NO^+$ [M+H$^+$]: 184.0757, found 184.0759.

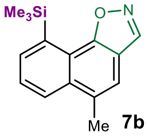
7b

5-Methyl-9-(trimethylsilyl)naphtho[2,1-*d*]isoxazole 7b: A solution of **4b** (30 mg, 0.10 mmol), HBF_4 (50% aqueous solution, 0.05 mL, 0.20 mmol, 2 equiv.) in methanol (10 mL) was stirred for 9 h at 65 °C, then treated with aqueous ammonia and extracted with Et_2O (5×10 mL). The solvent was evaporated to dryness, and the residue was purified by thin layer chromatography on Al_2O_3 with *n*-hexane/Et_2O (2:1, v/v) as the eluent. The first colourless fraction with R_f = 0.8 with blue fluorescence gave **7b** (9 mg, 35%), the second colourless fraction with R_f = 0.3 with blue fluorescence gave **7b** (6 mg, 20%). Compound **7b** was obtained as colourless oil. ^{1}H NMR (400 MHz, $CDCl_3$): *δ*= 0.54 (s, 9 H), 2.75 (d, *J* = 1.0 Hz, 3 H), 7.53 (d, *J* = 1.0 Hz, 1 H), 7.69 (dd, *J* = 8.4, 7.0 Hz, 1 H), 7.95 (dd, *J* = 7.0, 1.2 Hz, 1 H), 8.14 (dd, *J* = 8.4, 1.2 Hz, 1 H), 8.74 (s, 1 H) ppm. ^{13}C{^{1}H} NMR (100 MHz, $CDCl_3$): *δ*= 0.6, 20.6, 116.9, 117.9, 123.4, 126.4, 127.2, 132.1, 134.1, 134.7, 136.6, 146.4, 161.0 ppm. HRMS (ESI): *m/z* calcd. for $C_{15}H_{18}NOSi^+$ [M+H$^+$]: 256.1153, found 256.1149.

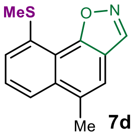
7d

5-Methyl-9-(methylthio)naphtho[2,1-*d*]isoxazole 7d: A solution of **4d** (68 mg, 0.25 mmol), HBF_4 (50% aqueous solution, 0.12 mL, 0.50 mmol, 2 equiv.) in methanol (10 mL) was stirred for 3 h at 65 °C, then treated with aqueous ammonia and extracted with CH_2Cl_2 (3×10 mL). The solvent was evaporated to dryness, and the residue was purified by thin layer chromatography on Al_2O_3 with *n*-hexane/Et_2O (2:1, v/v) as the eluent. The first colourless fraction with R_f = 0.9 with blue fluorescence gave **5d** (8 mg, 13%), the second colourless fraction with R_f = 0.6 with blue fluorescence gave **6d** (5 mg, 8%). The third colourless fraction with R_f = 0.4 with blue fluorescence gave **7d** (23 mg, 40%), Compound **7d** obtained as colourless crystals with mp = 127−129 °C (Et_2O). ^{1}H NMR (400 MHz, $CDCl_3$): *δ*= 2.68 (s, 3 H), 2.72 (s, 3 H), 7.47 (d, *J* = 7.6 Hz, 1 H), 7.53 (s, 1 H), 7.61–7.68 (m, 1 H), 7.86 (dd, *J* = 8.4, 1.0 Hz, 1 H), 8.75 (s, 1 H) ppm. ^{13}C{^{1}H} NMR (100 MHz, $CDCl_3$): *δ*= 16.2, 20.7, 117.4, 117.9, 118.7, 121.4, 122.5, 127.7, 131.8, 134.8, 137.2, 146.0, 160.6 ppm. HRMS (ESI): *m/z* calcd. for $C_{13}H_{12}NOS^+$ [M+H$^+$]: 230.0635, found 230.0633.

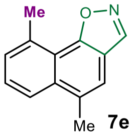
7e

5,9-Dimethylnaphtho[2,1-*d*]isoxazole 7e: A solution of **4e** (24 mg, 0.10 mmol) in glacial acetic acid (10 mL) was stirred for 3 h at 65 °C, then treated with aqueous ammonia and extracted with CH_2Cl_2 (3×10 mL). The solvent was evaporated to dryness, and the residue was purified by thin layer chromatography on Al_2O_3 with *n*-hexane/Et_2O (5:1, v/v) as the eluent. The colourless fraction with R_f = 0.3 with blue fluorescence gave **7e**. Compound **7e** was obtained as colourless crystals with mp = 107−109 °C (Et_2O), yield: 17 mg (88%). ^{1}H NMR (400 MHz, $CDCl_3$): *δ*= 2.73 (s, 3 H), 3.11 (s, 3 H), 7.46–7.53 (m, 2 H), 7.60 (dd, *J* = 8.4, 7.2 Hz, 1 H), 7.94 (d, *J* = 8.4 Hz, 1 H), 8.73 (s, 1 H) ppm. ^{13}C{^{1}H} NMR (100 MHz, $CDCl_3$): *δ*= 20.6, 22.9, 117.0, 117.9, 119.9, 122.8, 127.9, 128.8, 131.8, 134.5, 135.1, 146.1, 161.6 ppm. HRMS (ESI): *m/z* calcd. for $C_{13}H_{12}NO^+$ [M+H$^+$]: 198.0914, found 198.0915.

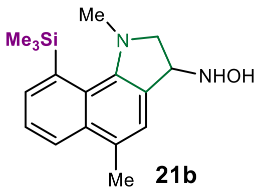
21b

***N*-(1,5-Dimethyl-9-(trimethylsilyl)-2,3-dihydro-1*H*-benzo[*g*]indol-3-yl)hydroxylamine 21b**: A solution of **4b** (30 mg, 0.10 mmol), HBF_4 (50% aqueous solution, 0.05 mL, 0.20 mmol, 2 equiv.) in acetone (10 mL) was stirred for 9 h at 65 °C, then treated with aqueous ammonia and extracted with Et_2O (5×10 mL). The solvent was evaporated to dryness, and the residue was purified by thin layer chromatography on Al_2O_3 with *n*-hexane/Et_2O (2:1, v/v) as the eluent. The colourless fraction with R_f = 0.3 with blue fluorescence gave **21b**. Compound **21b** obtained as colourless crystals with mp = 168−170 °C (Et_2O), yield: 12 mg (40%). ^{1}H NMR (400 MHz, DMSO-d$_6$): *δ*= 0.24 (s, 9 H), 2.54 (s, 3 H), 2.55 (s, 3 H), 3.81 (d, *J* = 10.1 Hz, 1 H), 3.86 (d, *J* = 14.8 Hz, 1 H), 4.15 (d, *J* = 10.1 Hz, 1 H), 4.31 (d, *J* = 14.8 Hz, 1 H), 7.02 (s, 1 H), 7.43 (dd, *J* = 8.3, 6.8 Hz, 1 H), 7.74 (dd, *J* = 6.8, 1.1 Hz, 1 H), 7.89 (dd, *J* = 8.3, 1.1 Hz, 1 H), 8.43 (s, 1 H) ppm. ^{13}C{^{1}H} NMR (100 MHz, DMSO-d$_6$): *δ*= 2.4, 18.8, 47.5, 58.8, 76.4, 124.5(0), 124.5(4), 125.4, 125.8, 130.2, 131.7, 132.5, 135.1, 135.3, 141.5 ppm. HRMS (ESI): *m/z* calcd. for $C_{17}H_{23}N_2OSi^-$ [M−H$^+$]: 299.1585, found 299.1582.

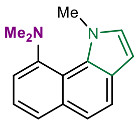
8c

***N*,*N*,1-trimethyl-1*H*-benzo[*g*]indol-9-amine 8c:** Prior to the synthesis of indole **8c**, the salts **4c·HI** and **4f·HI** were obtained by treating the aldoximes **4c** or **4f** with HI (55% aqueous solution, 1 equiv.) in Et_2O. The resulting mixture was stirred for 30 min and the precipitated salts were filtered off, washed with Et_2O, and dried in vacuo. *Method A*: The salt **4c·HI** (62 mg, 0.16 mmol) was dissolved in DMSO-*d6* (650 μL) and heated at 100 ºC for 22 h. Then the resulting mixture was treated with KOH 5% aq. solution (50 mL), then 100 mL of H_2O, and extracted with AcOEt (3×10 mL). The organic extracts were combined and evaporated in vacuo. The crude product was

purified by thin layer chromatography on Al_2O_3 with *n*-hexane as the eluent. The fraction with R_f = 0.8 and violet fluorescence gave indole **8c** in 17% yield (6 mg).

Method B: The salt **4c·HI** (70 mg, 0.18 mmol) was dissolved in *n*-BuOH (5 mL) and heated at 100 °C for 55 h. Then the resulting mixture was treated with KOH 5% aq. solution (50 mL), then 100 mL of H_2O, and extracted with AcOEt (3×10 mL). The organic extracts were combined and evaporated in vacuo. The crude product was purified by thin layer chromatography on Al_2O_3 with *n*-hexane as the eluent. The fraction with R_f = 0.8 and violet fluorescence gave indole **8c** in 25% yield (10 mg).

Method C: The salt **4f·HI** (26 mg, 0.065 mmol) was dissolved in DMSO-*d6* (650 µL) and heated at 100 °C for 19 h. Then the resulting mixture was treated with KOH 5% aq. solution (50 mL), then 100 mL of H_2O, and extracted with AcOEt (3 × 10 mL). The organic extracts were combined and evaporated in vacuo. The crude product was purified by thin layer chromatography on Al_2O_3 with *n*-hexane as the eluent. The fraction with R_f = 0.8 and violet fluorescence yielded indole **8c** in 53 % (8 mg).

The spectroscopic ^{1}H NMR data corresponds to that of the previously reported.[3] ^{1}H NMR (400 MHz, CD_3CN): *δ*= 2.67 (s, 6H), 4.04 (s, 3H), 6.60 (d, *J* = 3.0 Hz, 1H), 7.16 (dd, *J* = 7.6, 0.9 Hz, 1H), 7.21 (d, *J* = 3.0 Hz, 1H), 7.28–7.34 (m, 1H), 7.40 (d, *J* = 8.4 Hz, 1H), 7.52 (dd, *J* = 7.9, 0.9 Hz, 1H), 7.60 (d, *J* = 3.0 Hz, 1H) ppm.

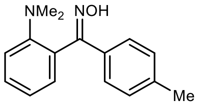

9a

(2-(Dimethylamino)phenyl)(*p*-tolyl)methanone oxime 9a: An unseparated mixture of **25a** and *N*,*N*-dimethylaniline (1.42 mmol of **25a**, 338 mg of mixture), $NH_2OH·HCl$ (198 mg, 2.84 mmol, 2 equiv.) in ethanol (25 mL) was stirred under reflux for 3 h. The solvent was evaporated to dryness, the residue was treated with water and extracted with CH_2Cl_2 (2×20 ml). The combined organic extracts were dried over Na_2SO_4 and filtered off. The solvent was evaporated to dryness. The residue was purified by thin layer chromatography on silica gel with *n*-hexane/AcOEt (4:1, v/v) as the eluent. The yellow fraction with R_f = 0.3 gave the product **9a** as a yellow solid. Yield: 140 mg (overall yield: 35% (two synthetic stages)). ^{1}H NMR (400 MHz, $CDCl_3$): *δ*= 2.38 (s, 3H), 2.79 (s, 6H), 7.05 (dd, *J* = 7.5, 1.1 Hz, 1H), 7.13–7.19 (m, 4H), 7.40–7.50 (m, 3H), 9.35 (s, 1H) ppm. ^{13}C{^{1}H} NMR (100 MHz, $CDCl_3$): *δ*= 117.7, 121.5, 126.1, 127.7, 129.0, 130.2, 131.5, 133.8, 139.3, 151.0, 160.0 ppm. HRMS (ESI): *m/z* calcd. for $C_{16}H_{19}N_2O^+$ [M+H$^+$]: 255.1492, found 255.1489.

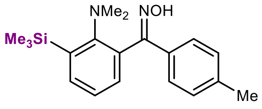

9b

(2-(Dimethylamino)-3-(trimethylsilyl)phenyl)(*p*-tolyl)methanone oxime 9b: A mixture of **25b** (309 mg, 1 mmol) and $NH_2OH·HCl$ (139 mg, 2 mmol, 2 equiv.) in ethanol was stirred under reflux for 3 h. The solvent was evaporated to dryness, the residue was treated with water and extracted with CH_2Cl_2 (2×20 ml). The combined organic extracts were dried over Na_2SO_4 and filtered off. The solvent was evaporated to dryness. Compound **9b** was obtained as an inseparable mixture of two stereoisomers as yellowish solid. Yield: 323 mg (99%). ^{1}H NMR (400 MHz, $CDCl_3$): *δ* = 0.27–0.30 (m, 9H), 2.37–2.39 (m, 3H), 2.45–2.53 (m, 6H), 7.11–7.23 (m, 3H), 7.24–7.34 (m, 1H), 7.37–7.43 (m, 1H), 7.52–7.63 (m, 2H), 8.26–9.09 (m, 1H) ppm. ^{13}C{^{1}H} NMR (100 MHz, $CDCl_3$): *δ* = 0.1, 0.2, 21.3, 21.4, 44.8, 45.0, 124.6, 124.9, 127.0, 128.5, 129.1, 130.6, 130.6, 131.5, 131.5, 133.5, 133.7, 136.2, 136.4, 136.5, 139.7, 139.9, 141.9, 142.0, 156.3, 156.9, 157.2, 159.9 ppm. HRMS (ESI): *m/z* calcd. for $C_{19}H_{27}N_2OSi^+$ [M+H$^+$]: 327.1888, found 327.1881.

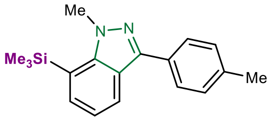

10b

1-Methyl-3-(*p*-tolyl)-7-(trimethylsilyl)-1*H*-indazole 10b: A solution of **10b** (0.09 mmol, 30 mg), HI (55% aqueous solution, 25 µL, 0.18 mmol, 2 equiv.) in methanol was stirred for 48 h at 65 °C. The solvent was evaporated to dryness, the residue was treated with water and extracted with CH_2Cl_2 (3×20 mL). The combined organic extracts were dried over Na_2SO_4 and filtered off. The solvent was evaporated to dryness. The product was purified by thin layer chromatography on Al_2O_3 with *n*-hexane/Et_2O (20:1, v/v) as the eluent. The colorless fraction with weak blue fluorescence with R_f = 0.3 gave compound **10b** as colorless oil. Yield: 6 mg (23%). ^{1}H NMR (400 MHz, $CDCl_3$): *δ* = 0.51 (s, 9H), 2.43 (s, 3H), 4.30 (s, 3H), 7.16 (dd, *J* = 8.1, 6.9 Hz, 1H), 7.31 (d, *J* = 8.1 Hz, 2H), 7.60 (dd, *J* = 6.9, 1.2 Hz, 1H), 7.79 (d, *J* = 8.1 Hz, 2H), 8.01 (dd, *J* = 8.1, 1.2 Hz, 1H) ppm. ^{13}C{^{1}H} NMR (100 MHz, $CDCl_3$): *δ* = 1.7, 21.4, 39.2, 120.5, 121.1, 121.4, 123.1, 127.7, 129.6, 130.2, 134.7, 137.9, 144.4, 145.4 ppm.

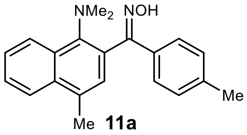

11a

(1-(Dimethylamino)-4-methylnaphthalen-2-yl)(*p*-tolyl)methanone oxime 11a: A solution of **27a** (121 mg, 0.40 mmol), $NH_2OH·HCl$ (56 mg, 0.80 mmol, 2 equiv.), and Et_3N (0.5 mL) in methanol (10 mL) was stirred for 24 h at 65 °C. The solvent was evaporated to dryness, and the residue was purified by thin layer chromatography on Al_2O_3 with *n*-hexane/Et_2O (1:1, v/v) as the eluent. The colourless fraction with R_f = 0.8−0.9 with blue fluorescence gave the product, which was recrystallized from *n*-hexane to give an inseparable mixture of *syn*- and *anti*-forms of **11a** (1:1). Compound **11a** was obtained as colourless solid, yield: 118 mg (93%). ^{1}H NMR (400 MHz, $CDCl_3$): *δ*= 2.37 (s, 3 H), 2.38 (s, 3 H), 2.65 (s, 3 H), 2.67 (s, 3 H), 2.74 (s, 6 H), 2.80 (s, 6 H), 7.07 (s, 1 H), 7.13 (d, *J* = 8.0 Hz, 2 H), 7.18 (d, *J* = 8.0 Hz, 2 H), 7.26 (s, 1 H), 7.42 (d, *J* = 8.2 Hz, 2 H), 7.50–7.61 (m, 6 H), 7.94–8.06 (m, 2 H), 8.14–8.21 (m, 1 H), 8.21–8.27 (m, 1 H), 9.23 (s, 2 H) ppm. ^{13}C{^{1}H} NMR (100 MHz, $CDCl_3$): *δ*= 19.2, 19.3, 21.4, 21.6, 43.8, 44.2, 124.8, 125.4, 125.5, 125.7, 125.8, 126.2, 126.3, 127.3, 127.7, 128.2, 128.5, 129.1, 129.2, 129.9, 130.0, 130.2(7), 130.3(4), 131.0, 131.2, 131.6, 132.3, 134.0, 134.2(7), 134.2(9), 139.5, 139.6, 146.4, 147.3, 157.4, 159.0 ppm. HRMS (ESI): *m/z* calcd. for $C_{21}H_{23}N_2O^+$ [M+H$^+$]: 319.1805, found 319.1805.

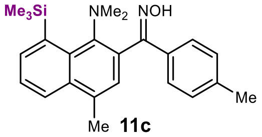

(1-(Dimethylamino)-4-methyl-8-(trimethylsilyl)naphthalen-2-yl)(*p*-tolyl)methanone oxime 11c: A solution of **27c** (67 mg, 0.20 mmol) and $NH_2OH·HCl$ (28 mg, 0.40 mmol, 2 equiv.) in ethanol (10 mL) was stirred for 24 h at 85 °C and treated with aqueous ammonia (10 mL). The products were extracted with CH_2Cl_2 (3×20 mL), the solvent was evaporated to dryness. The residue was purified by thin layer chromatography on Al_2O_3 with *n*-hexane/Et_2O (2:1, v/v) as the eluent. The first colourless fraction with R_f = 0.8 with blue fluorescence gave **13c** (12 mg, 17%), the second colourless fraction with R_f = 0.5 with blue fluorescence gave **11c** (48 mg, 62%). Compound **11c** was obtained as colourless oil. ^{1}H NMR (400 MHz, $CDCl_3$): *δ*= 0.24 (s, 9 H), 2.35 (s, 3 H), 2.43 (s, 3 H), 2.63 (s, 3 H), 2.65 (s, 3 H), 6.88 (s, 1 H), 7.13 (d, *J* = 8.3 Hz, 2 H), 7.45 (d, *J* = 8.3 Hz, 2 H), 7.50 (dd, *J* = 8.3, 6.8 Hz, 1 H), 7.85 (dd, *J* = 6.8, 1.2 Hz, 1 H), 7.93 (dd, *J* = 8.3, 1.2 Hz, 1 H), 8.10 (s, 1 H) ppm. ^{13}C{^{1}H} NMR (100 MHz, $CDCl_3$): *δ*= 2.3, 19.4, 21.4, 45.0, 45.7, 123.4, 125.1, 125.5, 127.3, 127.4, 129.3, 131.0, 133.9, 134.1, 135.9, 136.4, 138.7, 139.9, 147.3, 159.9 ppm. HRMS (ESI): *m/z* calcd. for $C_{24}H_{31}N_2OSi^+$ [M+H$^+$]: 391.2201, found 391.2202.

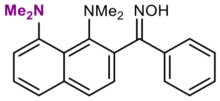

(1,8-Bis(dimethylamino)naphthalen-2-yl)(phenyl)methanone oxime 11d: A mixture of imine **27d** (50 mg, 0.16 mmol) and $NH_2OH·HCl$ (22 mg, 0.32 mmol, 2 equiv.) in 3 mL of ethanol was refluxed for 4.5 h. Then the resulting mixture was evaporated to dryness under the air. The residue was treated with aqueous ammonia (10 mL) and extracted with CH_2Cl_2 (3×10 mL). The extract was dried over Na_2SO_4 and evaporated. The product was used in further transformations without further purification due to its instability on sorbents. The spectroscopic ^{1}H NMR data corresponds to that of the previously reported.[2] ^{1}H NMR (400 MHz, $CDCl_3$): *δ* = 2.53–3.03 (m, 12H), 7.04 (d, *J* = 7.33 Hz, 1H), 7.26–7.48 (m, 8H), 7.54 (d, *J* = 8.30 Hz, 1H) ppm.

(1,8-Bis(dimethylamino)naphthalen-2-yl)(aryl)methanone *O*-methyl oxime *(general procedure)*: A mixture of corresponding imine ((1 eq.: 234 mg, 0.74 mmol of **27d** for the synthesis of **11g** or 271 mg, 0.82 mmol of **27h** for the synthesis of **11h**) and $NH_2OMe·HCl$ (2 equiv.) in ethanol (7 mL) was refluxed overnight and then the resulting mixture was evaporated to dryness under the air. The residue was treated with aqueous ammonia (20 mL), extracted with CH_2Cl_2 (3×15 mL). The extract was dried over Na_2SO_4 and evaporated. The compounds **11g** and **11h** were purified by thin layer chromatography on Al_2O_3 with *n*-hexane/EtOAc (50:1, v/v) as the eluent. The yellowish fractions with R_f = 0.8–0.9 resulted in the products almost in pure *E*-form with insignificant amount of *Z*-form.

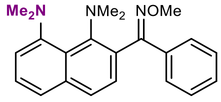

(1,8-Bis(dimethylamino)naphthalen-2-yl)(phenyl)methanone *O*-methyl oxime 11g: **11g** was obtained as yellow oil in 62% yield (158 mg). ^{1}H NMR (400 MHz, $CDCl_3$): *δ*= 2.52–3.02 (m, 12H), 4.10 (s, 3H), 7.05 (dd, *J* = 7.5, 1.0 Hz, 1H), 7.22 (d, *J* = 8.3 Hz, 1H), 7.29–7.48 (m, 7H), 7.53 (d, *J* = 8.3 Hz, 1H) ppm. ^{13}C{^{1}H} NMR (100 MHz, $CDCl_3$): δ= 43.7, 62.3, 65.9, 113.9, 122.2, 122.6, 123.1, 125.7, 126.2, 127.1, 127.9, 128.1, 129.2, 137.4, 138.4, 147.9, 151.9, 158.9 ppm. HRMS (ESI): *m/z* calcd. for $C_{22}H_{26}N_3O^+$ [M+H$^+$]: 348.2071, found 348.2075.

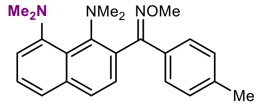

(1,8-Bis(dimethylamino)naphthalen-2-yl)(*p*-tolyl)methanone *O*-methyl oxime 11h: **11h** was obtained as yellow oil in 63% yield (186 mg). ^{1}H NMR (400 MHz, $CDCl_3$): *δ*= 2.41 (s, 3H), 2.67–3.03 (m, 12H), 4.11 (s, 3H), 7.08 (dd, *J* = 7.4, 1.0 Hz, 1H), 7.13–7.19 (m, 2H), 7.23 (d, *J* = 8.3 Hz, 1H), 7.36–7.44 (m, 3H), 7.49 (dd, *J* = 8.2, 1.0 Hz, 1H), 7.55 (d, *J* = 8.3 Hz, 1H) ppm. ^{13}C{^{1}H} NMR (100 MHz, $CDCl_3$): δ= 15.4, 21.4, 43.8, 62.3, 113.7, 122.2, 122.7, 123.0, 126.2, 127.1, 128.0, 128.9, 129.6, 134.5, 138.4, 139.3, 147.8, 151.9, 158.9 ppm. HRMS (ESI): *m/z* calcd. for $C_{23}H_{28}N_3O^+$ [M+H$^+$]: 362.2227, found 362.2233.

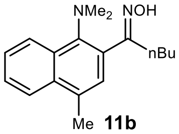

1-(1-(Dimethylamino)-4-methylnaphthalen-2-yl)pentan-1-one oxime 11b: *n*-Butyllithium (1.6 M solution in hexanes, 0.07 mL, 0.11 mmol, 1.1 equiv.) was added via syringe to **5a** (26 mg, 0.10 mmol) in dry Et_2O (10 mL) in a flame-dried flask under argon atmosphere at room temperature. Resulting mixture was stirred for 12 h at the room temperature and treated with water (10 mL). The products were extracted with CH_2Cl_2 (3×20 mL), the solvent was evaporated to dryness. The residue was dissolved in methanol (15 mL), $NH_2OH·HCl$ (14 mg, 0.20 mmol, 2 equiv.) and Et_3N (0.5 mL) were added, the resulting mixture was stirred for 24 h at 65 °C and treated with water (10 mL). The products were extracted with CH_2Cl_2 (3×20 mL), the solvent was evaporated to dryness. The residue was purified by thin layer chromatography on Al_2O_3 with *n*-hexane/Et_2O (1:1, v/v) as the eluent. The colourless fraction with R_f = 0.5 with blue fluorescence gave **11b**. Compound **11b** was obtained as pale yellow oil, yield: 21 mg (73%). ^{1}H NMR (400 MHz, $CDCl_3$): *δ*= 0.87 (t, *J* = 7.3 Hz, 3 H), 1.30–1.39 (m, 2 H), 1.43–1.51 (m, 2 H), 2.64 (d, *J* = 1.0 Hz, 3 H), 2.72–2.78 (m, 2 H), 3.00 (s, 6 H), 7.11 (d, *J* = 1.0 Hz, 1 H), 7.49–7.55 (m, 2 H), 7.93–8.01 (m, 1 H), 8.16–8.21 (m, 1 H), 8.42 (s, 1 H) ppm. ^{13}C{^{1}H} NMR (100 MHz, $CDCl_3$): *δ*= 13.9, 19.2, 23.3, 28.0, 29.6, 44.7, 124.9, 125.4, 125.6, 126.0, 128.2, 130.5, 130.7, 132.1, 134.1, 146.1, 162.9 ppm. HRMS (ESI): *m/z* calcd. for $C_{18}H_{25}N_2O^+$ [M+H$^+$]: 285.1962, found 285.1965.

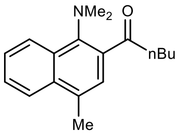

When trying to isolate the corresponding ketimine, its hydrolysis on Al_2O_3 occurs with the formation of the corresponding ketone 1-(1-(dimethylamino)-4-methylnaphthalen-2-yl)pentan-1-one as yellow oil, yield 22 mg (80%). ^{1}H NMR (400 MHz, $CDCl_3$): *δ*= 0.96 (t, *J* = 7.4 Hz, 3 H), 1.38–1.48 (m, 2 H), 1.74 (p, *J* = 7.5 Hz, 2 H), 2.64 (s, 3 H), 2.92 (t, *J* = 7.5 Hz, 2 H), 2.98 (s, 6 H), 7.19 (s, 1 H), 7.51–7.58 (m, 2 H), 7.94–8.01 (m, 1 H), 8.21–8.26 (m, 1 H) ppm. ^{13}C{^{1}H} NMR (100 MHz, $CDCl_3$): *δ*= 14.1, 19.4, 22.7, 26.8, 42.9, 44.9, 124.9(7), 125.0(1), 125.8, 125.9, 126.8, 131.0, 132.1, 134.9, 135.3, 146.6, 207.9 ppm. HRMS (ESI): *m/z* calcd. for $C_{18}H_{24}NO^+$ [M+H$^+$]: 270.1853, found 270.1855.

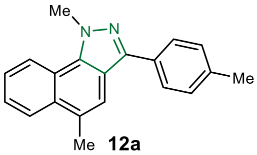
12a

1,5-Dimethyl-3-(*p*-tolyl)-1*H*-benzo[*g*]indazole 12a and 5-Methyl-3-(*p*-tolyl)naphtho[2,1-*d*]isoxazole 13a: A solution of naphthaldehyde oxime **11a** (48 mg, 0.15 mmol), HI (55% aqueous solution, 0.05 mL, 0.30 mmol, 2 equiv.) in methanol (10 mL) was stirred for 72 h at 65 °C, then treated with aqueous ammonia and extracted with CH_2Cl_2 3×10 mL. The solvent was evaporated to dryness, and the residue was purified by thin layer chromatography on Al_2O_3 (1×10 cm) with *n*-hexane/Et_2O (2:1, v/v) as the eluent. The first colourless fraction with R_f = 0.8 with blue fluorescence gave **13a** (21 mg, 37%), the second colourless fraction with R_f = 0.6 with blue fluorescence gave **12a** (8 mg, 19%). Compound **12a** was obtained as colourless crystals with mp = 141−143 °C (Et_2O). ^{1}H NMR (400 MHz, $CDCl_3$): δ= 2.45 (s, 3 H), 2.72 (s, 3 H), 4.58 (s, 3 H), 7.34 (d, *J* = 7.9 Hz, 2 H), 7.61–7.69 (m, 2 H), 7.79 (s, 1 H), 7.84 (d, *J* = 7.9 Hz, 2 H), 8.07–8.16 (m, 1 H), 8.49–8.56 (m, 1 H) ppm. ^{13}C{^{1}H} NMR (100 MHz, $CDCl_3$): δ= 20.5, 21.5, 41.1, 118.7, 119.5, 121.9, 122.4, 125.6, 126.0, 126.1, 127.8, 128.1, 129.6, 131.0, 132.7, 136.8, 137.6, 143.7 ppm. HRMS (ESI): *m/z* calcd. for $C_{20}H_{19}N_2^+$ [M+H$^+$]: 287.1543, found 287.1546.

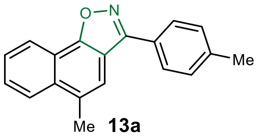
13a

Compound **13a** was obtained as colourless crystals with mp = 148−150 °C (Et_2O). ^{1}H NMR (400 MHz, $CDCl_3$): δ= 2.48 (s, 3 H), 2.76 (d, *J* = 1.1 Hz, 3 H), 7.40 (d, *J* = 8.2 Hz, 2 H), 7.68 (d, *J* = 1.1 Hz, 1 H), 7.69–7.76 (m, 2 H), 7.91 (d, *J* = 8.2 Hz, 2 H), 8.08–8.14 (m, 1 H), 8.46–8.53 (m, 1 H) ppm. ^{13}C{^{1}H} NMR (100 MHz, $CDCl_3$): δ= 20.1, 21.6, 115.5, 118.2, 119.7, 122.5, 125.2, 126.6, 127.0, 128.2(1), 128.2(3), 130.0, 131.4, 133.2, 140.4, 157.8, 161.7 ppm. HRMS (ESI): *m/z* calcd. for $C_{19}H_{16}NO^+$ [M+H$^+$]: 274.1227, found 274.1242.

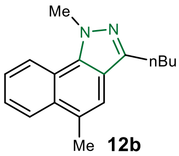
12b

3-Butyl-1,5-dimethyl-1*H*-benzo[*g*]indazole 12b: A solution of naphthaldehyde oxime **11b** (28 mg, 0.10 mmol), HI (55% aqueous solution, 0.03 mL, 0.20 mmol, 2 equiv.) in methanol (10 mL) was stirred for 48 h at 65 °C, then treated with aqueous ammonia and extracted with CH_2Cl_2 3×10 mL. The solvent was evaporated to dryness, and the residue was dissolved in Et_2O and filtered off. Compound **12b** obtained as yellow oil, yield: 20 mg (80%). ^{1}H NMR (400 MHz, $CDCl_3$): δ= 0.98 (t, *J* = 7.4 Hz, 3 H), 1.41–1.52 (m, 2 H), 1.76–1.85 (m, 2 H), 2.70 (s, 3 H), 2.93–3.00 (m, 2 H), 4.47 (s, 3 H), 7.48 (s, 1 H), 7.59–7.65 (m, 2 H), 8.06–8.11 (m, 1 H), 8.44–8.50 (m, 1 H) ppm. ^{13}C{^{1}H} NMR (100 MHz, $CDCl_3$): δ= 14.1, 20.4, 22.9, 26.7, 32.2, 40.5, 118.8, 119.6, 122.0, 122.4, 125.6, 125.8, 125.9, 126.8, 132.8, 136.1, 145.2 ppm. HRMS (ESI): *m/z* calcd. For $C_{17}H_{21}N_2^+$ [M+H$^+$]: 253.1700, found 253.1695.

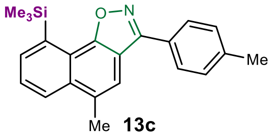
13c

5-Methyl-3-(*p*-tolyl)-9-(trimethylsilyl)naphtho[2,1-*d*]isoxazole 13c: A solution of naphthaldehyde oxime **11c** (39 mg, 0.10 mmol), HI (55% aqueous solution, 0.03 mL, 0.20 mmol, 2 equiv.) in methanol (10 mL) was stirred for 24 h at 65 °C, then treated with aqueous ammonia and extracted with CH_2Cl_2 3×10 mL. The solvent was evaporated to dryness, the residue was purified by thin layer chromatography on Al_2O_3 with *n*-hexane/Et_2O (2:1, v/v) as the eluent. The colourless fraction with R_f = 0.8 with blue fluorescence gave **13c**. Compound **13c** was obtained as colourless crystals with mp = 185−187 °C (Et_2O), yield: 28 mg (80%). ^{1}H NMR (400 MHz, $CDCl_3$): δ= 0.58 (s, 9 H), 2.48 (s, 3 H), 2.78 (s, 3 H), 7.40 (d, *J* = 8.0 Hz, 2 H), 7.69 (dd, *J* = 8.4, 7.0 Hz, 1 H), 7.72 (s, 1 H), 7.92 (d, *J* = 8.0 Hz, 2 H), 7.97 (dd, *J* = 7.0, 1.2 Hz, 1 H), 8.15 (dd, *J* = 8.4, 1.2 Hz, 1 H) ppm. ^{13}C{^{1}H} NMR (100 MHz, $CDCl_3$): δ= 0.7, 20.7, 21.6, 115.9, 118.2, 123.6, 126.2, 126.6, 127.2, 128.2, 130.0, 132.1, 133.7, 134.7, 136.6, 140.3, 157.6, 162.4 ppm. HRMS (ESI): *m/z* calcd. for $C_{22}H_{24}NOSi^+$ [M+H$^+$]: 346.1622, found 346.1626.

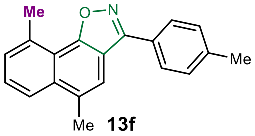
13f

5,9-Dimethyl-3-(*p*-tolyl)naphtho[2,1-*d*]isoxazole 13f: A solution of **27f** (33 mg, 0.10 mmol) and $NH_2OH{\cdot}HCl$ (14 mg, 0.20 mmol, 2 equiv.) in ethanol (10 mL) was stirred for 24 h at 85 °C and treated with aqueous ammonia (10 mL). The products were extracted with CH_2Cl_2 (3×10 mL), the solvent was evaporated to dryness. The residue was purified by thin layer chromatography on Al_2O_3 with *n*-hexane/Et_2O (2:1, v/v) as the eluent. The colourless fraction with R_f = 0.7−0.8 with blue fluorescence gave **13f**. Compound **13f** was obtained as beige crystals with mp = 155−157 °C (Et_2O), yield: 26 mg (90%). ^{1}H NMR (400 MHz, $CDCl_3$): δ= 2.48 (s, 3 H), 2.74 (s, 3 H), 3.13 (s, 3 H), 7.40 (d, *J* = 7.9 Hz, 2 H), 7.50 (d, *J* = 7.1 Hz, 1 H), 7.60 (dd, *J* = 8.4, 7.1 Hz, 1 H), 7.67 (s, 1 H), 7.90 (d, *J* = 7.9 Hz, 2 H), 7.94 (d, *J* = 8.4 Hz, 1 H) ppm. ^{13}C{^{1}H} NMR (100 MHz, $CDCl_3$): δ= 20.7, 21.6, 22.9, 116.1, 118.2, 120.0, 122.7, 126.6, 127.8, 128.3, 128.8, 130.0, 131.8, 134.2, 135.2, 140.2, 157.3, 163.0 ppm. HRMS (ESI): *m/z* calcd. for $C_{20}H_{18}NO^+$ [M+H$^+$]: 288.1383, found 288.1382.

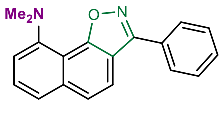
13d

***N*,*N*-dimethyl-3-phenylnaphtho[2,1-*d*]isoxazol-9-amine 13d**: *Method A*: A mixture of imine **11d** (183 mg, 0.58 mmol, 1 eq.) and $NH_2OH{\cdot}HCl$ (60 mg, 0.87 mmol, 1.5 equiv.) in ethanol (7 mL) was refluxed for 20 h and then the resulting mixture was evaporated to dryness under the air. The residue was treated with aqueous ammonia (20 mL), extracted with CH_2Cl_2 (3×10 mL). The extract was dried over Na_2SO_4 and evaporated. The isoxazole **11d** was formed along with oxime **11d** in 1:0.77 ratio, respectively. **11d** was purified by thin layer chromatography on Al_2O_3 with CH_2Cl_2/hexane (2:1, v/v) as the eluent. The yellowish fraction with R_f = 0.9 gave isoxazole **13d** in 39% yield (65 mg).

Method B: HCl (37 % aqueous solution, 10 μL, 2 equiv.) was added to a solution of **11d** (20 mg, 0.06 mmol, 1 equiv.) in DMSO-*d6* (650 μL). The mixture was heated for 41 h at 100 °C. The reaction mixture was treated with aqueous ammonia (10 mL) and water (100 mL) and extracted with CH_2Cl_2 (3×10 mL). The extract was dried over Na_2SO_4 and evaporated. **13d** was purified by thin layer chromatography on Al_2O_3 with *n*-hexane/EtOAc (1:10, v/v) as the eluent. The yellowish fraction with R_f = 0.8 gave isoxazole **13d** in 35% yield (6 mg).

The spectroscopic ^{1}H NMR data corresponds to that of the previously reported.[2] ^{1}H NMR (400 MHz, $CDCl_3$): δ= 3.03 (s, 6H), 7.26 (dd, *J* = 7.3, 1.2 Hz, 1H), 7.54–7.62 (m, 5H), 7.72 (d, *J* = 8.7 Hz, 1H), 7.83 (d, *J* = 8.7 Hz, 1H), 7.99–8.03 (m, 2H) ppm.

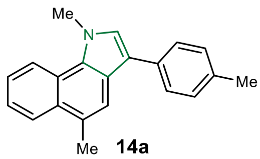

14a

1,5-Dimethyl-3-(*p*-tolyl)-1*H*-benzo[*g*]indole 14a: A solution of **11a** (45 mg, 0.15 mmol) and $NH_2OMe{\cdot}HCl$ (25 mg, 0.30 mmol, 2 equiv.) in ethanol (10 mL) was stirred for 24 h at 85 °C and treated with aqueous ammonia (10 mL). The products were extracted with Et_2O (5×10 mL), the solvent was evaporated to dryness. The residue was purified by thin layer chromatography on Al_2O_3 with *n*-hexane/Et_2O (2:1, v/v) as the eluent. The colourless fraction with R_f = 0.7 with blue fluorescence was collected. The fraction was evaporated to dryness, then treated with *n*-hexane (1–2 mL) and filtered off, the product was recrystallized from Et_2O. Compound **14a** obtained as colourless crystals with mp = 176−178 °C (Et_2O), yield: 16 mg (38%). ^1H NMR (400 MHz, $CDCl_3$): *δ*= 2.43 (s, 3 H), 2.73 (d, *J* = 1.1 Hz, 3 H), 4.33 (s, 3 H), 7.14 (s, 1 H), 7.29 (d, *J* = 7.8 Hz, 2 H), 7.48–7.53 (m, 1 H), 7.53–7.62 (m, 3 H), 7.82 (d, *J* = 1.1 Hz, 1 H), 8.10 (dd, *J* = 8.2, 1.5 Hz, 1 H), 8.53 (dd, *J* = 8.3, 1.5 Hz, 1 H) ppm. $^{13}C\{^1H\}$ NMR (100 MHz, $CDCl_3$): *δ*= 20.5, 21.3, 38.8, 117.5, 120.2, 121.2, 123.2, 123.5, 123.9, 125.1, 125.6, 126.5, 126.8, 128.0, 129.6, 130.0, 130.8, 132.8, 135.6 ppm. HRMS (ESI): *m/z* calcd. for $C_{21}H_{20}N^+$ [M+H$^+$]: 286.1591, found 286.1586.

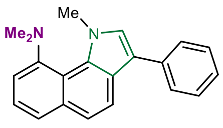

14g

***N,N*,1-trimethyl-3-phenyl-1*H*-benzo[*g*]indol-9-amine 14g**: Prior to the synthesis of indole **14g**, salt **11g·HI** was obtained similarly to **4c·HI**. Salt **11g·HI** (20 mg, 0.042 mmol) was dissolved in DMSO-*d6* (650 µL) and heated at 100 °C for 21 h. Then the resulting mixture was treated with KOH 5% aq. solution (50 mL), then 100 mL of H_2O, and extracted with Et_2O (3×10 mL). The organic extracts were combined and evaporated in vacuo. The crude product was purified by thin layer chromatography on Al_2O_3 with *n*-hexane as the eluent. The fraction with R_f = 0.8 and violet fluorescence gave indole **14g** in 77% yield (10 mg). ^1H NMR (400 MHz, DMSO-*d6*): *δ*= 2.68 (s, 6H), 4.05 (s, 3H), 7.15 (dd, *J* = 8.1, 0.9 Hz, 1H), 7.26–7.35 (m, 2H), 7.44–7.51 (m, 3H), 7.54 (dd, *J* = 8.1, 0.9 Hz, 1H), 7.64–7.69 (m, 3H), 7.85 (d, *J* = 8.6 Hz, 1H) ppm. $^{13}C\{^1H\}$ NMR (100 MHz, DMSO-d6): δ = 42.75, 113.97, 117.07, 117.62, 119.14, 121.59, 122.27, 123.58, 124.07, 125.73, 127.29, 128.83, 129.36, 132.46, 133.34, 135.04, 148.30 ppm. HRMS (ESI): *m/z* calcd. for $C_{21}H_{21}N_2^+$ [M+H$^+$]: 301.1700, found 301.1699.

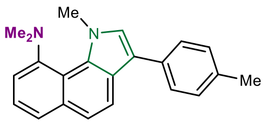

14h

***N,N*,1-trimethyl-3-(*p*-tolyl)-1*H*-benzo[*g*]indol-9-amine 14h**: Prior to the synthesis of indole **14h**, salt **11h·HI** was obtained similarly to **4c·HI**. Salt **11h·HI** (22 mg, 0.044 mmol) was dissolved in DMSO-*d6* (650 µL) and heated at 100 °C for 21 h. Then the resulting mixture was treated with KOH 5% aq. solution (50 mL), then 100 mL of H_2O, and extracted with Et_2O (3 × 10 mL). The organic extracts were combined and evaporated in vacuo. The crude product was purified by thin layer chromatography on Al_2O_3 with *n*-hexane as the eluent. The fraction with R_f = 0.8 and violet fluorescence gave indole **14h** in 57% yield (8 mg). ^1H NMR (400 MHz, CD_3CN): *δ*= 2.39 (s, 3H), 2.70 (s, 6H), 4.07 (s, 3H), 7.18 (dd, *J* = 7.7, 0.8 Hz, 1H), 7.27–7.31 (m, 2H), 7.31–7.37 (m, 1H), 7.40 (s, 1H), 7.47 (d, *J* = 8.6 Hz, 1H), 7.52–7.59 (m, 3H), 7.86 (d, *J* = 8.6 Hz, 1H) ppm. $^{13}C\{^1H\}$ NMR (100 MHz, CD_3CN): δ= 21.18, 40.37, 43.42, 115.08, 118.91, 119.14, 120.45, 122.56, 123.54, 125.21, 128.62, 129.82, 130.49, 133.46, 133.91, 134.92, 136.63, 149.82 ppm. HRMS (ESI): *m/z* calcd. for $C_{22}H_{23}N_2^+$ [M+H$^+$]: 315.1856, found 315.1871.

Synthesis of the starting materials:

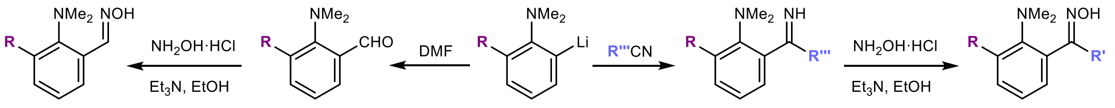

Scheme S1. General scheme for the synthesis of compounds **1** and **9** (note, that compounds **22** and **24** are known and can be prepared according to a literature procedure[1])

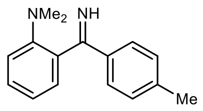

25a

2-(Imino(*p*-tolyl)methyl)-*N,N*-dimethylaniline 25a: *n*-Butyllithium (1.6 M solution in hexanes, 1 mL, 1.6 mmol, 1 equiv.) was added via syringe to a solution of *N*,*N*-dimethylaniline (0.2 mL, 1.58 mmol), TMEDA (0.036 mL, 0.237 mmol) freshly distilled over KOH in dry *n*-hexane (15 mL) in a flame-dried flask under argon atmosphere at 72 °C. The solution was stirred at 72 °C for 24 h and cooled to −24 °C. A solution of *p*-tolunitrile (187 mg, 1.6 mmol, 1 equiv.) in dry *n*-hexane (5 mL) was added via syringe. Resulting mixture was stirred for 12 h at −24°C and treated with water (10 mL). The products were extracted with *n*-hexane (3×20 mL). The combined organic extracts were dried over Na_2SO_4 and filtered off. The solvent was evaporated to dryness giving the mixture of compound **25a** and *N*,*N*-dimethylaniline. The mixture was further used in the synthesis of the corresponding oxime to prevent hydrolysis of imine **25a** on sorbents.

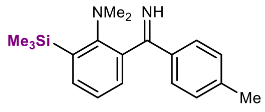

25b

2-(Imino(*p*-tolyl)methyl)-N,N-dimethyl-6-(trimethylsilyl)aniline 25b: *t*-Butyllithium (1.7 M solution in pentane, 2.3 mL, 2 mmol, 2 equiv.) was added via syringe to a solution of 2-bromo-*N*,*N*-dimethyl-6-(trimethylsilyl)aniline (544 mg, 2 mmol) in dry *n*-hexane (30 mL) in a flame-dried flask under argon atmosphere at 5 °C. The resulting mixture was stirred for 24 h at the same temperature and then a solution of *p*-tolunitrile (516 mg, 2.2 mmol, 2.2 equiv.) in dry Et_2O (10 mL) was added via syringe. The resulting mixture was stirred for 24 h at 5 °C and treated with water (10 mL). The products were extracted with CH_2Cl_2 (3×20 mL). The combined organic extracts were dried over Na_2SO_4 and filtered off. The solvent was evaporated to dryness. The product was purified by column chromatography on silica gel with CH_2Cl_2/AcOEt (4:1, v/v) as the eluent. The yellow fraction with R_f = 0.4 gave

the product **25b** as yellow solid. Yield: 334 mg (54%). ^{1}H NMR (400 MHz, $CDCl_3$): δ= 0.31 (s, 9H), 2.41 (s, 3H), 2.57 (s, 6H), 7.10 (dd, J = 7.4, 1.7 Hz, 1H), 7.18–7.24 (m, 3H), 7.57 (dd, J = 7.4, 1.8 Hz, 1H), 7.62–7.66 (m, 2H), 8.00–11.70 (br s, 1H) ppm. ^{13}C{^{1}H} NMR (100 MHz, $CDCl_3$): δ = 0.1, 21.4, 45.6, 124.5, 128.5, 129.0, 130.7, 136.1, 136.2, 139.7, 141.3, 142.3, 155.5, 178.3 ppm. HRMS (ESI): *m/z* calcd. for $C_{19}H_{27}N_2Si^+$ [M+H$^+$]: 311.1939, found 311.1935.

Caution! *Tert-butyllithium is extremely pyrophoric. It must be handled using proper needle and syringe techniques. All manipulations were performed on the smallest practical scale following the procedures described in ref.*[4]

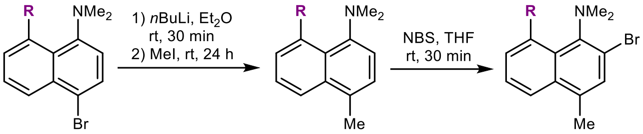

Scheme S2. General scheme for the synthesis of 2-bromo-4-methyl-1-(dimethylamino)naphthalenes

1-(Dimethylamino)-4-methylnaphthalenes (*general procedure*): *n*-Butyllithium (1.6 M solution in hexanes, 1.25 mL, 2 mmol, 1 equiv.) was added via syringe to a solution of corresponding 4-bromo-1-dimethylaminonaphthalene (2 mmol: 502 mg of 8-H, 644 mg of 8-$SiMe_3$, 592 mg of 8-SMe, 528 mg of 8-Me) in dry Et_2O (25 mL) in a flame-dried flask under argon atmosphere at room temperature. Resulting mixture was stirred for 30 min at the same temperature and then MeI (0.15 mL, 2.4 mmol, 1.2 equiv.) was added via syringe. Resulting mixture was stirred for 24 h at the room temperature and then treated with water (10 mL). The products were extracted with CH_2Cl_2 (3×20 mL), the solvent was evaporated to dryness. The residue was purified by column chromatography on Al_2O_3 (2×20 cm) with *n*-hexane as the eluent.

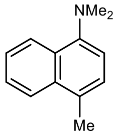

***N,N*,4-Trimethylnaphthalen-1-amine**: Colourless oil, yield: 352 mg (95%). The spectroscopic ^{1}H NMR data corresponds to that of the previously reported.[5] ^{1}H NMR (400 MHz, $CDCl_3$): δ= 2.92 (d, J = 1.1 Hz, 3 H), 3.15 (s, 6 H), 7.26 (d, J = 7.6 Hz, 1 H), 7.50 (dd, J = 7.7, 1.1 Hz, 1 H), 7.78–7.83 (m, 2 H), 8.20–8.29 (m, 1 H), 8.63–8.69 (m, 1 H) ppm.

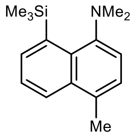

***N,N*,4-Trimethyl-8-(trimethylsilyl)naphthalen-1-amine**: Colourless oil, yield: 452 mg (88%). ^{1}H NMR (400 MHz, $CDCl_3$): δ= 0.32 (s, 9 H), 2.59 (s, 6 H), 2.67 (d, J = 0.9 Hz, 3 H), 7.19 (d, J = 7.5 Hz, 1 H), 7.26–7.30 (m, 1 H), 7.49 (dd, J = 8.4, 6.8 Hz, 1 H), 7.86 (dd, J = 6.8, 1.2 Hz, 1 H), 7.96 (dd, J = 8.3, 1.3 Hz, 1 H) ppm. ^{13}C{^{1}H} NMR (100 MHz, $CDCl_3$): δ= 2.4, 19.6, 47.4, 115.5, 125.0, 125.4, 126.4, 130.8, 133.7, 134.6, 135.4, 137.2, 151.8 ppm. HRMS (ESI): *m/z* calcd. for $C_{16}H_{24}NSi^+$ [M+H$^+$]: 258.1673, found 258.1671.

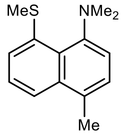

***N,N*,4-Trimethyl-8-(methylthio)naphthalen-1-amine**: Colourless oil, yield: 439 mg (95%). ^{1}H NMR (400 MHz, $CDCl_3$): δ= 2.43 (s, 3 H), 2.65 (s, 3 H), 2.70 (s, 6 H), 7.20–7.25 (m, 2 H), 7.29 (d, J = 7.6 Hz, 1 H), 7.42–7.47 (m, 1 H), 7.67–7.75 (m, 1 H) ppm. ^{13}C{^{1}H} NMR (100 MHz, $CDCl_3$): δ= 17.5, 20.1, 45.6, 118.1, 120.2, 120.5, 125.6, 127.0, 128.6, 131.0, 134.7, 138.4, 150.2 ppm. HRMS (ESI): *m/z* calcd. for $C_{14}H_{18}NS^+$ [M+H$^+$]: 232.1155, found 232.1151.

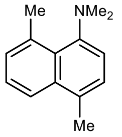

***N,N*,4,8-Tetramethylnaphthalen-1-amine**: Colourless oil, yield: 378 mg (95%). ^{1}H NMR (400 MHz, $CDCl_3$): δ= 2.65 (d, J = 0.9 Hz, 3 H), 2.72 (s, 6 H), 3.00 (s, 3 H), 7.13 (d, J = 7.5 Hz, 1 H), 7.24–7.30 (m, 2 H), 7.40 (dd, J = 8.4, 7.0 Hz, 1 H), 7.85 (d, J = 8.4 Hz, 1 H) ppm. ^{13}C{^{1}H} NMR (100 MHz, $CDCl_3$): δ= 20.1, 23.8, 45.8, 116.1, 122.9, 125.2, 126.3, 129.2, 129.4, 129.9, 135.2, 135.8, 151.2 ppm. HRMS (ESI): *m/z* calcd. for $C_{14}H_{18}N^+$ [M+H$^+$]: 200.1434, found 200.1435.

2-Bromo-4-methyl-1-(dimethylamino)naphthalenes (*general procedure*): A solution of NBS (356 mg, 2 mmol, 1 equiv.) in THF (15 mL) was added dropwise to a stirred solution of NMe_2-substituted arene (2 mmol: 370 mg of 8-H, 514 mg of 8-$SiMe_3$, 462 mg of 8-SMe, 398 mg of 8-Me) in THF (5 mL) at room temperature. The reaction mixture was stirred for 30 min and the solvent was evaporated to dryness. The residue was washed with *n*-hexane (30 mL) on the filter. The filtrate containing the product was evaporated to dryness and purified by column chromatography on Al_2O_3 (2×20 cm) with *n*-hexane as the eluent. The colourless fraction with R_f = 0.9 gave the corresponding product.

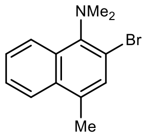

2-Bromo-*N,N*,4-trimethylnaphthalen-1-amine: Colourless oil, yield: 465 mg (88%). ^{1}H NMR (400 MHz, $CDCl_3$): δ= 2.65 (d, J = 1.0 Hz, 3 H), 3.07 (s, 6 H), 7.48 (d, J = 1.0 Hz, 1 H), 7.53–7.62 (m, 2 H), 7.89–8.01 (m, 1 H), 8.34–8.46 (m, 1 H) ppm. ^{13}C{^{1}H} NMR (100 MHz, $CDCl_3$): δ= 18.9, 42.8, 119.8, 124.6, 125.3, 126.1, 126.3, 131.6, 132.9, 133.5, 134.5, 145.4 ppm. HRMS (ESI): *m/z* calcd. for $C_{13}H_{15}{}^{79}BrN^+$ [M+H$^+$]: 264.0383, found 264.0385, *m/z* calcd. for $C_{13}H_{15}{}^{81}BrN^+$ [M+H$^+$]: 266.0362, found 266.0364.

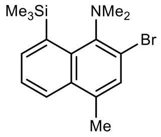

2-Bromo-*N,N*,4-trimethyl-8-(trimethylsilyl)naphthalen-1-amine: Colourless oil, yield: 403 mg (60%). ^{1}H NMR (400 MHz, $CDCl_3$): δ= 0.30 (s, 9 H), 2.61 (d, J = 1.0 Hz, 3 H), 2.83 (s, 6 H), 7.44 (d, J = 1.0 Hz, 1 H), 7.47 (dd, J = 8.3, 6.8 Hz, 1 H), 7.88–7.93 (m, 2 H) ppm. ^{13}C{^{1}H} NMR (100 MHz, $CDCl_3$): δ= 2.6, 19.3, 46.0, 118.0, 125.2, 125.6, 132.8, 133.3, 133.8, 136.7, 138.0, 138.9, 147.1 ppm. HRMS (ESI): *m/z* calcd. for $C_{14}H_{23}{}^{79}BrNSi^+$ [M+H$^+$]: 336.0778, found 336.0776, *m/z* calcd. for $C_{14}H_{23}{}^{81}BrNSi^+$ [M+H$^+$]: 338.0758, found 338.0758.

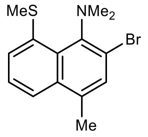

2-Bromo-*N,N*,4-trimethyl-8-(methylthio)naphthalen-1-amine: Pale yellow oil, yield: 291 mg (47%). ^{1}H NMR (400 MHz, $CDCl_3$): δ= 2.44 (s, 3 H), 2.62 (s, 3 H), 2.88 (s, 6 H), 7.28 (d, J = 7.6 Hz, 1 H), 7.43–7.51 (m, 2 H), 7.65 (dd, J = 8.2, 1.1 Hz, 1 H) ppm. ^{13}C{^{1}H} NMR (100 MHz, $CDCl_3$): δ= 18.0, 19.8, 42.2, 120.0, 121.3, 122.0, 126.0, 132.0, 132.6, 133.8, 134.4, 138.6, 144.6 ppm. HRMS (ESI): *m/z* calcd. for $C_{14}H_{17}{}^{79}BrNS^+$ [M+H$^+$]: 310.0260, found 310.0254, *m/z* calcd. for $C_{14}H_{17}{}^{81}BrNS^+$ [M+H$^+$]: 312.0240, found 312.0233.

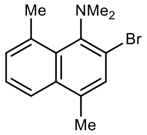

2-Bromo-*N*,*N*,4,8-tetramethylnaphthalen-1-amine: Colourless oil, yield: 372 mg (67%). ^{1}H NMR (400 MHz, $CDCl_3$): *δ*= 2.61 (s, 3 H), 2.86 (s, 6 H), 2.88 (s, 3 H), 7.31–7.35 (m, 1 H), 7.38 (dd, *J* = 8.2, 7.0 Hz, 1 H), 7.46 (s, 1 H), 7.79 (dd, *J* = 8.2, 1.6 Hz, 1 H) ppm. ^{13}C{^{1}H} NMR (100 MHz, $CDCl_3$): *δ*= 20.0, 24.3, 42.3, 123.3, 124.1, 125.7, 130.7, 131.8, 134.3, 134.4, 134.9, 135.8, 145.7 ppm. HRMS (ESI): *m/z* calcd. for $C_{14}H_{17}{}^{79}BrN^+$ [M+H$^+$]: 278.0539, found 278.0538, *m/z* calcd. for $C_{14}H_{17}{}^{81}BrN^+$ [M+H$^+$]: 280.0519, found 280.0518.

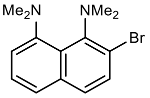

2-Bromo-*N*1,*N*1,*N*8,*N*8-tetramethylnaphthalene-1,8-diamine: was synthesized according to a previously described procedure.[6] The spectroscopic ^{1}H NMR data corresponds to that of the previously reported.[6] ^{1}H NMR (400 MHz, $CDCl_3$): *δ*= 2.76 (s, 6H), 3.02 (s, 6H), 7.08 (dd, *J* = 7.4, 0.9 Hz, 1H), 7.28–7.34 (m, 2H), 7.38 (dd, *J* = 8.0, 0.9 Hz, 1H), 7.51 (d, *J* = 8.7 Hz, 1H) ppm.

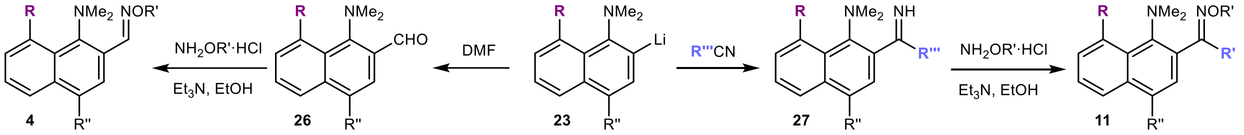

Scheme S3. General scheme for the synthesis of compounds **4** and **11**

1-(Dimethylamino)-4-methyl-2-naphthaldehydes (*general procedure*): *n*-Butyllithium (1.6 M solution in hexanes, 0.75 mL, 1.2 mmol, 1.2 equiv.) was added via syringe to a solution of 2-bromo-1-dimethylaminonaphthalene (1 mmol: 264 mg of 8-H, 336 mg of 8-$SiMe_3$, 310 mg of 8-SMe, 278 mg of 8-Me) in dry Et_2O (10 mL) in a flame-dried flask under argon atmosphere at room temperature. Resulting mixture was stirred for 1 h at the same temperature and then DMF (0.12 mL, 1.5 mmol, 1.5 equiv.) was added via syringe. Resulting mixture was stirred for 24 h at the room temperature and treated with water (10 mL). The products were extracted with CH_2Cl_2 (3×20 mL), the solvent was evaporated to dryness. The residue was purified by column chromatography on Al_2O_3 (2×20 cm) with *n*-hexane/CH_2Cl_2 (10:1, v/v) as the eluent. The yellow fraction with R_f = 0.5 gave corresponding aldehyde.

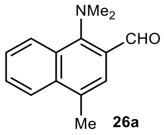

1-(Dimethylamino)-4-methyl-2-naphthaldehyde 26a: Yellow oil, yield: 192 mg (90%). ^{1}H NMR (400 MHz, $CDCl_3$): *δ*= 2.65 (s, 3 H), 3.22 (s, 6 H), 7.54–7.59 (m, 1 H), 7.61–7.66 (m, 1 H), 7.70 (s, 1 H), 7.99 (dd, *J* = 8.5, 1.5 Hz, 1 H), 8.26 (dd, *J* = 8.2, 1.6 Hz, 1 H), 10.53 (s, 1 H) ppm. ^{13}C{^{1}H} NMR (100 MHz, $CDCl_3$): *δ*= 19.3, 46.4, 123.8, 125.2, 126.0, 126.3, 128.6, 130.5, 131.9, 132.3, 137.2, 154.8, 192.6 ppm. HRMS (ESI): *m/z* calcd. for $C_{14}H_{16}NO^+$ [M+H$^+$]: 214.1227, found 214.1225.

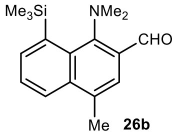

1-(Dimethylamino)-4-methyl-8-(trimethylsilyl)-2-naphthaldehyde 26b: Yellow oil, yield: 208 mg (73%). ^{1}H NMR (400 MHz, $CDCl_3$): *δ*= 0.30 (s, 9 H), 2.65 (s, 3 H), 2.98 (s, 6 H), 7.56 (dd, *J* = 8.3, 6.9 Hz, 1 H), 7.72 (s, 1 H), 7.90–7.94 (m, 2 H), 10.75 (s, 1 H) ppm. ^{13}C{^{1}H} NMR (100 MHz, $CDCl_3$): *δ*= 2.5, 19.5, 49.6, 124.9, 125.5, 127.6, 130.9, 132.4, 136.7, 136.8, 137.4, 140.1, 155.5, 192.1 ppm. HRMS (ESI): *m/z* calcd. for $C_{17}H_{24}NOSi^+$ [M+H$^+$]: 286.1622, found 286.1618.

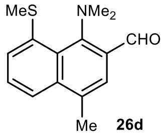

1-(Dimethylamino)-4-methyl-8-(methylthio)-2-naphthaldehyde 26d: Yellow oil, yield: 197 mg (76%). ^{1}H NMR (400 MHz, $CDCl_3$): *δ*= 2.44 (s, 3 H), 2.65 (s, 3 H), 2.93 (s, 6 H), 7.29 (d, *J* = 7.6 Hz, 1 H), 7.53–7.59 (m, 1 H), 7.70 (dd, *J* = 8.3, 1.1 Hz, 1 H), 7.78 (s, 1 H), 10.46 (s, 1 H) ppm. ^{13}C{^{1}H} NMR (100 MHz, $CDCl_3$): *δ*= 17.7, 20.3, 46.3, 120.2, 121.5, 124.4, 128.7, 130.7, 133.5, 133.7, 138.6, 141.2, 154.2, 191.3 ppm. HRMS (ESI): *m/z* calcd. for $C_{15}H_{18}NOS^+$ [M+H$^+$]: 260.1104, found 260.1103.

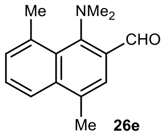

1-(Dimethylamino)-4,8-dimethyl-2-naphthaldehyde 26e: Yellow oil, yield: 193 mg (85%). ^{1}H NMR (400 MHz, $CDCl_3$): *δ*= 2.66 (d, *J* = 1.0 Hz, 3 H), 2.89 (s, 3 H), 3.03 (s, 6 H), 7.37 (d, *J* = 7.0 Hz, 1 H), 7.50 (dd, *J* = 8.4, 7.0 Hz, 1 H), 7.75 (d, *J* = 1.0 Hz, 1 H), 7.86 (d, *J* = 8.4 Hz, 1 H), 10.42 (s, 1 H) ppm. ^{13}C{^{1}H} NMR (100 MHz, $CDCl_3$): *δ*= 20.3, 23.8, 46.4, 123.5, 124.0, 128.3, 130.8, 132.6, 133.4, 134.3, 137.4, 138.7, 155.5, 192.3 ppm. HRMS (ESI): *m/z* calcd. for $C_{15}H_{18}NO^+$ [M+H$^+$]: 228.1383, found 228.1387.

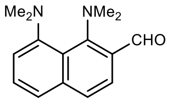

26c

1,8-Bis(dimethylamino)-2-naphthaldehyde 26c: was synthesized according to a previously described procedure.[7] The spectroscopic ^{1}H NMR data corresponds to that of the previously reported.[7] ^{1}H NMR (400 MHz, $CDCl_3$): δ= 2.79 (s, 6H), 3.24 (s, 6H), 7.03 (d, *J* = 7.4, 0.8, 1H), 7.31–7.42 (m, 3H), 7.69 (m, 2H), 7.45 (d, *J* = 8.5 Hz, 1H), 10.24 (s, 1 H) ppm.

2-(Imino(*p*-tolyl)methyl)-*N*,*N*,4-trimethylnaphthalen-1-amines (*general procedure*): *n*-Butyllithium (1.6 M solution in hexanes, 0.38 mL, 0.60 mmol, 1.2 equiv.) was added via syringe to a solution corresponding 2-bromo-4-methyl-1-dimethylaminonaphthalene (0.50 mmol: 132 mg of 8-H, 168 mg of 8-$SiMe_3$, 139 mg of 8-Me) in dry Et_2O (15 mL) in a flame-dried flask under argon atmosphere at room temperature. Resulting mixture was stirred for 1 h at the same temperature and then solution of *p*-tolunitrile (70 mg, 0.60 mmol, 1.2 equiv.) in E_2O (5 mL) was added via syringe. Resulting mixture was stirred for 24 h at the room temperature and treated with water (10 mL). The products were extracted with CH_2Cl_2 (3×20 mL), the solvent was evaporated to dryness. The residue was purified by thin layer chromatography on Al_2O_3 with *n*-hexane/Et_2O (2:1, v/v) as the eluent. The yellowish fraction with R_f = 0.5 gave the corresponding imine.

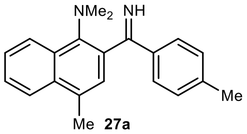

27a

2-(Imino(*p*-tolyl)methyl)-*N*,*N*,4-trimethylnaphthalen-1-amine 27a: Colourless oil, yield: 94 mg (62%). ^{1}H NMR (400 MHz, $CDCl_3$): *δ*= 2.40 (s, 3 H), 2.64 (s, 3 H), 2.80 (s, 6 H), 7.07 (s, 1 H), 7.20 (d, *J* = 8.0 Hz, 2 H), 7.54–7.59 (m, 2 H), 7.66 (d, *J* = 8.0 Hz, 2 H), 7.97–8.03 (m, 1 H), 8.23–8.28 (m, 1 H), 9.17 (s, 1 H) ppm. ^{13}C{^{1}H} NMR (100 MHz, $CDCl_3$): *δ*= 19.2, 21.5, 44.6, 124.8, 125.7, 125.8, 126.4, 127.1, 128.6, 129.0, 130.2, 131.9, 133.8, 134.2, 136.4, 141.1, 145.6, 179.0 ppm. HRMS (ESI): *m/z* calcd. for $C_{21}H_{23}N_2^+$ [M+H$^+$]: 303.1856, found 303.1861.

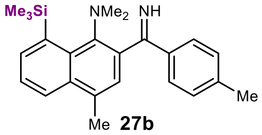

27b

2-(Imino(*p*-tolyl)methyl)-*N*,*N*,4-trimethyl-8-(trimethylsilyl)naphthalen-1-amine 27e: Colourless oil, yield: 114 mg (61%). ^{1}H NMR (400 MHz, $CDCl_3$): *δ*= 0.26 (s, 9 H), 2.41 (s, 3 H), 2.56 (s, 3 H), 2.61 (s, 6 H), 6.91 (s, 1 H), 7.23 (d, *J* = 8.0 Hz, 2 H), 7.51 (dd, *J* = 8.3, 6.8 Hz, 1 H), 7.69–7.87 (m, 2 H), 7.89 (dd, *J* = 6.9, 1.3 Hz, 1 H), 7.94 (dd, *J* = 8.3, 1.3 Hz, 1 H), 9.33 (s, 1 H) ppm. ^{13}C{^{1}H} NMR (100 MHz, $CDCl_3$): *δ*= 2.3, 19.3, 21.6, 22.8, 31.7, 125.1, 125.5, 126.9, 128.9, 129.3, 130.8, 132.8, 134.0, 136.1, 136.2, 136.5, 138.6, 141.6, 146.1, 179.0 ppm. HRMS (ESI): *m/z* calcd. for $C_{24}H_{31}N_2Si^+$ [M+H$^+$]: 375.2252, found 375.2268.

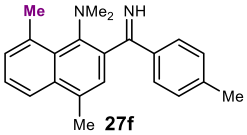

27f

2-(Imino(*p*-tolyl)methyl)-*N*,*N*,4,8-tetramethylnaphthalen-1-amine 27f: Pale orange oil, yield: 93 mg (59%). ^{1}H NMR (400 MHz, $CDCl_3$): *δ*= 2.40 (s, 3 H), 2.61 (s, 6 H), 2.62 (s, 3 H), 2.84 (s, 3 H), 7.01 (s, 1 H), 7.20 (d, *J* = 7.9 Hz, 2 H), 7.33 (d, *J* = 7.0 Hz, 1 H), 7.43 (dd, *J* = 8.3, 7.0 Hz, 1 H), 7.68 (d, *J* = 7.9 Hz, 2 H), 7.86 (d, *J* = 8.3 Hz, 1 H), 9.39 (s, 1 H) ppm. ^{13}C{^{1}H} NMR (100 MHz, $CDCl_3$): *δ*= 20.1, 21.6, 23.5, 44.3, 123.0, 126.0, 126.9, 128.7, 129.0, 130.2, 131.1, 132.4, 135.5, 136.2, 136.4, 137.1, 141.3, 145.5, 178.9 ppm. HRMS (ESI): *m/z* calcd. for $C_{22}H_{25}N_2^+$ [M+H$^+$]: 317.2013, found 317.2008.

2-(Imino(aryl)methyl)-N1,N1,N8,N8-tetramethylnaphthalene-1,8-diamine *(general procedure)*[2]: *n*-Butyllithium (1.6 M solution in hexanes, 1.54 mL, 2.4 mmol, 1.2 equiv.) was added via syringe to a solution of 2-bromo-1,8-bis(dimethylamino)naphthalene (584 mg, 2 mmol) in dry Et_2O (3 mL) in a flame-dried flask under argon atmosphere at −24 °C. The mixture was stirred for 1 h at the same temperature. A solution of the corresponding nitrile (benzonitrile for the **27d** or *p*-tolunitrile for the **27h** synthesis) in dry Et_2O was added via syringe and a resulting mixture was stirred overnight at −24 °C. Then, a reaction mixture was quenched with water, organic phase was separated. The water layer was extracted with CH_2Cl_2 (3×10 mL). The combined organic extracts were dried over Na_2SO_4 and filtered off. The solvent was evaporated to dryness. The imines **27d** and **27h** were then purified by recrystallization.

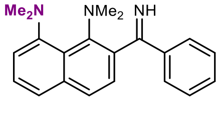

27d

2-(Imino(phenyl)methyl)-*N*1,*N*1,*N*8,*N*8-tetramethylnaphthalene-1,8-diamine 27d: **27d** was recrystallized from Et_2O and obtained as yellowish crystals in 69 % yield (437 mg). The spectroscopic ^{1}H NMR data corresponds to that of the previously reported.[2] ^{1}H NMR (400 MHz, $CDCl_3$): δ= 2.65 (s, 6H), 2.76 (s, 6H), 7.03 (dd, *J* = 7.4, 0.8 Hz, 1H), 7.21 (br d, *J* = 7.6 Hz, 1H), 7.32–7.38 (m, 3H), 7.39–7.43 (m, 2H), 7.45 (d, *J* = 8.3 Hz, 1H), 7.53–7.70 (br m, 2H), 9.54 (s, 1H) ppm.

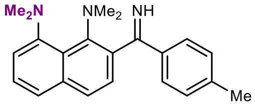

27h

2-(Imino(*p*-tolyl)methyl)-*N*1,*N*1,*N*8,*N*8-tetramethylnaphthalene-1,8-diamine 27h: **27h** was recrystallized from a mixture of hexane with a drop of AcOEt. The product was obtained as yellowish solid with mp = 110−112 °C in 45 % yield (299 mg). ^{1}H NMR (400 MHz, $CDCl_3$): δ = 2.38 (s, 3H), 2.68 (s, 6H), 2.78 (s, 6H), 7.03 (d, *J* = 7.4, 0.8 Hz, 1H), 7.13–7.22 (m, 3H), 7.32–7.38 (m, 1H), 7.38–7.48 (m, 2H), 7.40–7.50 (m, 2H), 9.28 (s, 1H) ppm. ^{13}C{^{1}H} NMR (100 MHz, $CDCl_3$): δ = 21.5, 44.5, 45.0, 114.1, 122.1, 122.8, 123.1, 126.4, 127.3, 128.4, 128.9, 132.7, 136.9, 138.6, 140.9, 147.4, 152.2, 180.1 ppm. HRMS (ESI): *m/z* calcd. for $C_{22}H_{26}N_3^+$ [M+H$^+$]: 332.2121, found 332.2119.

Copies of ^{1}H and ^{13}C NMR spectra of newly obtained compounds

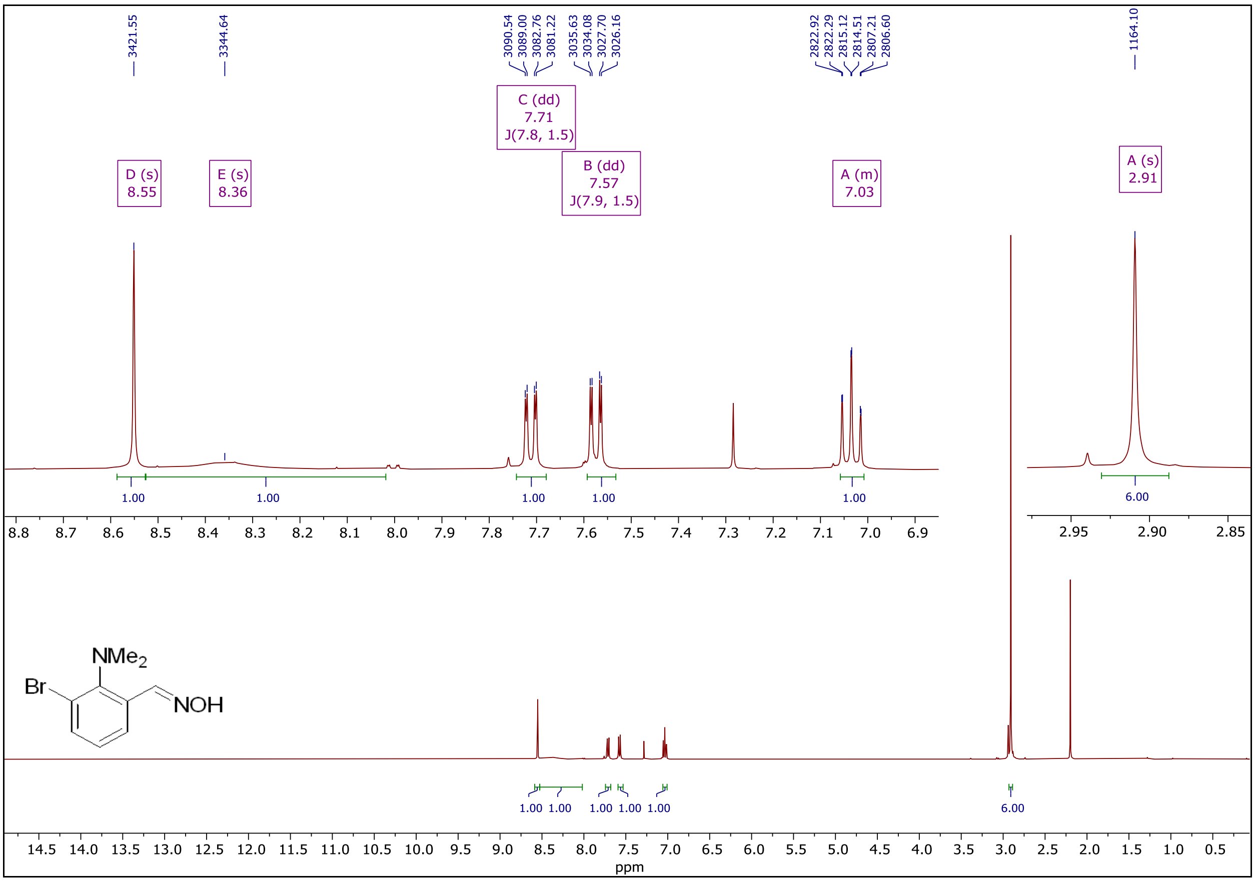

Fig. S1. ^{1}H NMR spectrum of compound **1c** (400 MHz, $CDCl_3$).

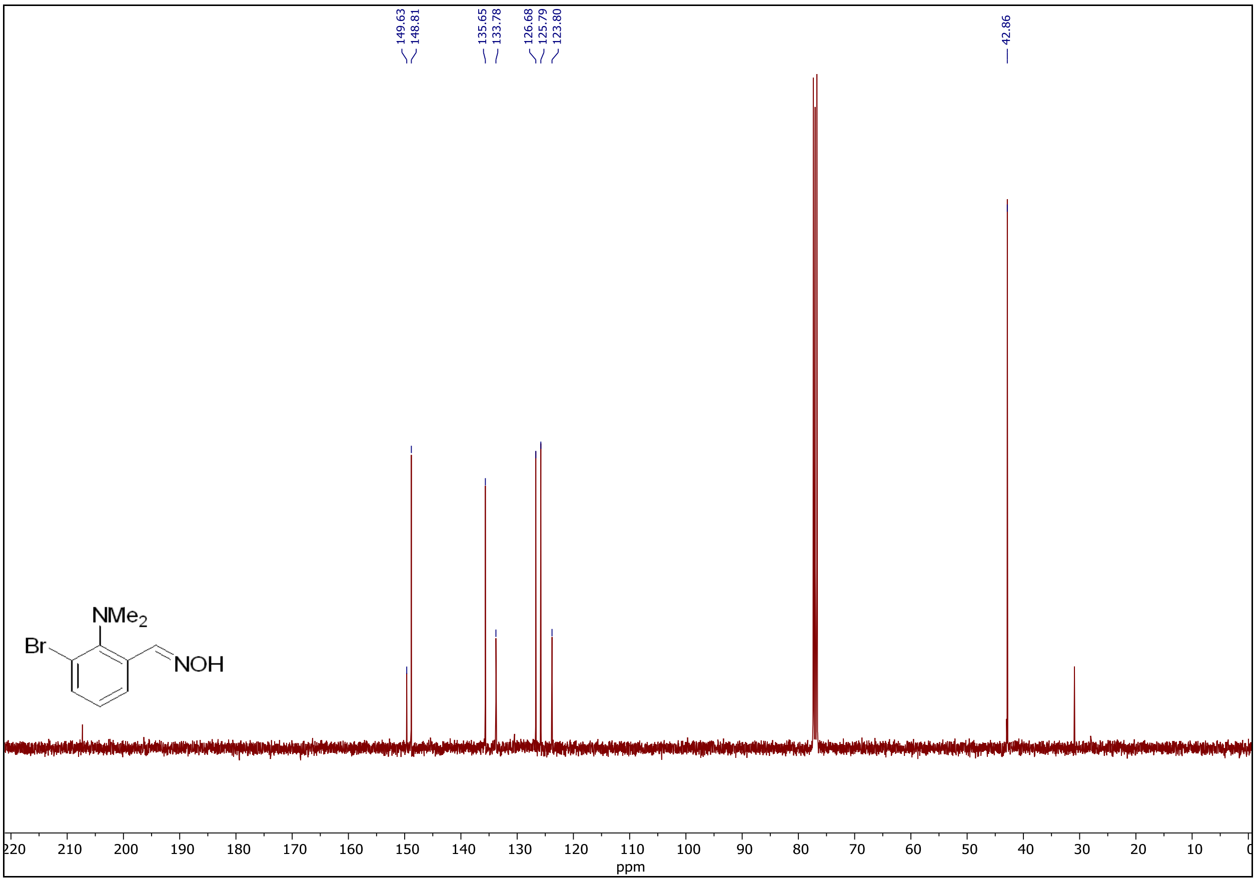

Fig. S2. ^{13}C{^{1}H} NMR spectrum of compound **1c** (100 MHz, $CDCl_3$).

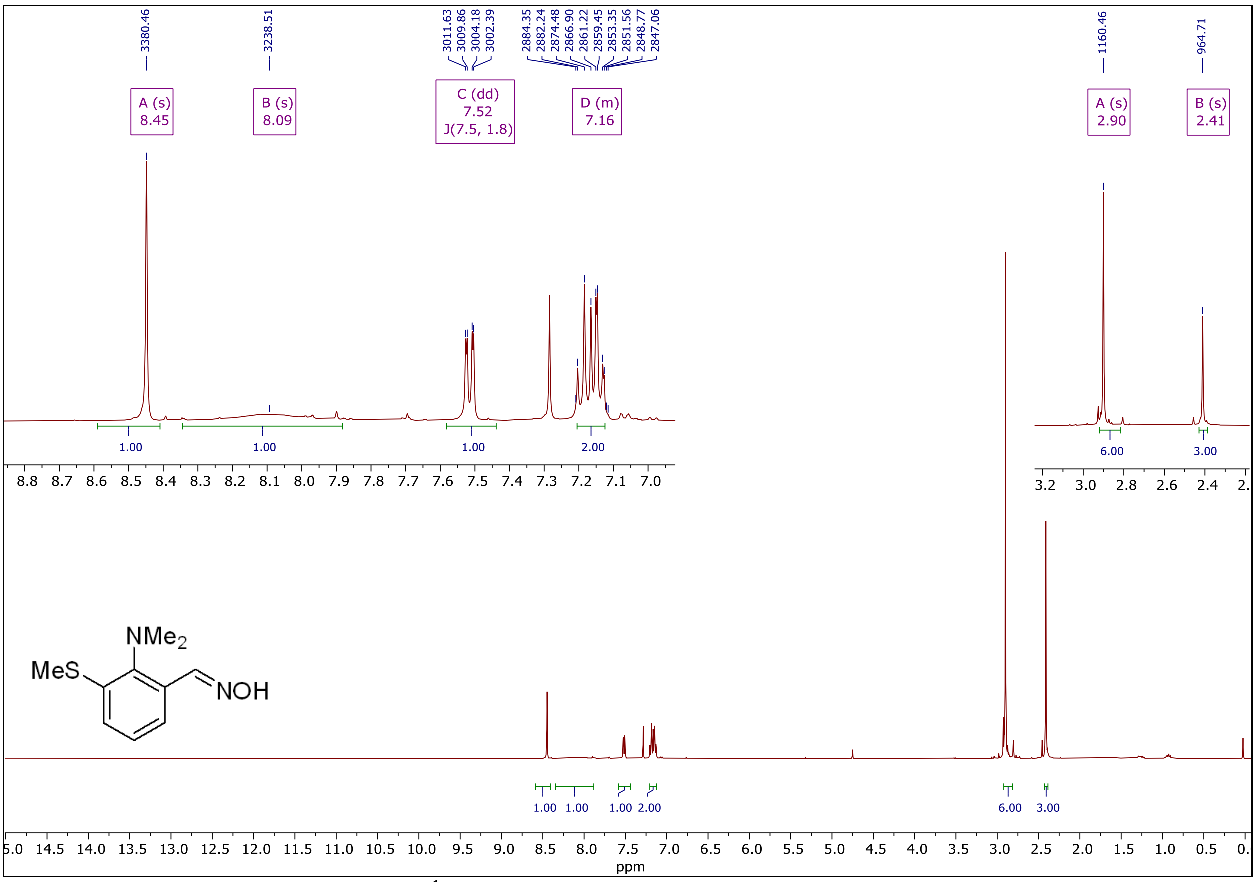

Fig. S3. ^{1}H NMR spectrum of compound **1d** (400 MHz, $CDCl_3$).

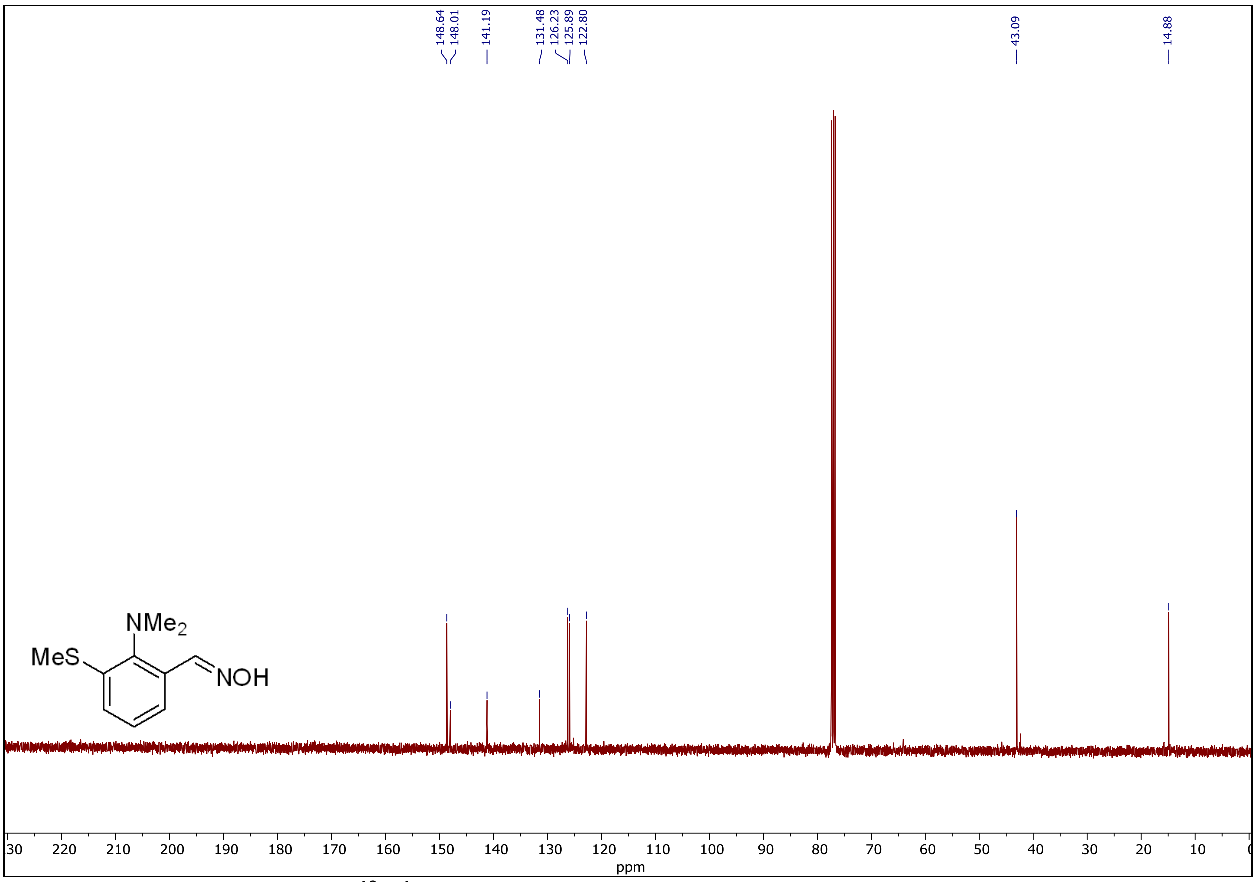

Fig. S4. $^{13}C\{^{1}H\}$ NMR spectrum of compound **1d** (100 MHz, $CDCl_3$).

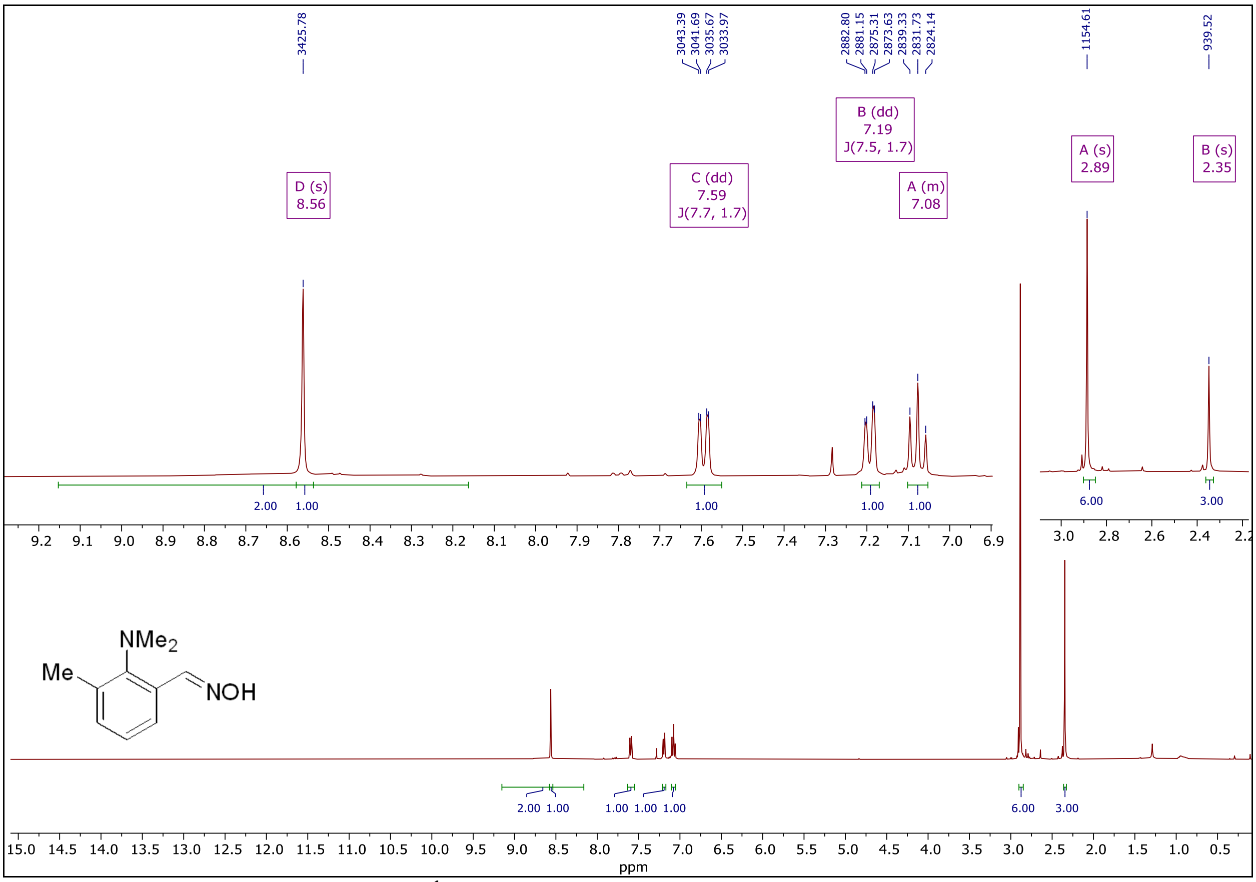

Fig. S5. ^{1}H NMR spectrum of compound **1e** (400 MHz, $CDCl_3$).

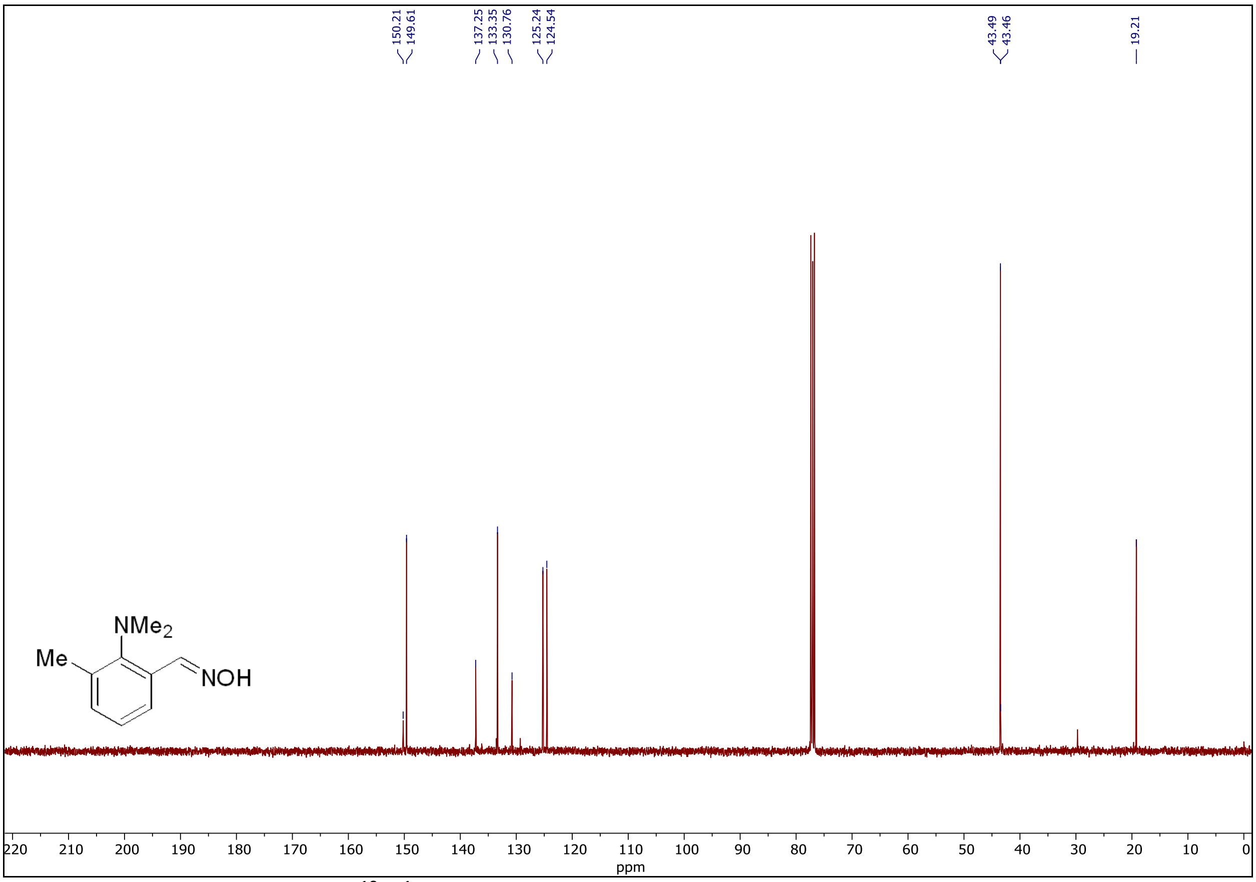

Fig. S6. ^{13}C{^{1}H} NMR spectrum of compound **1e** (100 MHz, $CDCl_3$).

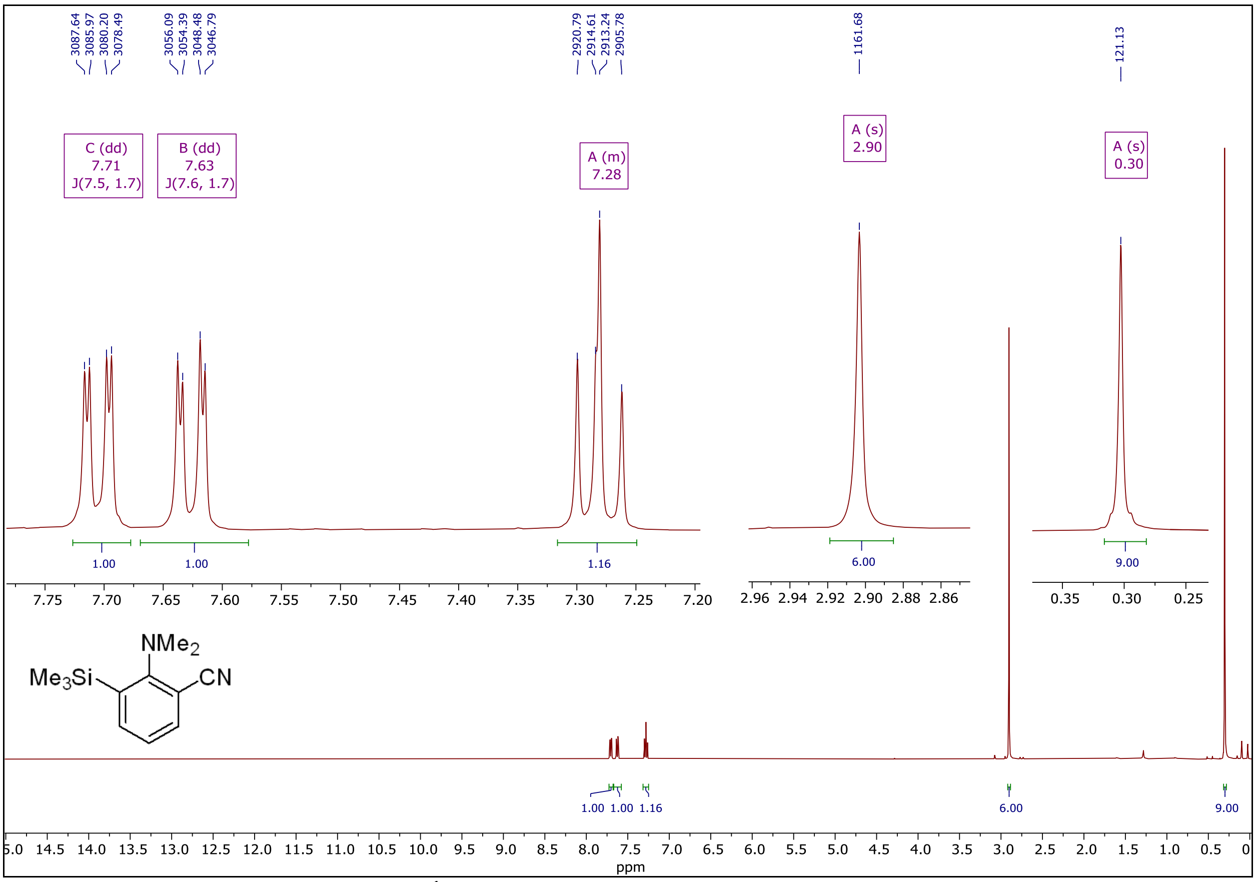

Fig. S7. ^{1}H NMR spectrum of compound **2b** (400 MHz, $CDCl_3$).

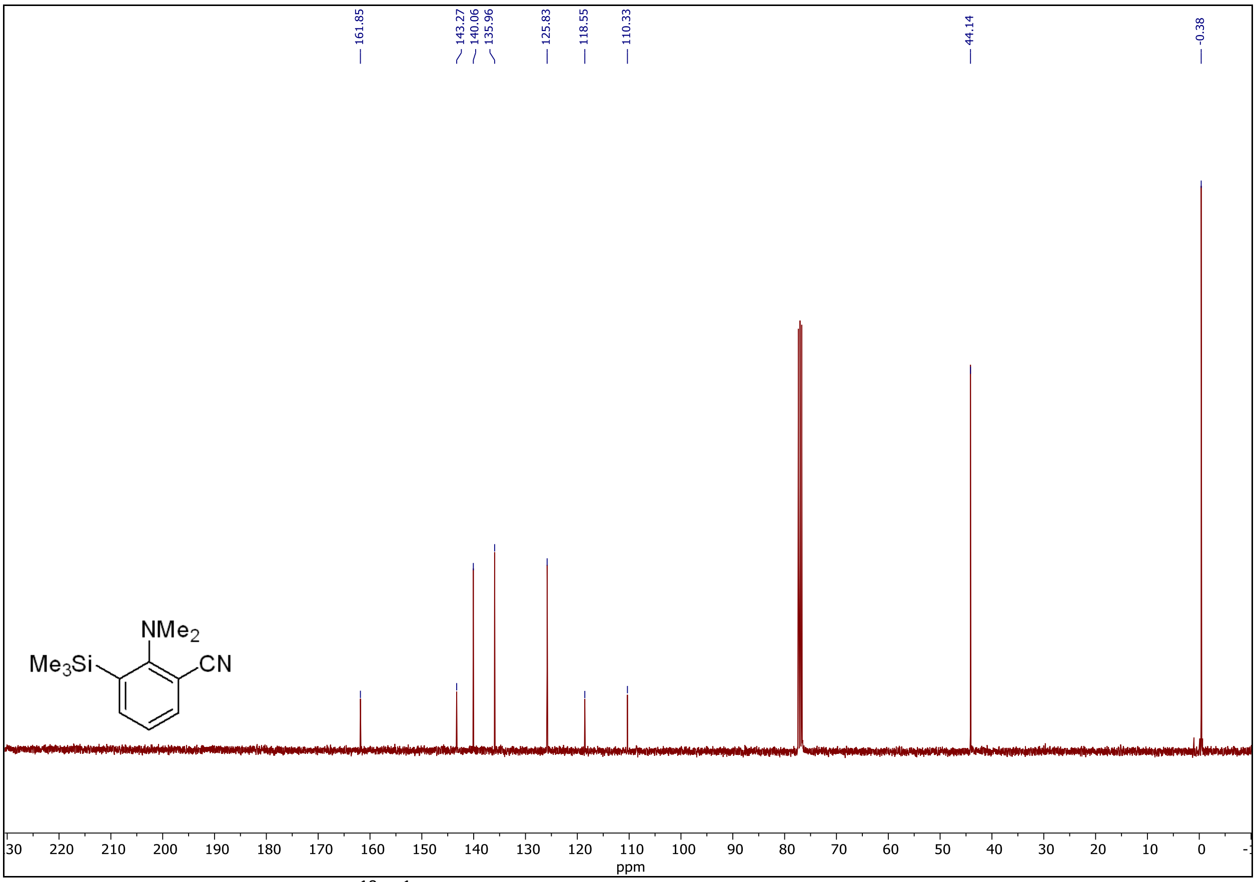

Fig. S8. $^{13}C\{^{1}H\}$ NMR spectrum of compound **2b** (100 MHz, $CDCl_3$).

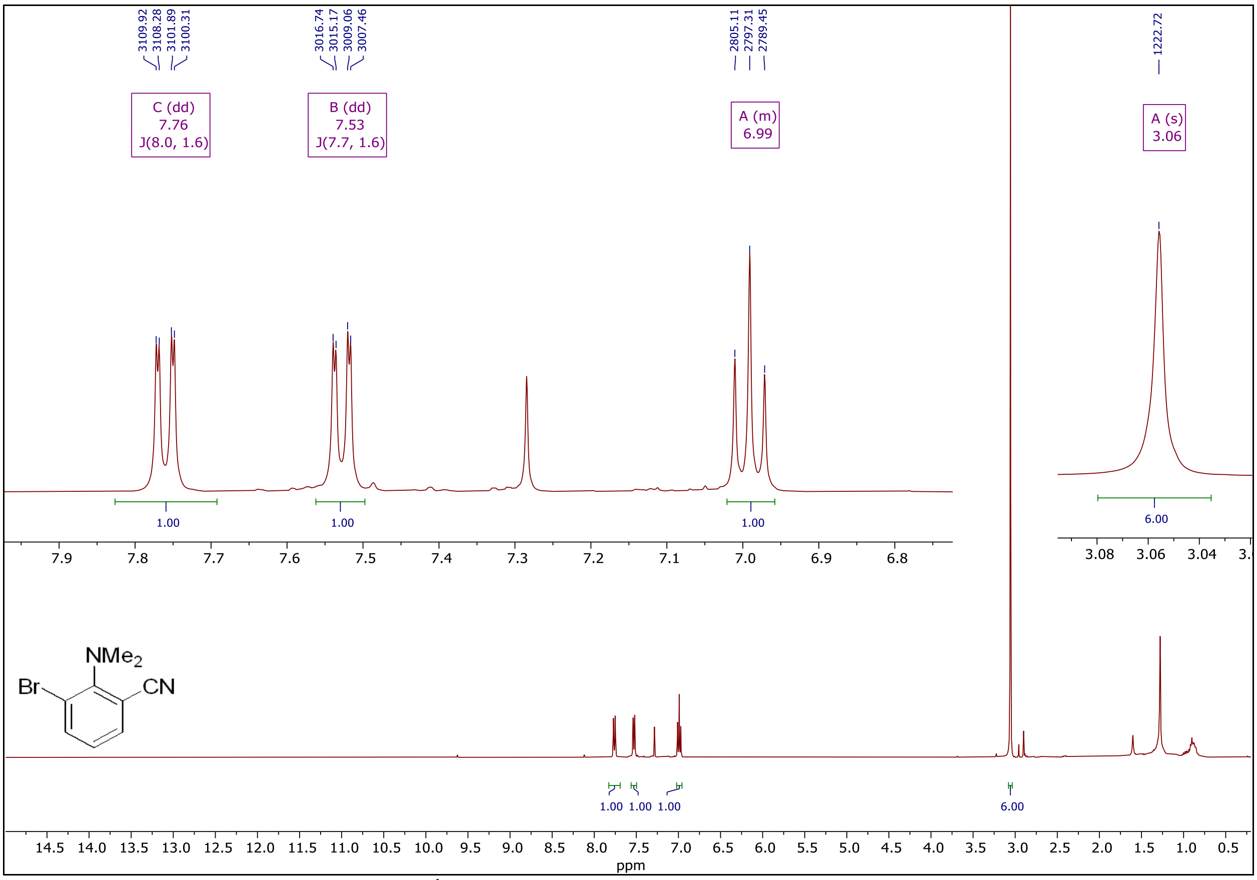

Fig. S9. ^{1}H NMR spectrum of compound **2c** (400 MHz, $CDCl_3$).

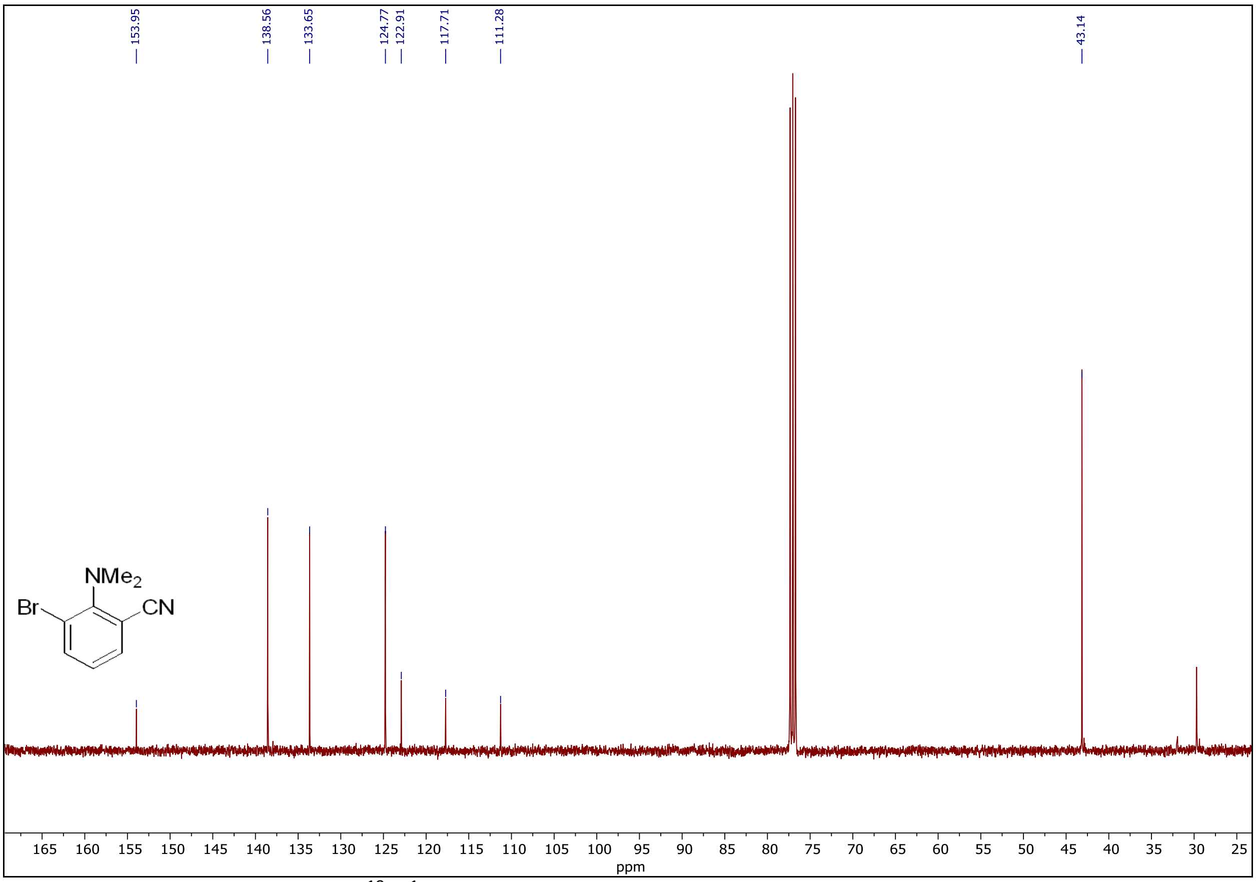

Fig. S10. $^{13}C\{^{1}H\}$ NMR spectrum of compound **2c** (100 MHz, $CDCl_3$).

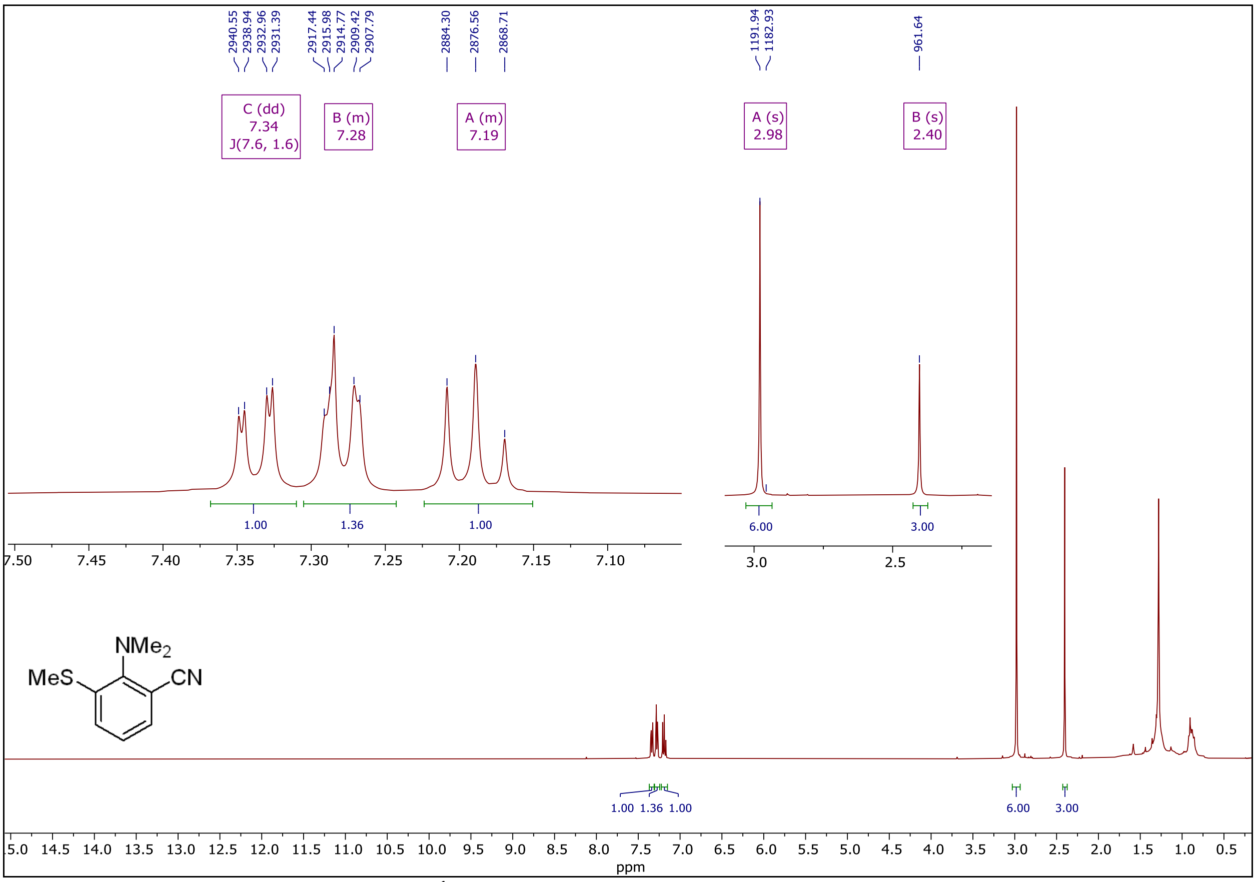

Fig. S11. ^{1}H NMR spectrum of compound **2d** (400 MHz, $CDCl_3$).

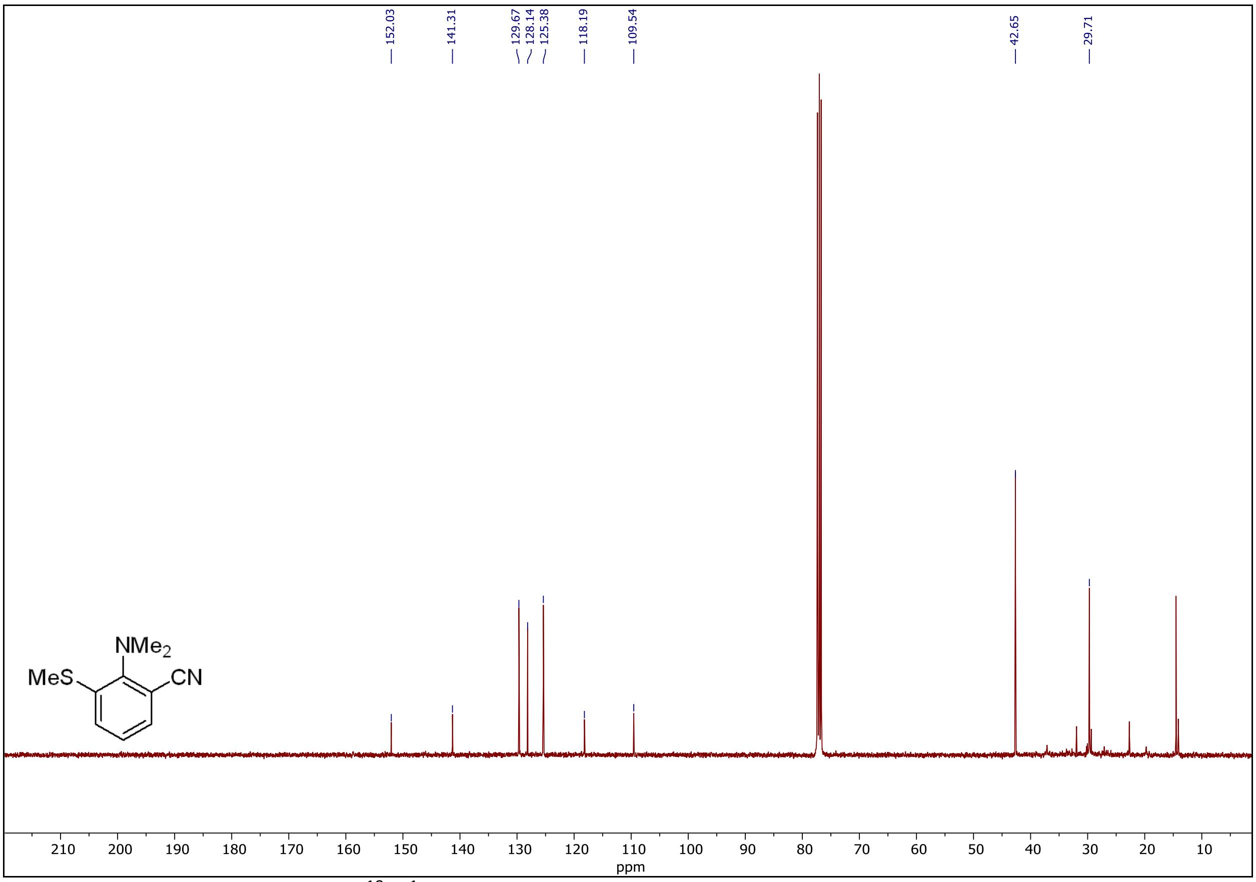

Fig. S12. $^{13}C\{^{1}H\}$ NMR spectrum of compound **2d** (100 MHz, $CDCl_3$).

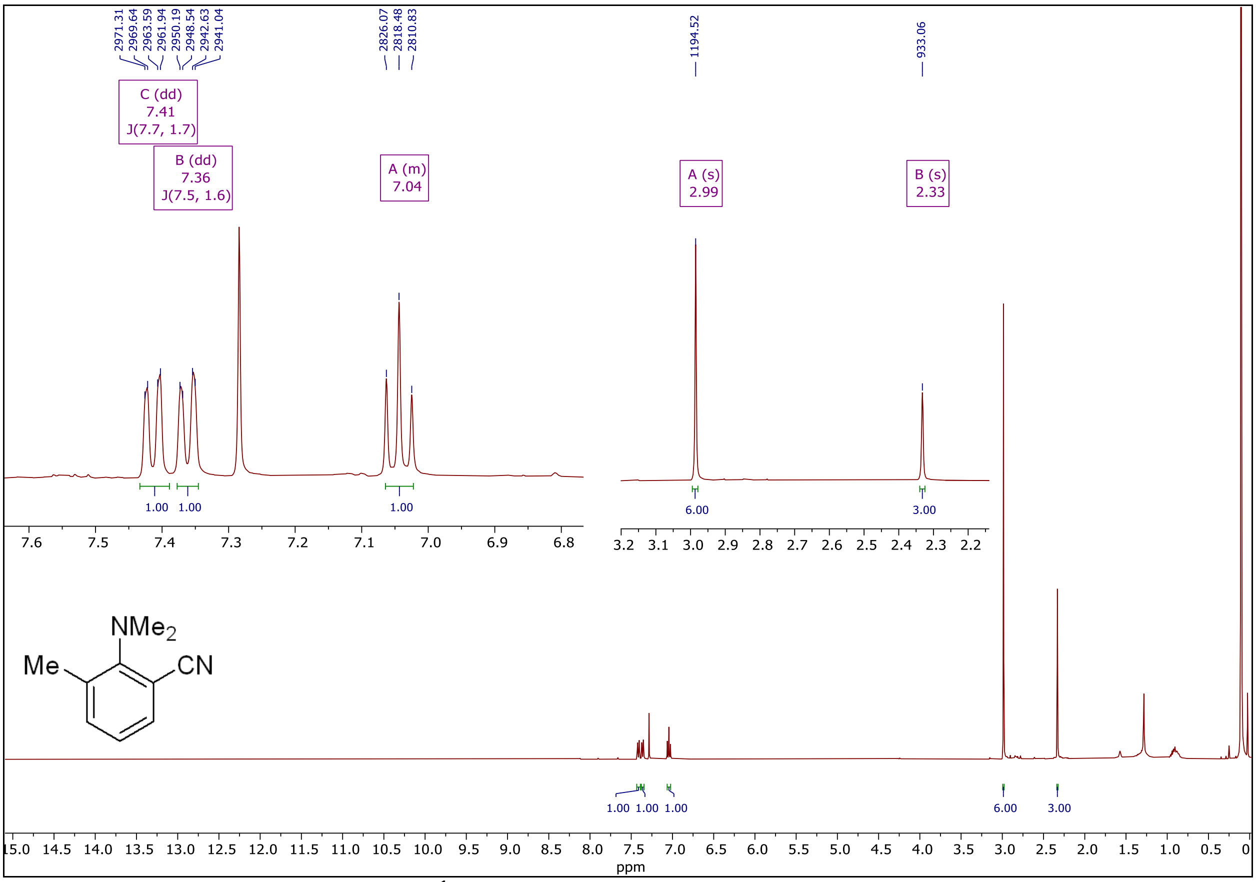

Fig. S13. ^{1}H NMR spectrum of compound **2e** (400 MHz, $CDCl_3$).

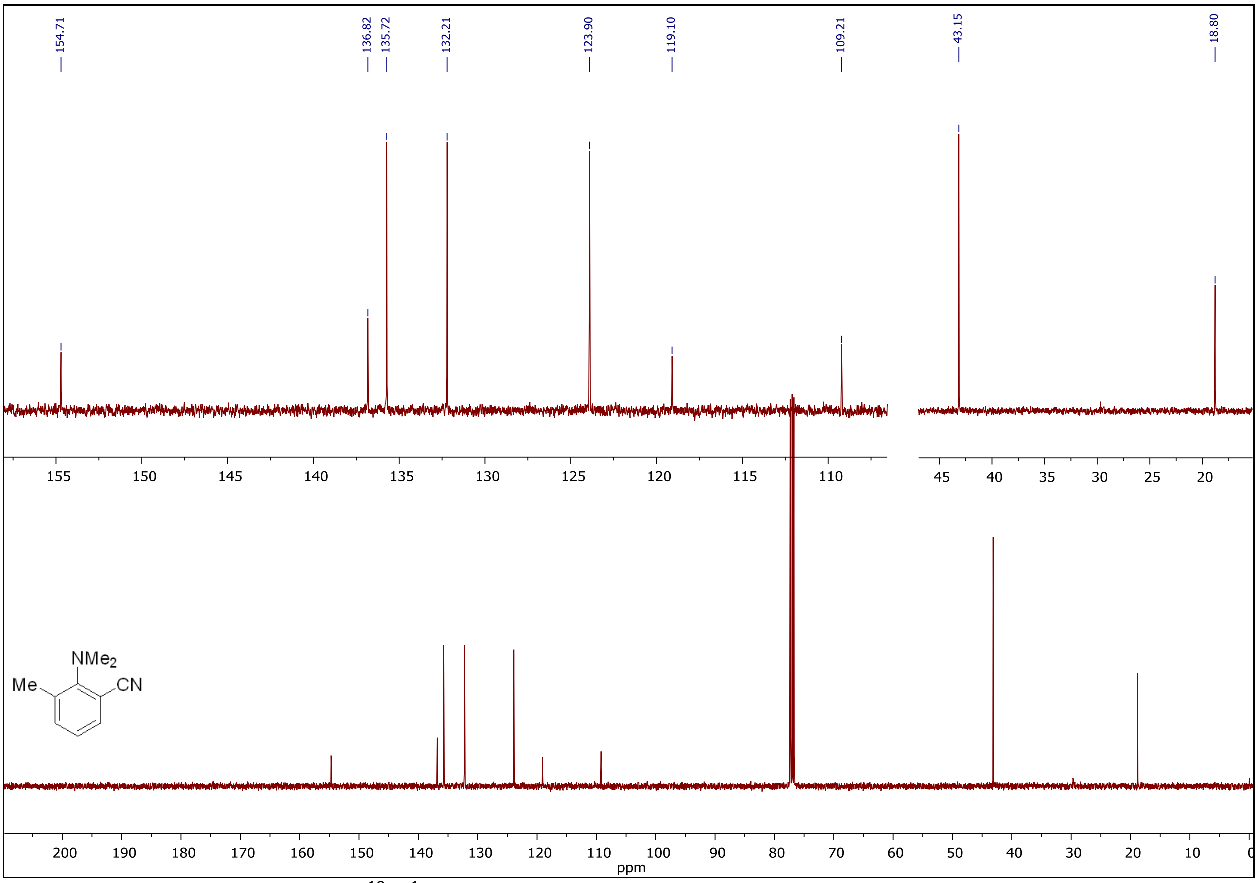

Fig. S14. ^{13}C{^{1}H} NMR spectrum of compound **2e** (100 MHz, $CDCl_3$).

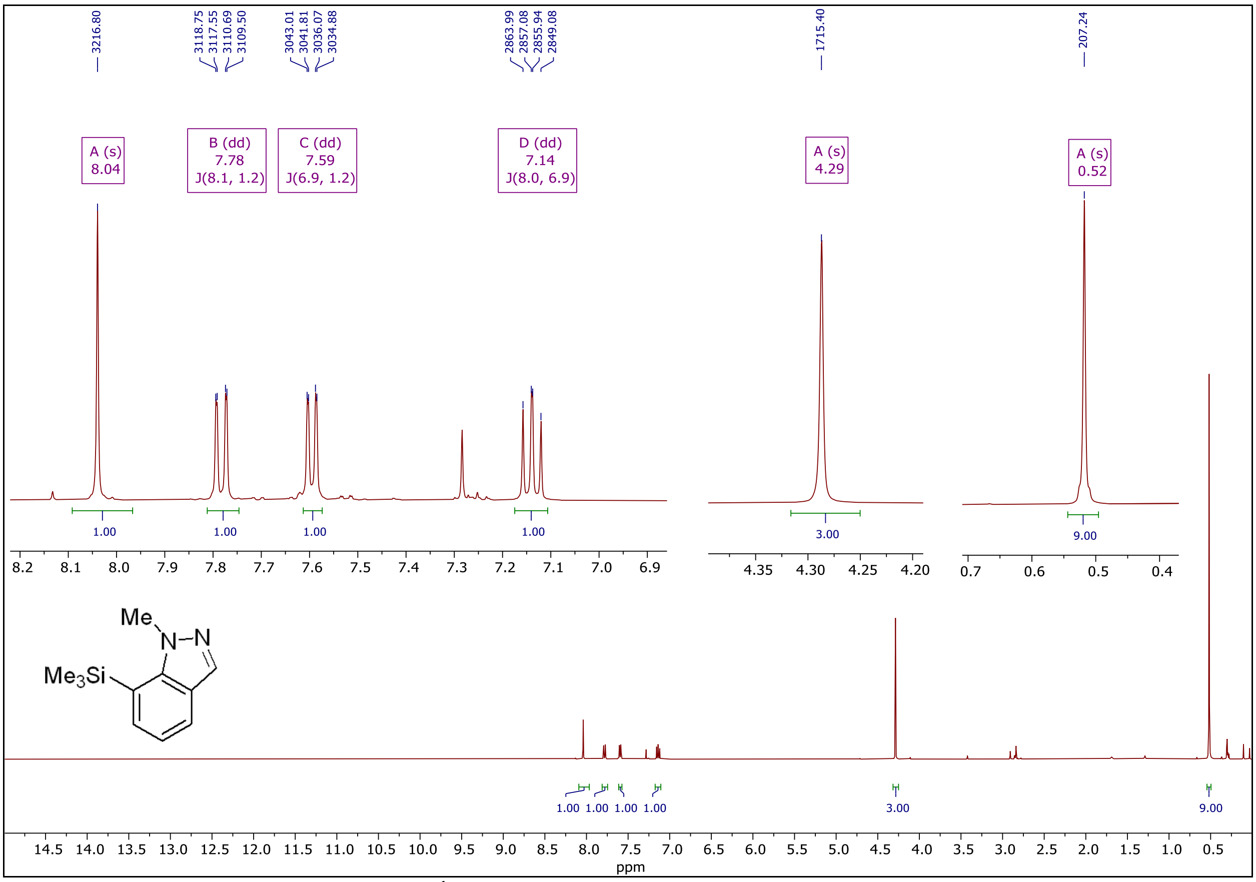

Fig. S15. ^{1}H NMR spectrum of compound **3b** (400 MHz, $CDCl_3$).

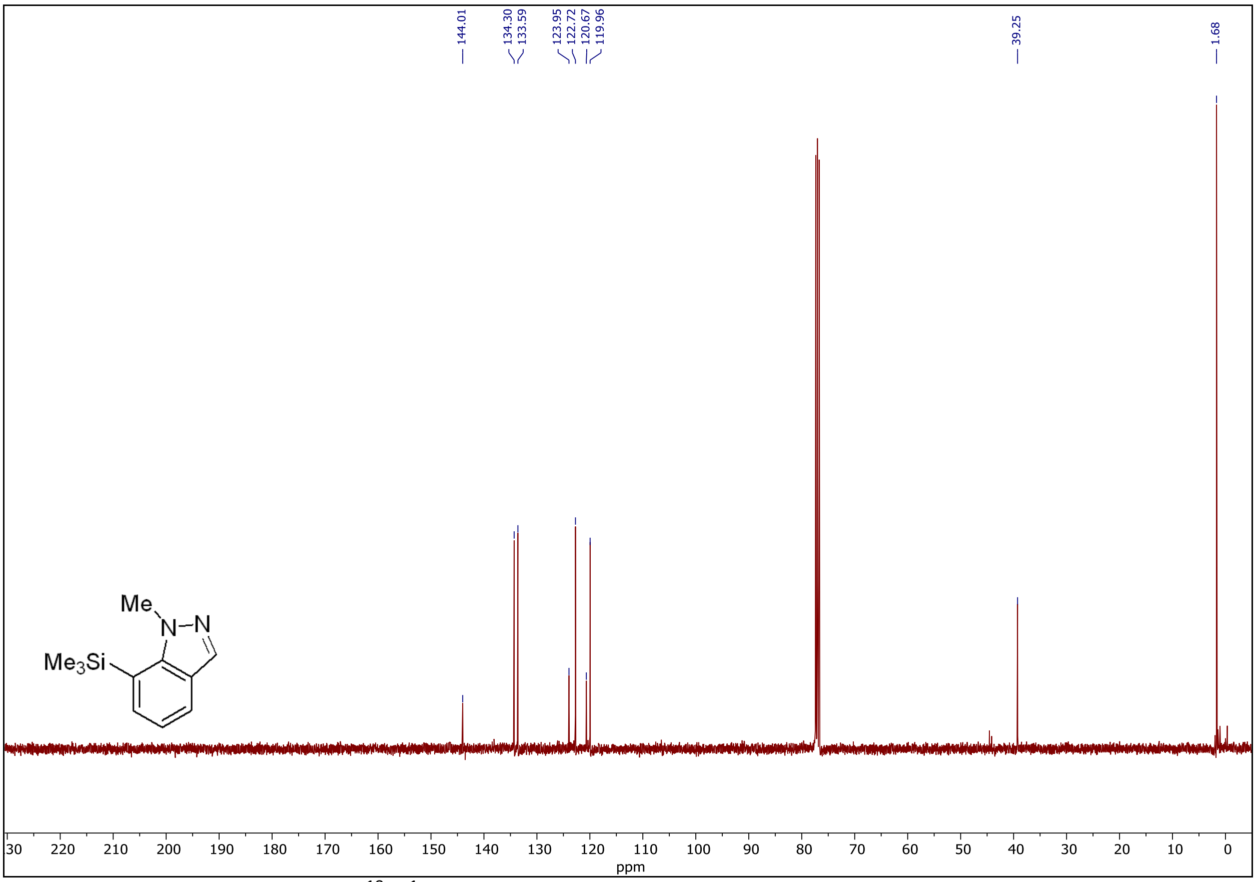

Fig. S16. $^{13}C\{^{1}H\}$ NMR spectrum of compound **3b** (100 MHz, $CDCl_3$).

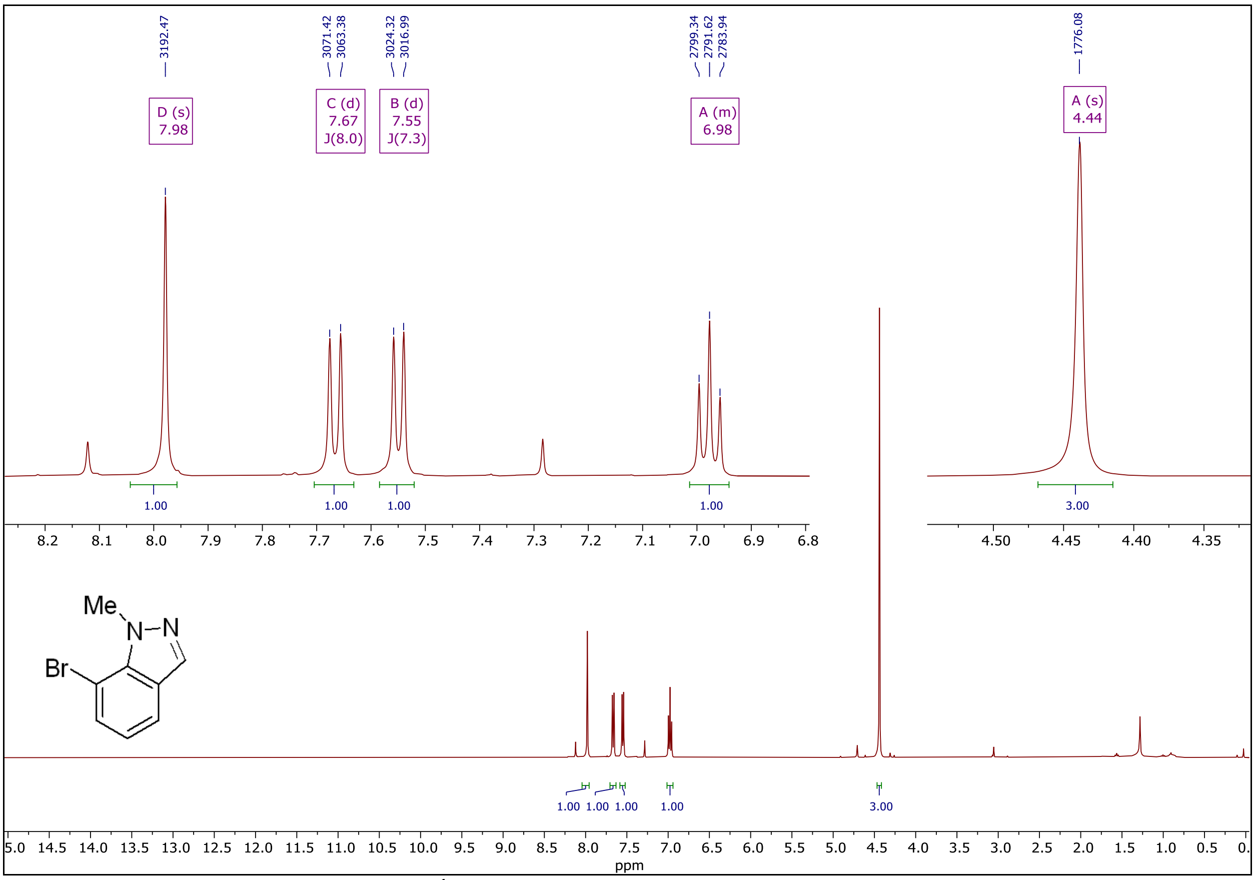

Fig. S17. ^{1}H NMR spectrum of compound **3c** (400 MHz, $CDCl_3$).

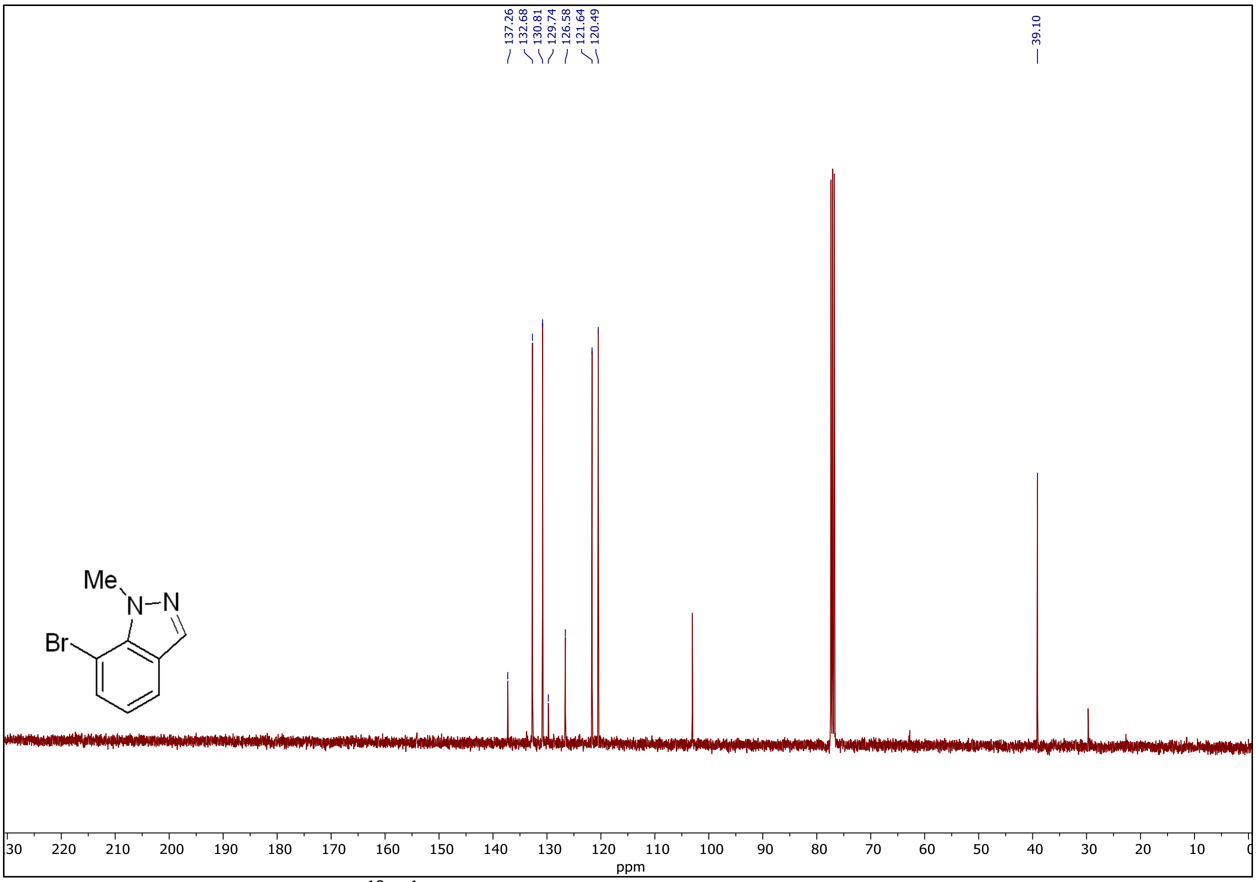

Fig. S18. $^{13}C\{^1H\}$ NMR spectrum of compound **3c** (100 MHz, $CDCl_3$).

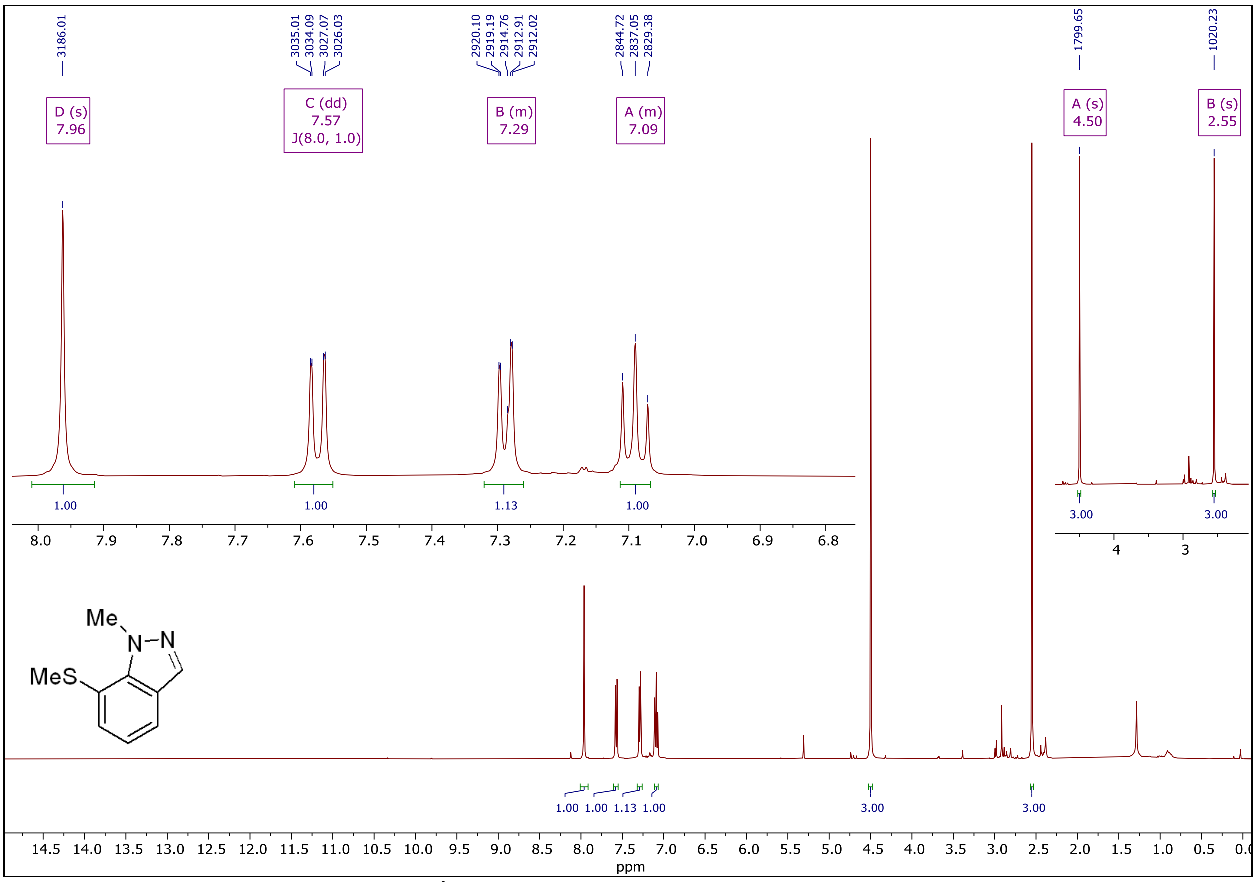

Fig. S19. ^{1}H NMR spectrum of compound **3d** (400 MHz, $CDCl_3$).

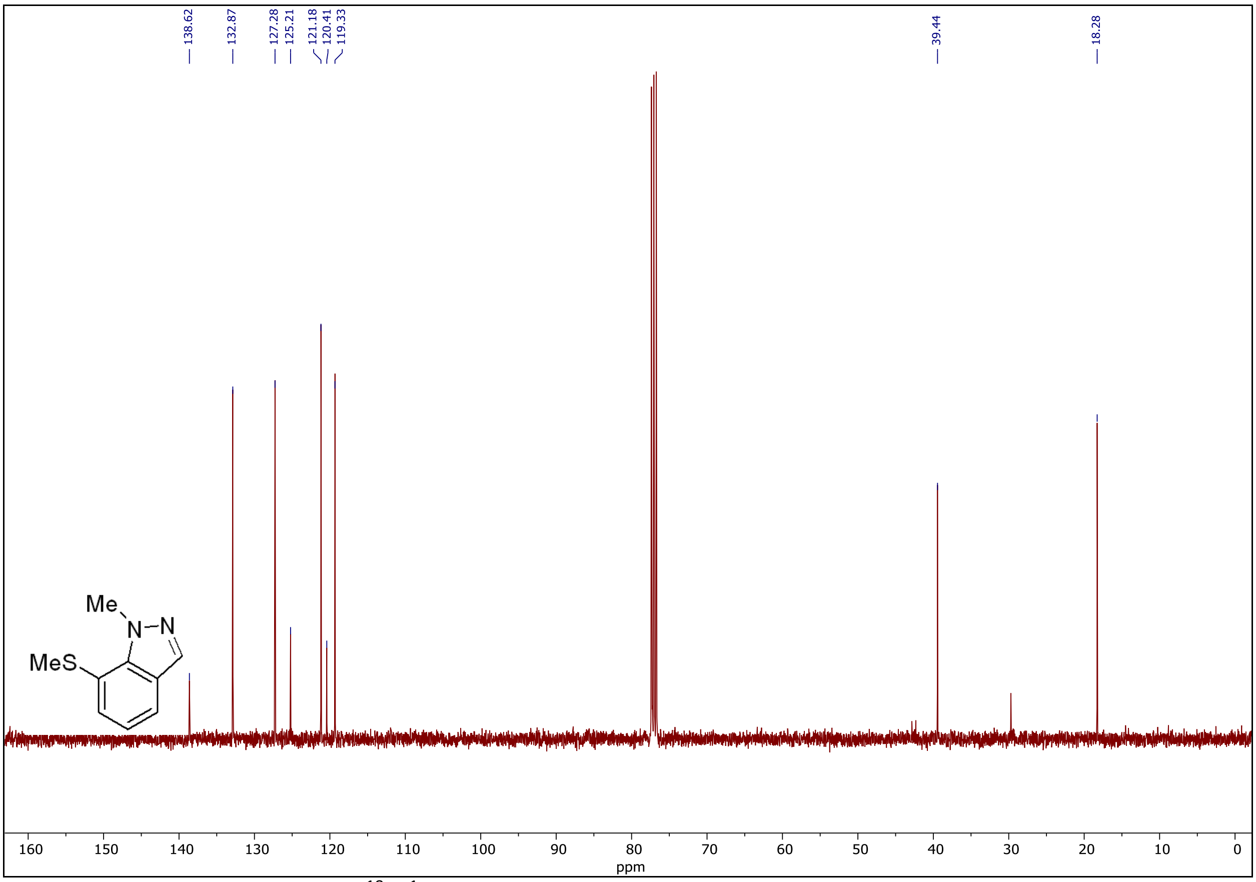

Fig. S20. ^{13}C{^{1}H} NMR spectrum of compound **3d** (100 MHz, $CDCl_3$).

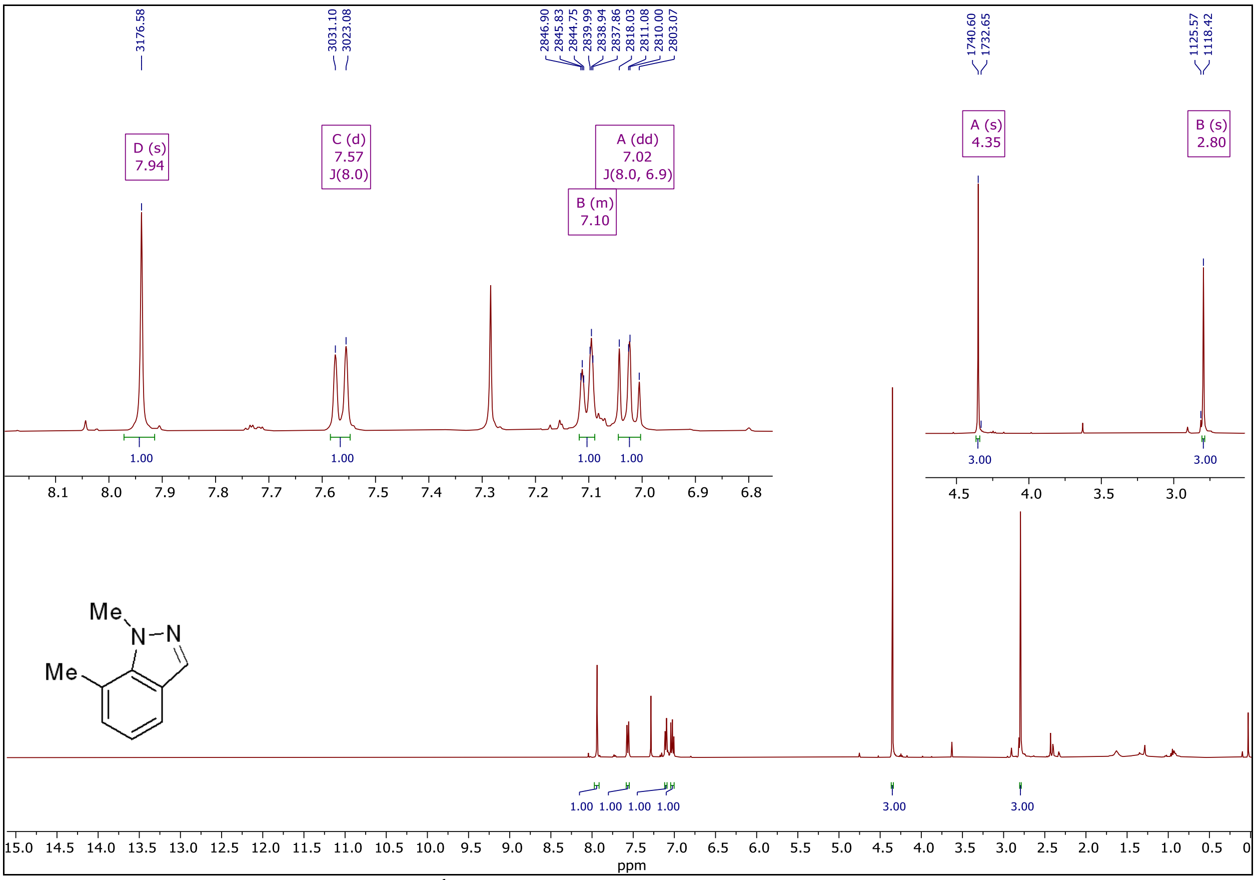

Fig. S21. ^{1}H NMR spectrum of compound **3e** (400 MHz, $CDCl_3$).

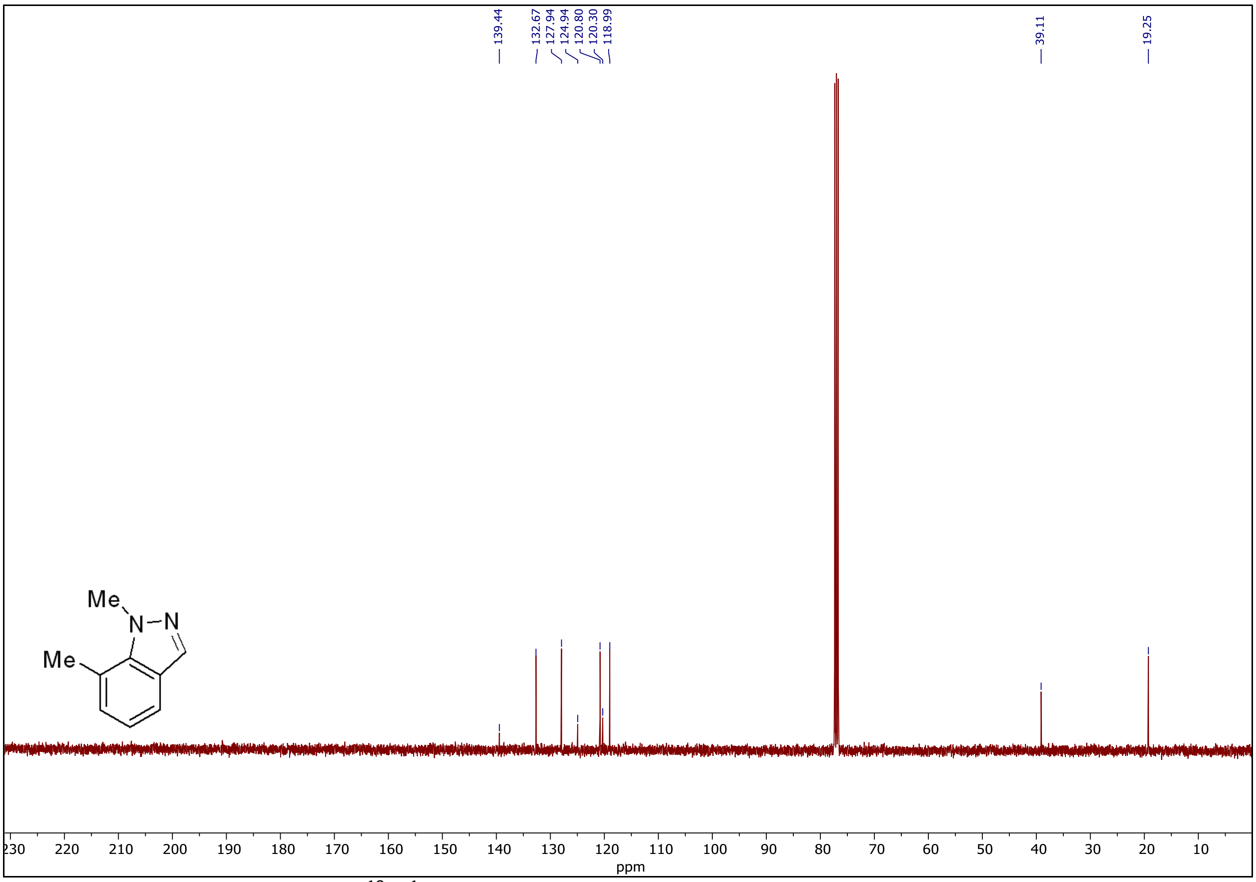

Fig. S22. ^{13}C{^{1}H} NMR spectrum of compound **3e** (100 MHz, $CDCl_3$).

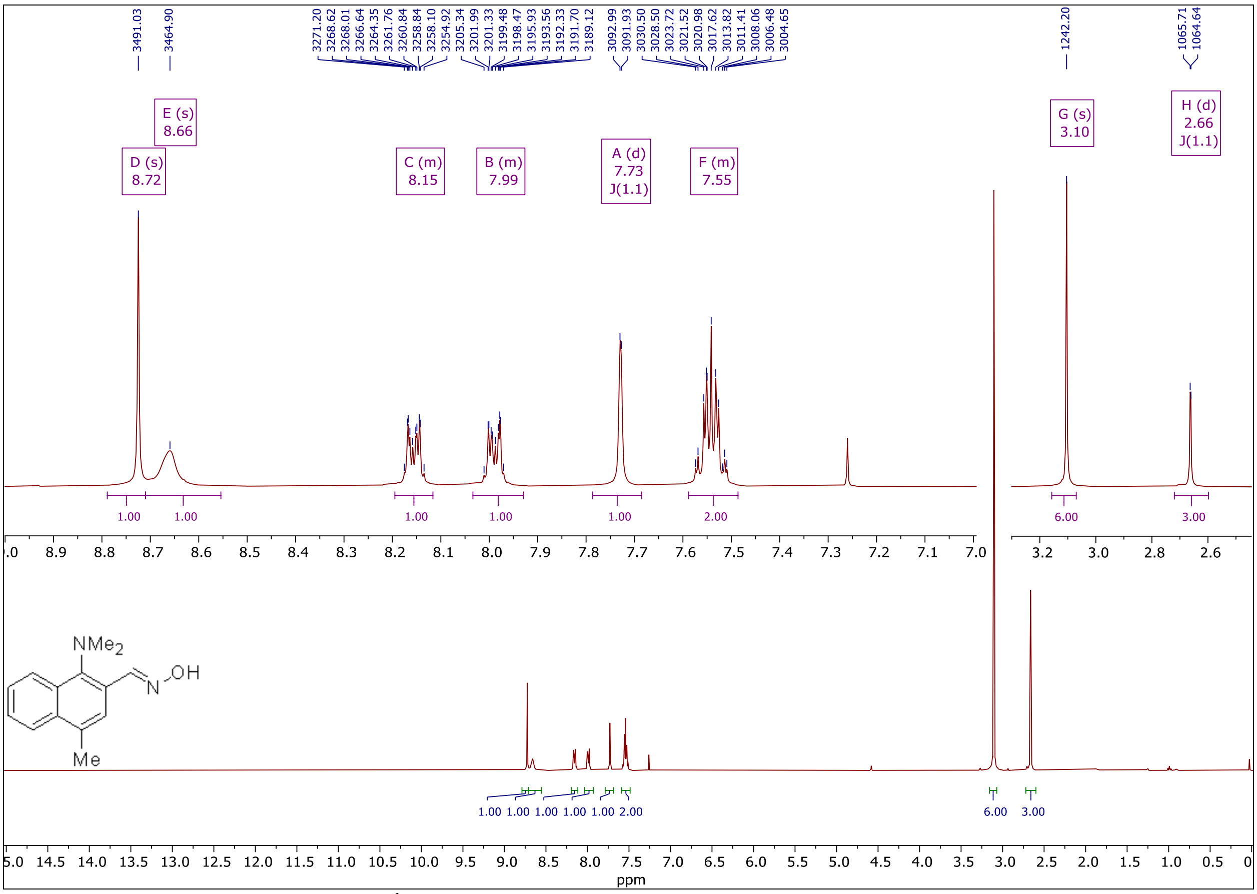

Fig. S23. ^{1}H NMR spectrum of compound **4a** (400 MHz, $CDCl_3$).

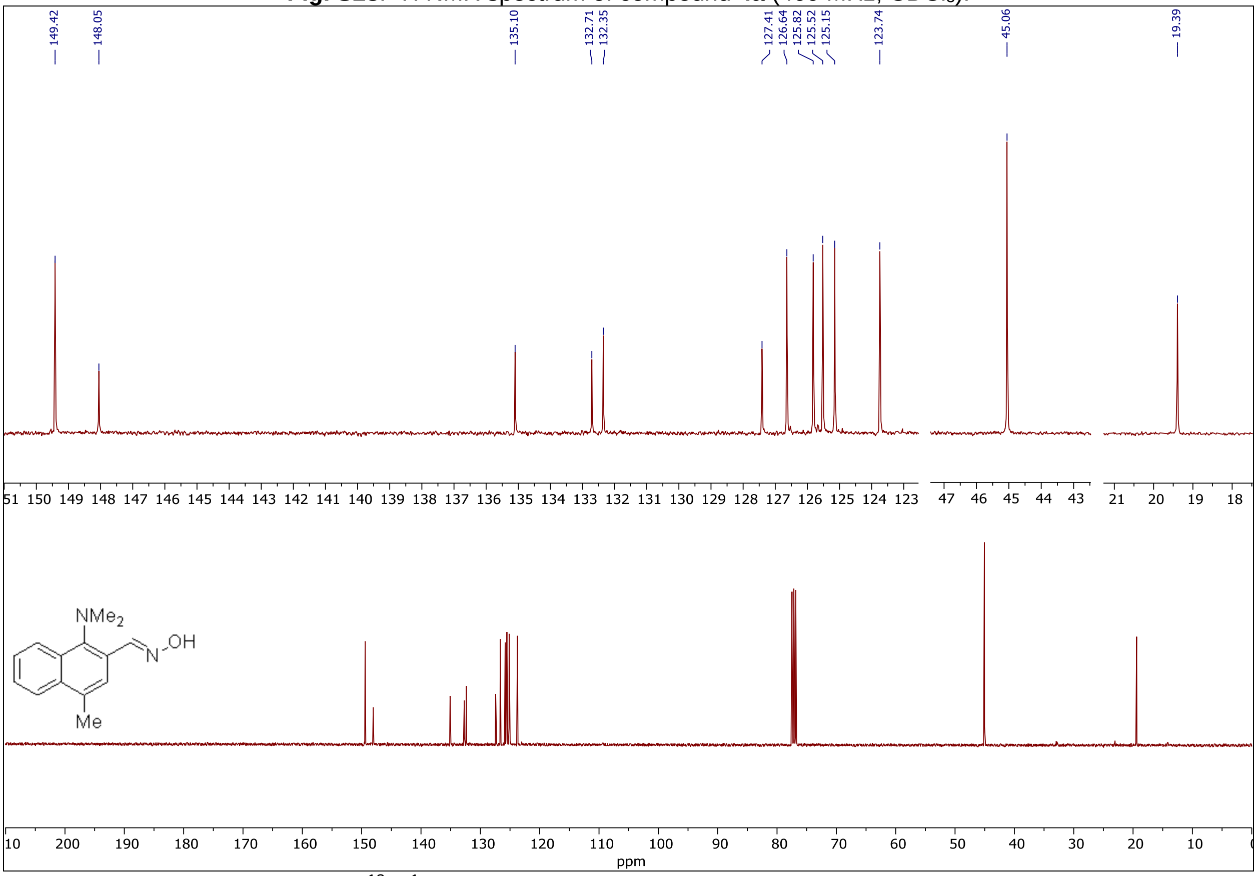

Fig. S24. $^{13}C\{^1H\}$ NMR spectrum of compound **4a** (100 MHz, $CDCl_3$).

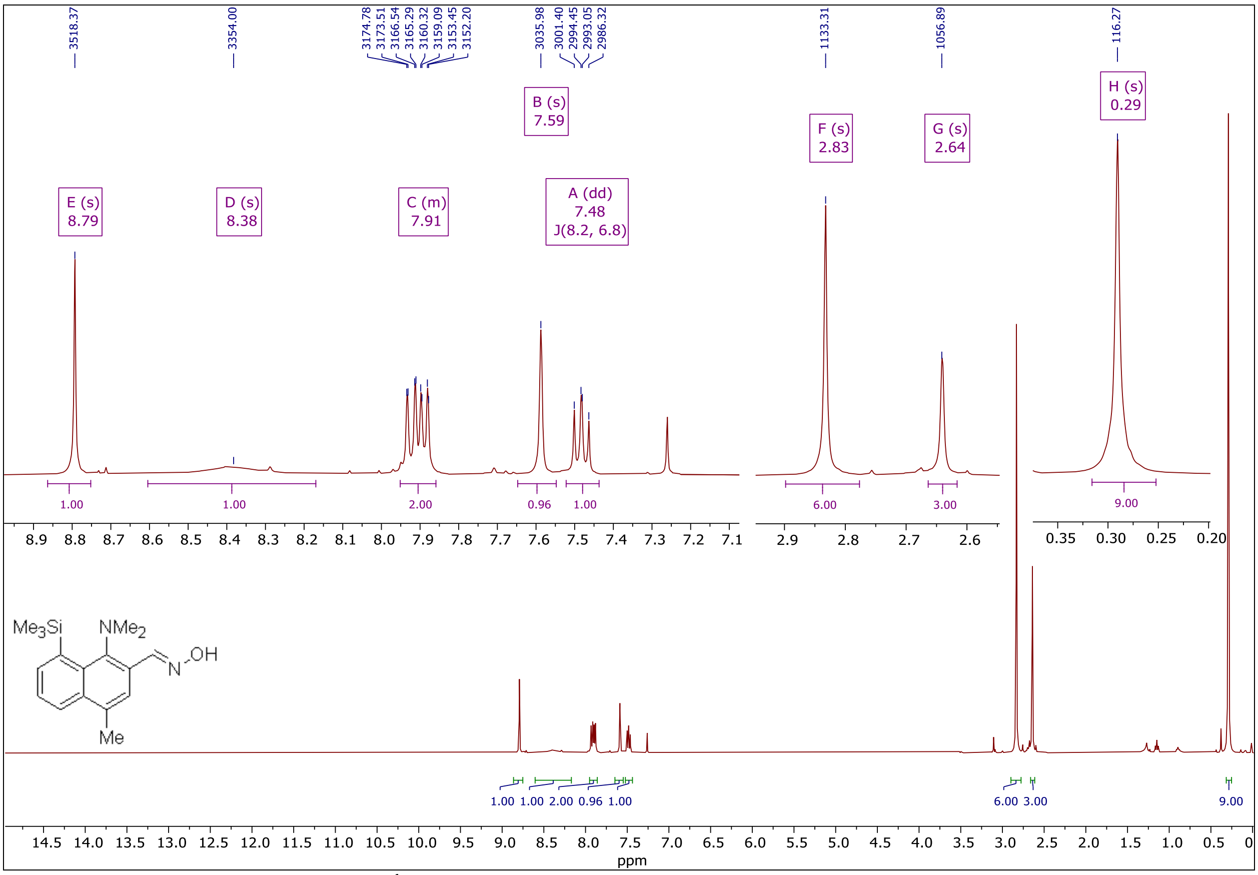

Fig. S25. ^{1}H NMR spectrum of compound **4b** (400 MHz, $CDCl_3$).

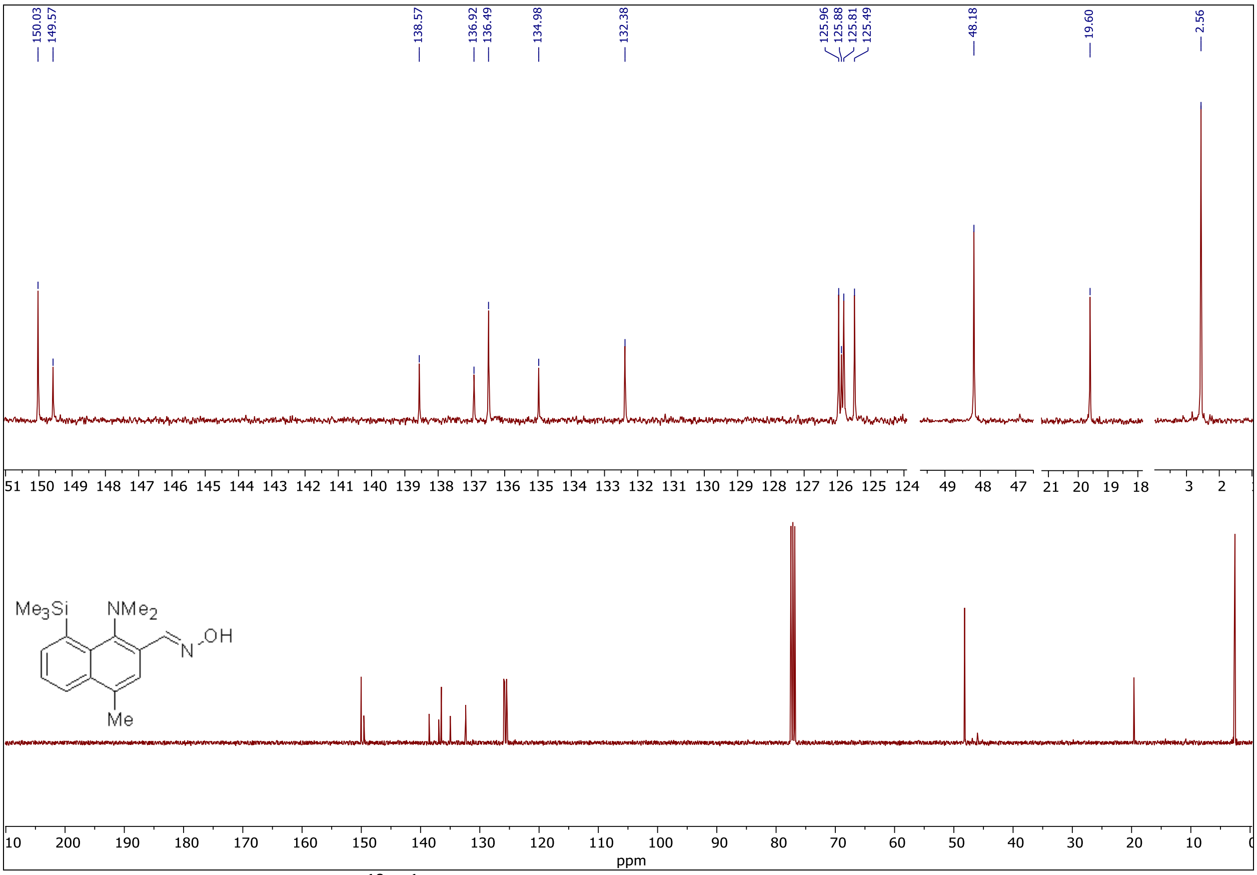

Fig. S26. $^{13}C\{^{1}H\}$ NMR spectrum of compound **4b** (100 MHz, $CDCl_3$).

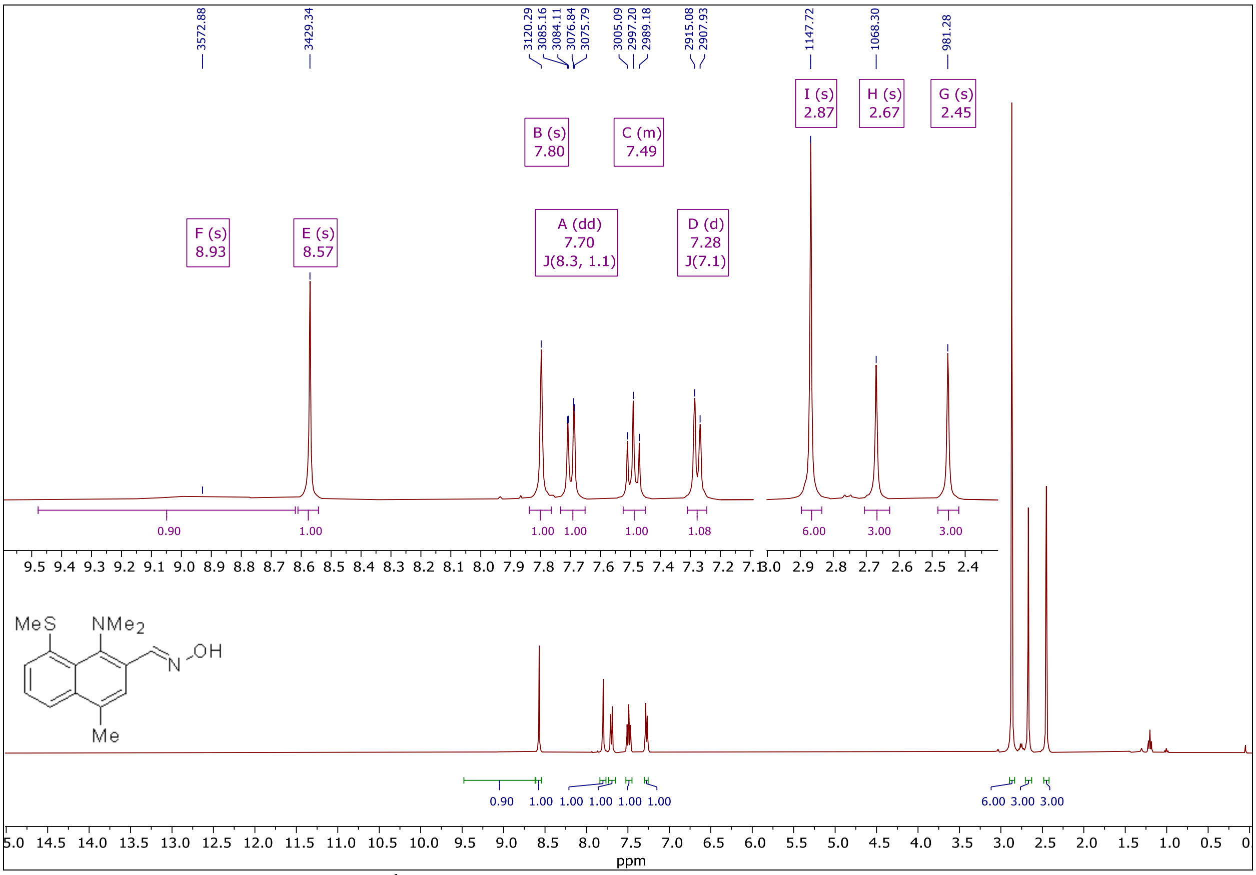

Fig. S27. ^{1}H NMR spectrum of compound **4d** (400 MHz, $CDCl_3$).

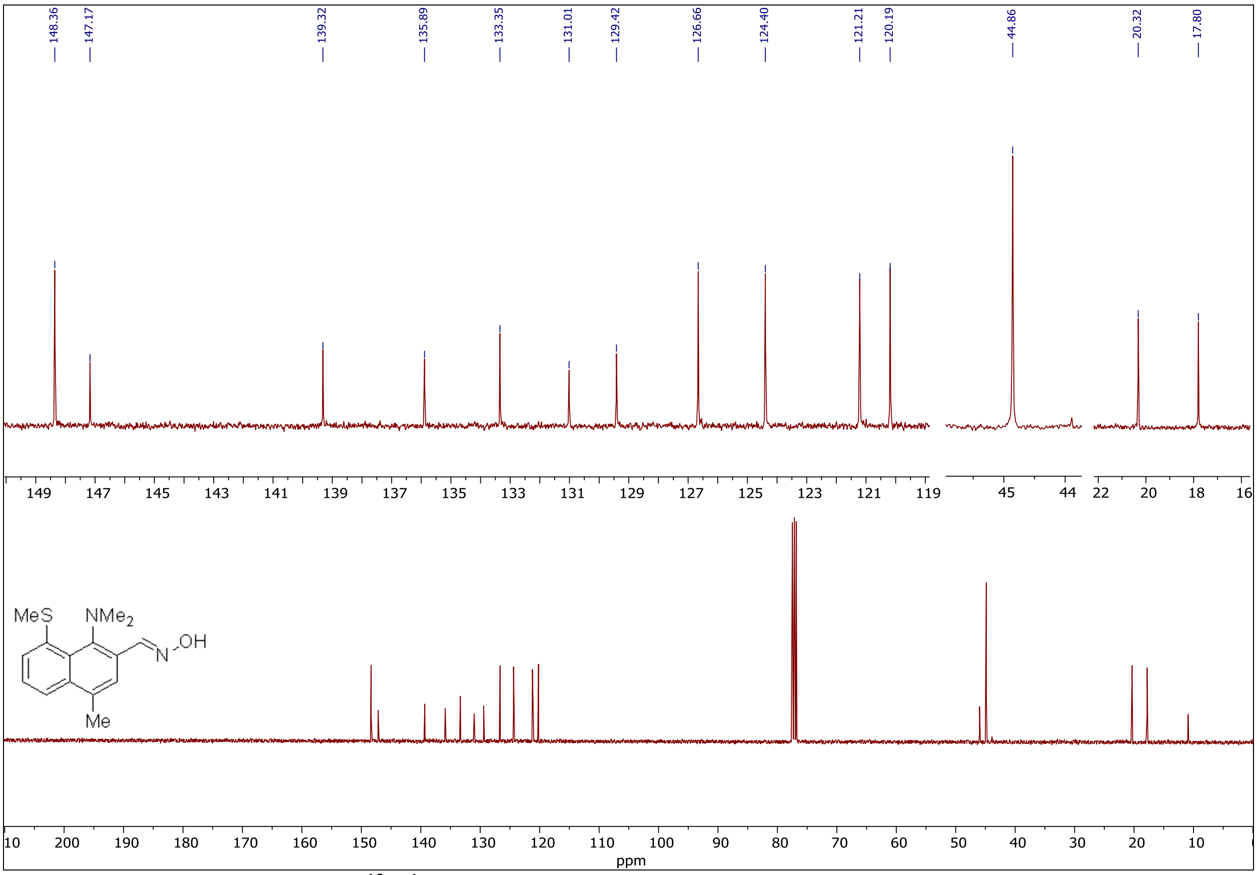

Fig. S28. $^{13}C\{^{1}H\}$ NMR spectrum of compound **4d** (100 MHz, $CDCl_3$).

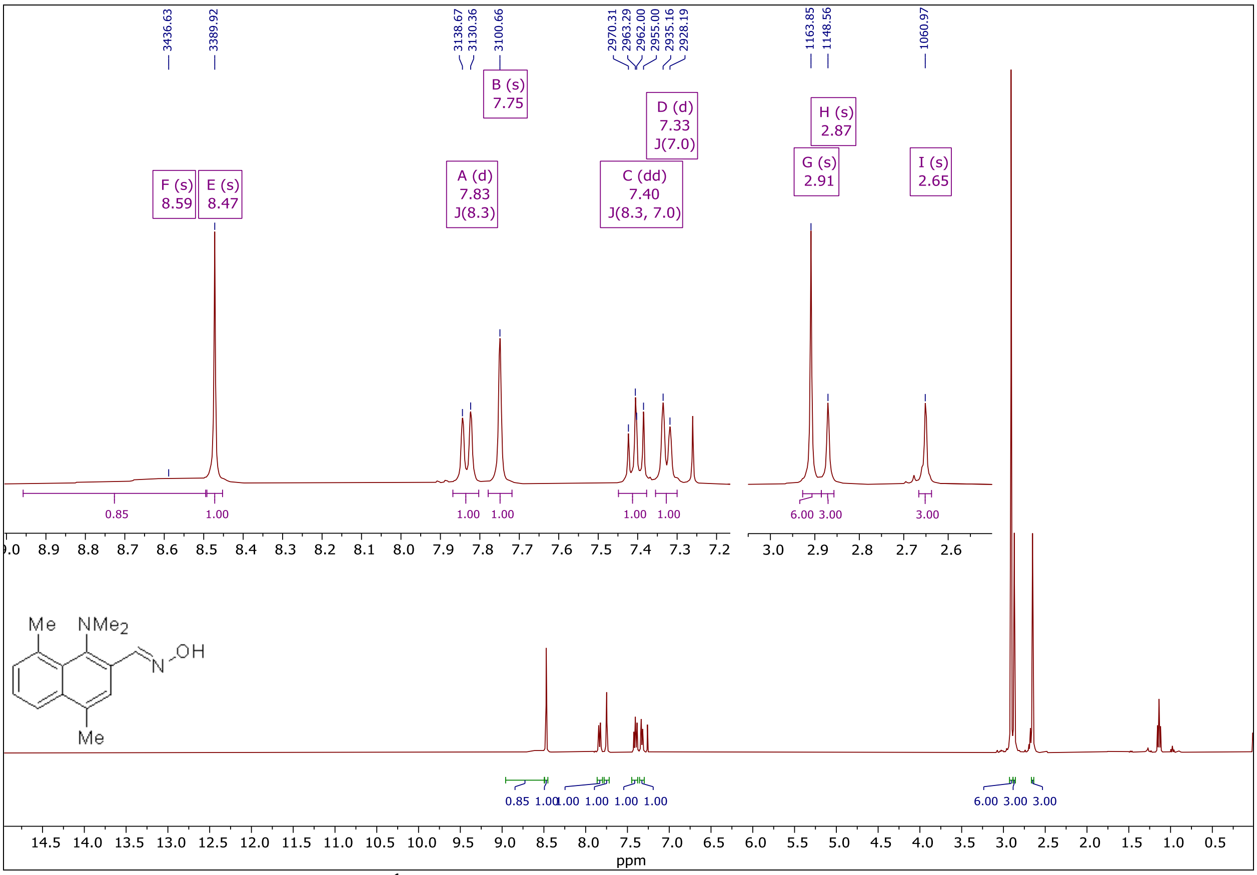

Fig. S29. ^{1}H NMR spectrum of compound **4e** (400 MHz, $CDCl_3$).

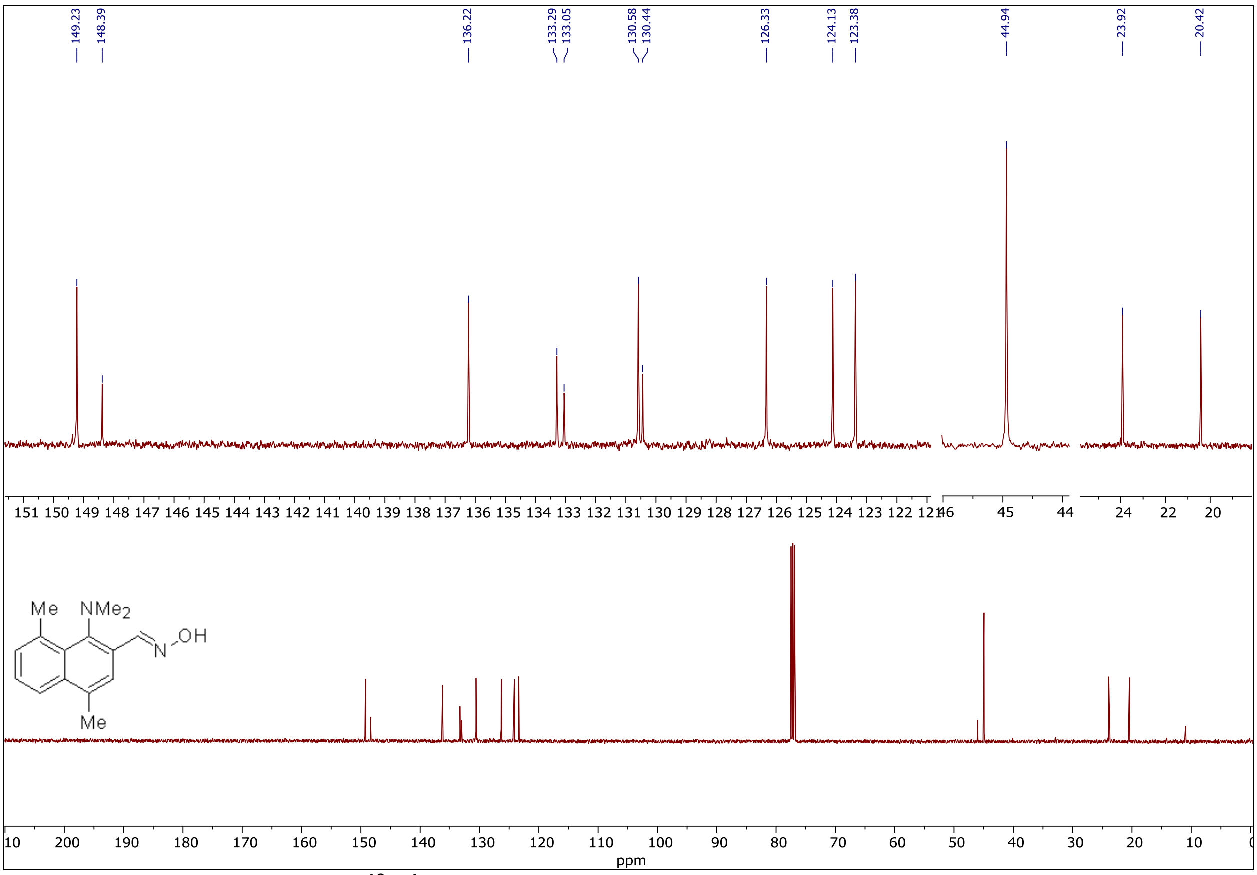

Fig. S30. $^{13}C\{^1H\}$ NMR spectrum of compound **4e** (100 MHz, $CDCl_3$).

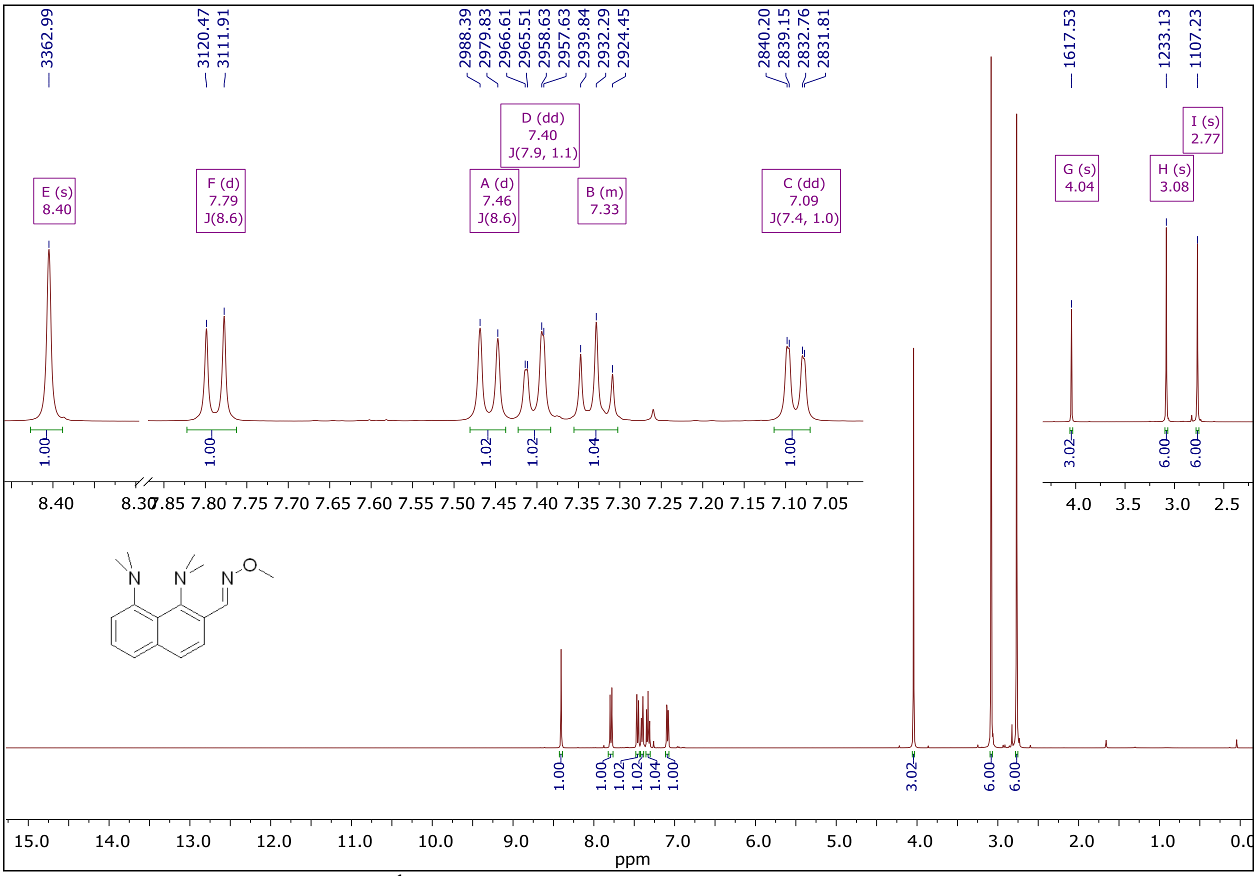

Fig. S31. ^{1}H NMR spectrum of compound **4f** (400 MHz, $CDCl_3$).

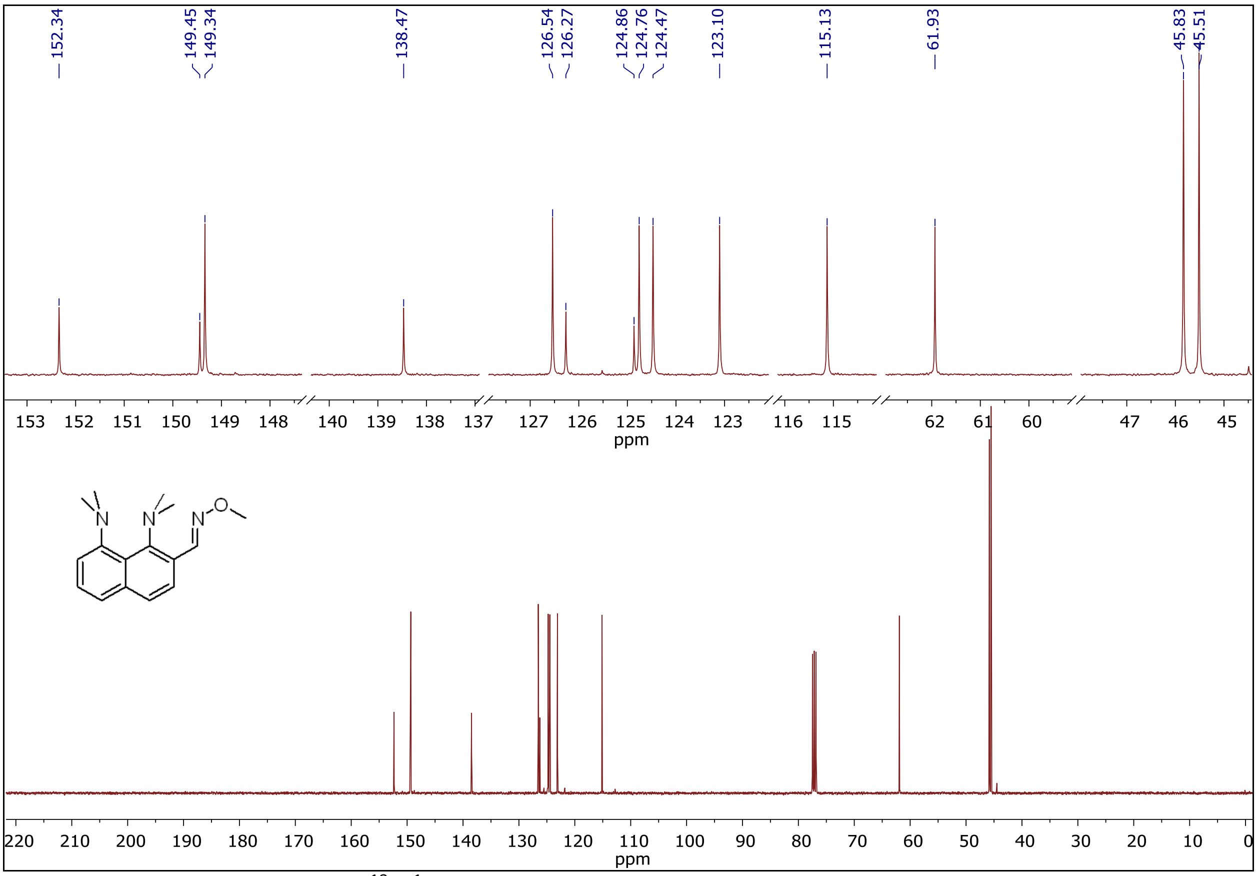

Fig. S32. $^{13}C\{^1H\}$ NMR spectrum of compound **4f** (100 MHz, $CDCl_3$).

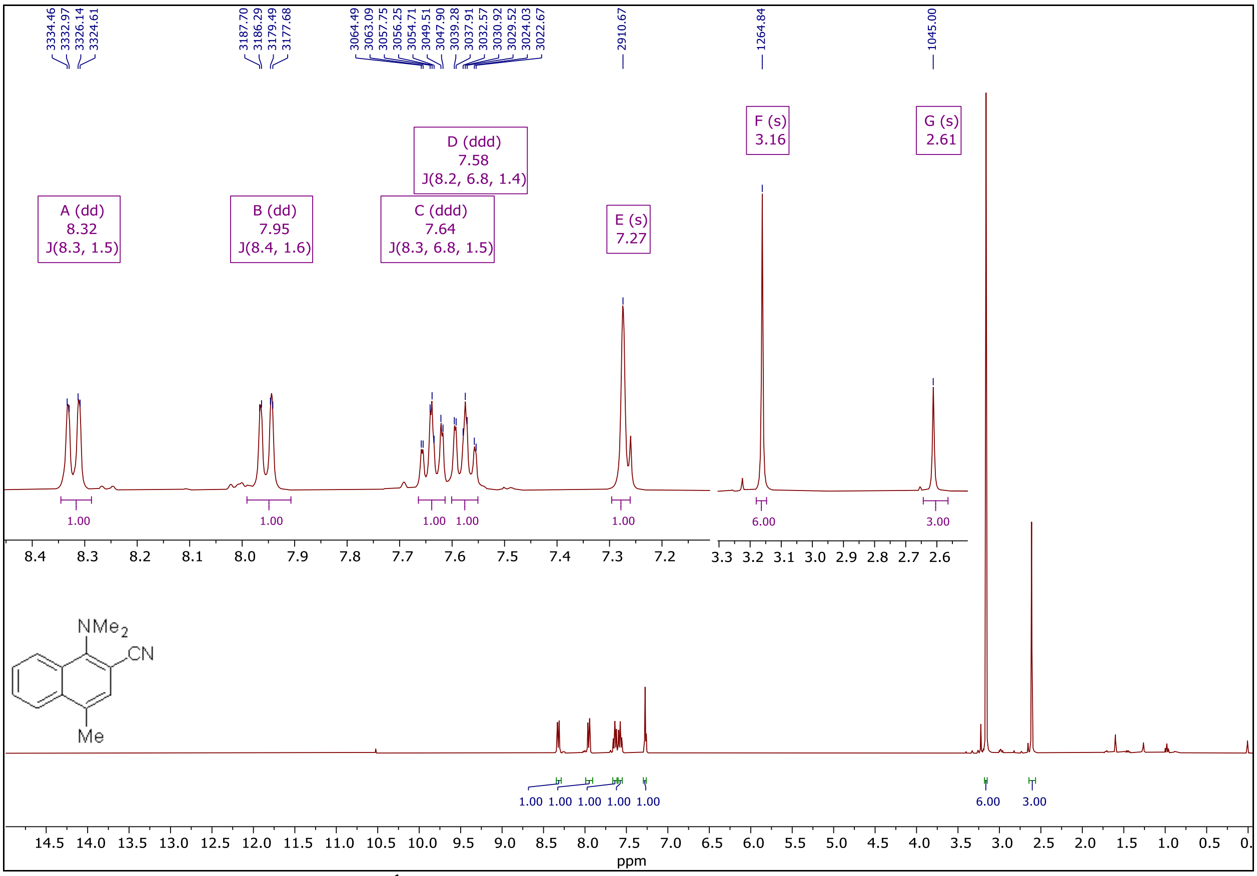

Fig. S33. ^{1}H NMR spectrum of compound **5a** (400 MHz, $CDCl_3$).

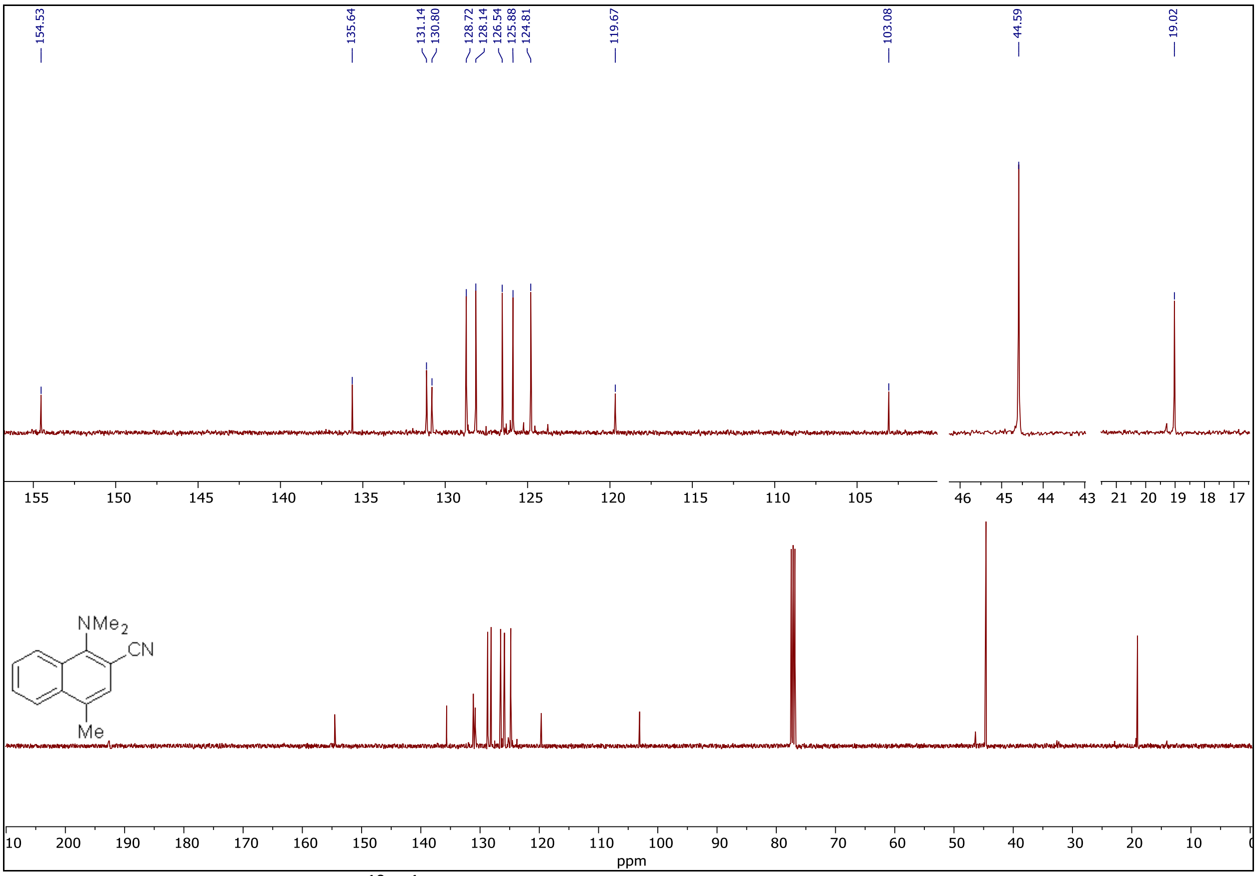

Fig. S34. $^{13}C\{^1H\}$ NMR spectrum of compound **5a** (100 MHz, $CDCl_3$).

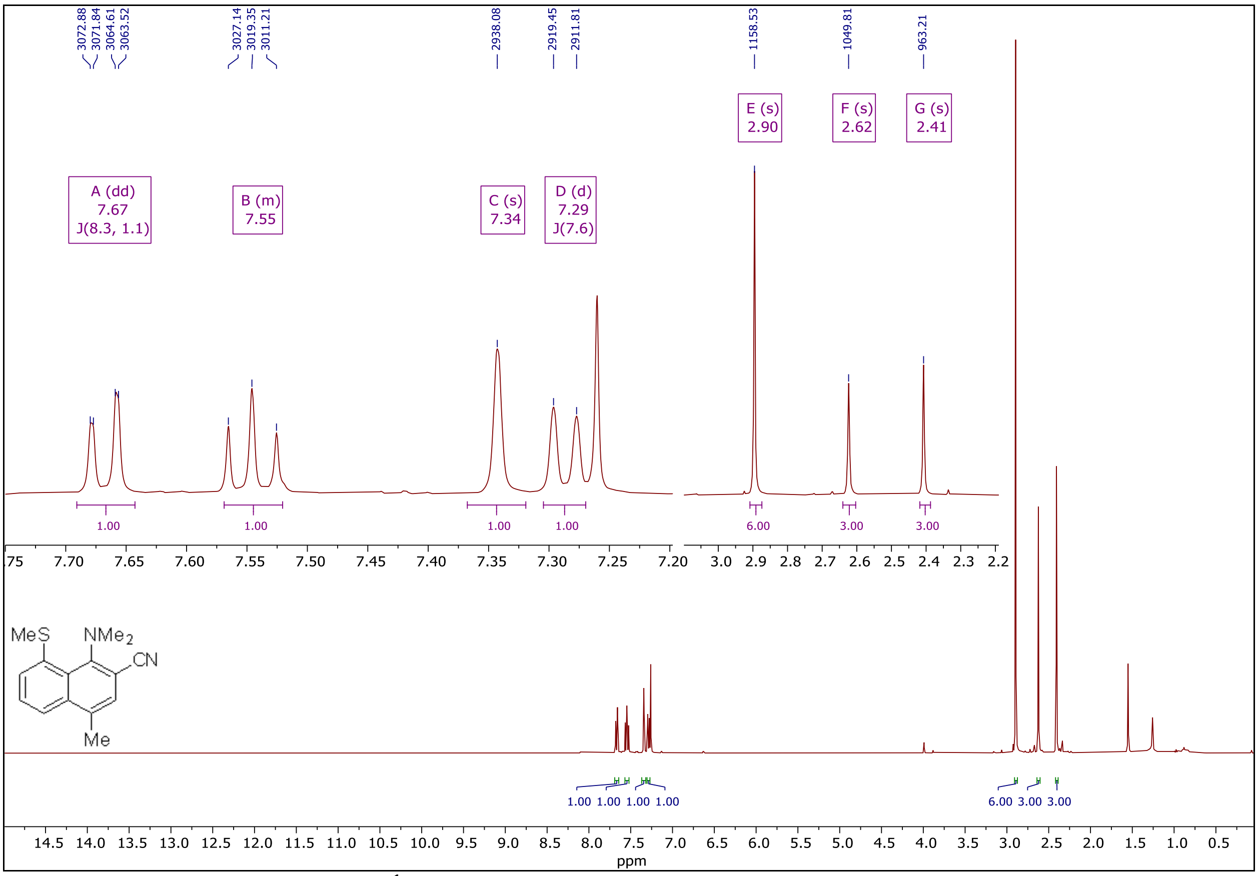

Fig. S35. ^{1}H NMR spectrum of compound **5d** (400 MHz, $CDCl_3$).

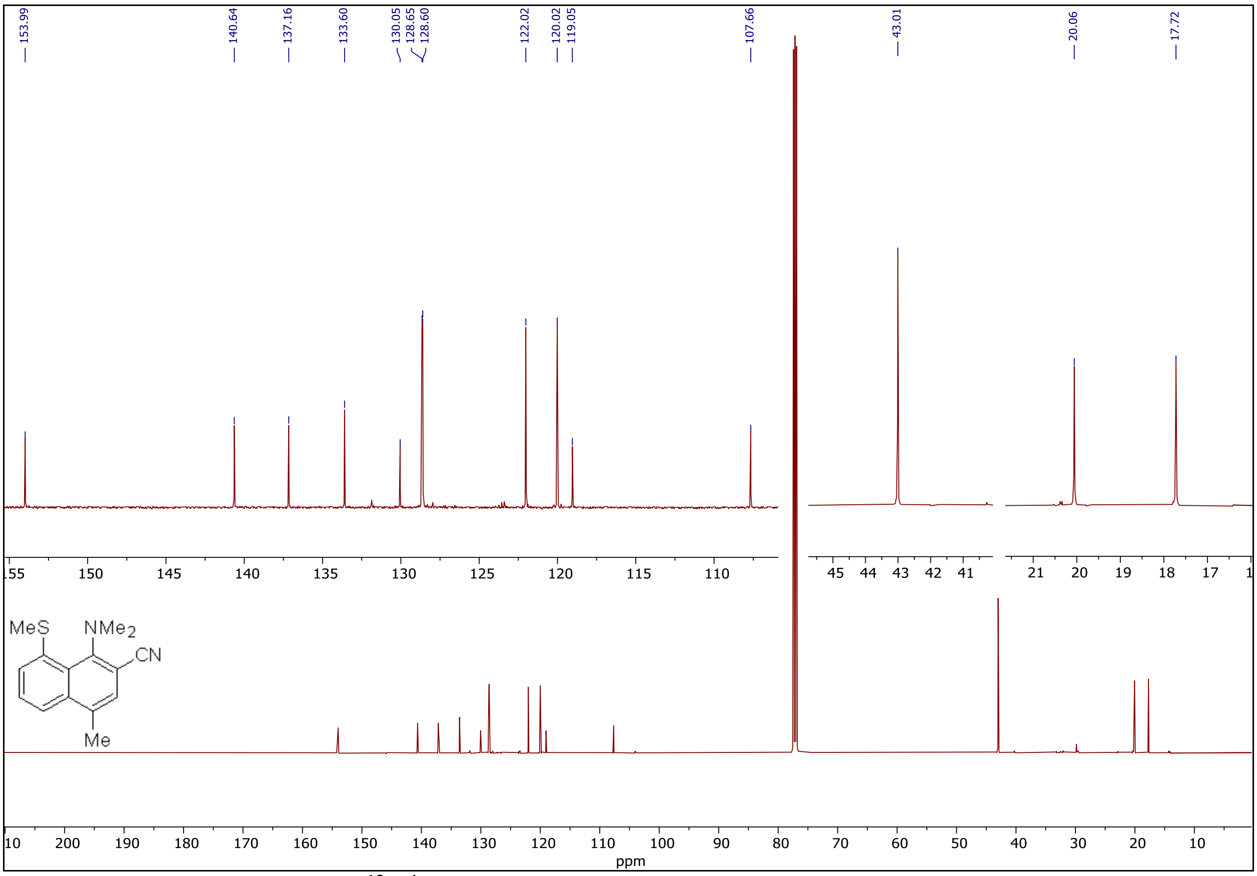

Fig. S36. $^{13}C\{^{1}H\}$ NMR spectrum of compound **5d** (100 MHz, $CDCl_3$).

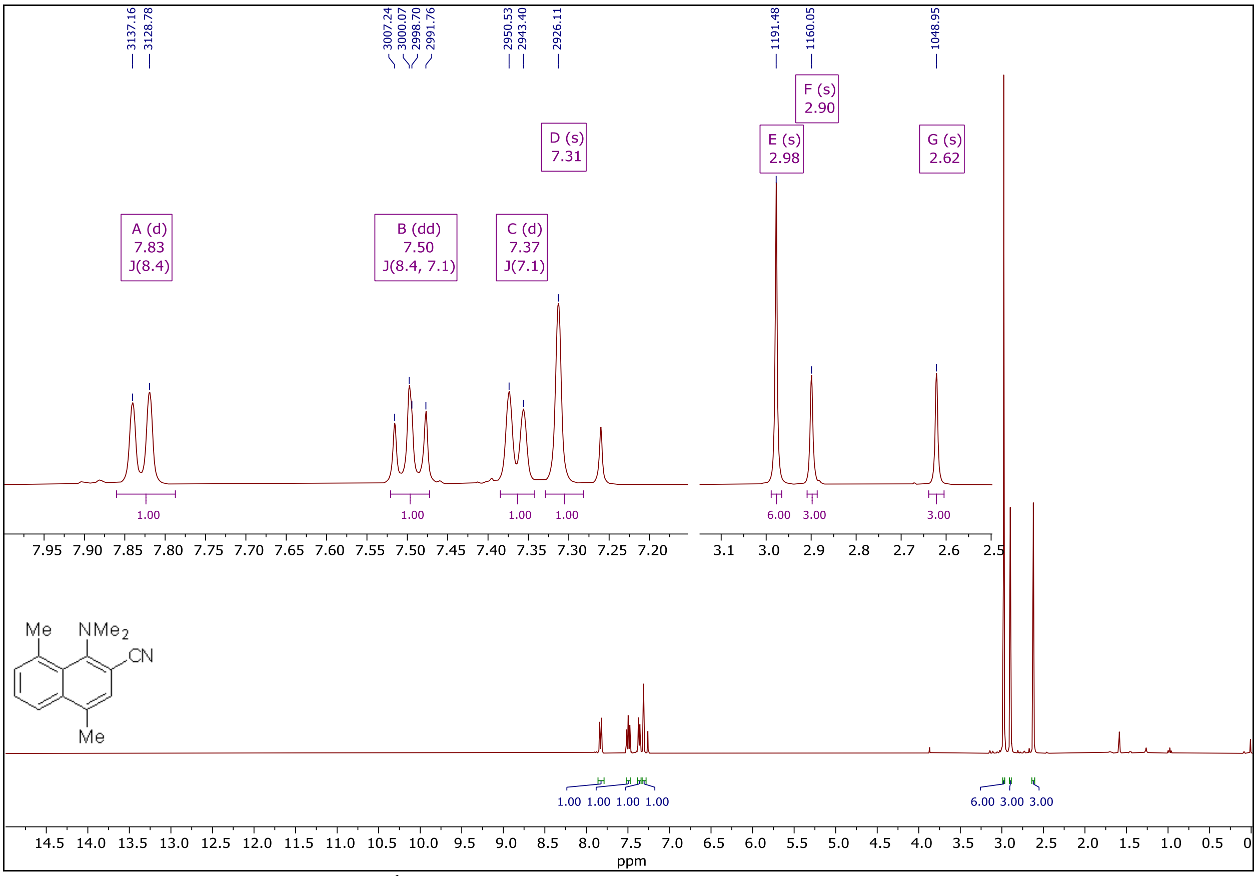

Fig. S37. ^{1}H NMR spectrum of compound **5e** (400 MHz, $CDCl_3$).

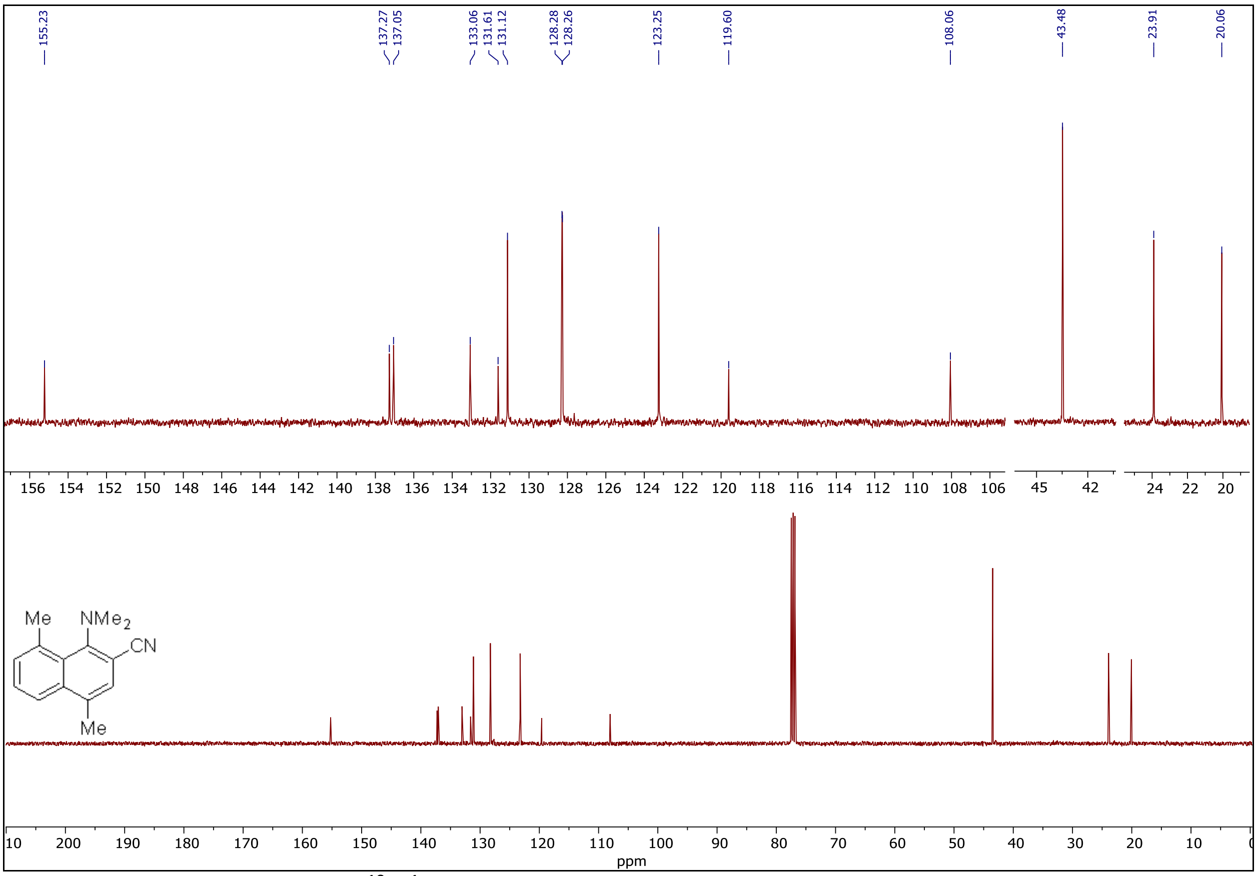

Fig. S38. $^{13}C\{^{1}H\}$ NMR spectrum of compound **5e** (100 MHz, $CDCl_3$).

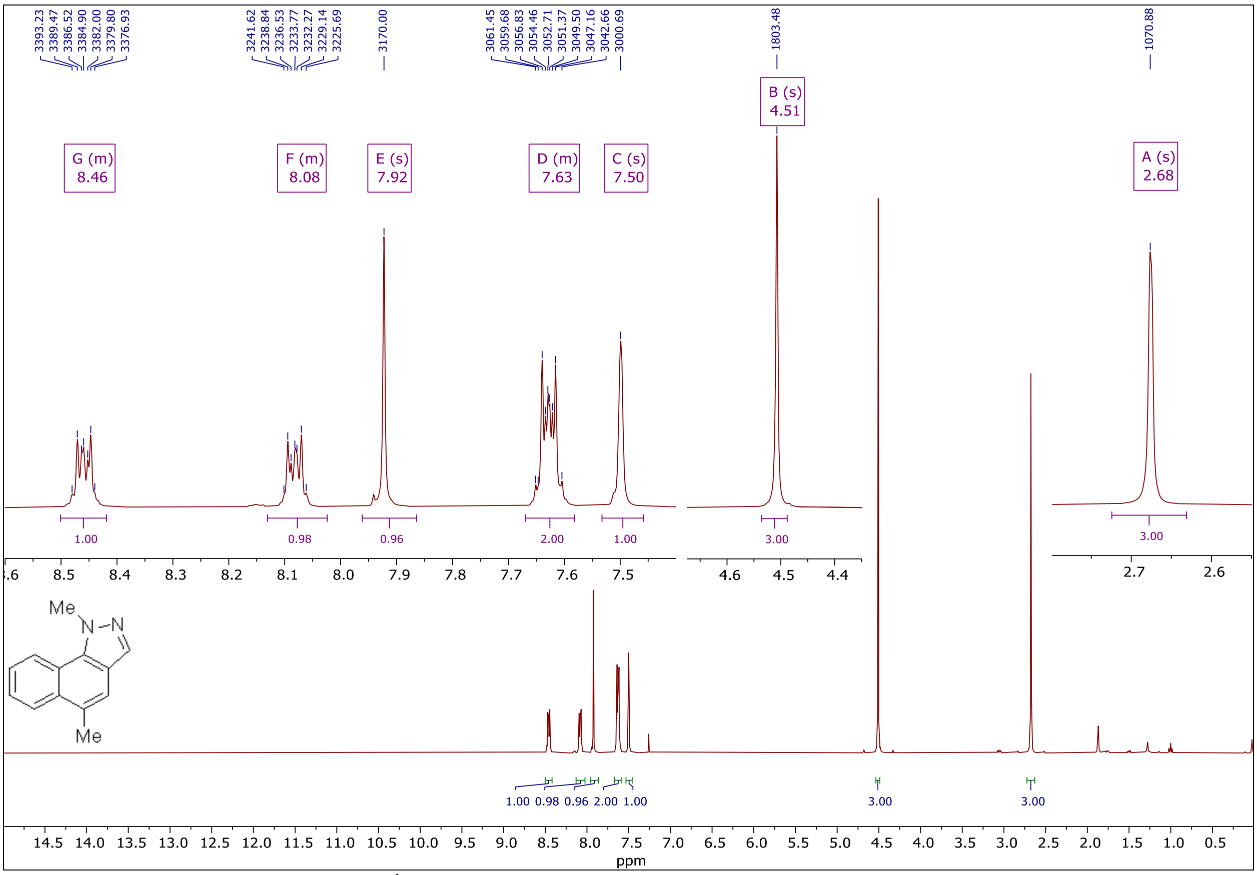

Fig. S39. ^{1}H NMR spectrum of compound **6a** (400 MHz, $CDCl_3$).

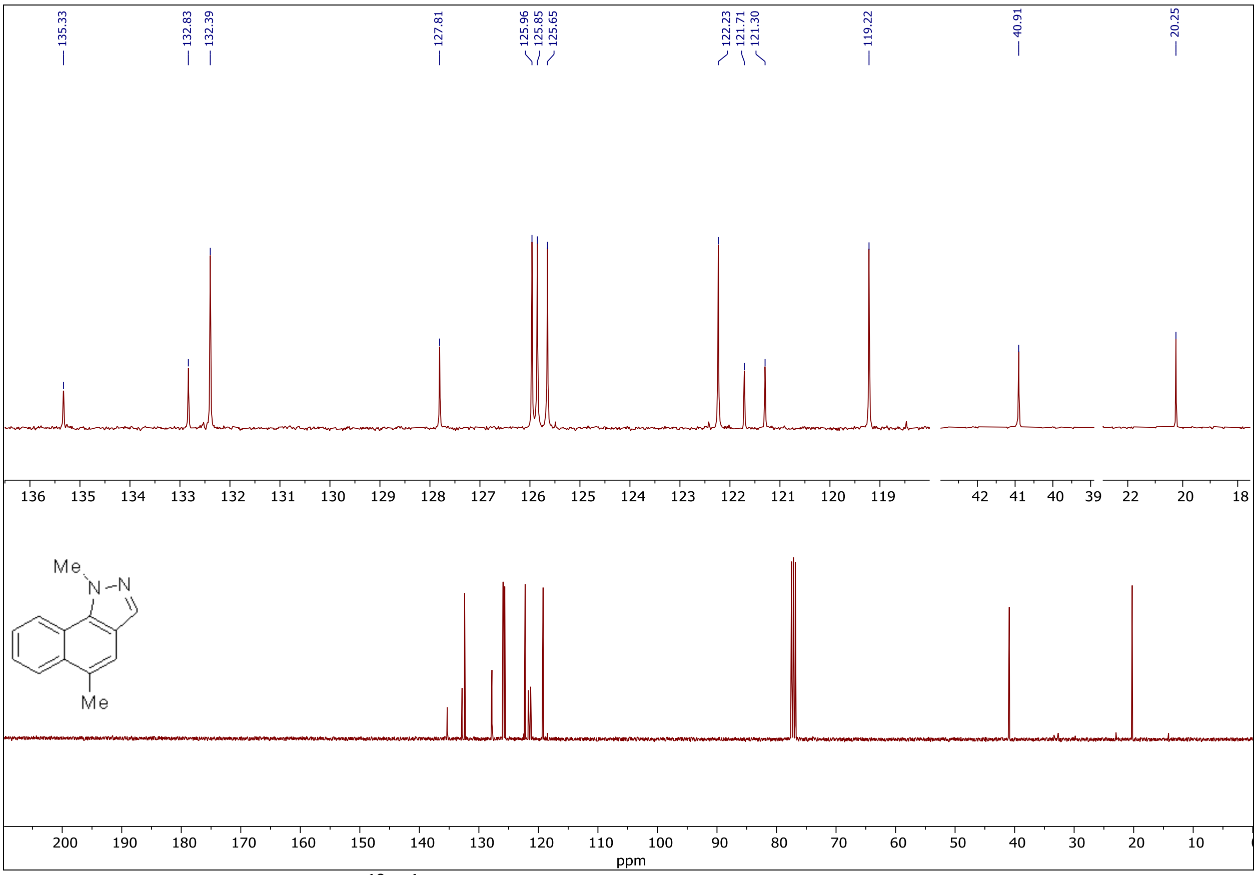

Fig. S40. $^{13}C\{^1H\}$ NMR spectrum of compound **6a** (100 MHz, $CDCl_3$).

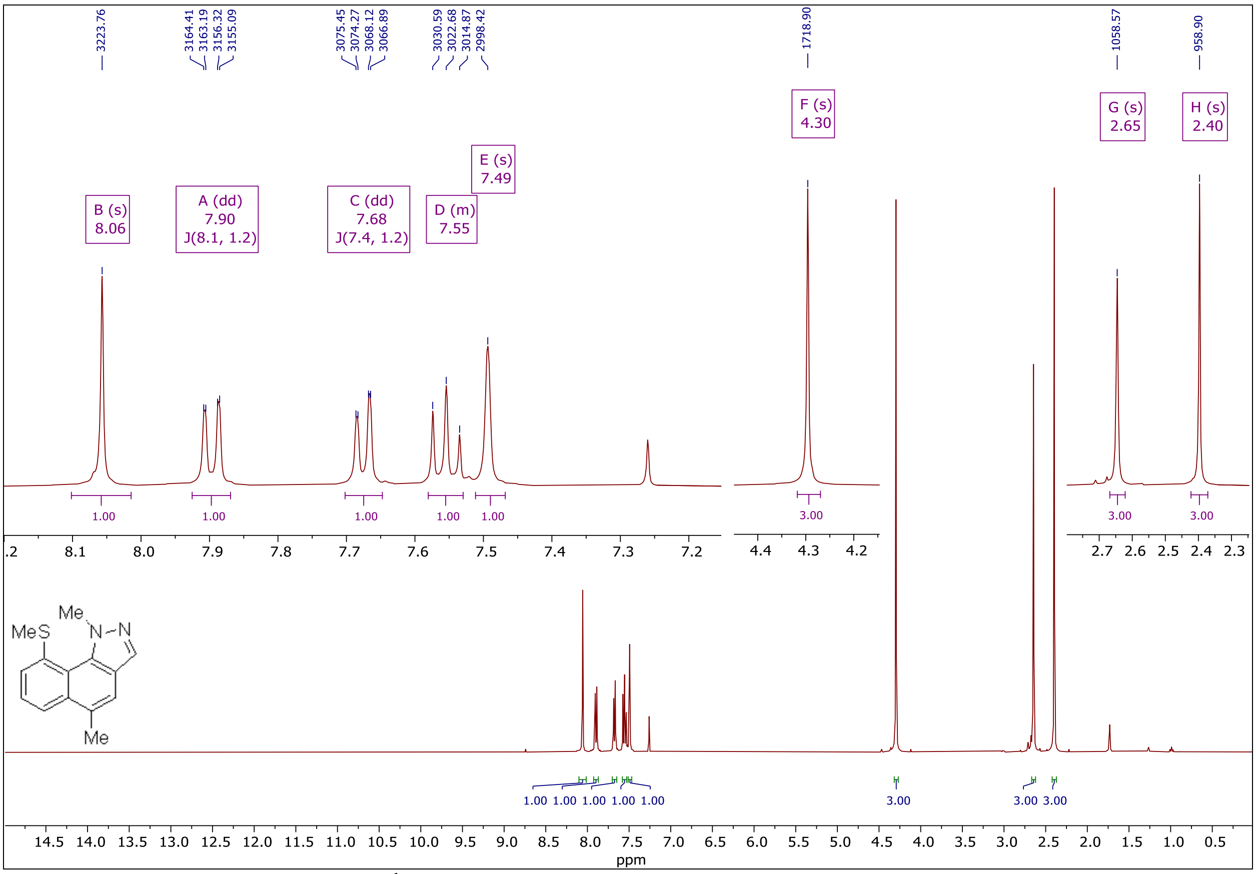

Fig. S41. ^{1}H NMR spectrum of compound **6d** (400 MHz, $CDCl_3$).

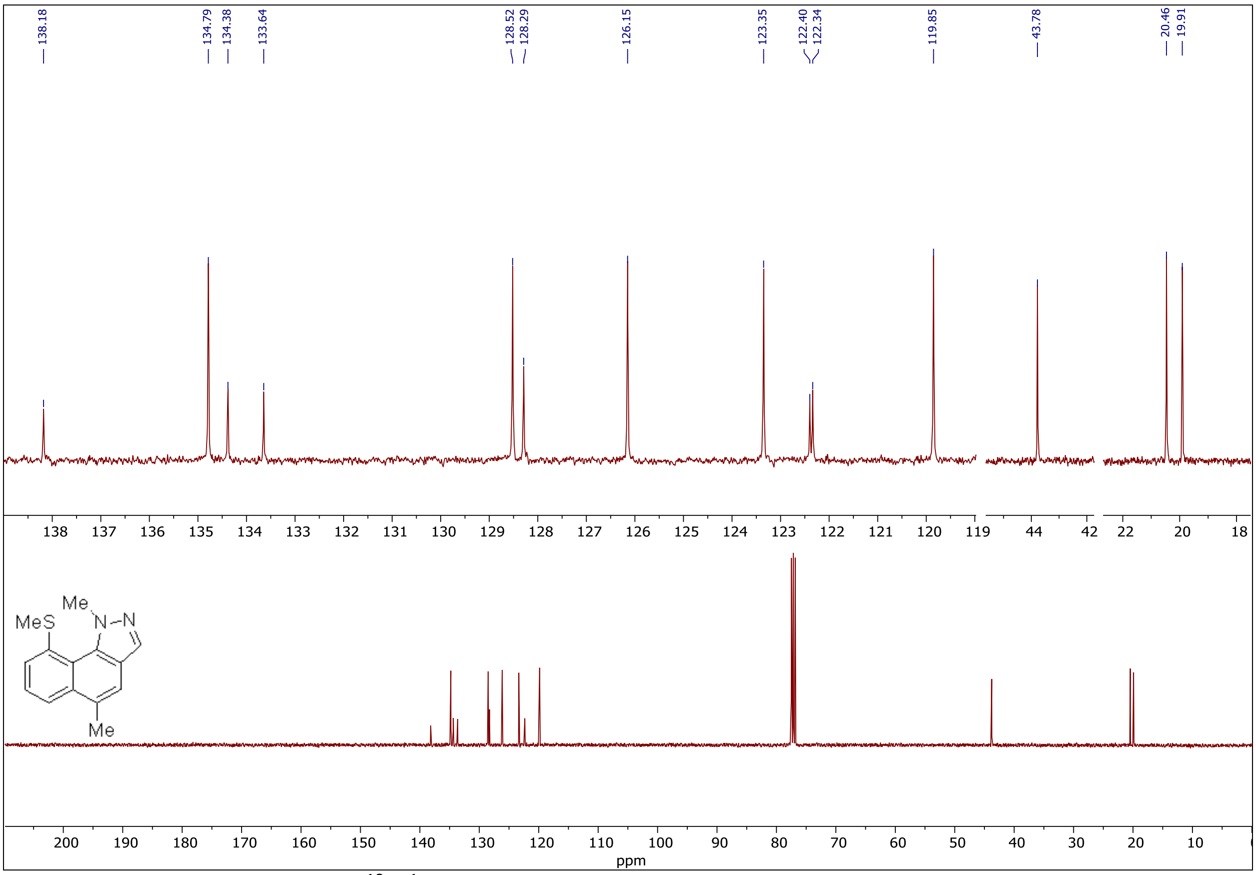

Fig. S42. $^{13}C\{^{1}H\}$ NMR spectrum of compound **6d** (100 MHz, $CDCl_3$).

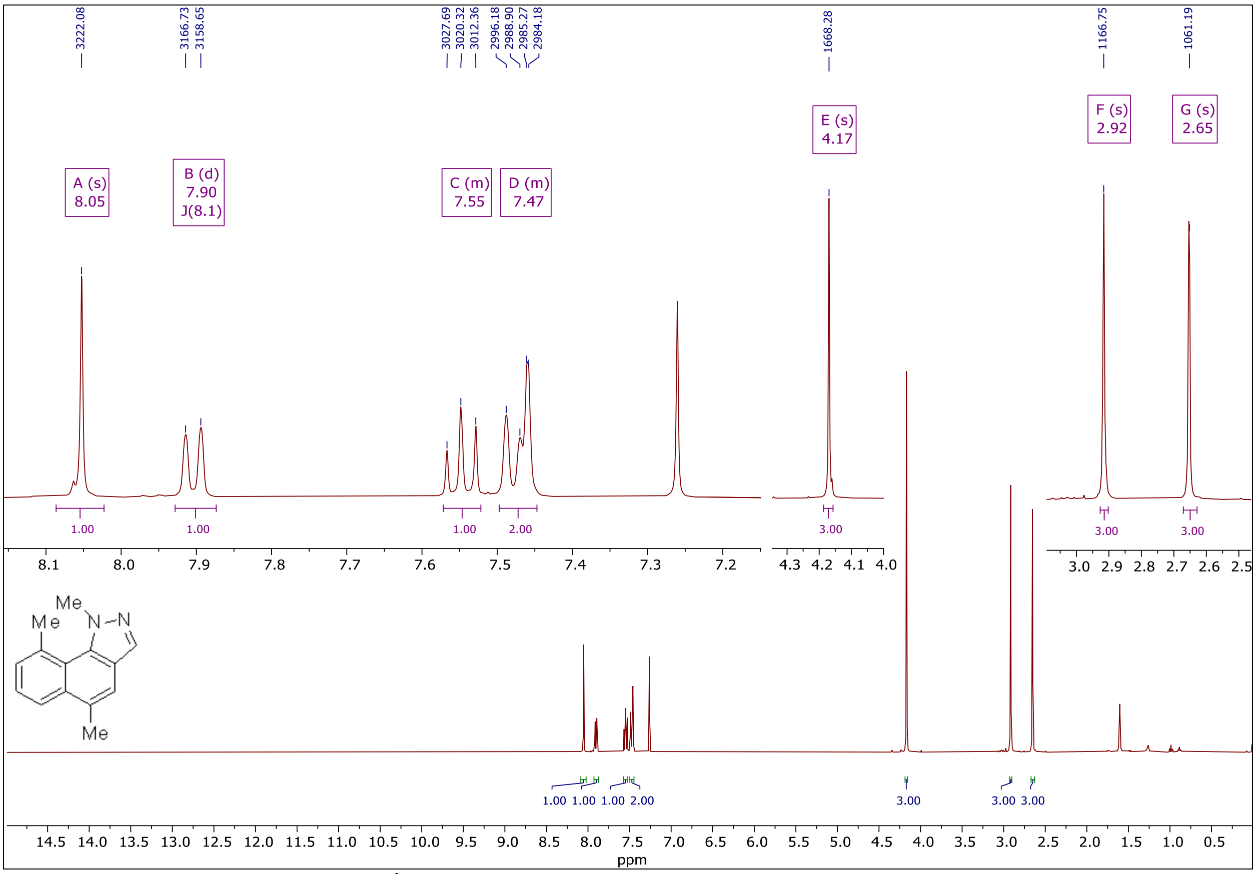

Fig. S43. ^{1}H NMR spectrum of compound **6e** (400 MHz, $CDCl_3$).

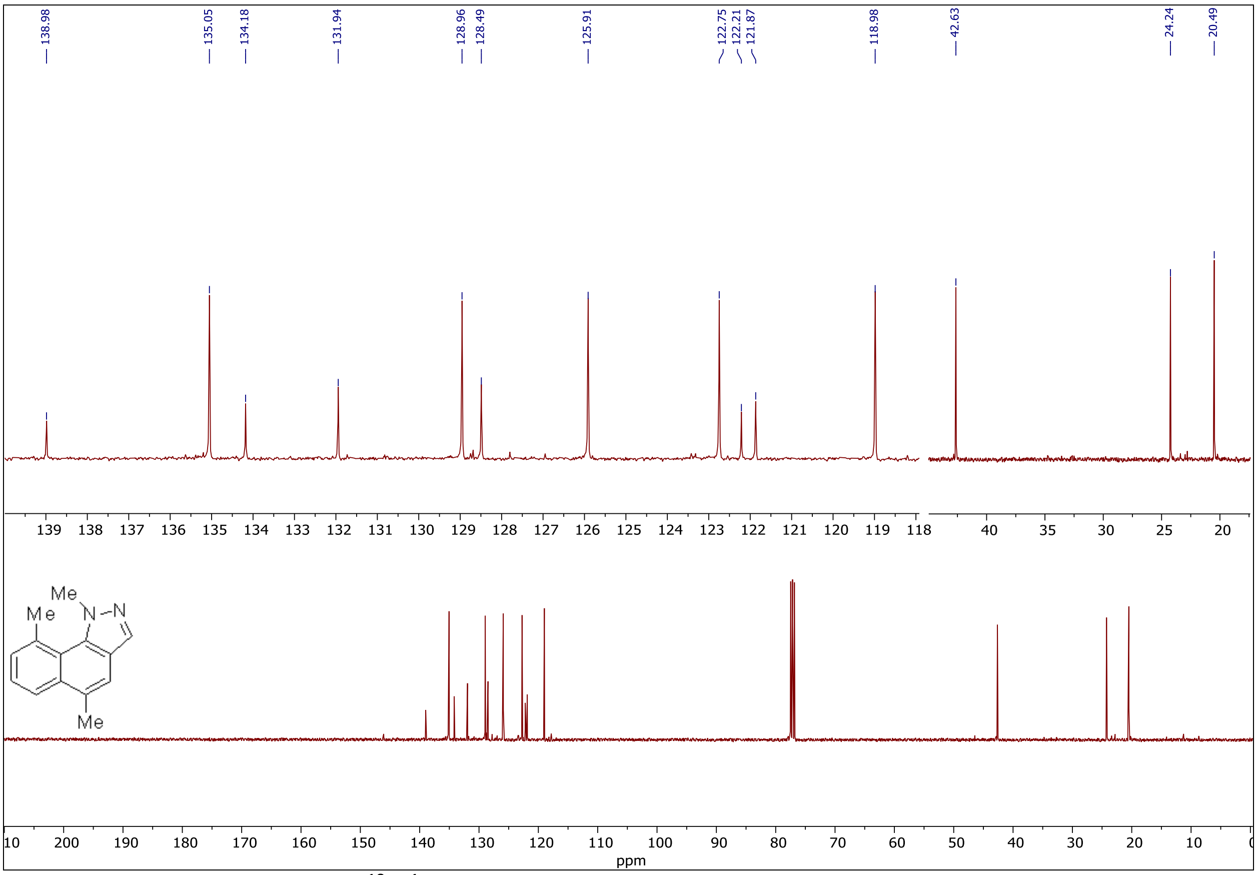

Fig. S44. ^{13}C{^{1}H} NMR spectrum of compound **6e** (100 MHz, $CDCl_3$).

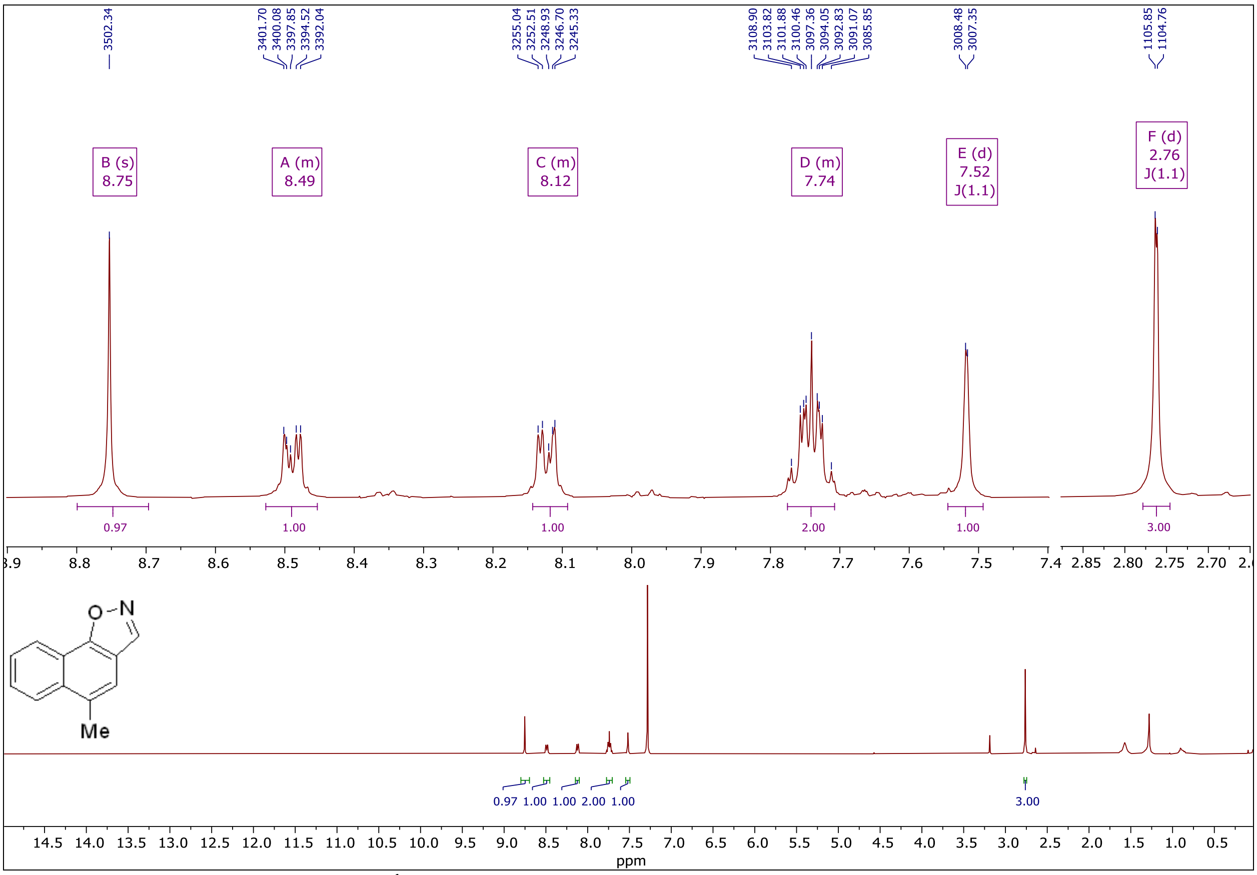

Fig. S45. ^{1}H NMR spectrum of compound **7a** (400 MHz, $CDCl_3$).

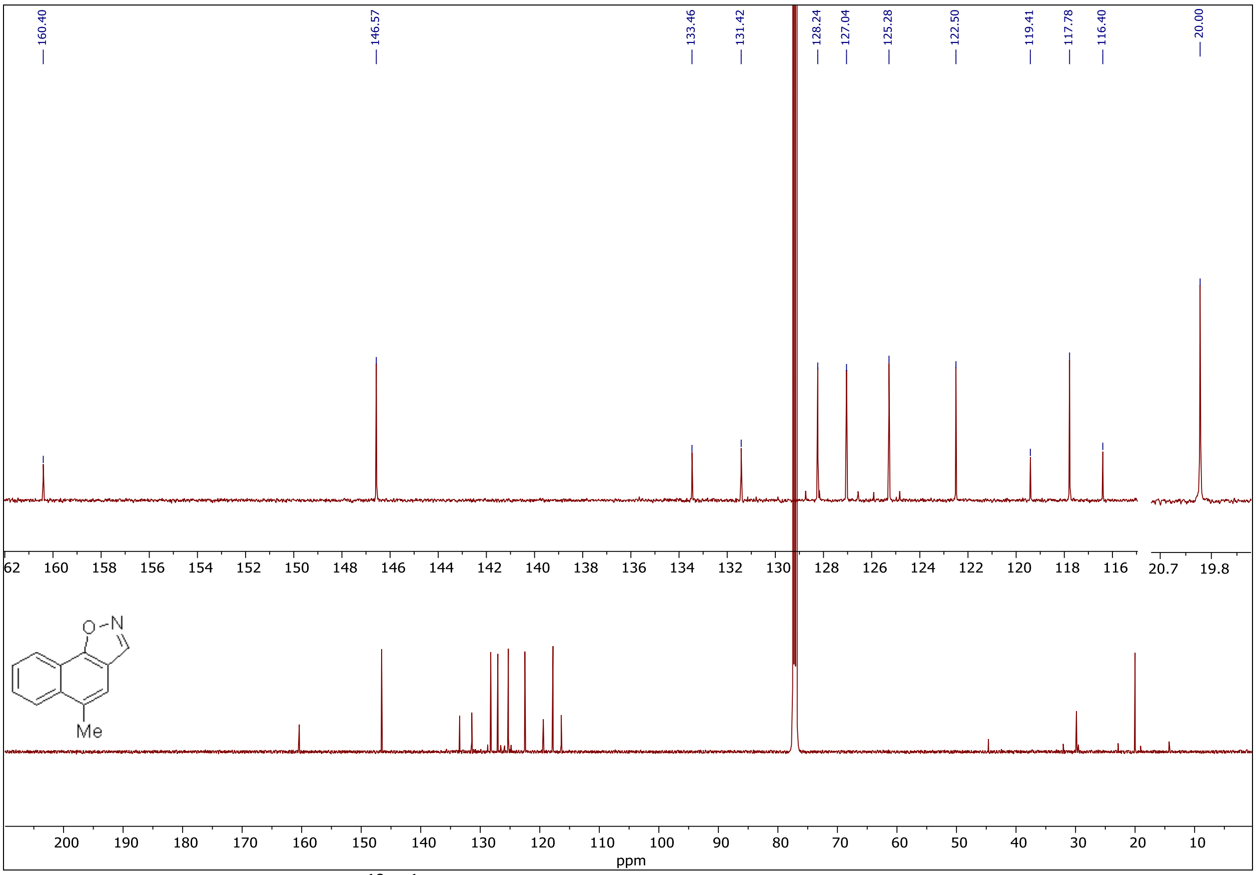

Fig. S46. $^{13}C\{^1H\}$ NMR spectrum of compound **7a** (100 MHz, $CDCl_3$).

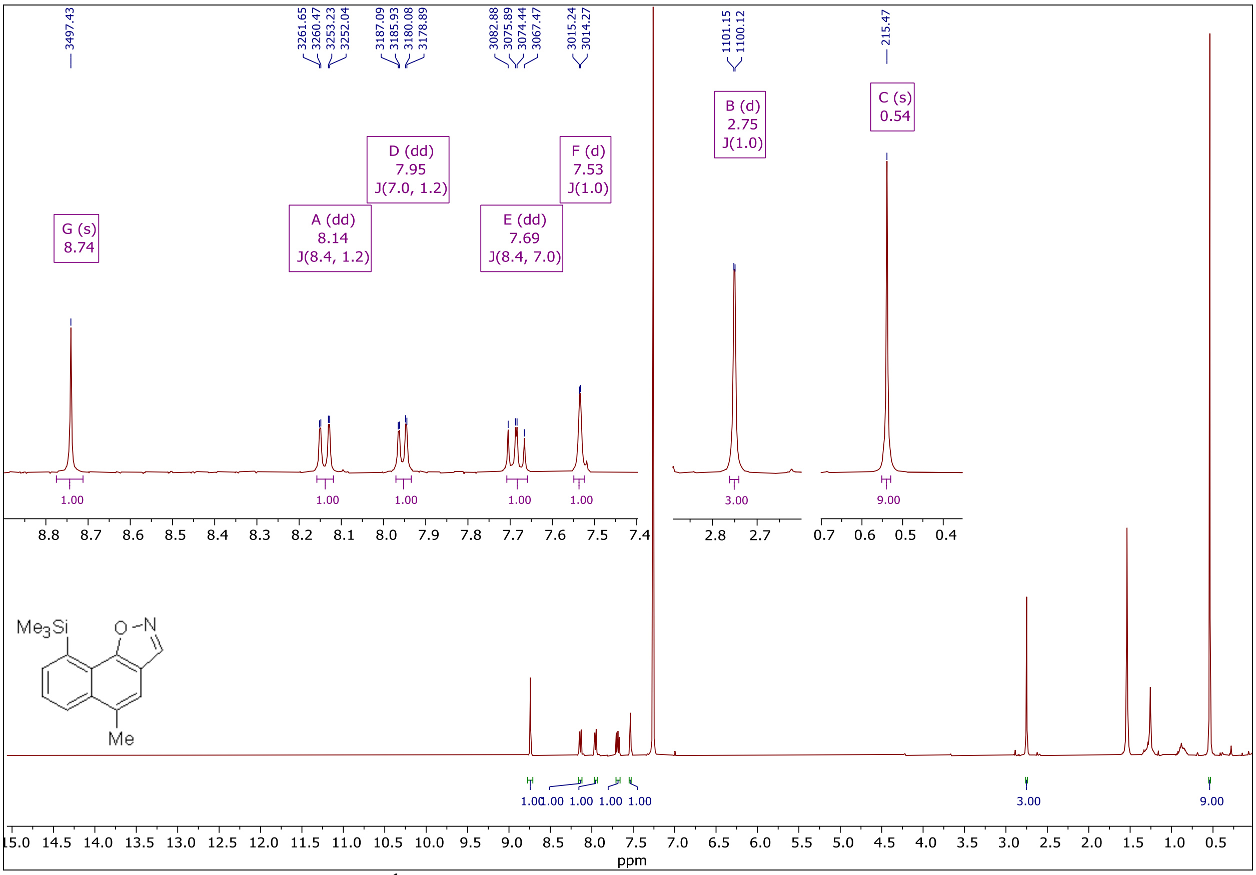

Fig. S47. ^{1}H NMR spectrum of compound **7b** (400 MHz, $CDCl_3$).

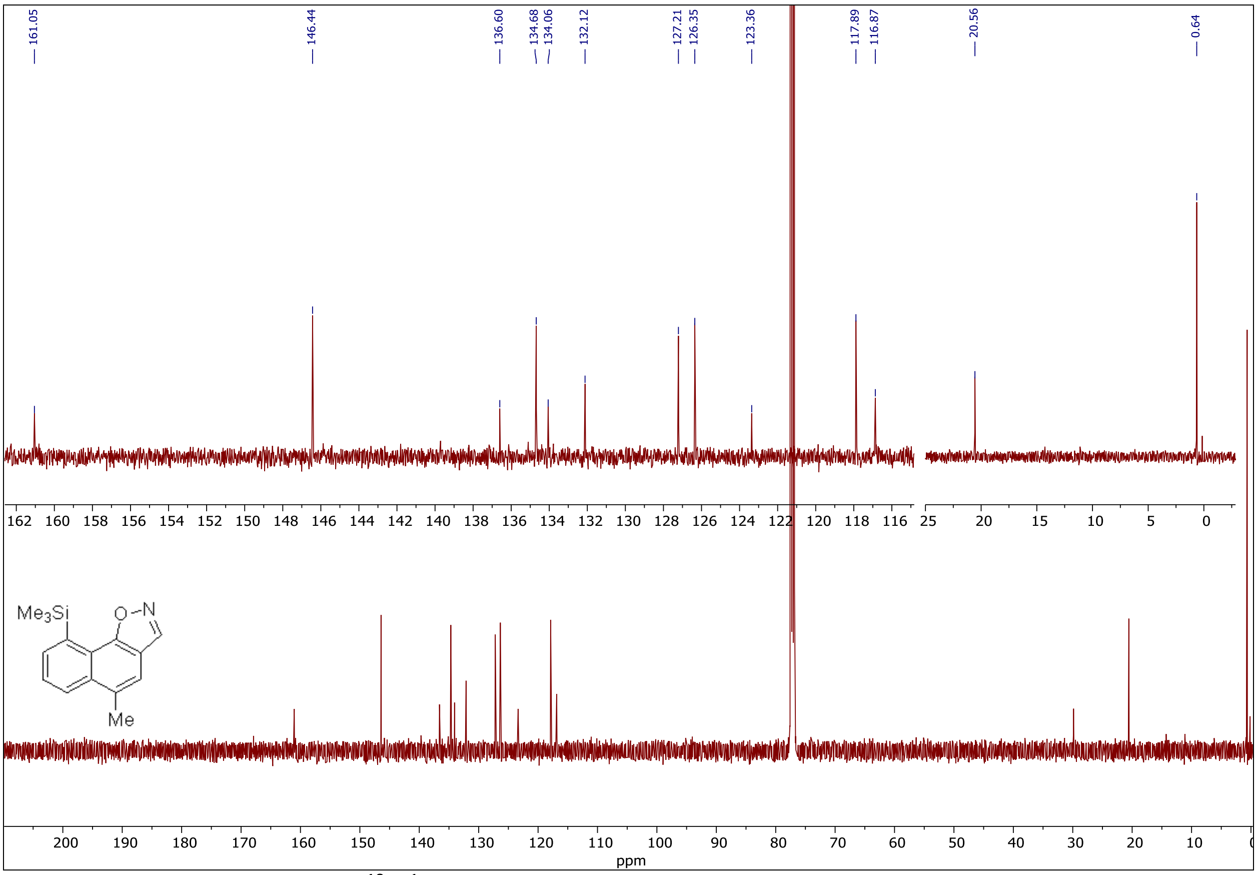

Fig. S48. $^{13}C\{^{1}H\}$ NMR spectrum of compound **7b** (100 MHz, $CDCl_3$).

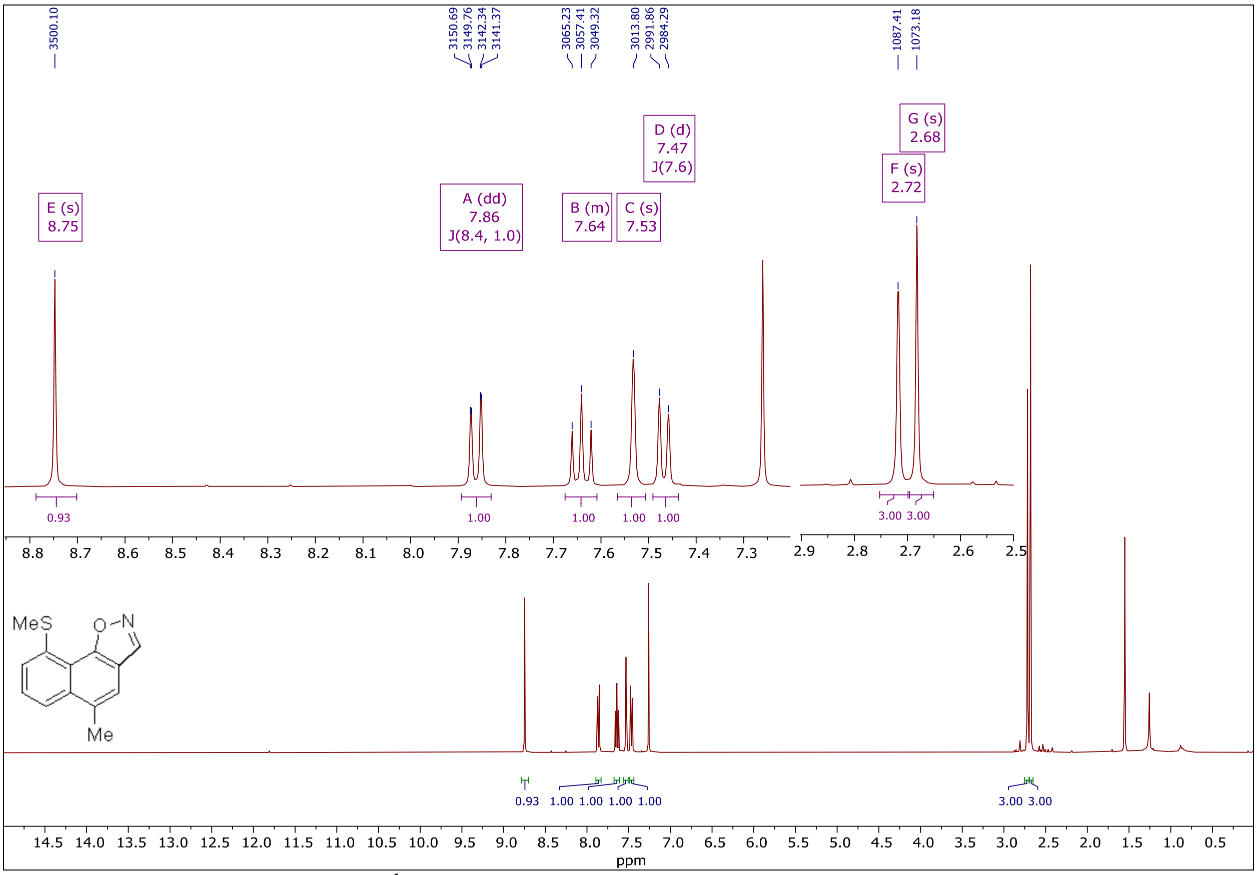

Fig. S49. ^{1}H NMR spectrum of compound **7d** (400 MHz, $CDCl_3$).

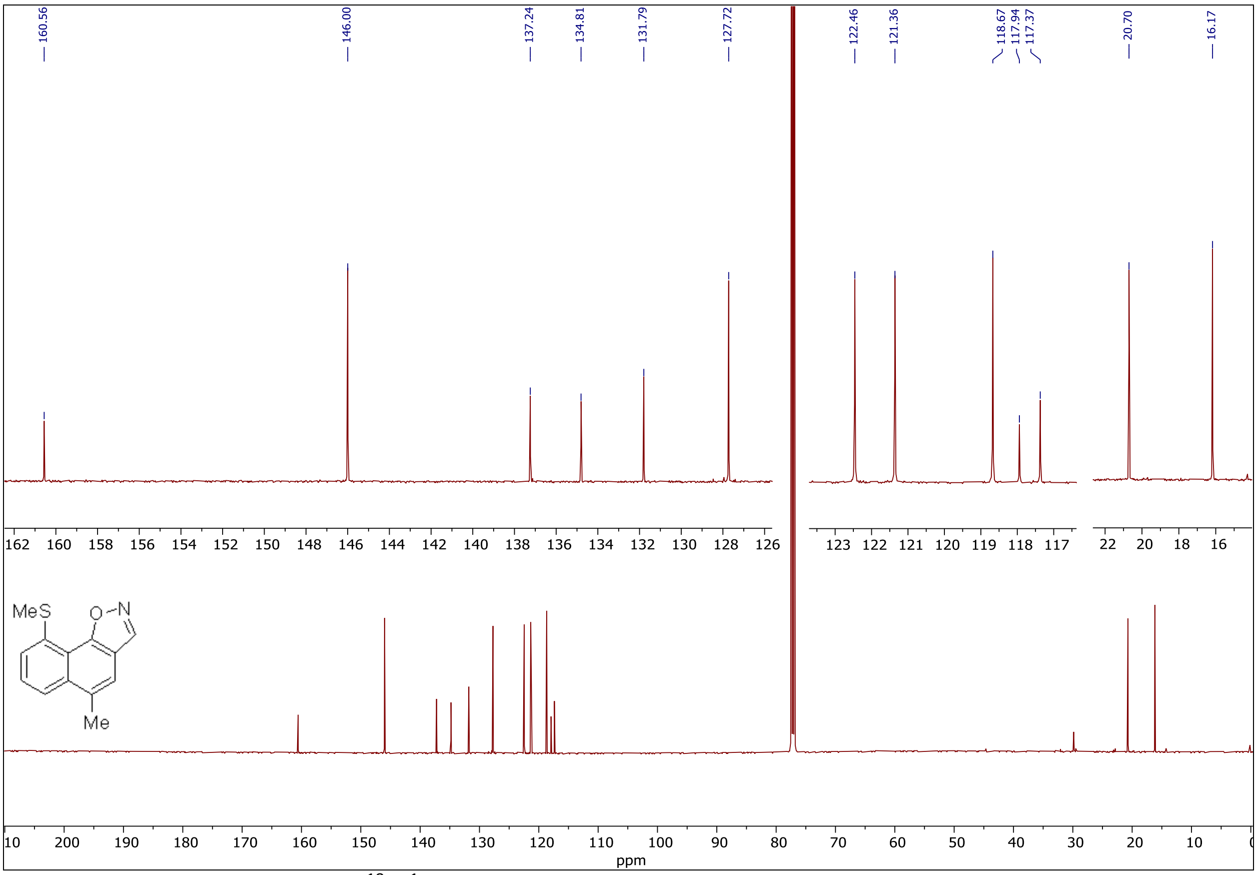

Fig. S50. $^{13}C\{^{1}H\}$ NMR spectrum of compound **7d** (100 MHz, $CDCl_3$).

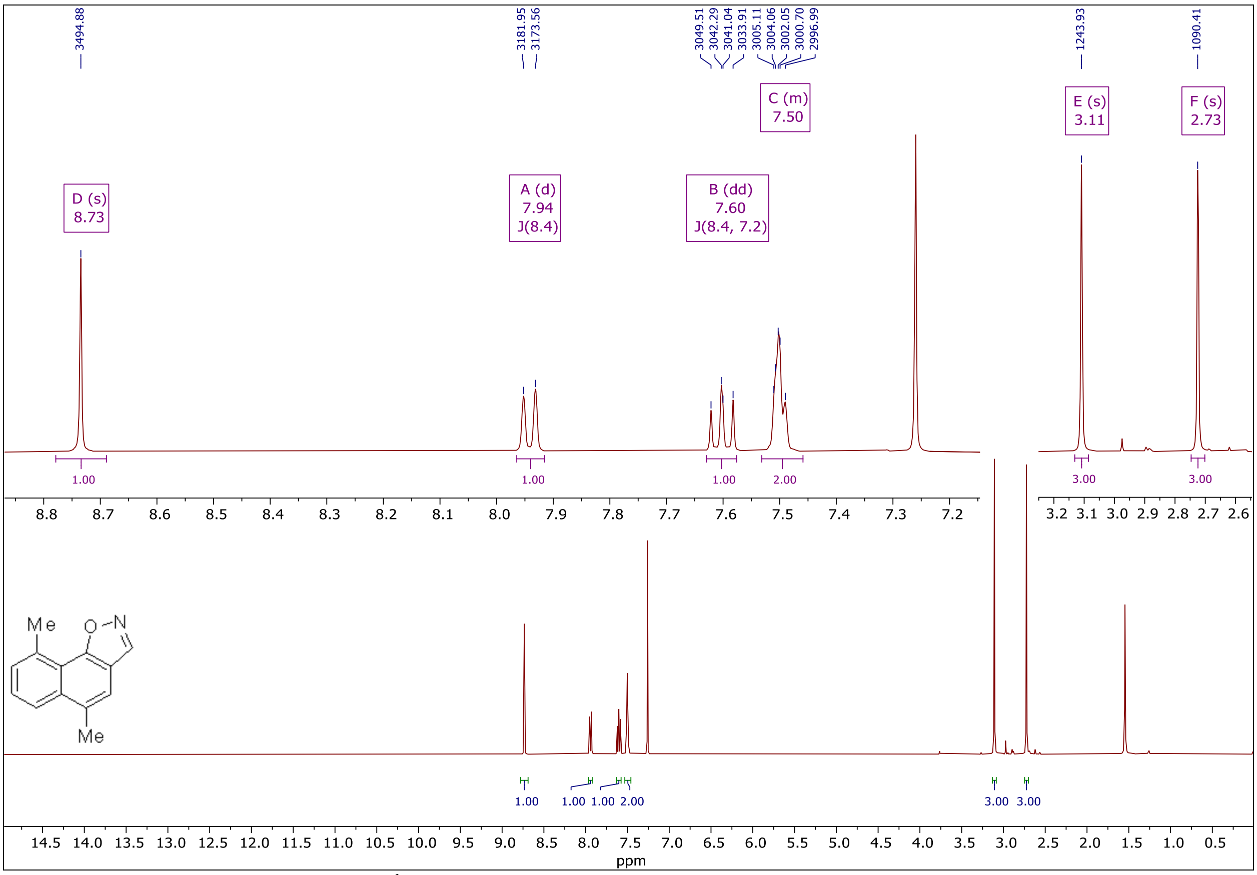

Fig. S51. ^{1}H NMR spectrum of compound **7e** (400 MHz, $CDCl_3$).

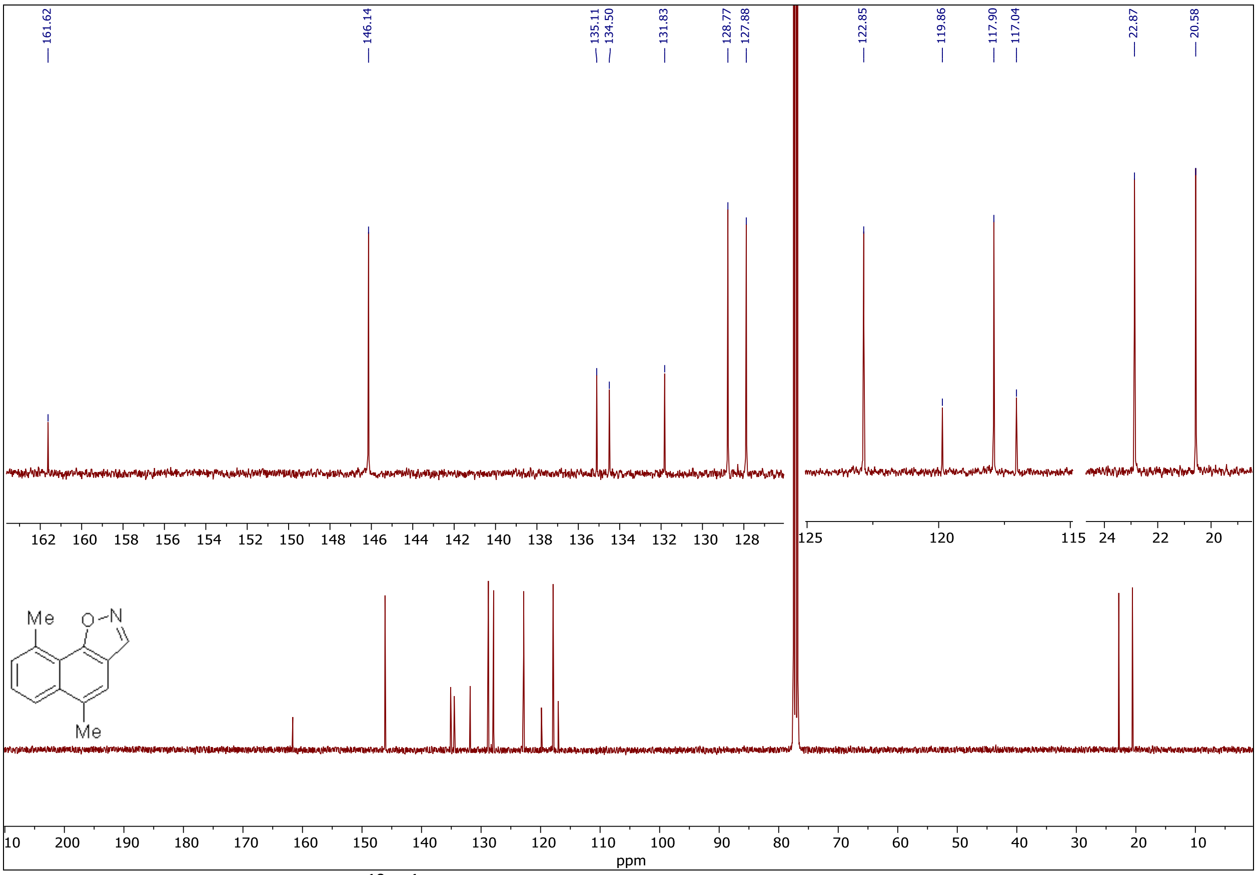

Fig. S52. $^{13}C\{^{1}H\}$ NMR spectrum of compound **7e** (100 MHz, $CDCl_3$).

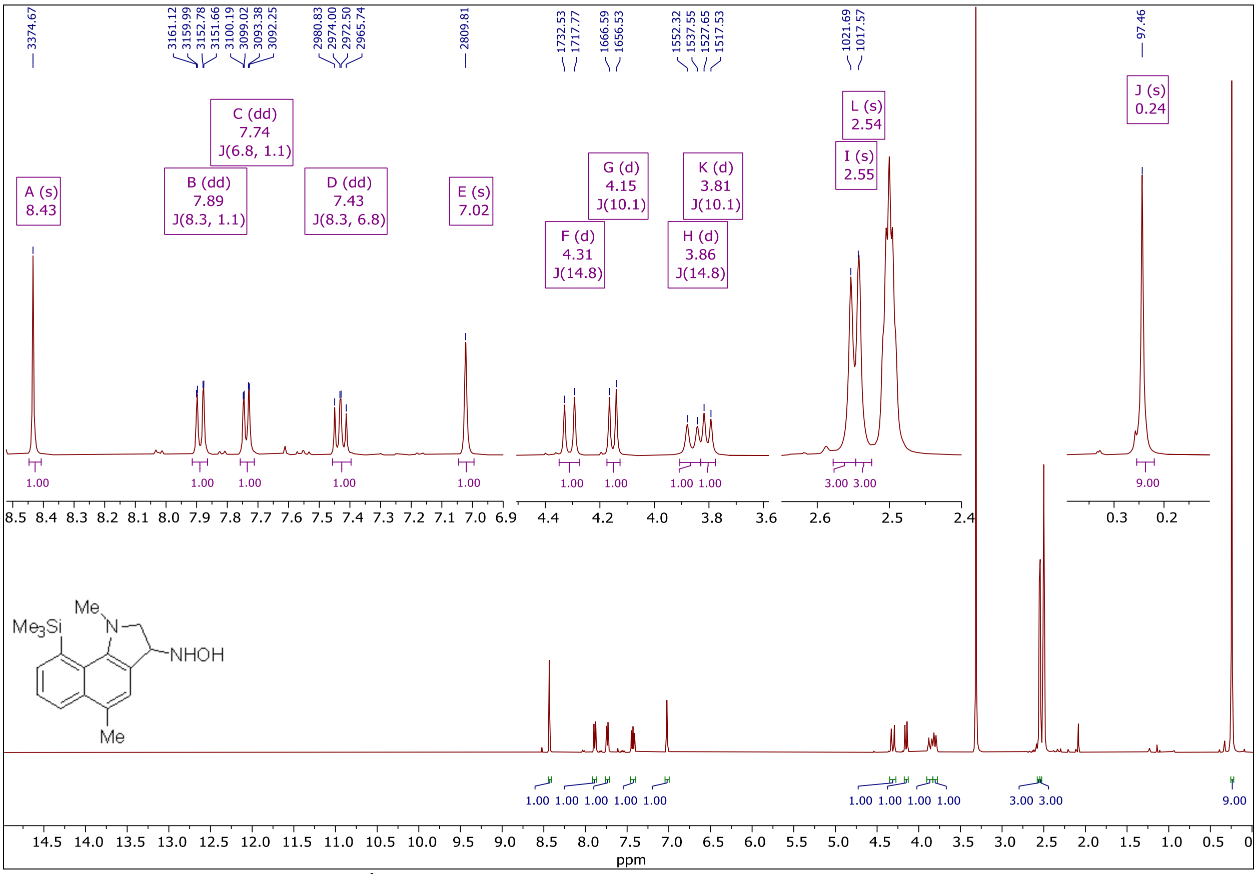

Fig. S53. ^{1}H NMR spectrum of compound **21b** (400 MHz, DMSO-d_6).

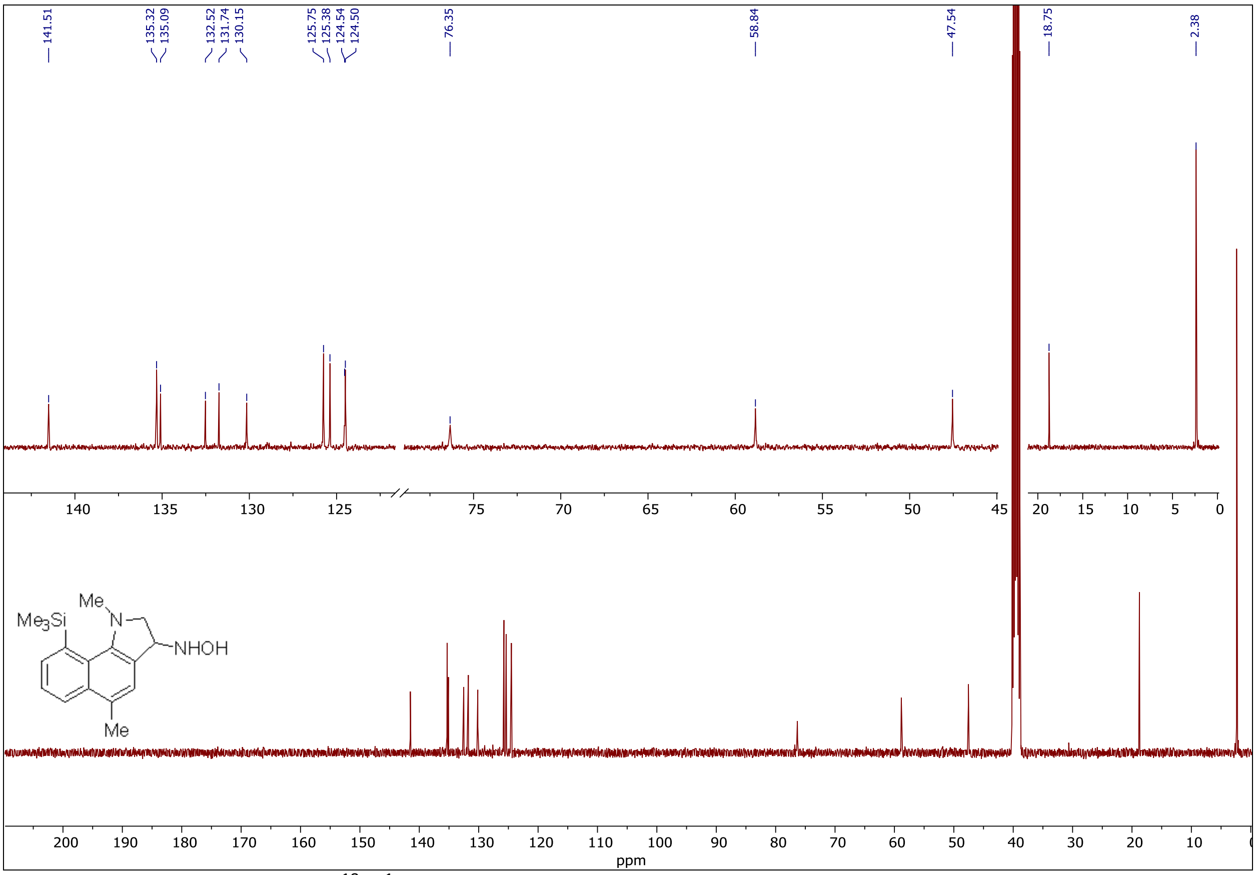

Fig. S54. ^{13}C{^{1}H} NMR spectrum of compound **21b** (100 MHz, DMSO-d_6).

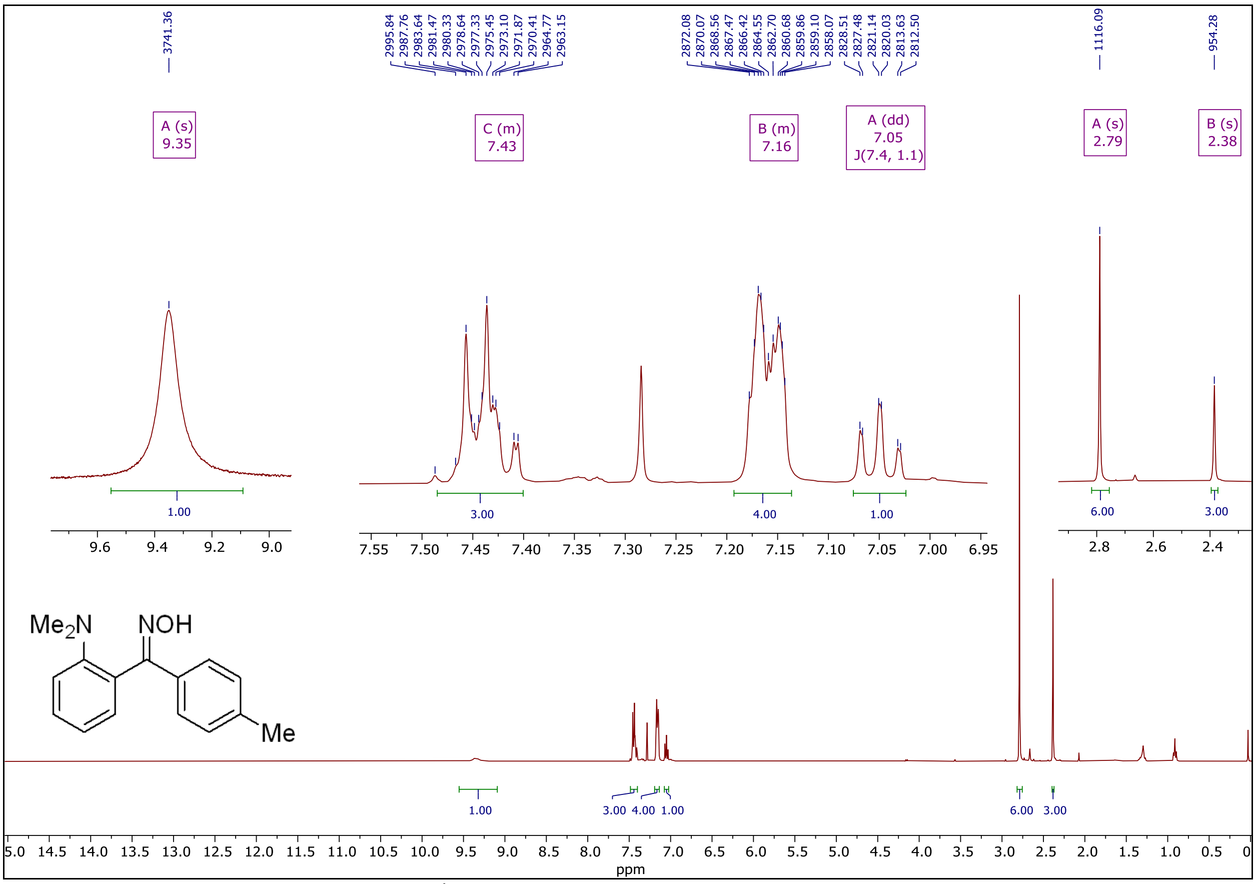

Fig. S55. ^{1}H NMR spectrum of compound **9a** (400 MHz, $CDCl_3$).

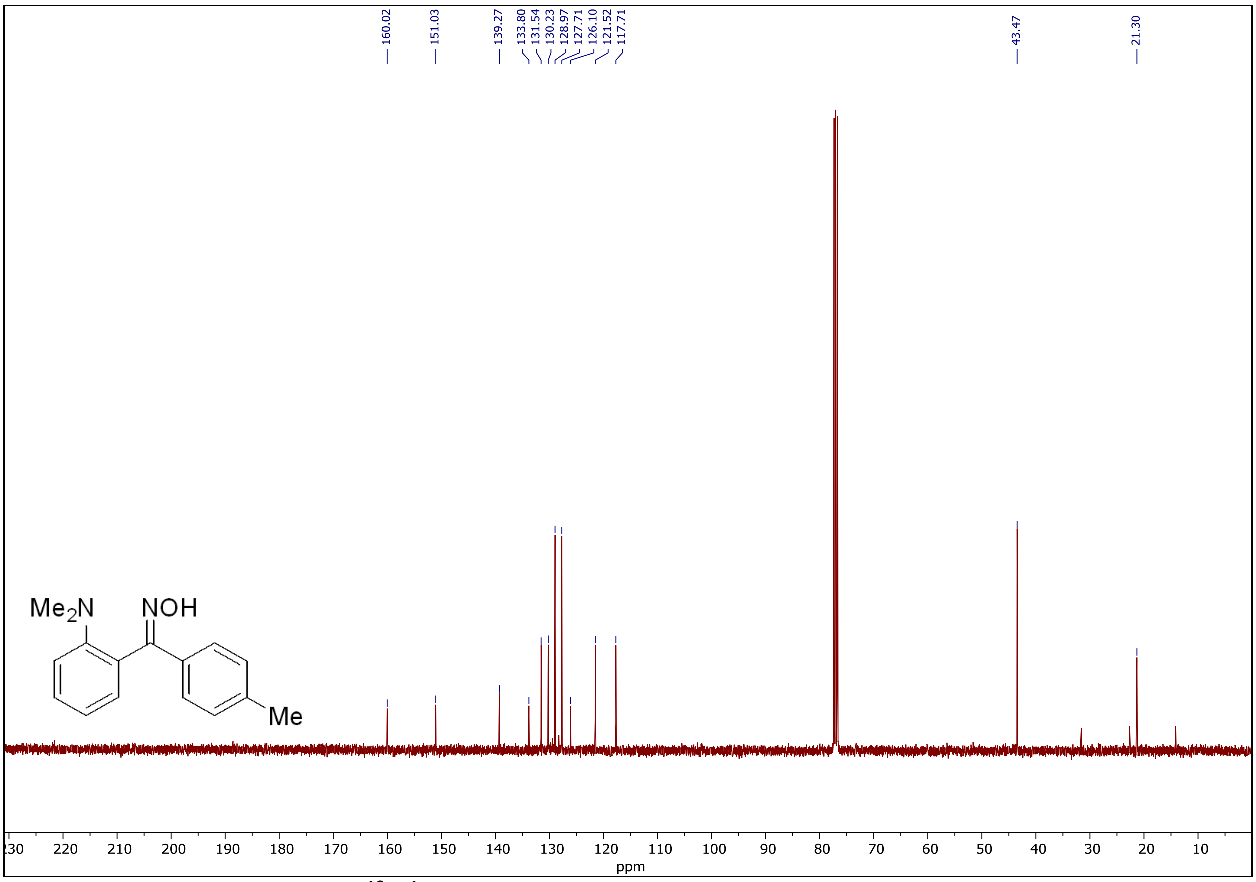

Fig. S56. $^{13}C\{^{1}H\}$ NMR spectrum of compound **9a** (100 MHz, $CDCl_3$).

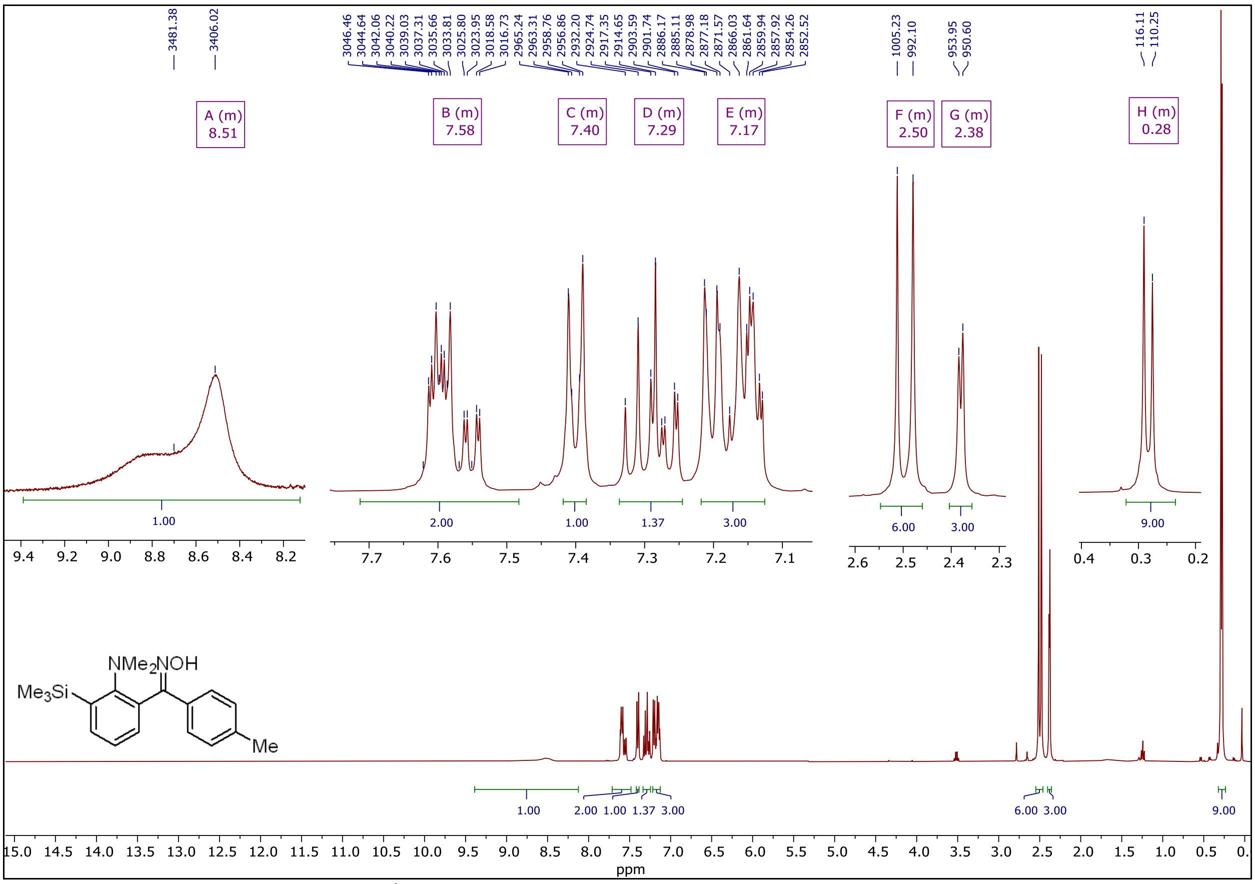

Fig. S57. ^{1}H NMR spectrum of compound **9b** (400 MHz, $CDCl_3$).

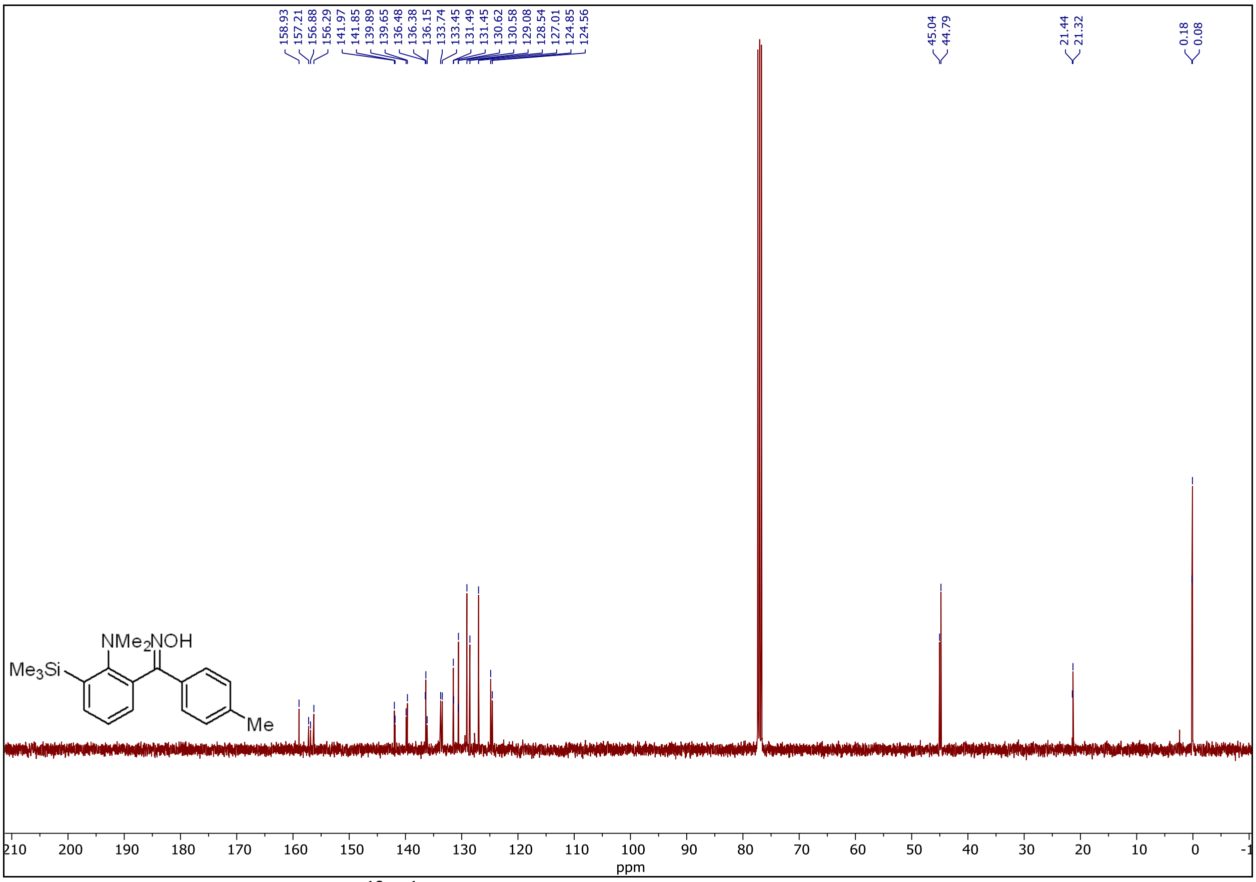

Fig. S58. $^{13}C\{^1H\}$ NMR spectrum of compound **9b** (100 MHz, $CDCl_3$).

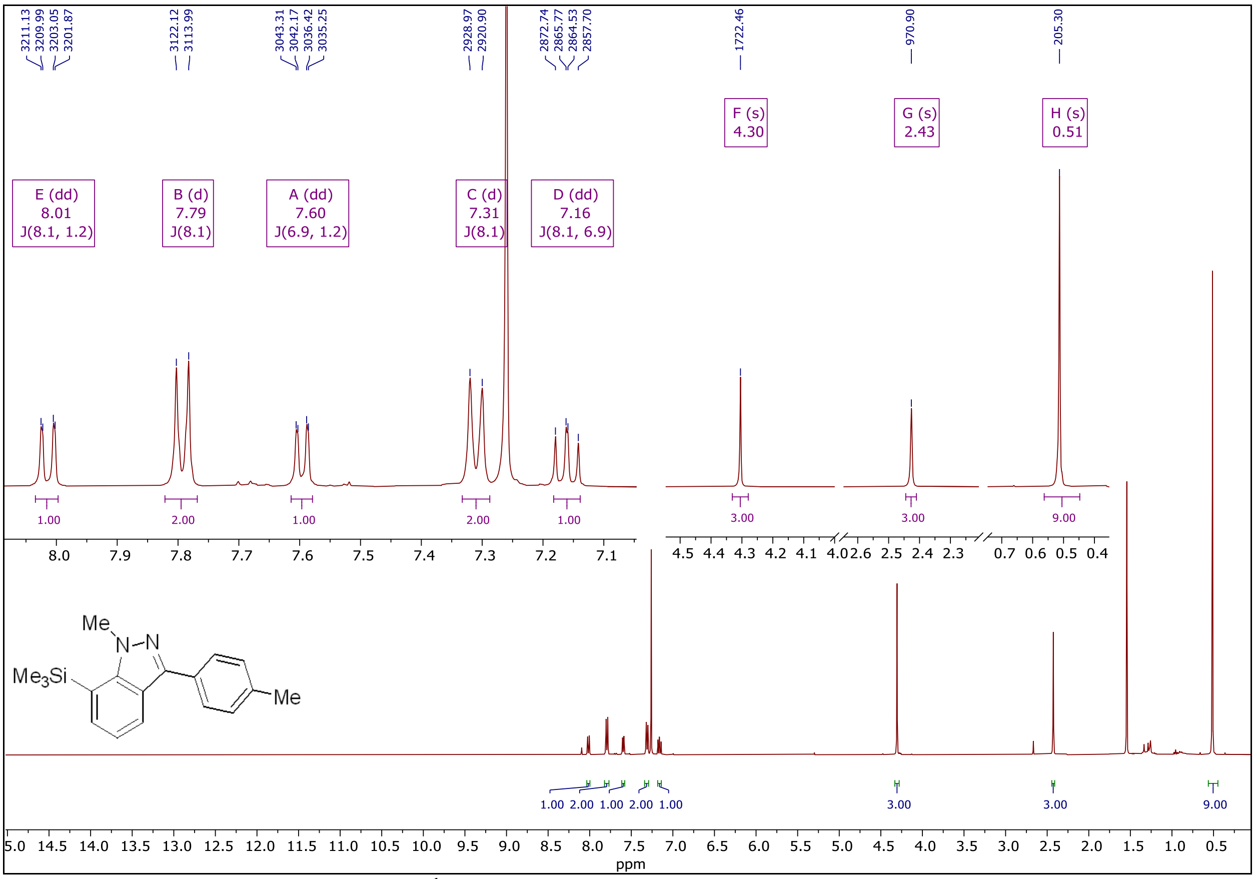

Fig. S59. ^{1}H NMR spectrum of compound **10b** (400 MHz, $CDCl_3$).

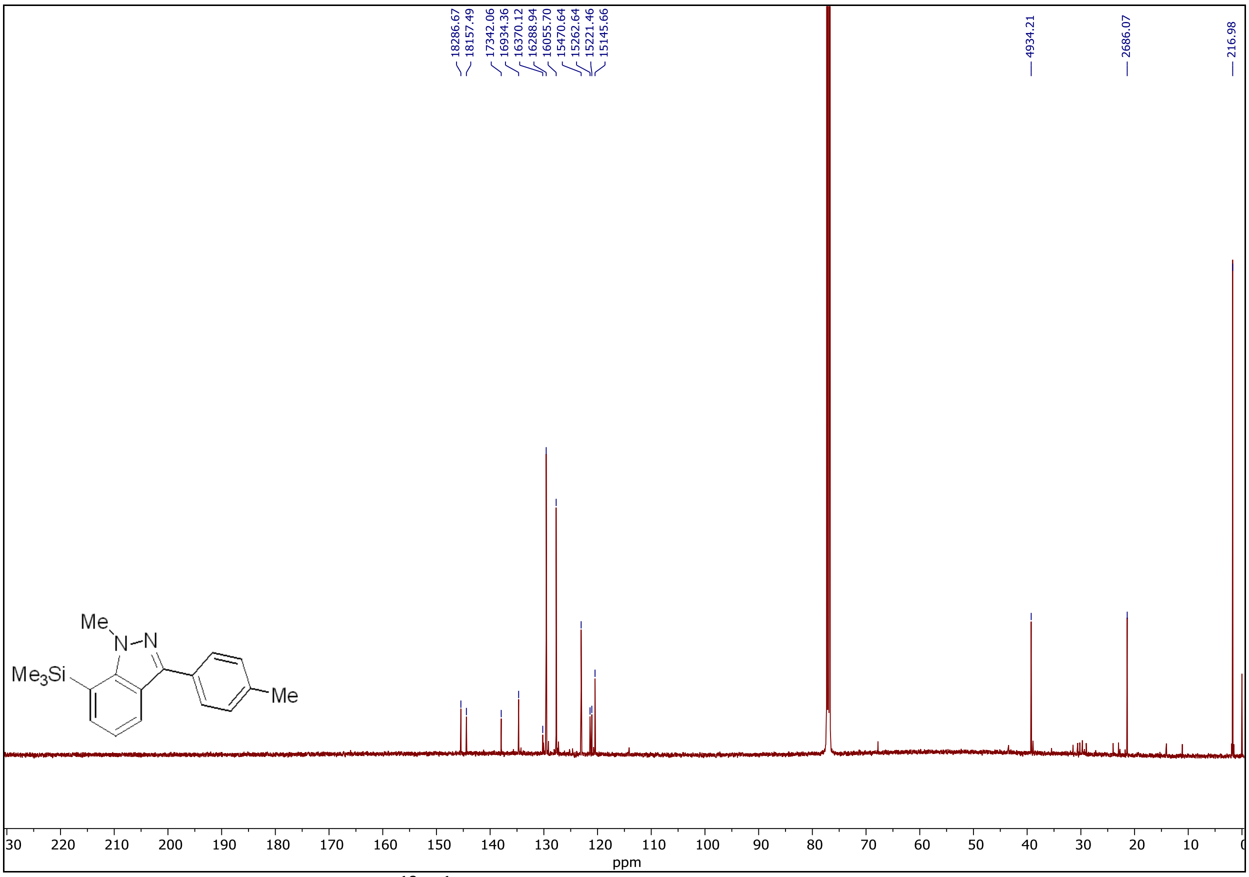

Fig. S60.^{13}C{^{1}H} NMR spectrum of compound **10b** (100 MHz, $CDCl_3$).

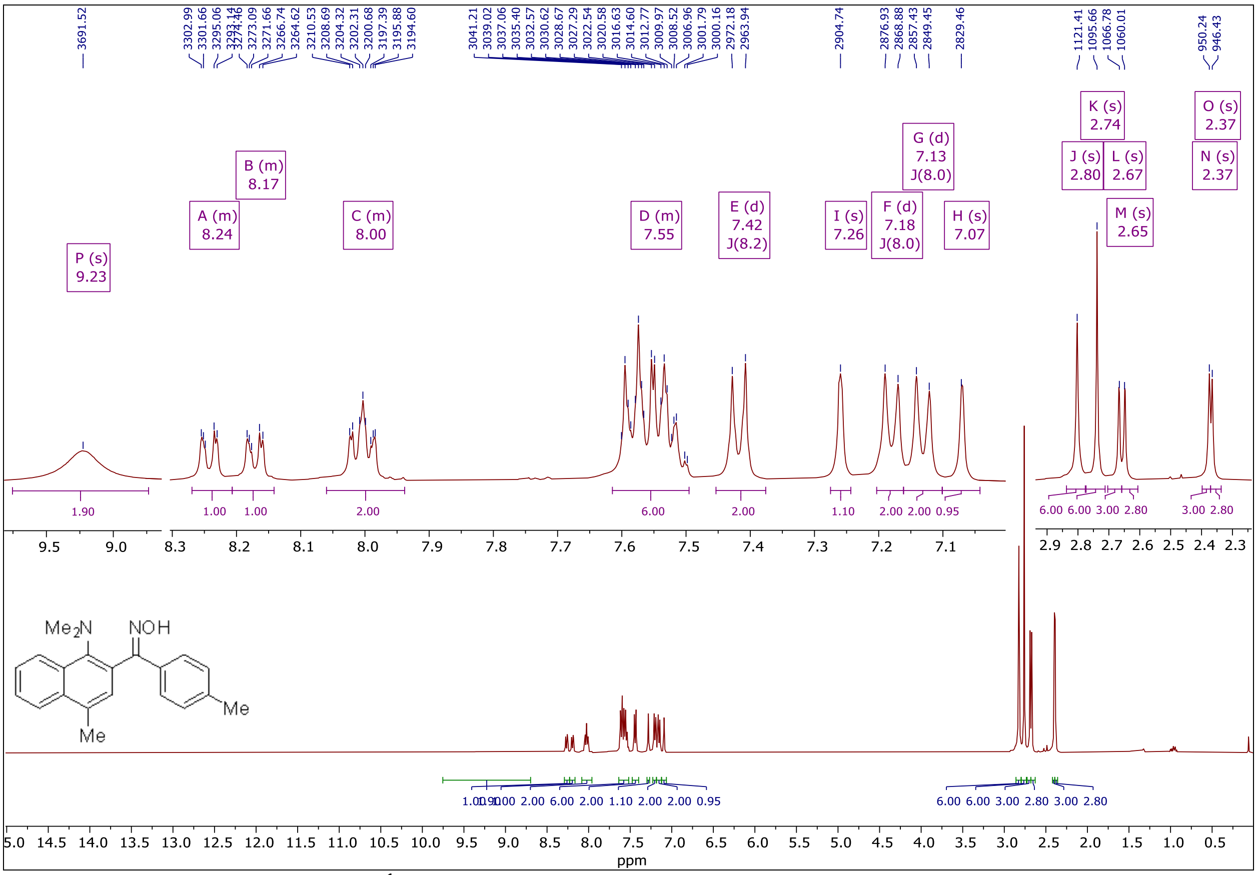

Fig. S61. ^{1}H NMR spectrum of compound **11a** (400 MHz, $CDCl_3$).

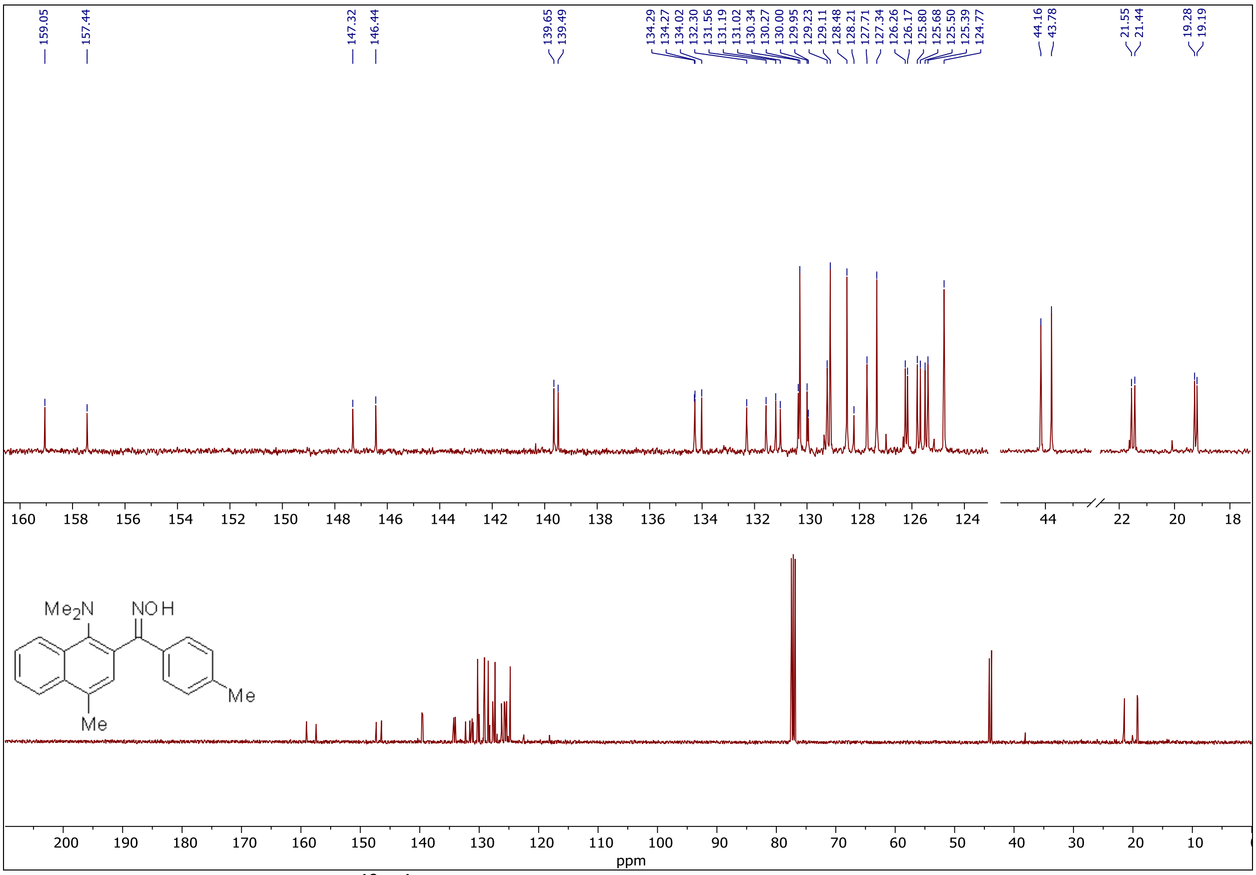

Fig. S62. ^{13}C{^{1}H} NMR spectrum of compound **11a** (100 MHz, $CDCl_3$).

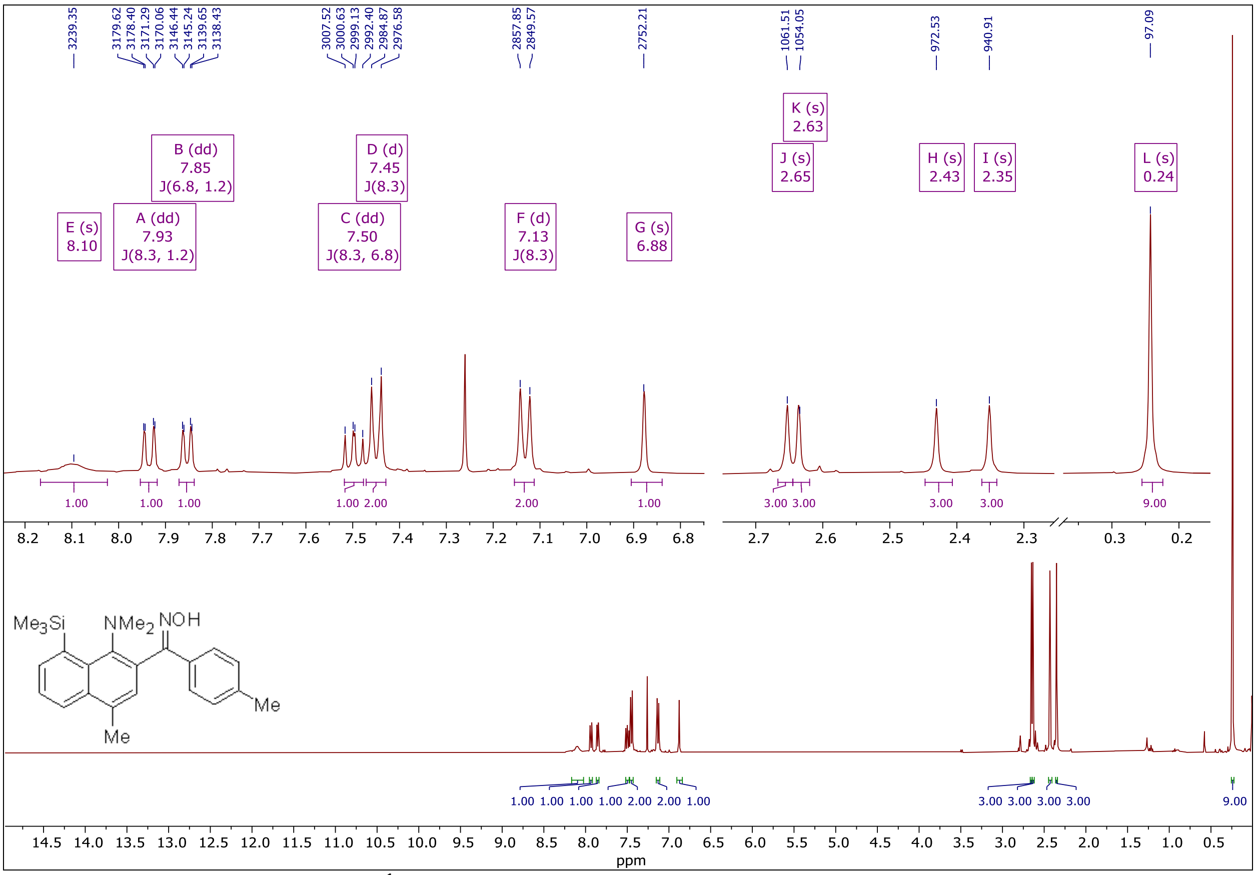

Fig. S63. ^{1}H NMR spectrum of compound **11c** (400 MHz, $CDCl_3$).

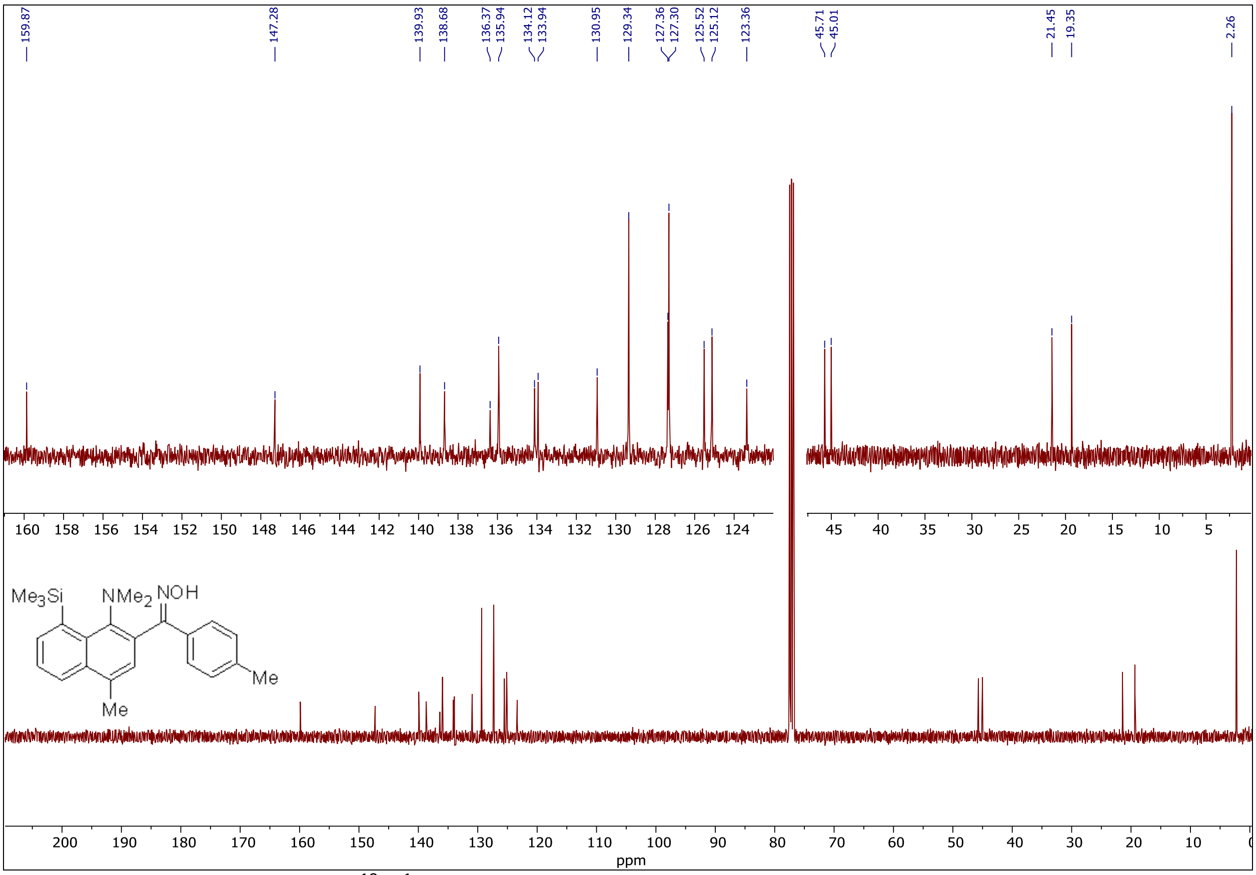

Fig. S64. $^{13}C\{^{1}H\}$ NMR spectrum of compound **11c** (100 MHz, $CDCl_3$).

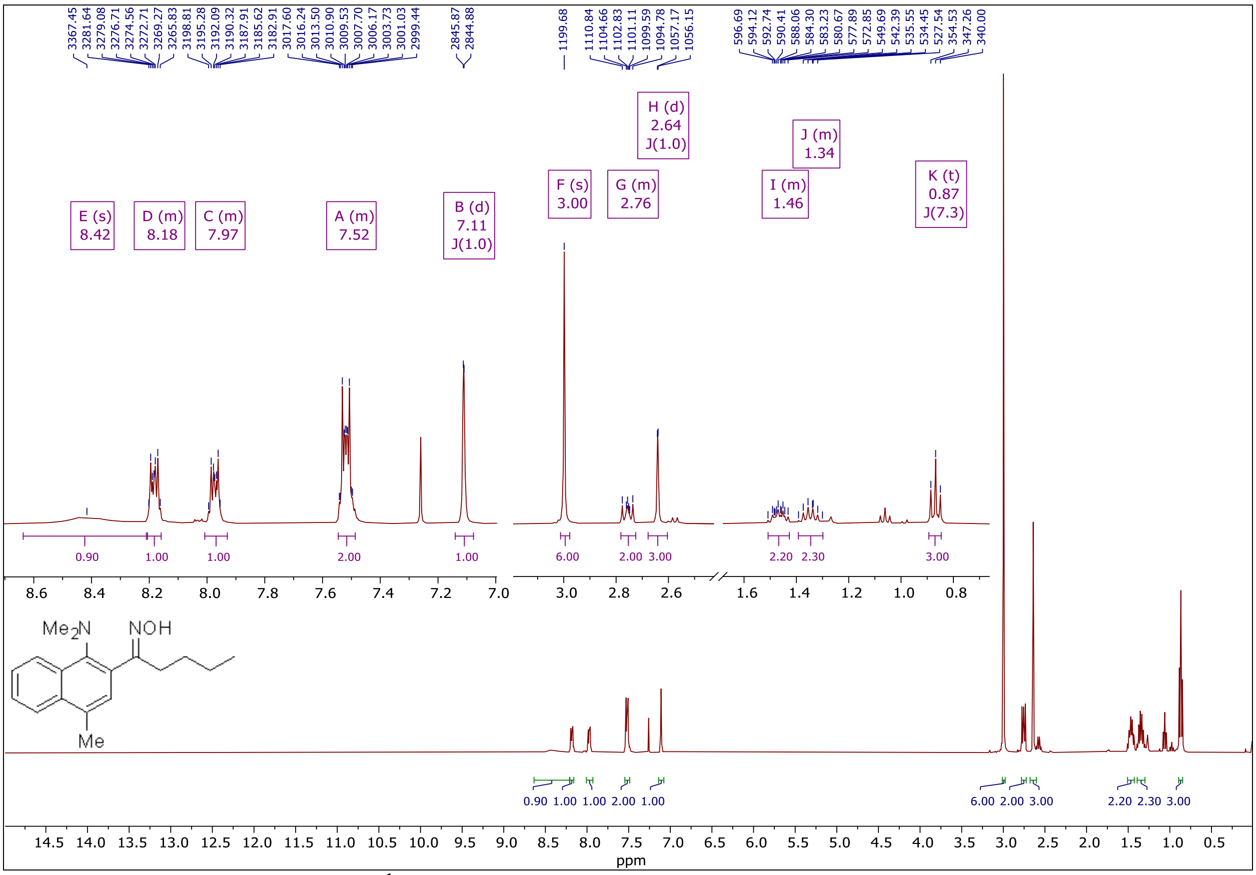

Fig. S65. ^{1}H NMR spectrum of compound **11b** (400 MHz, $CDCl_3$).

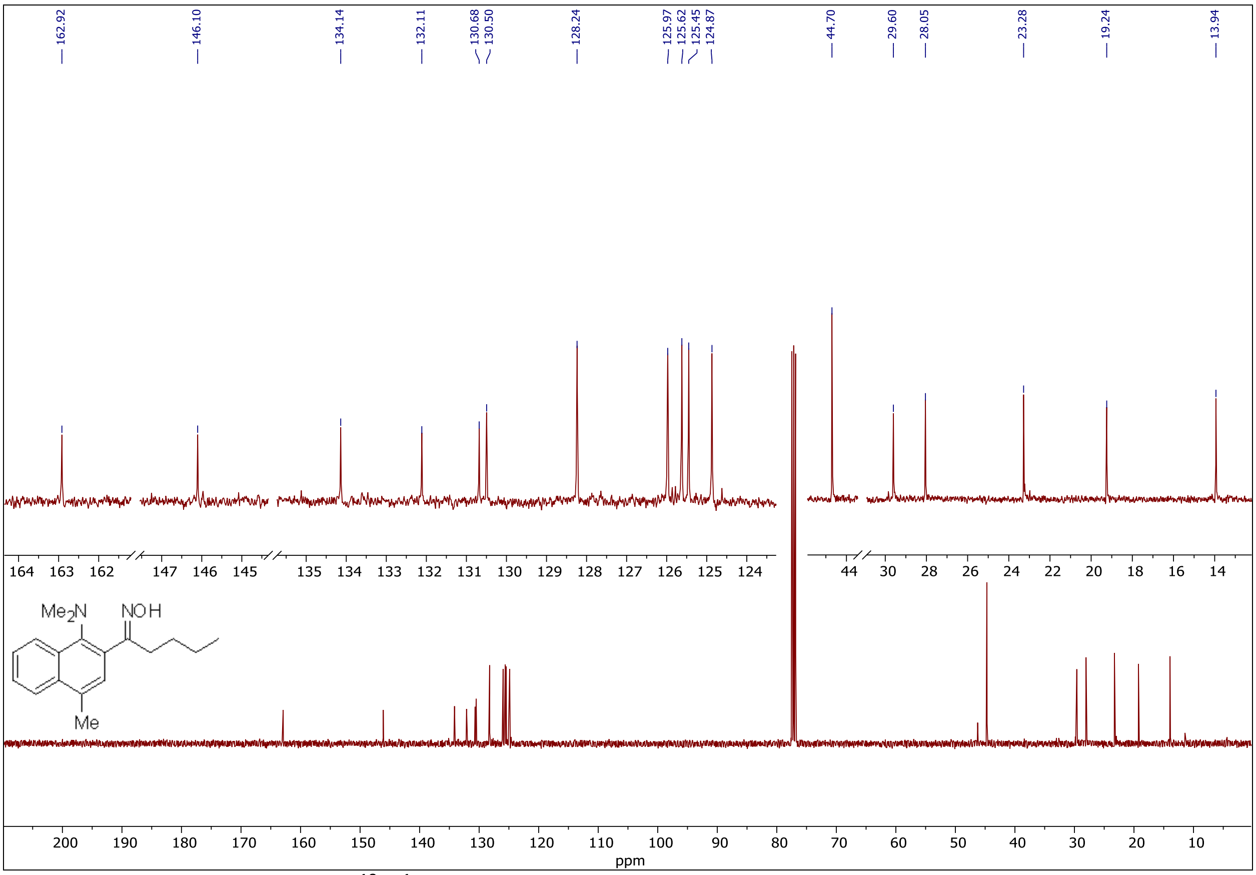

Fig. S66. ^{13}C{^{1}H} NMR spectrum of compound **11b** (100 MHz, $CDCl_3$).

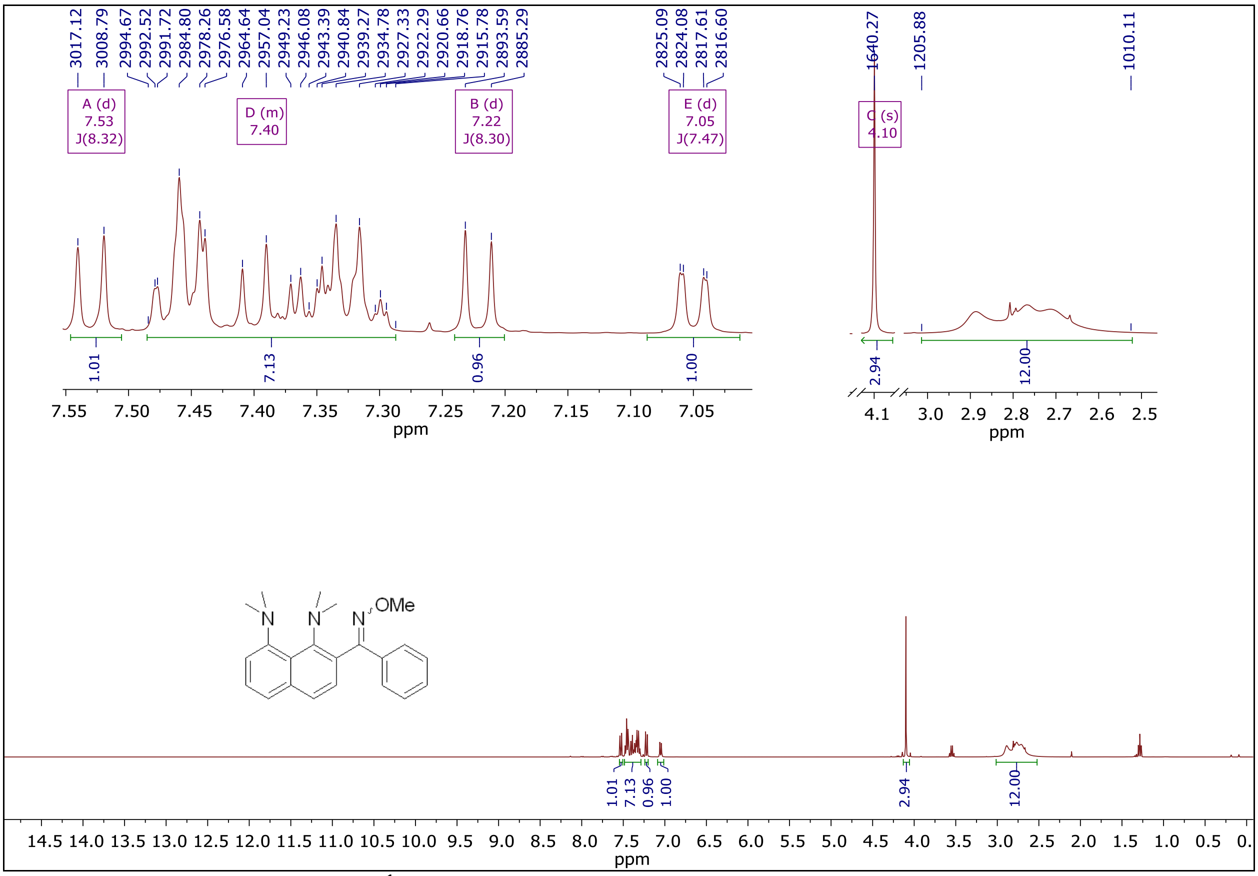

Fig. S67. ^{1}H NMR spectrum of compound **11g** (400 MHz, $CDCl_3$).

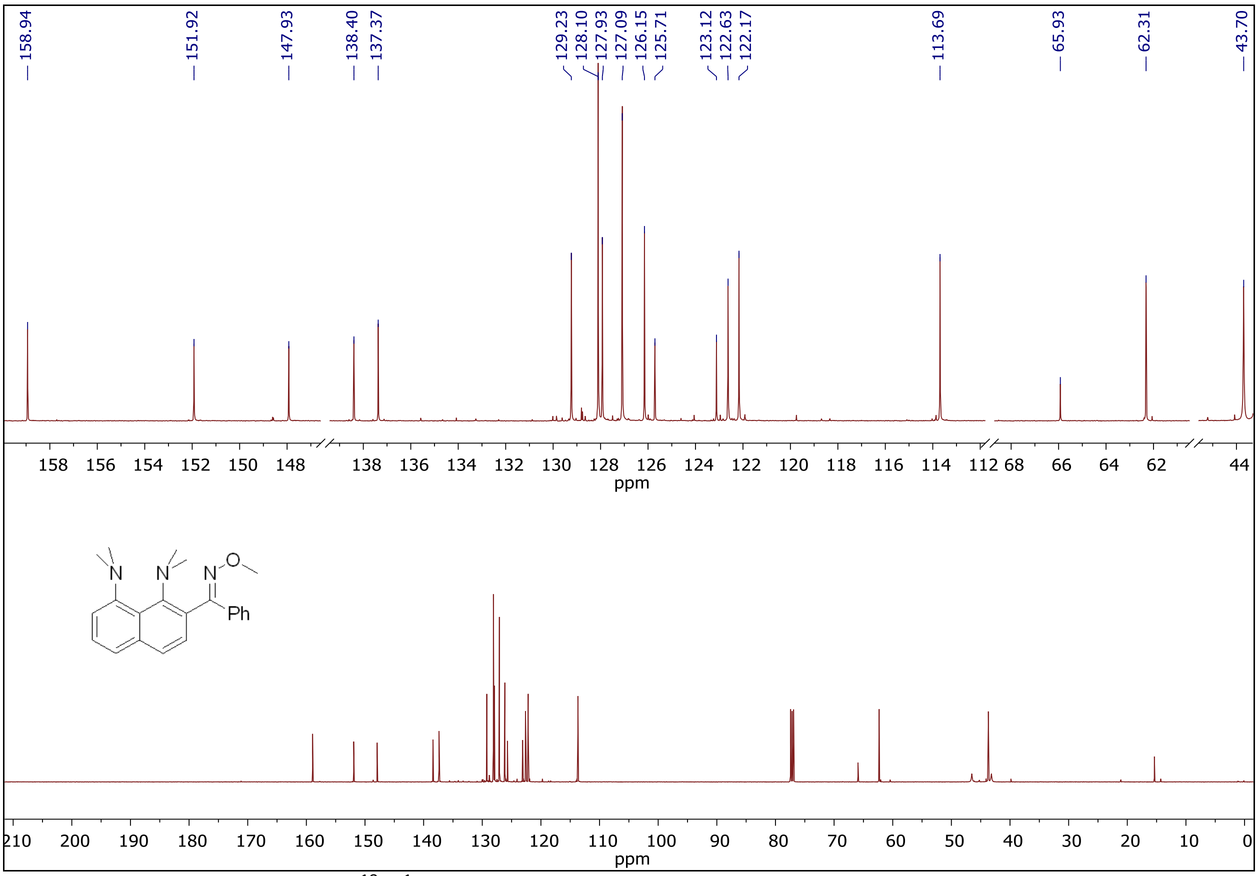

Fig. S68. $^{13}C\{^1H\}$ NMR spectrum of compound **11g** (100 MHz, $CDCl_3$).

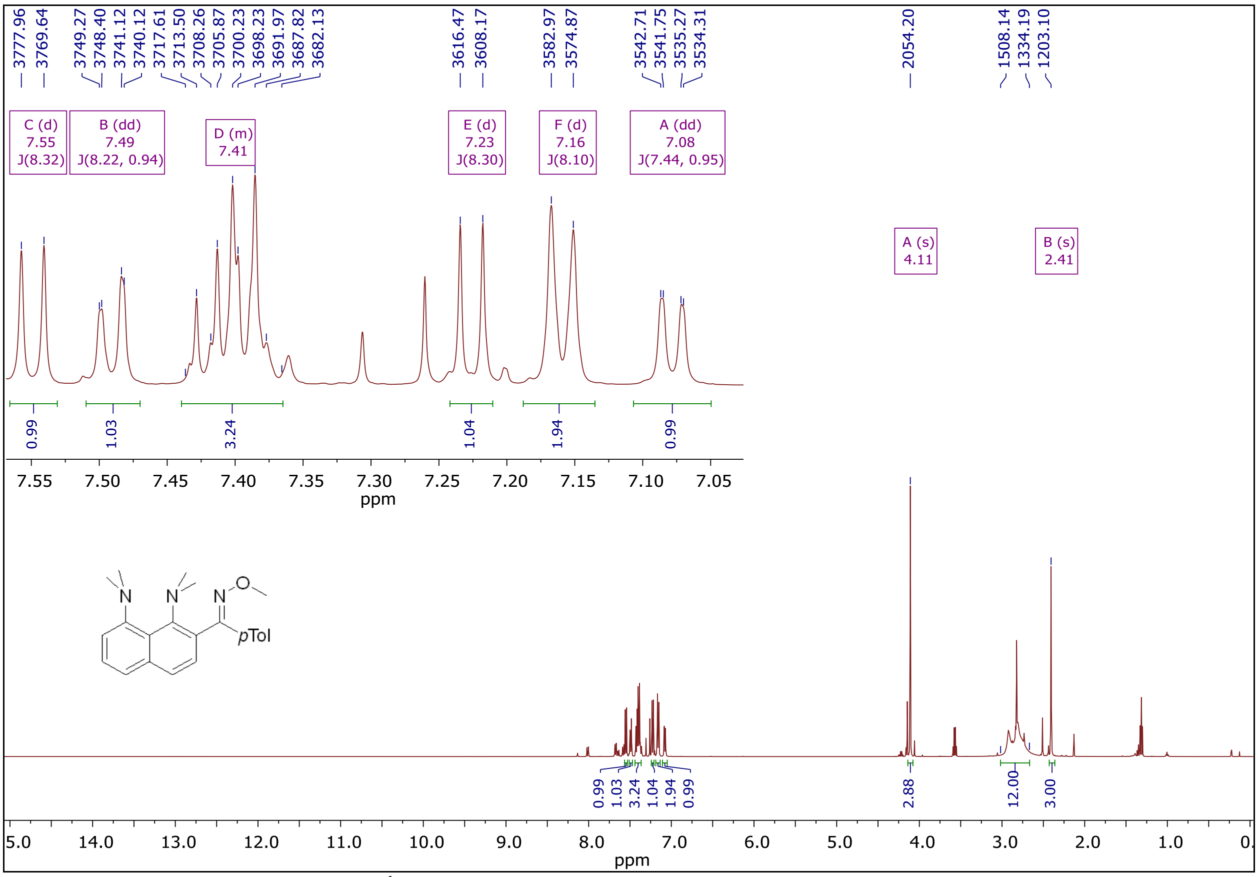

Fig. S69. ^{1}H NMR spectrum of compound **11h** (400 MHz, $CDCl_3$).

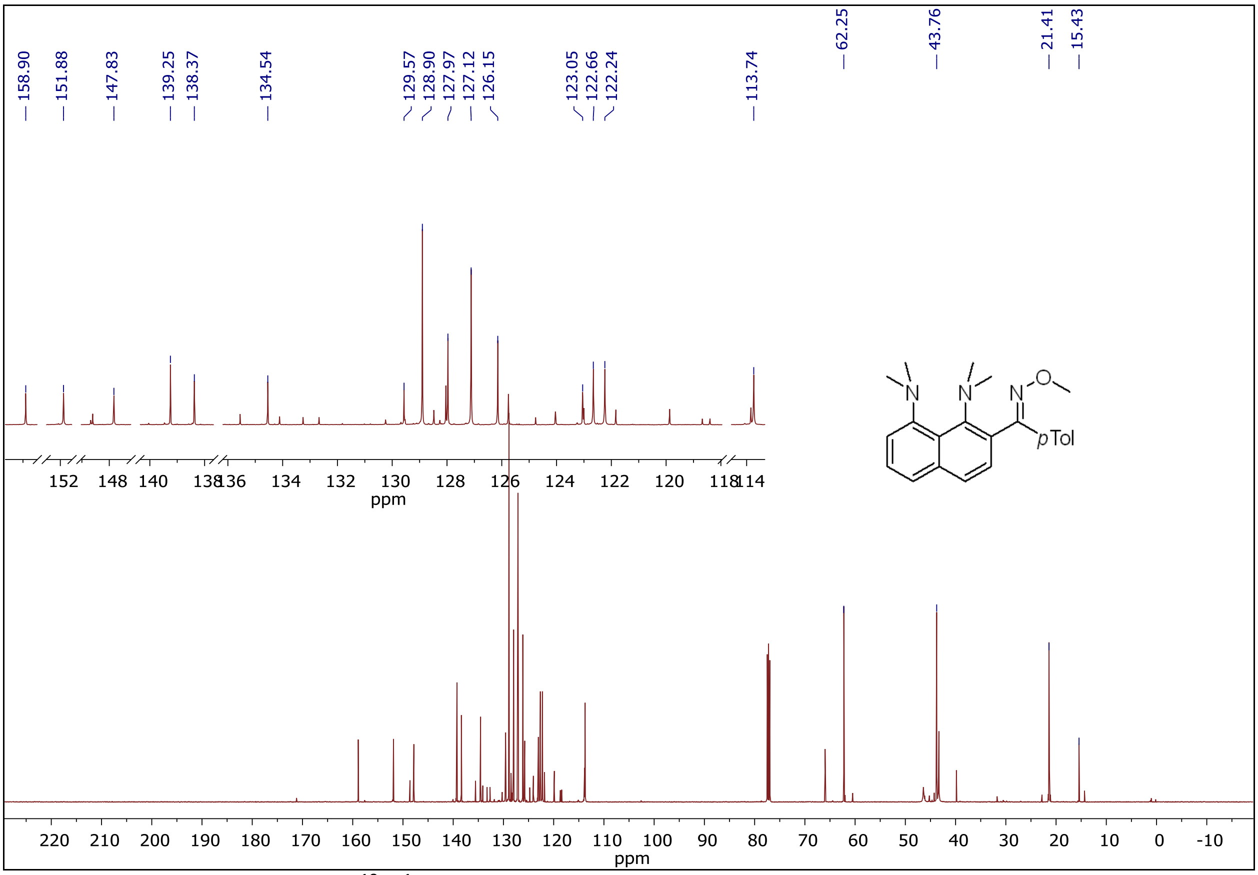

Fig. S70. $^{13}C\{^{1}H\}$ NMR spectrum of compound **11h** (100 MHz, $CDCl_3$).

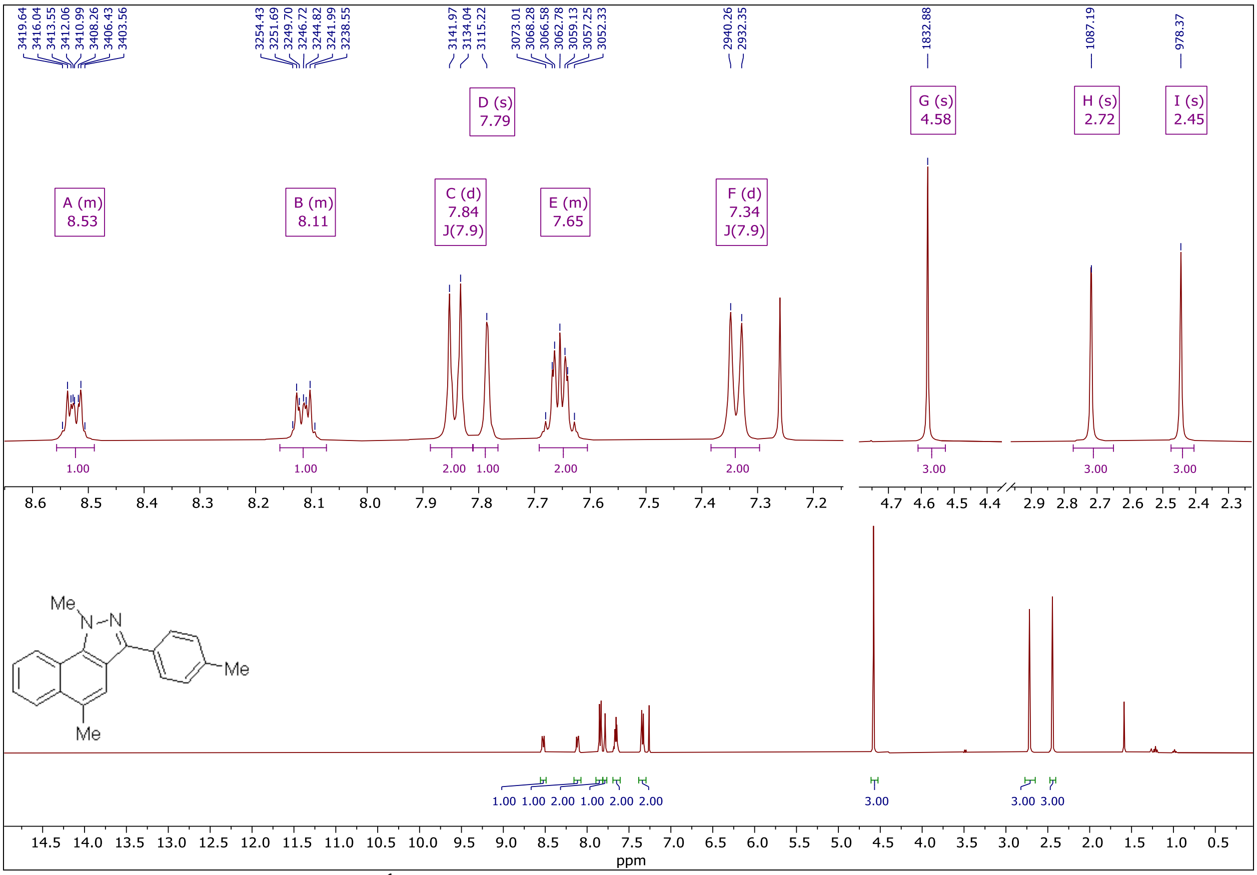

Fig. S71. ^{1}H NMR spectrum of compound **12a** (400 MHz, $CDCl_3$).

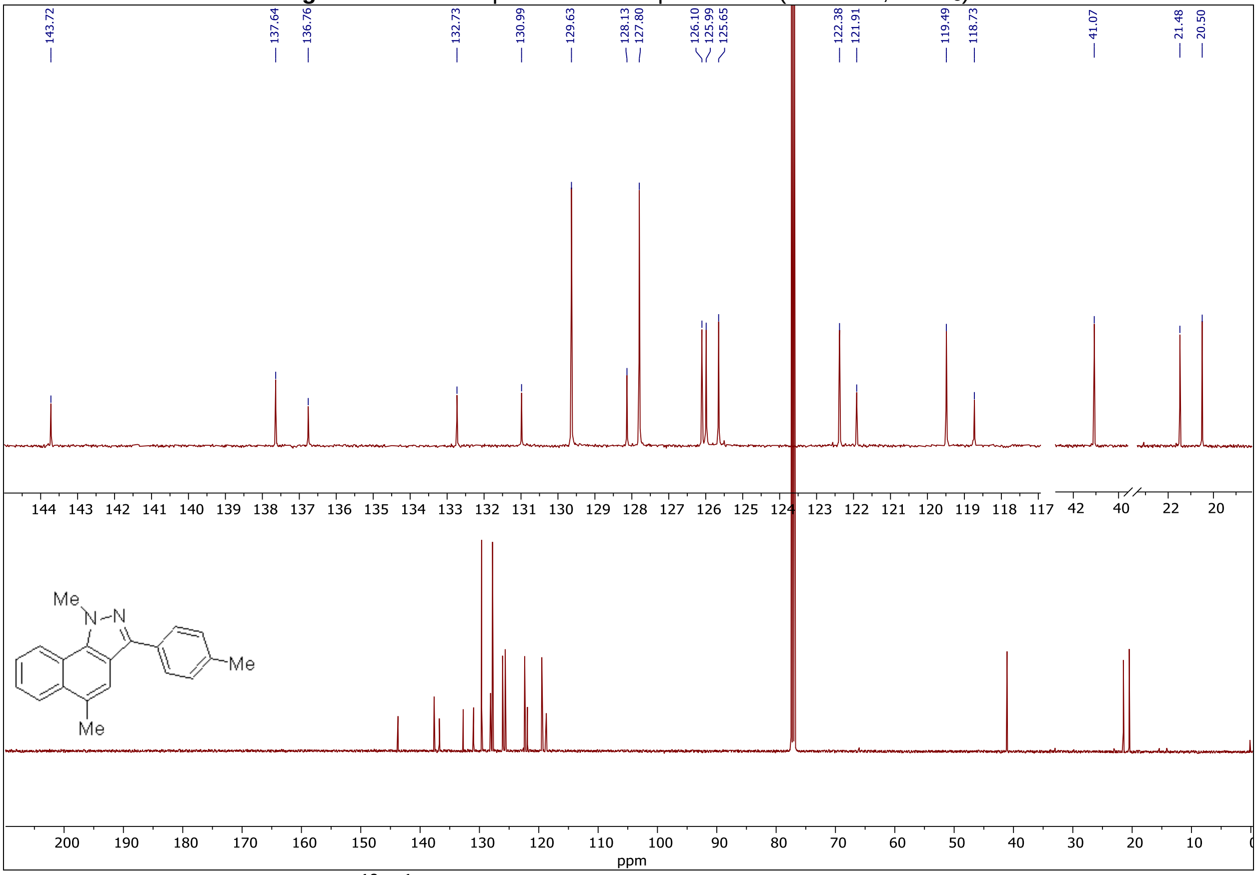

Fig. S72. ^{13}C{^{1}H} NMR spectrum of compound **12a** (100 MHz, $CDCl_3$).

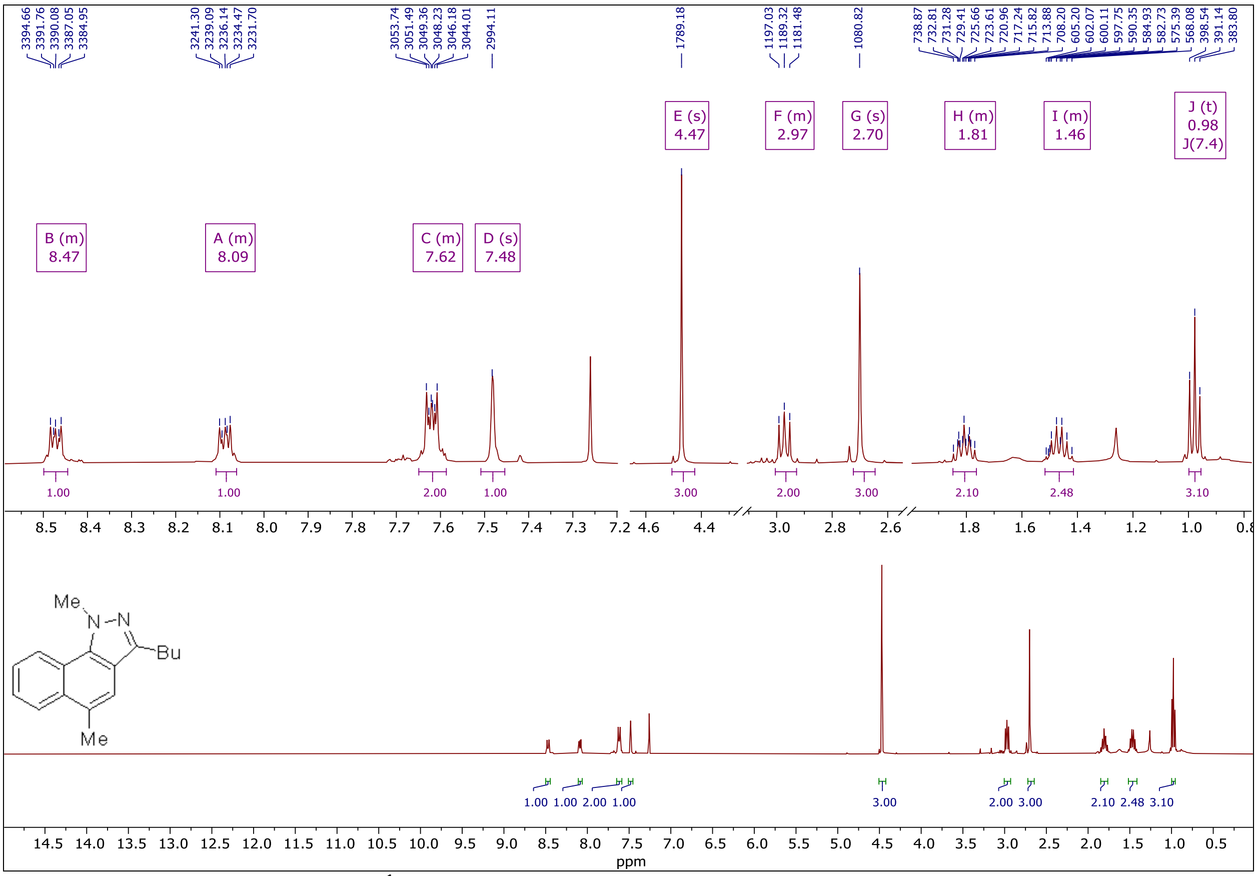

Fig. S73. ^{1}H NMR spectrum of compound **12b** (400 MHz, $CDCl_3$).

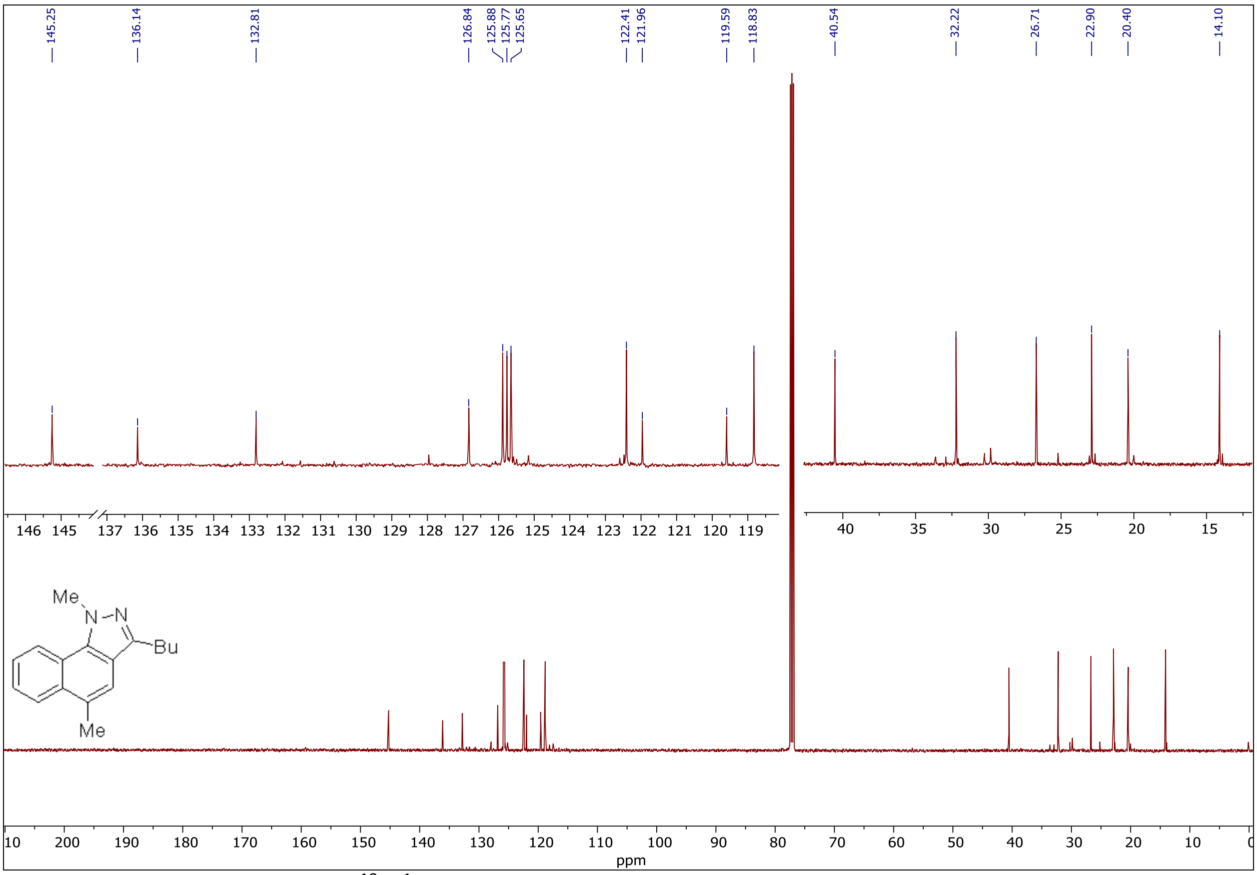

Fig. S74. ^{13}C{^{1}H} NMR spectrum of compound **12b** (100 MHz, $CDCl_3$).

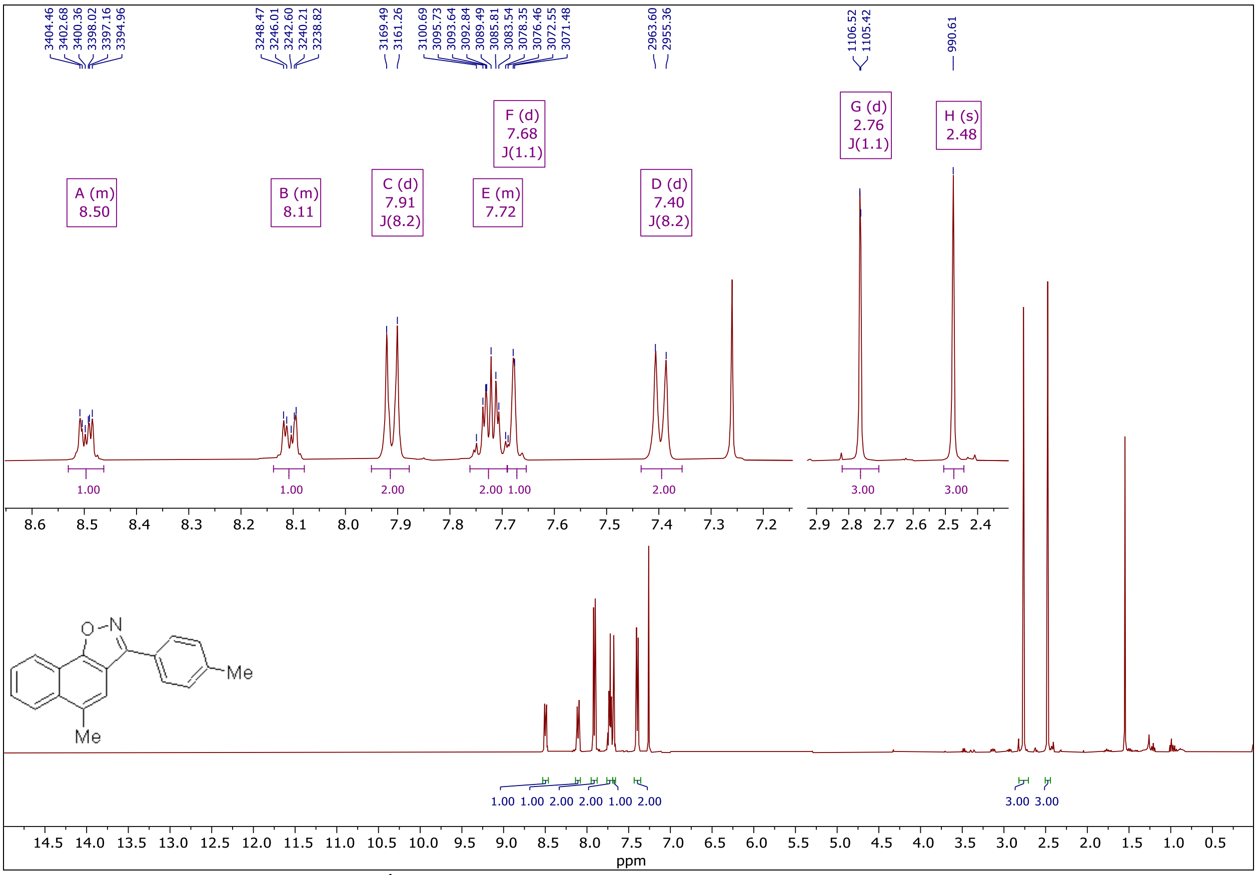

Fig. S75. ^{1}H NMR spectrum of compound **13a** (400 MHz, $CDCl_3$).

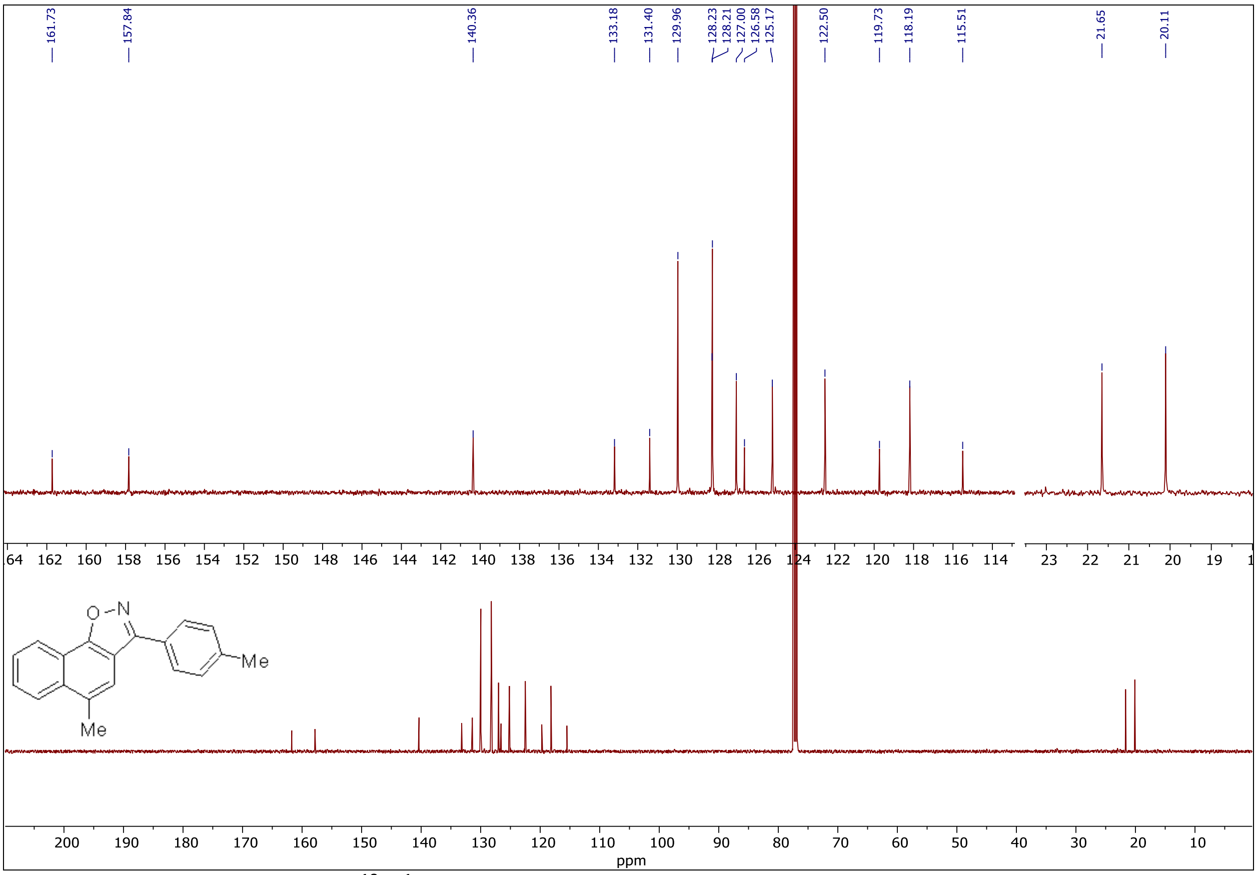

Fig. S76. ^{13}C{^{1}H} NMR spectrum of compound **13a** (100 MHz, $CDCl_3$).

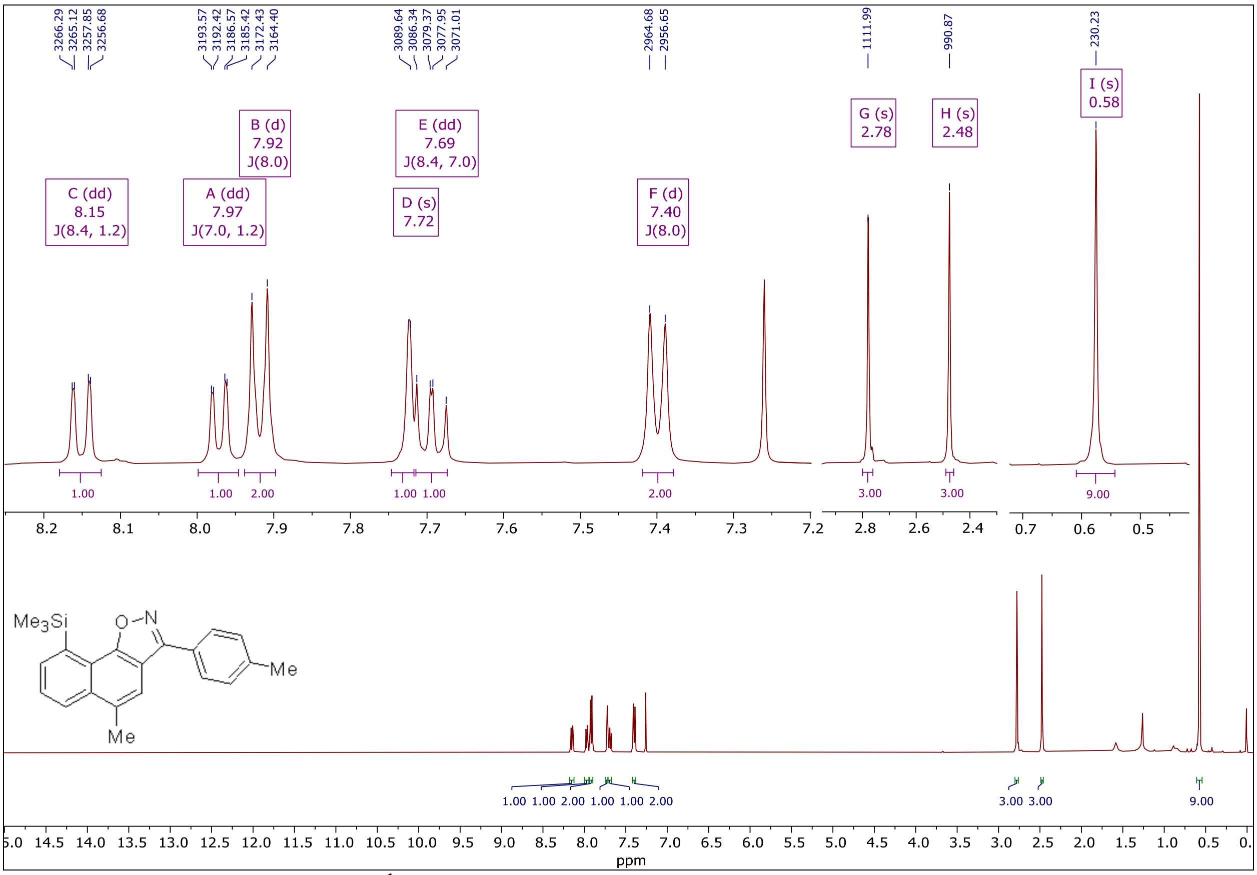

Fig. S77. ^{1}H NMR spectrum of compound **13c** (400 MHz, $CDCl_3$).

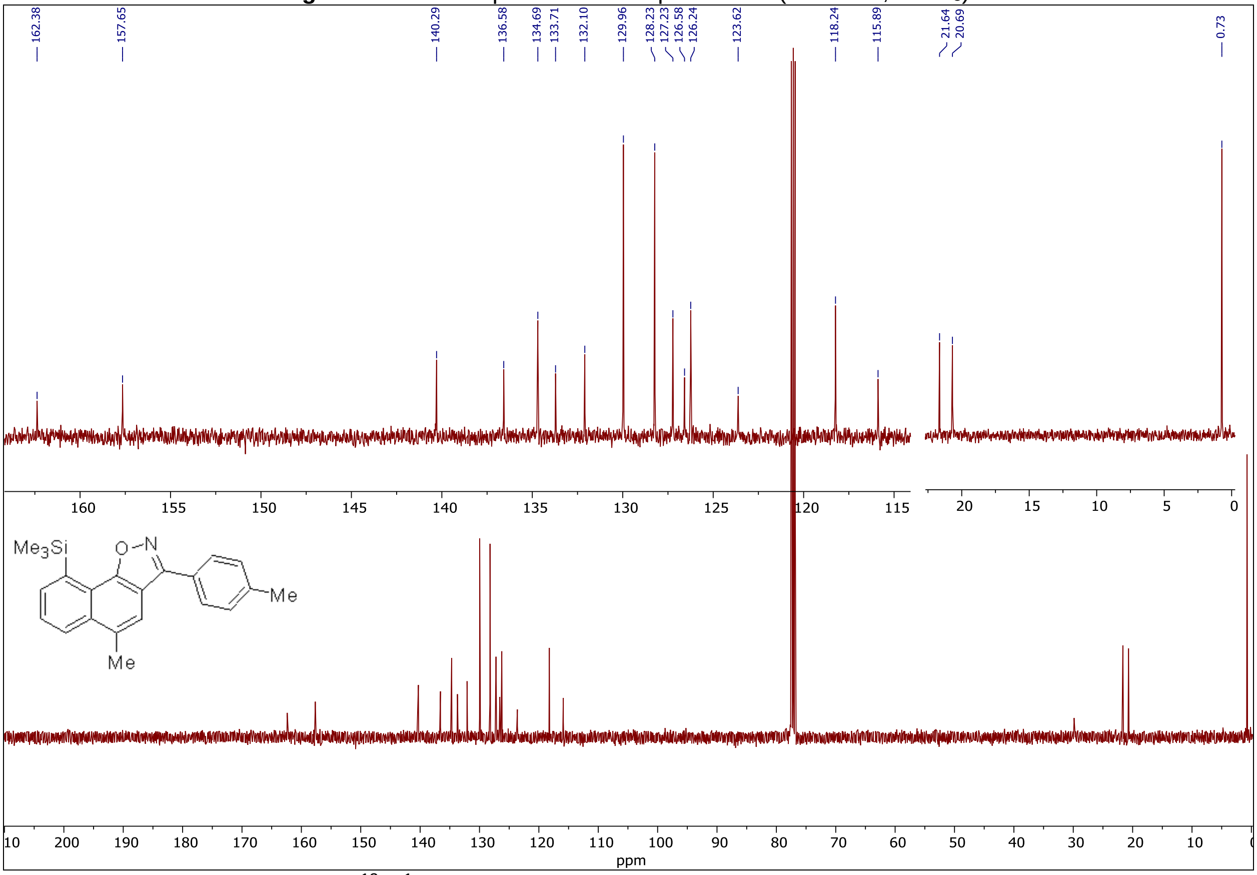

Fig. S78. $^{13}C\{^{1}H\}$ NMR spectrum of compound **13c** (100 MHz, $CDCl_3$).

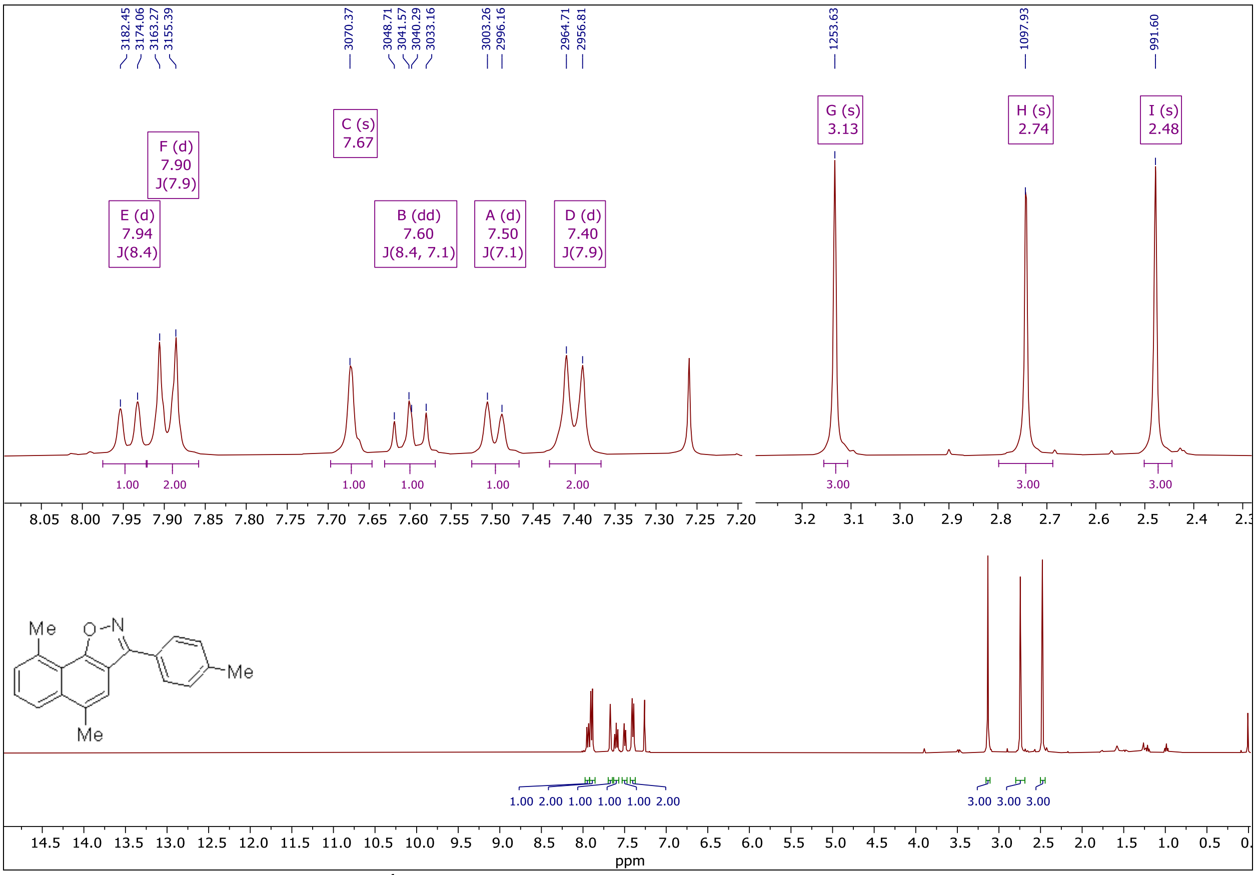

Fig. S79. ^{1}H NMR spectrum of compound **13f** (400 MHz, $CDCl_3$).

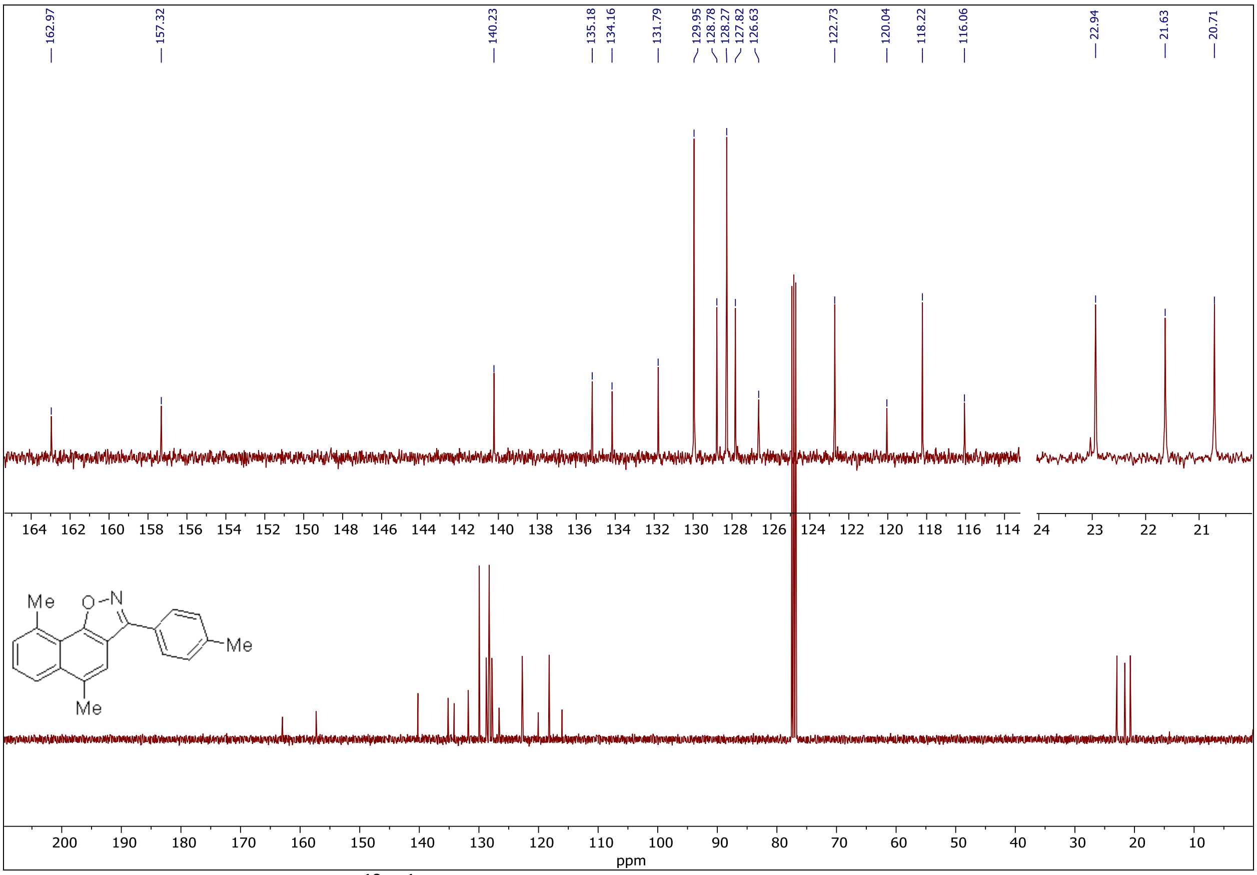

Fig. S80. $^{13}C\{^{1}H\}$ NMR spectrum of compound **13f** (100 MHz, $CDCl_3$).

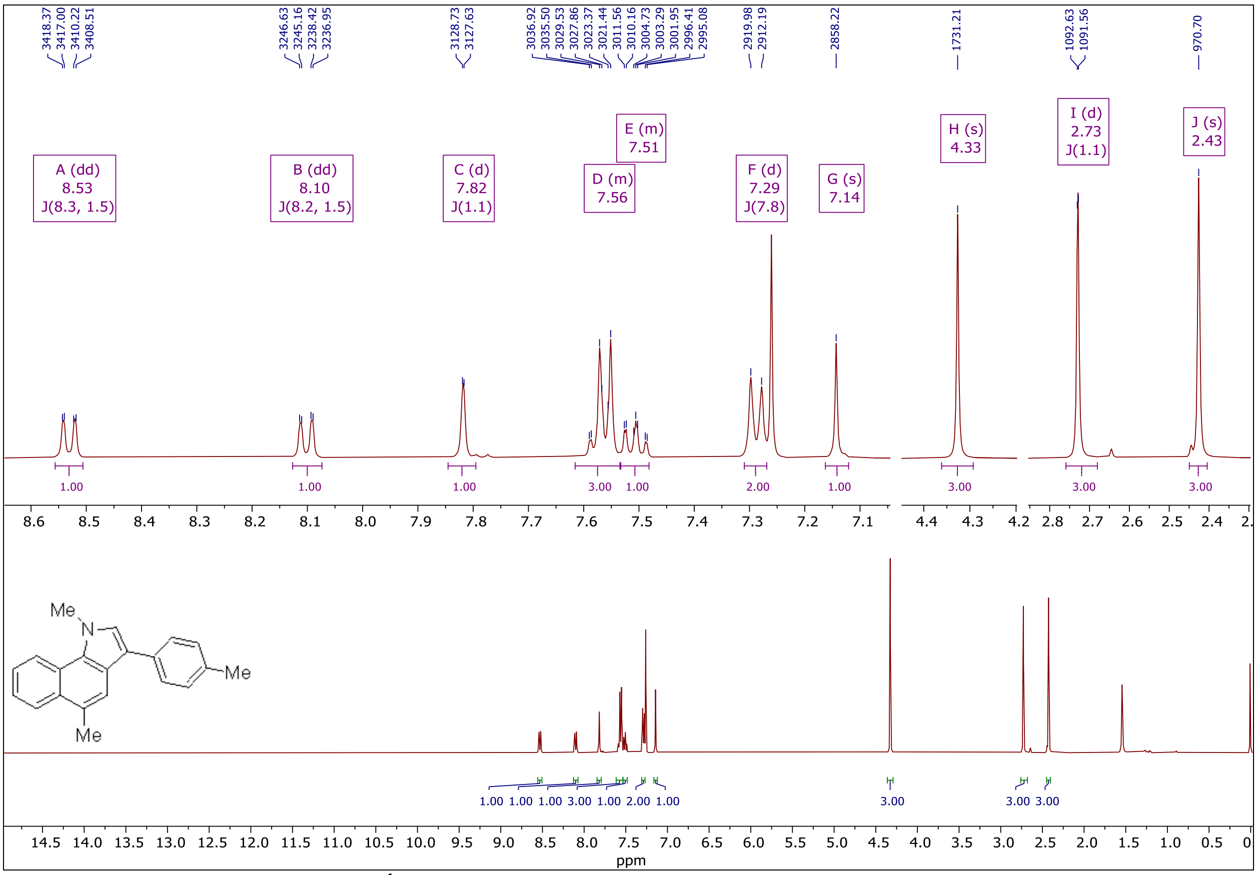

Fig. S81. ^{1}H NMR spectrum of compound **14a** (400 MHz, $CDCl_3$).

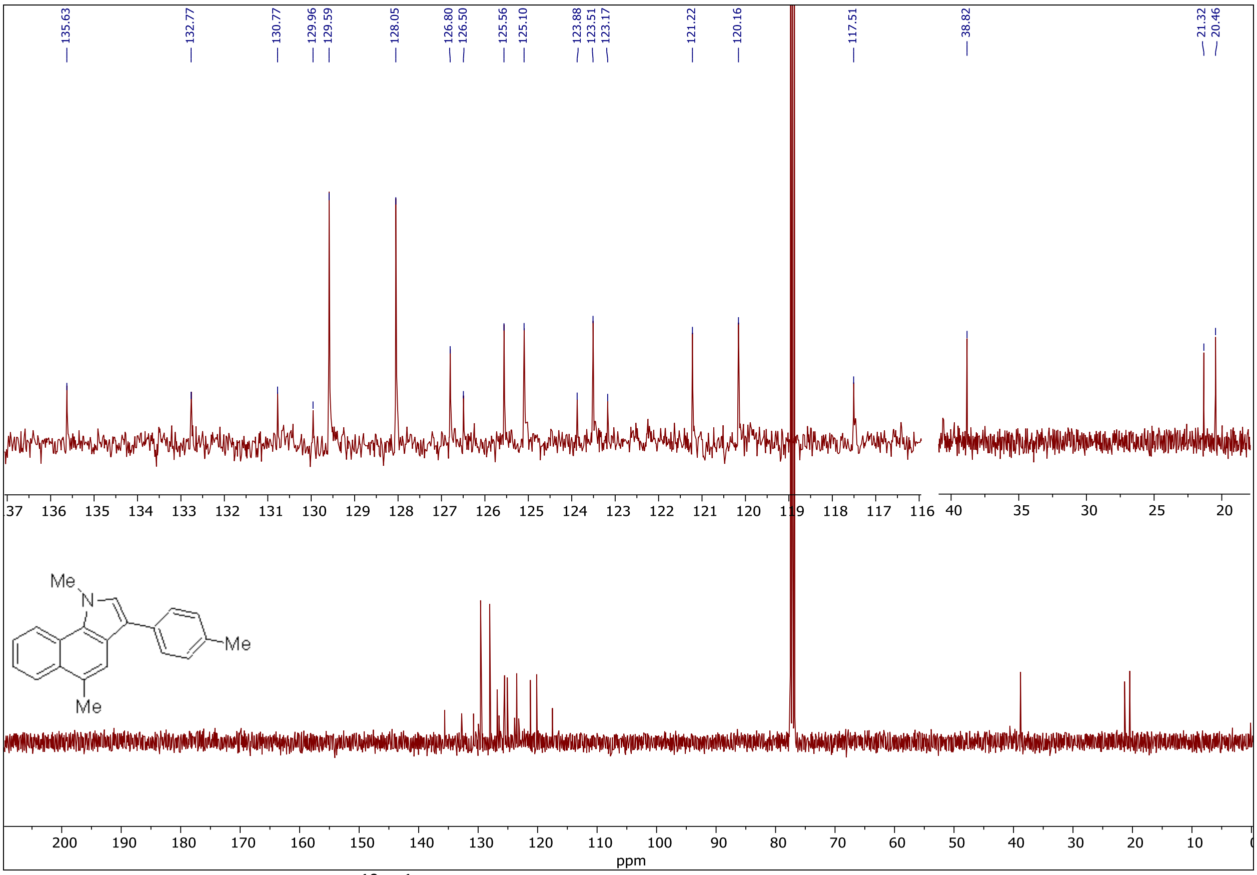

Fig. S82. ^{13}C{^{1}H} NMR spectrum of compound **14a** (100 MHz, $CDCl_3$).

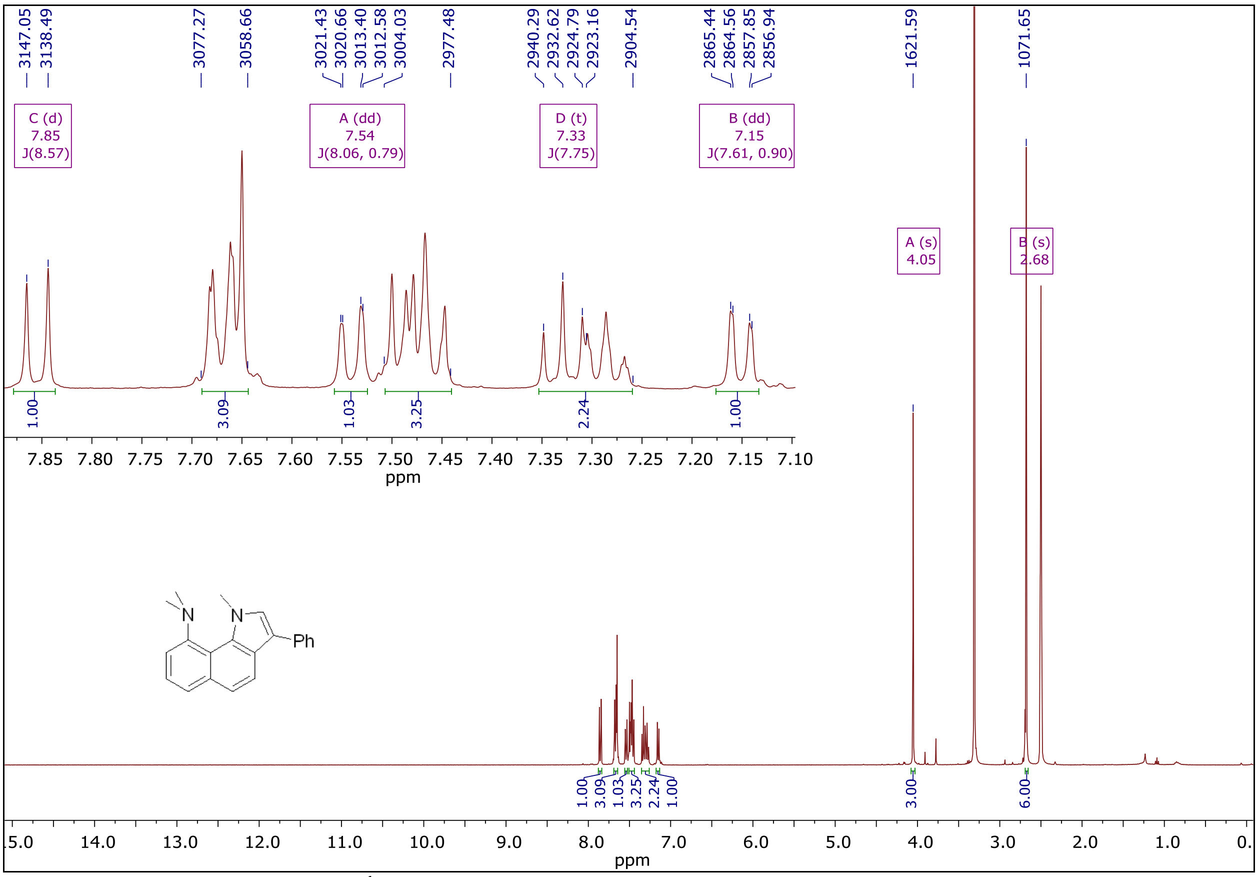

Fig. S83. ^{1}H NMR spectrum of compound **14g** (400 MHz, DMSO-*d6*).

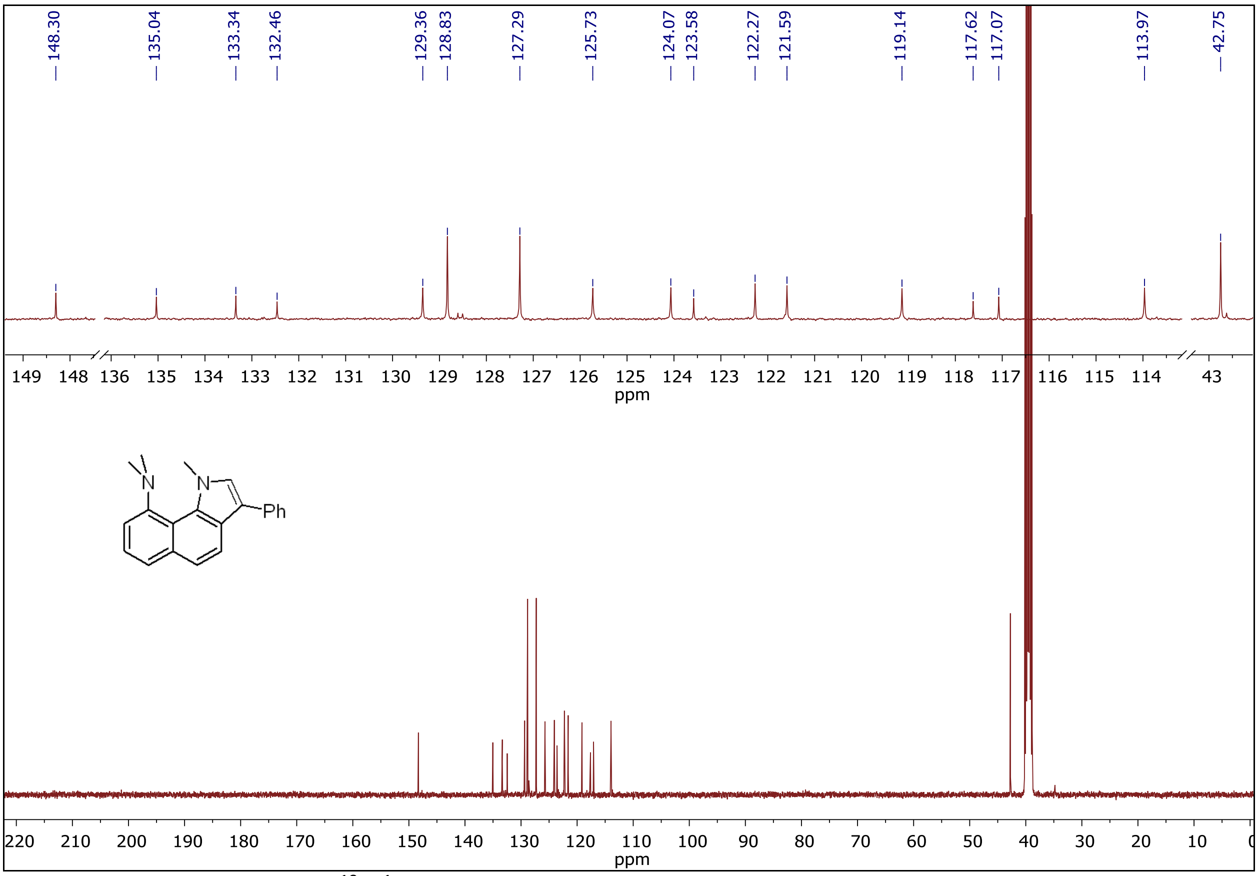

Fig. S84. ^{13}C{^{1}H} NMR spectrum of compound **14g** (100 MHz, DMSO-*d6*).

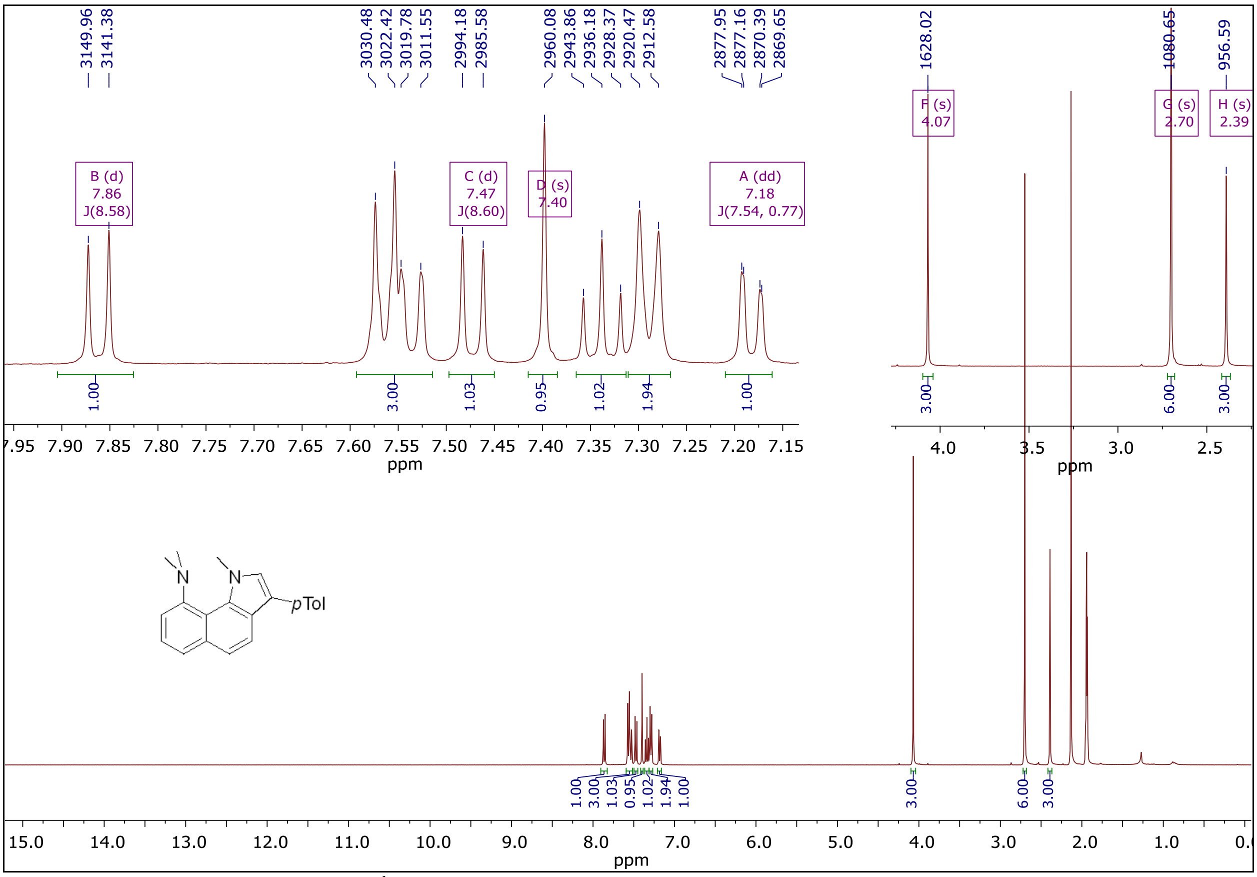

Fig. S85. ^{1}H NMR spectrum of compound **14h** (400 MHz, CD_3CN).

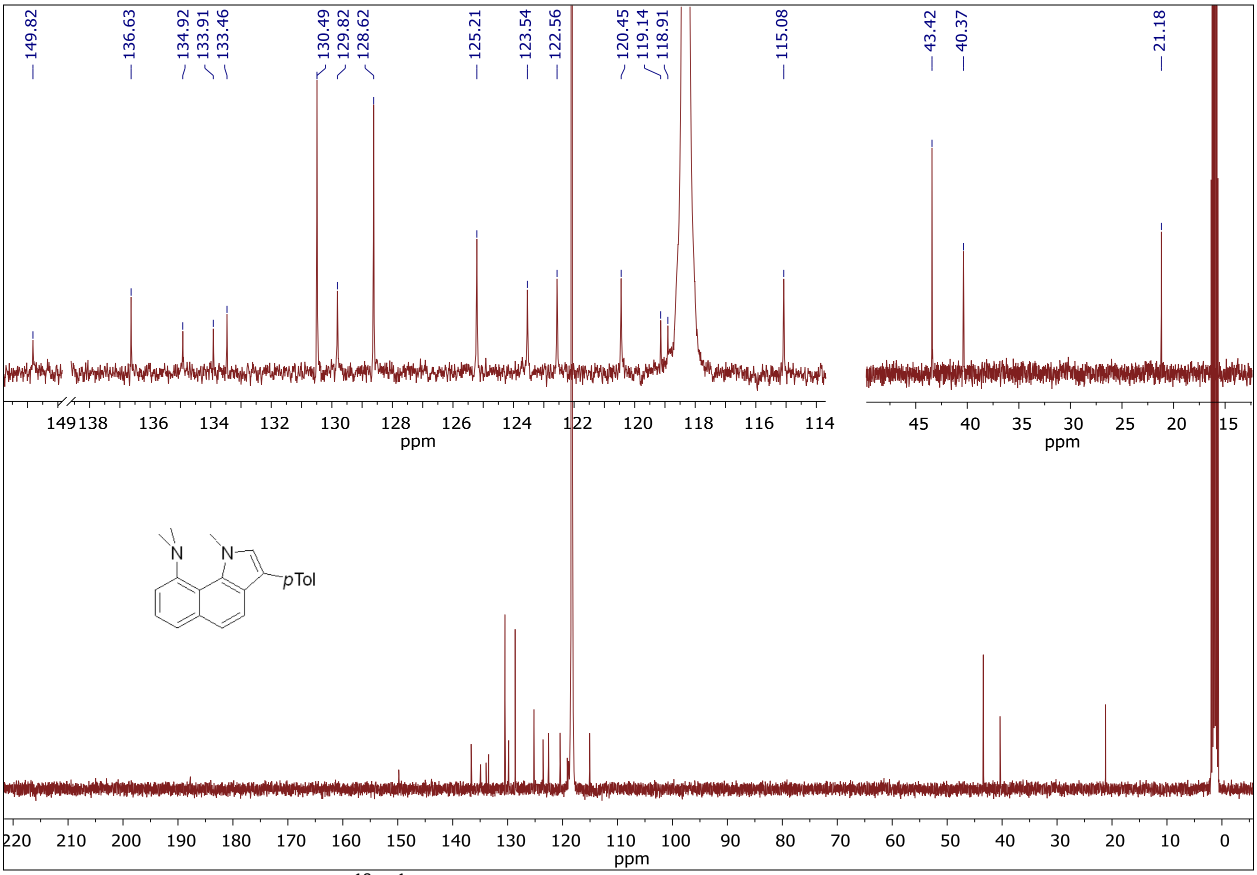

Fig. S86. $^{13}C\{^{1}H\}$ NMR spectrum of compound **14h** (100 MHz, CD_3CN).

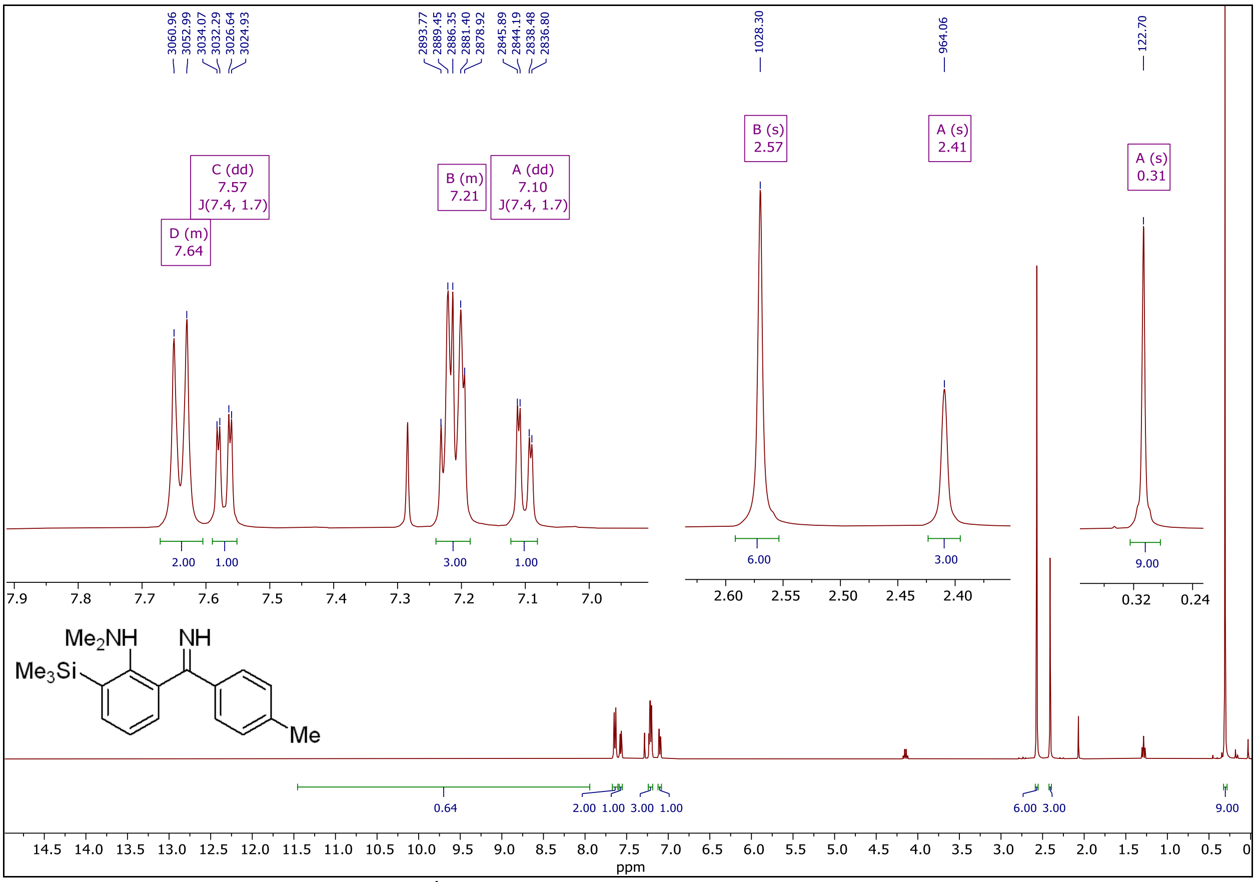

Fig. S87. ^{1}H NMR spectrum of compound **25b** (400 MHz, $CDCl_3$).

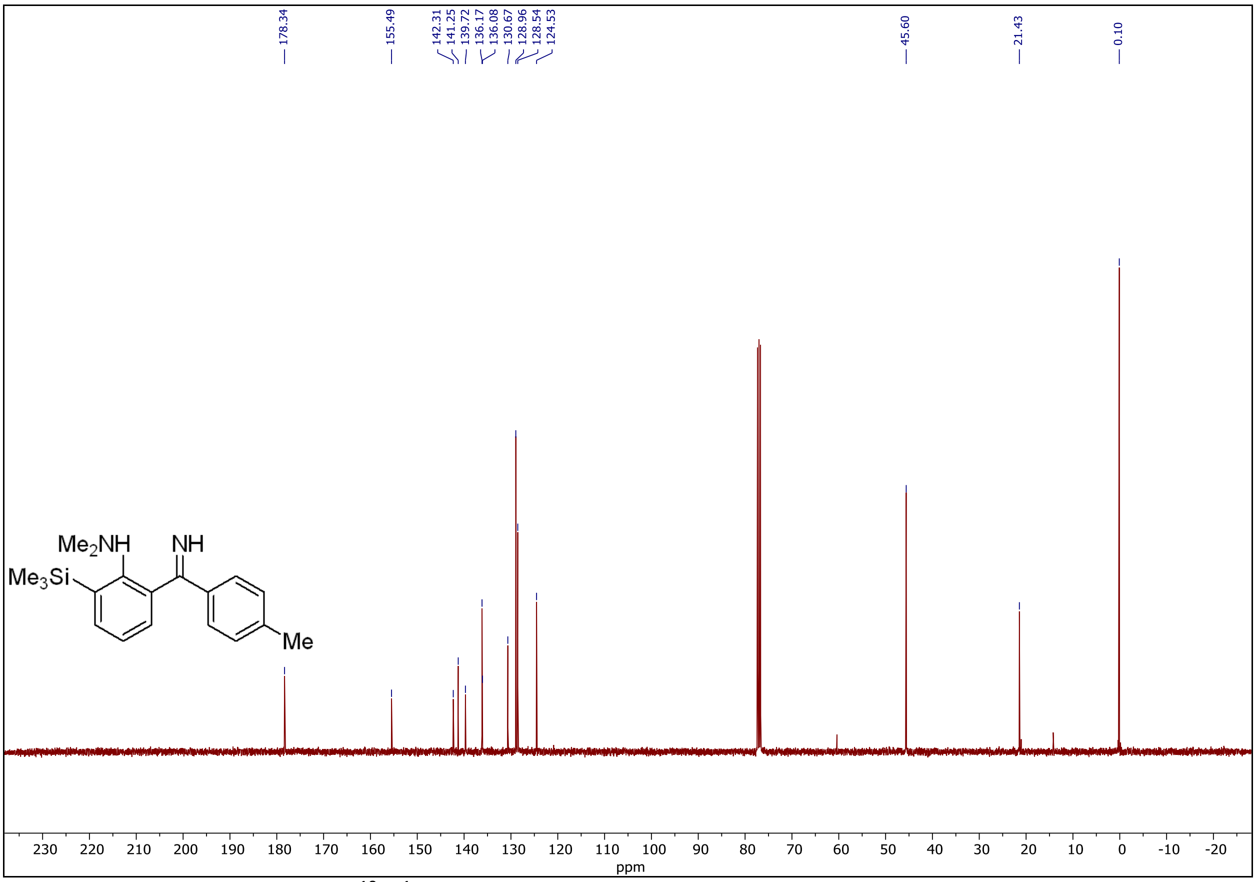

Fig. S88. $^{13}C\{^{1}H\}$ NMR spectrum of compound **25b** (100 MHz, $CDCl_3$).

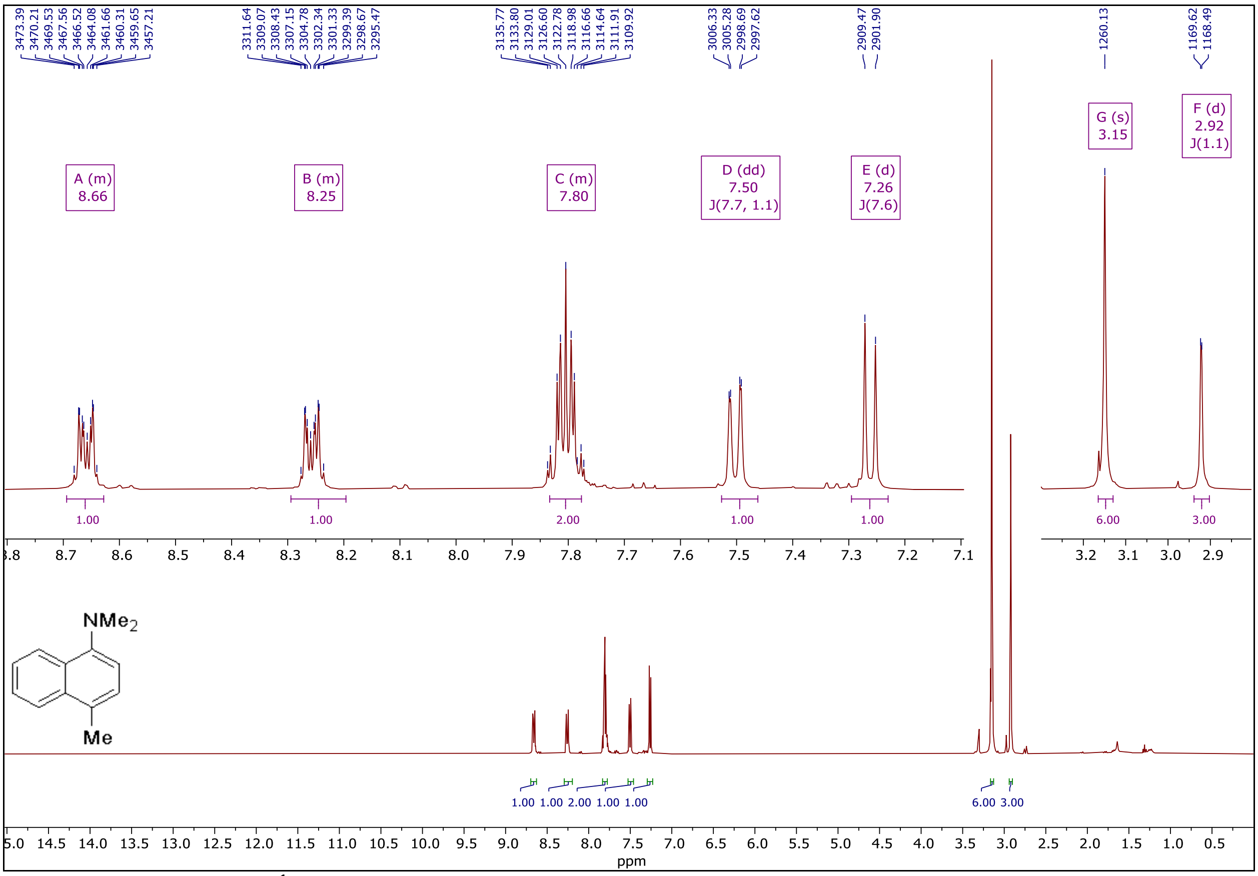

Fig. S89. ^{1}H NMR spectrum of *N*,*N*,4-trimethylnaphthalen-1-amine (400 MHz, $CDCl_3$).

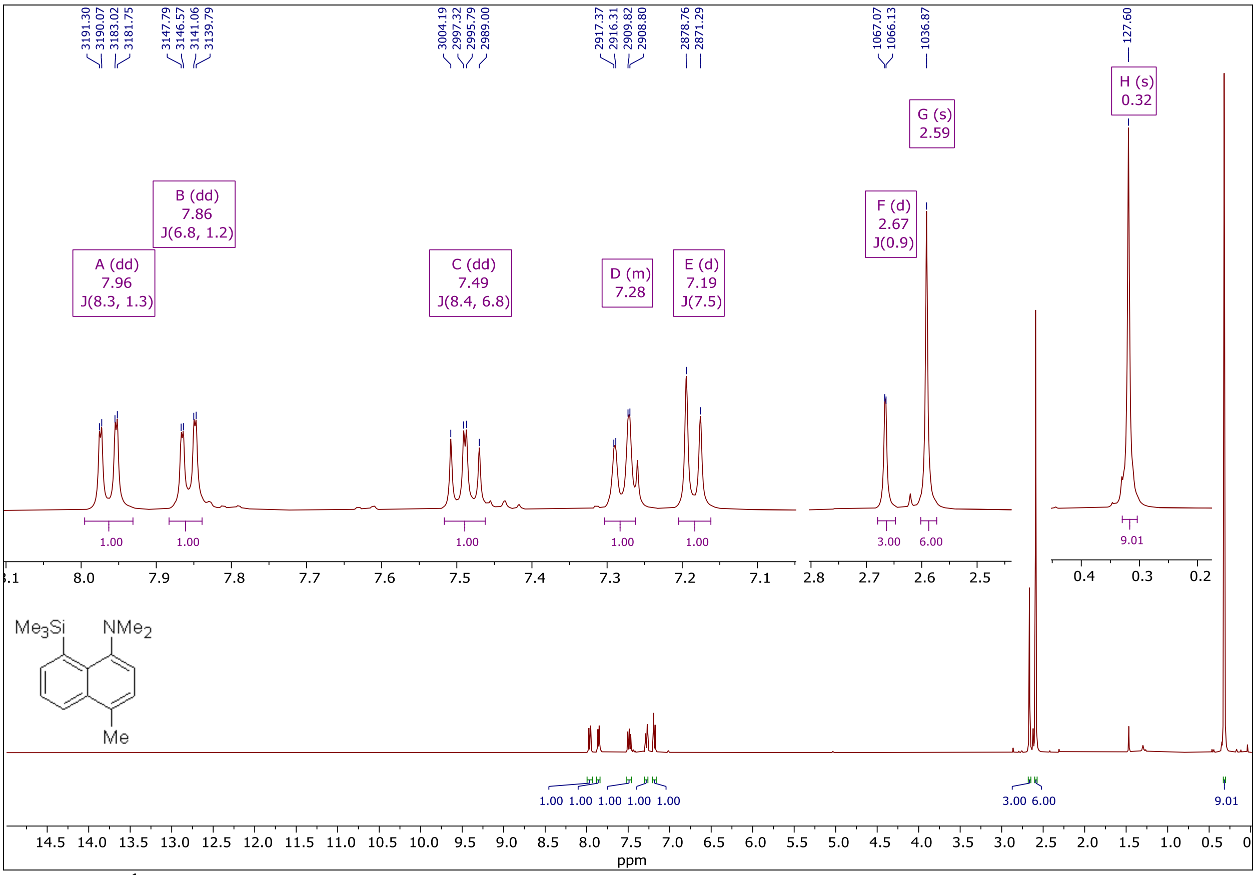

Fig. S90. ^{1}H NMR spectrum of compound *N*,*N*,4-trimethyl-8-(trimethylsilyl)naphthalen-1-amine (400 MHz, $CDCl_3$).

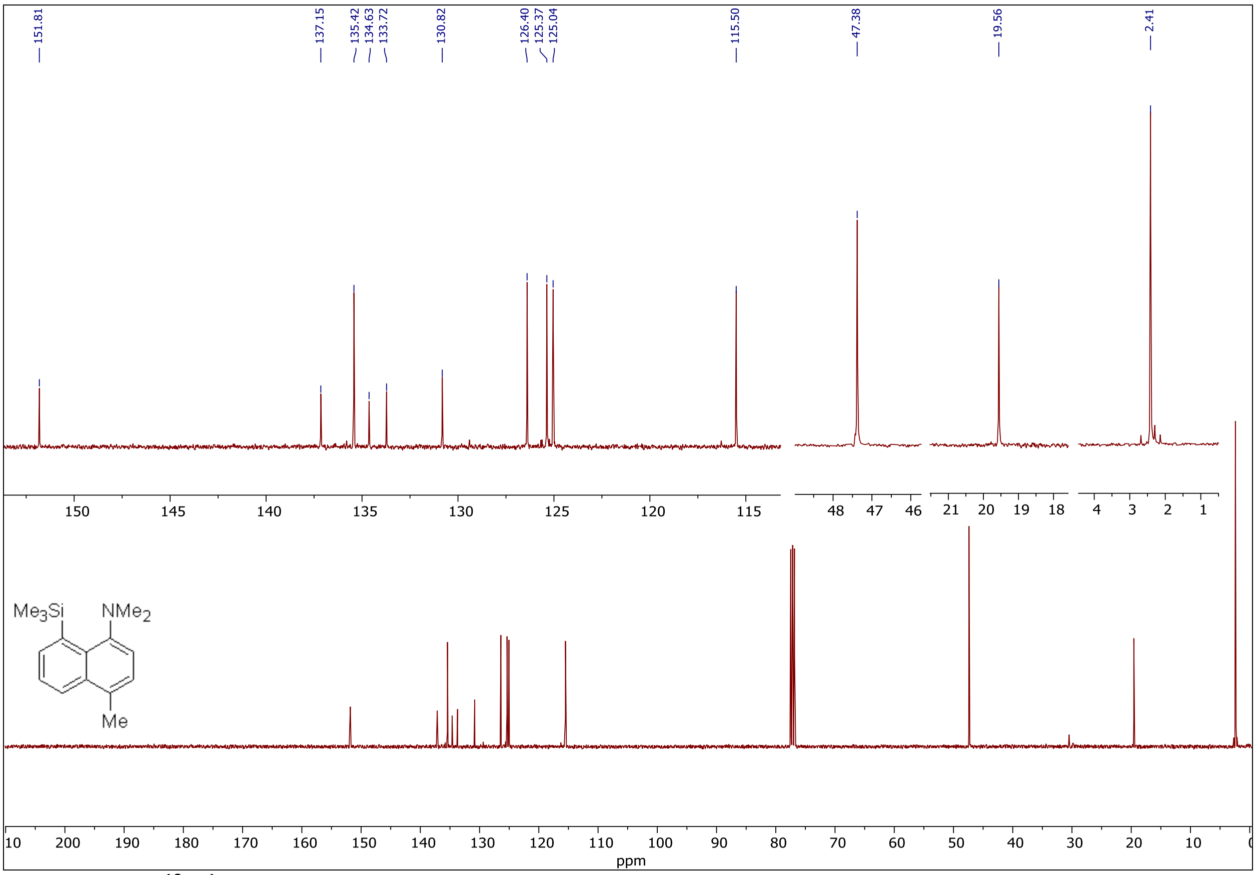

Fig. S91. $^{13}C\{^{1}H\}$ NMR spectrum of *N*,*N*,4-trimethyl-8-(trimethylsilyl)naphthalen-1-amine (100 MHz, $CDCl_3$).

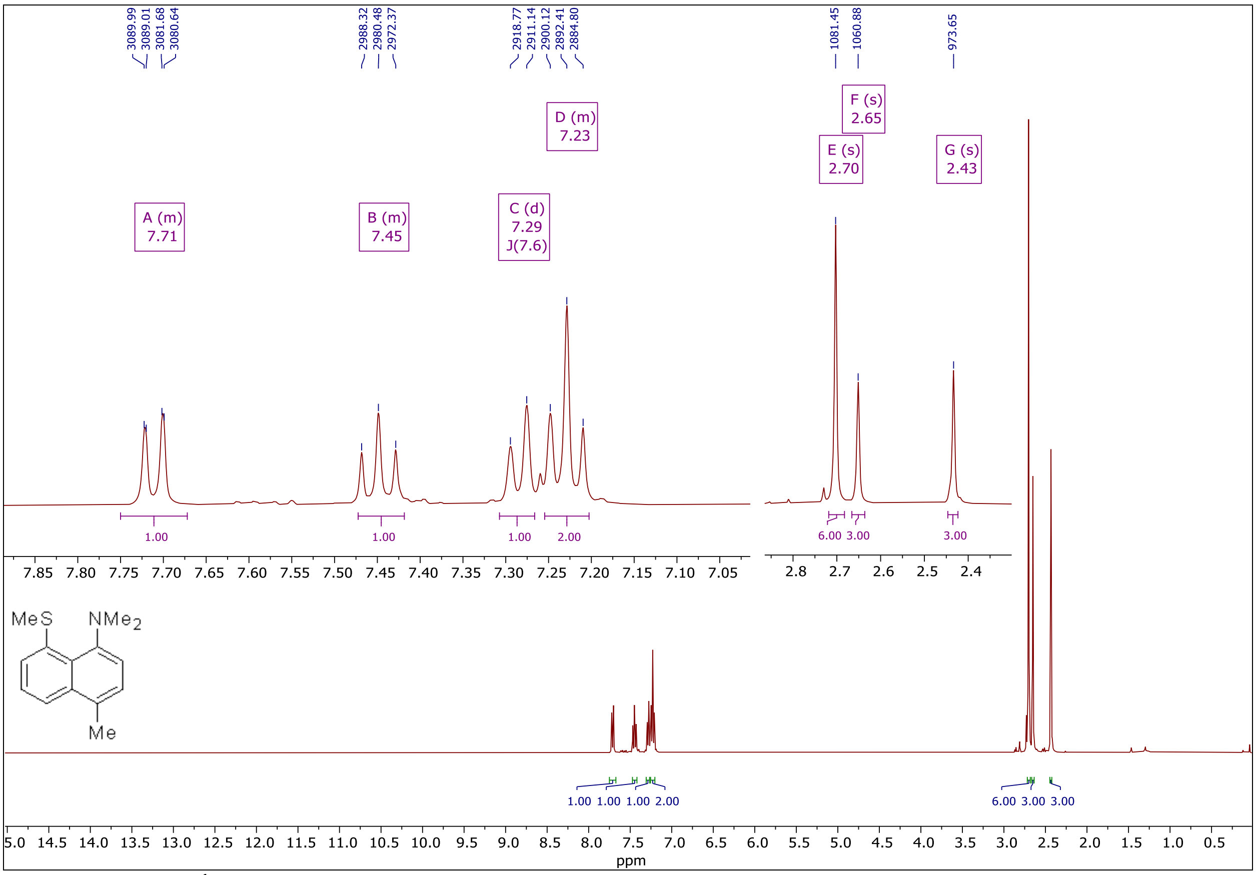

Fig. S92. ^{1}H NMR spectrum of *N*,*N*,4-trimethyl-8-(methylthio)naphthalen-1-amine (400 MHz, $CDCl_3$).

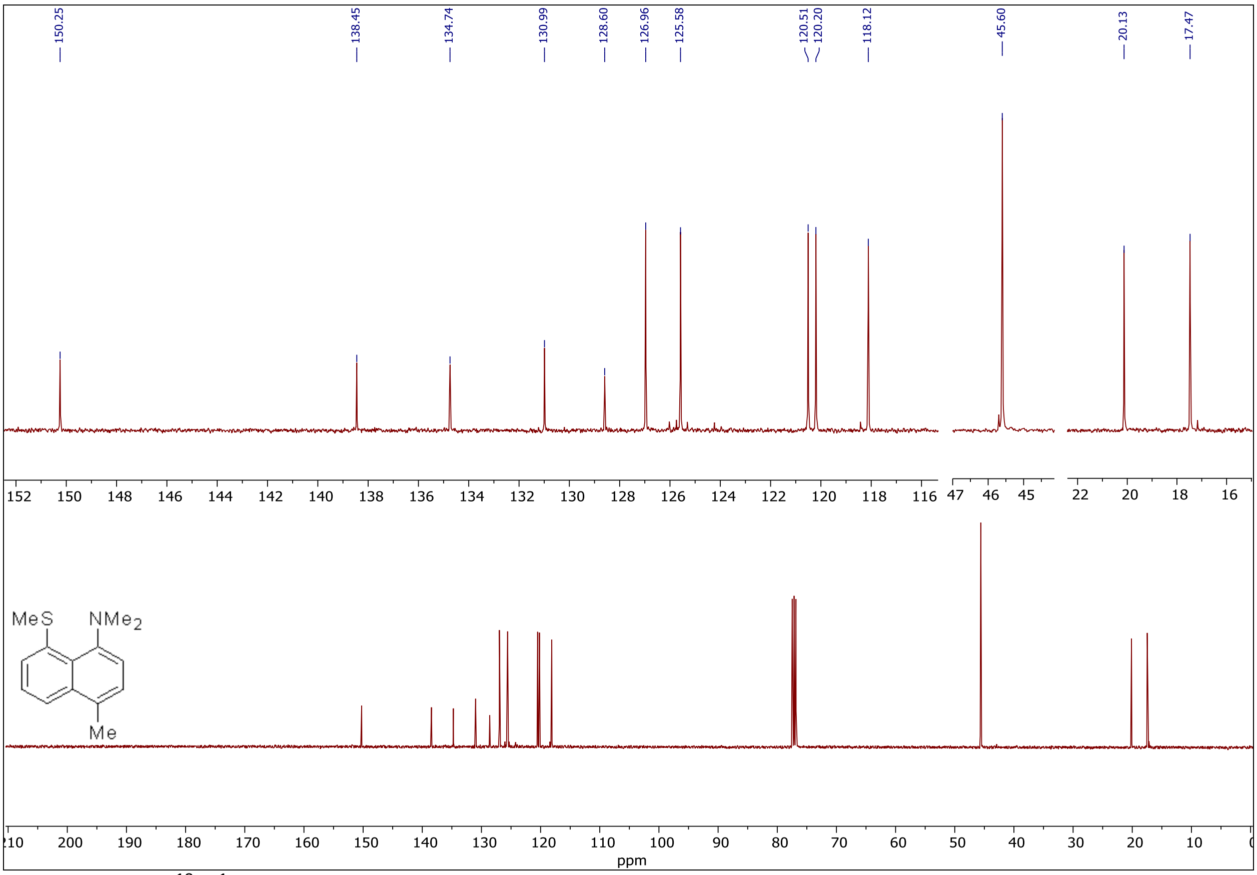

Fig. S93. $^{13}C\{^{1}H\}$ NMR spectrum of *N*,*N*,4-trimethyl-8-(methylthio)naphthalen-1-amine (100 MHz, $CDCl_3$).

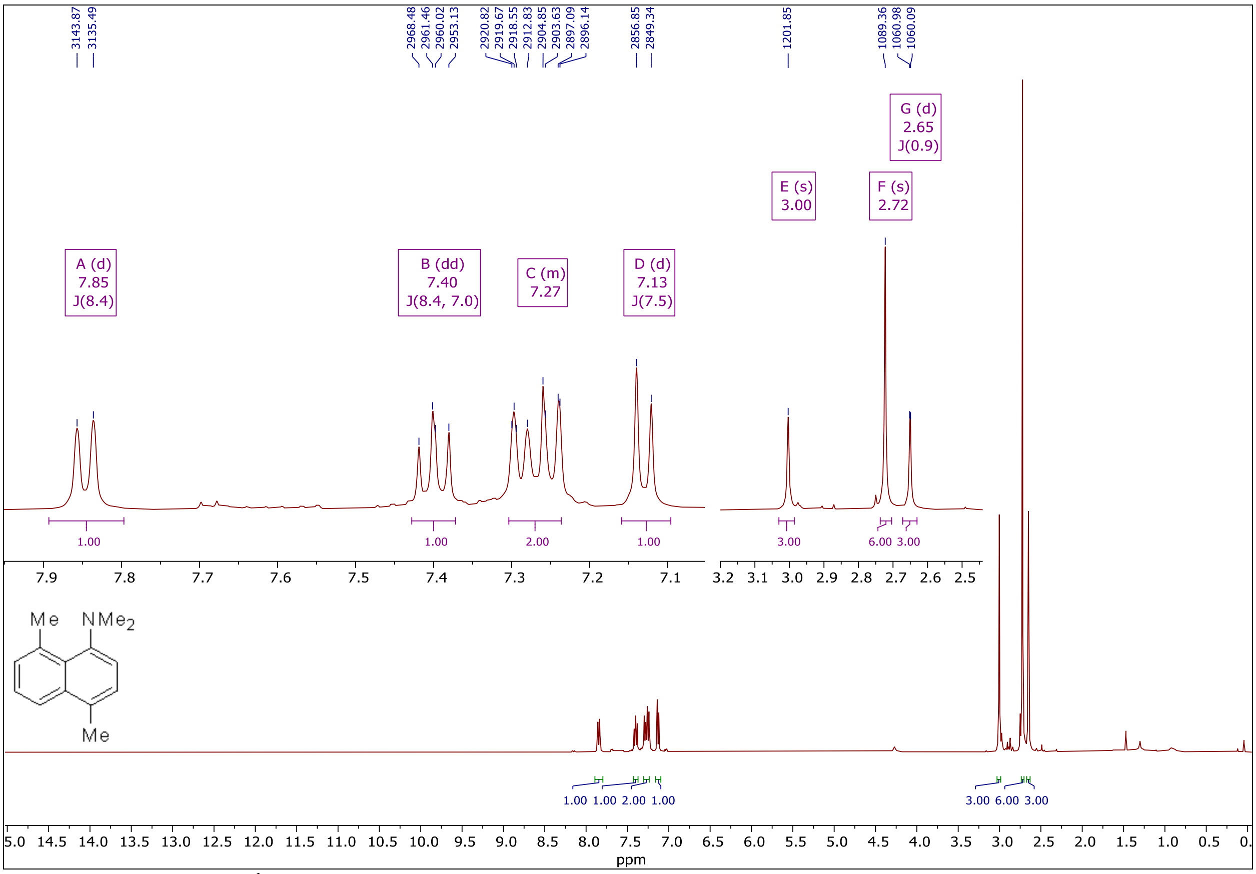

Fig. S94. ^{1}H NMR spectrum of *N*,*N*,4,8-tetramethylnaphthalen-1-amine (400 MHz, $CDCl_3$).

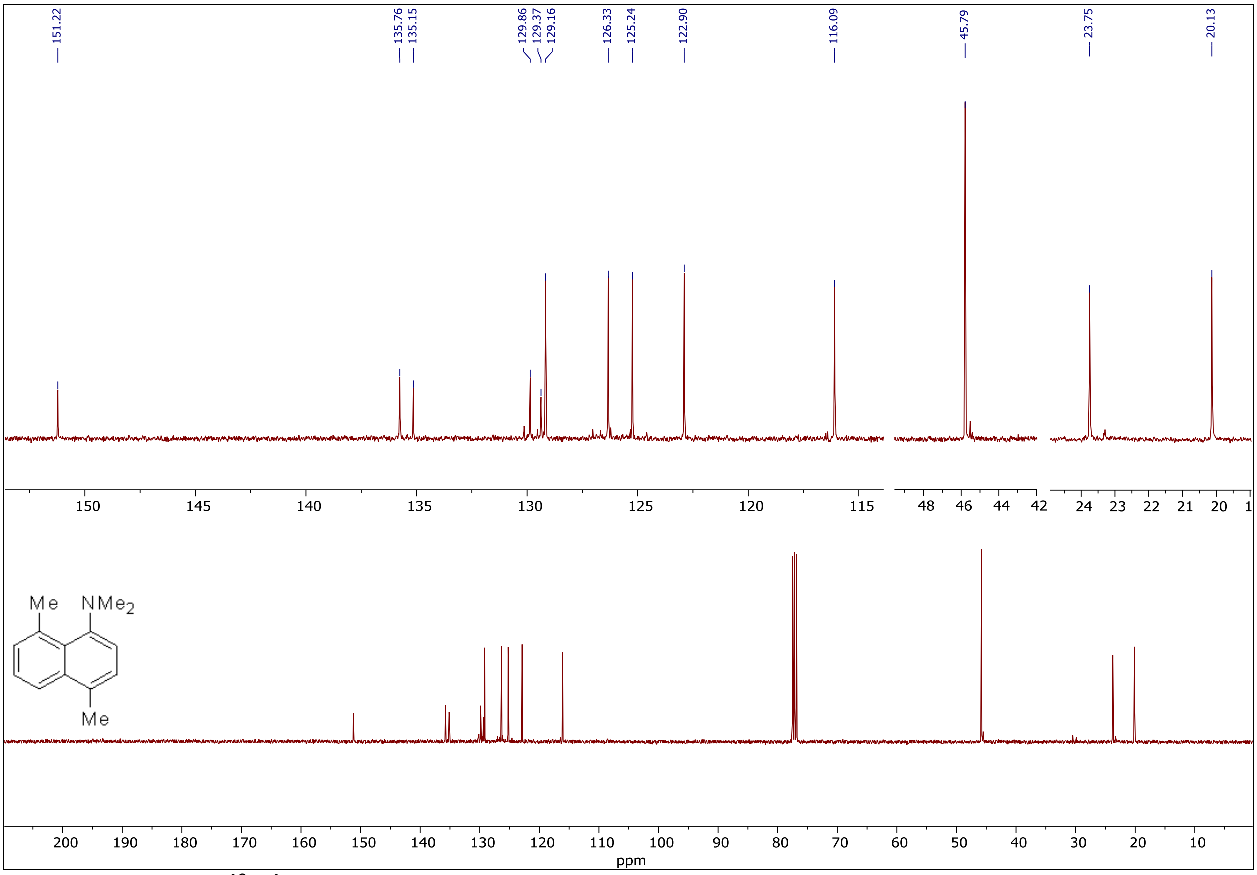

Fig. S95. $^{13}C\{^1H\}$ NMR spectrum of *N*,*N*,4,8-tetramethylnaphthalen-1-amine (100 MHz, $CDCl_3$).

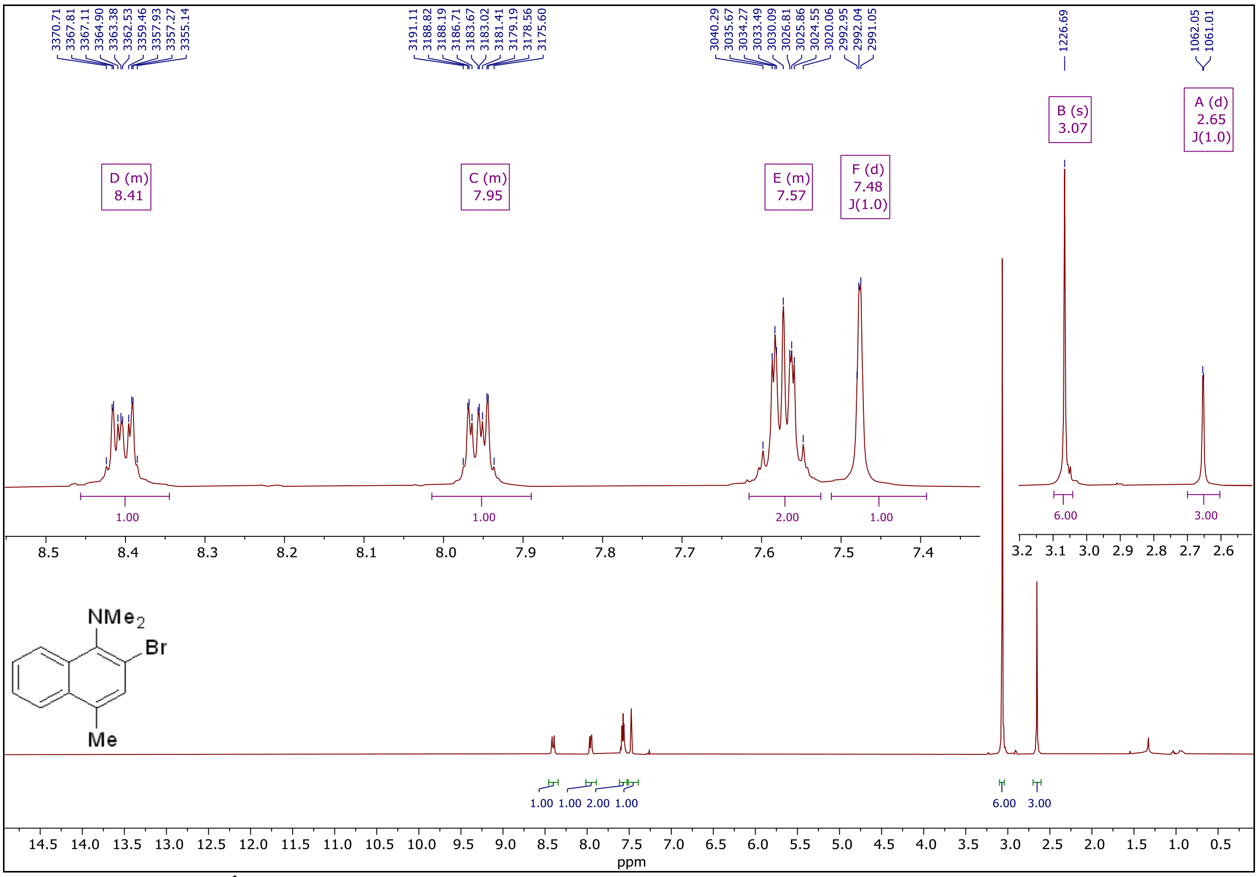

Fig. S96. ^1H NMR spectrum of 2-bromo-*N*,*N*,4-trimethylnaphthalen-1-amine (400 MHz, $CDCl_3$).

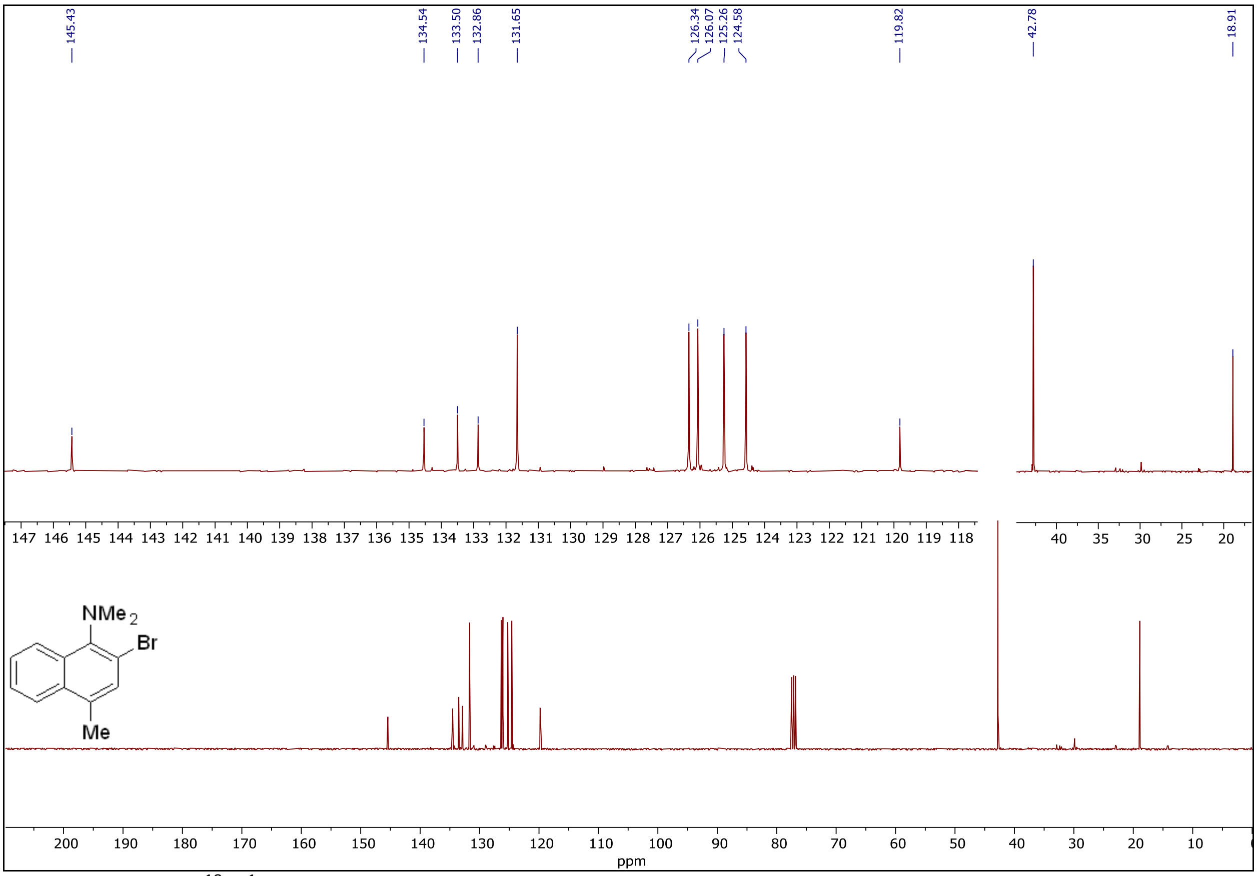

Fig. S97. $^{13}C\{^{1}H\}$ NMR spectrum of 2-bromo-*N*,*N*,4-trimethylnaphthalen-1-amine (100 MHz, $CDCl_3$).

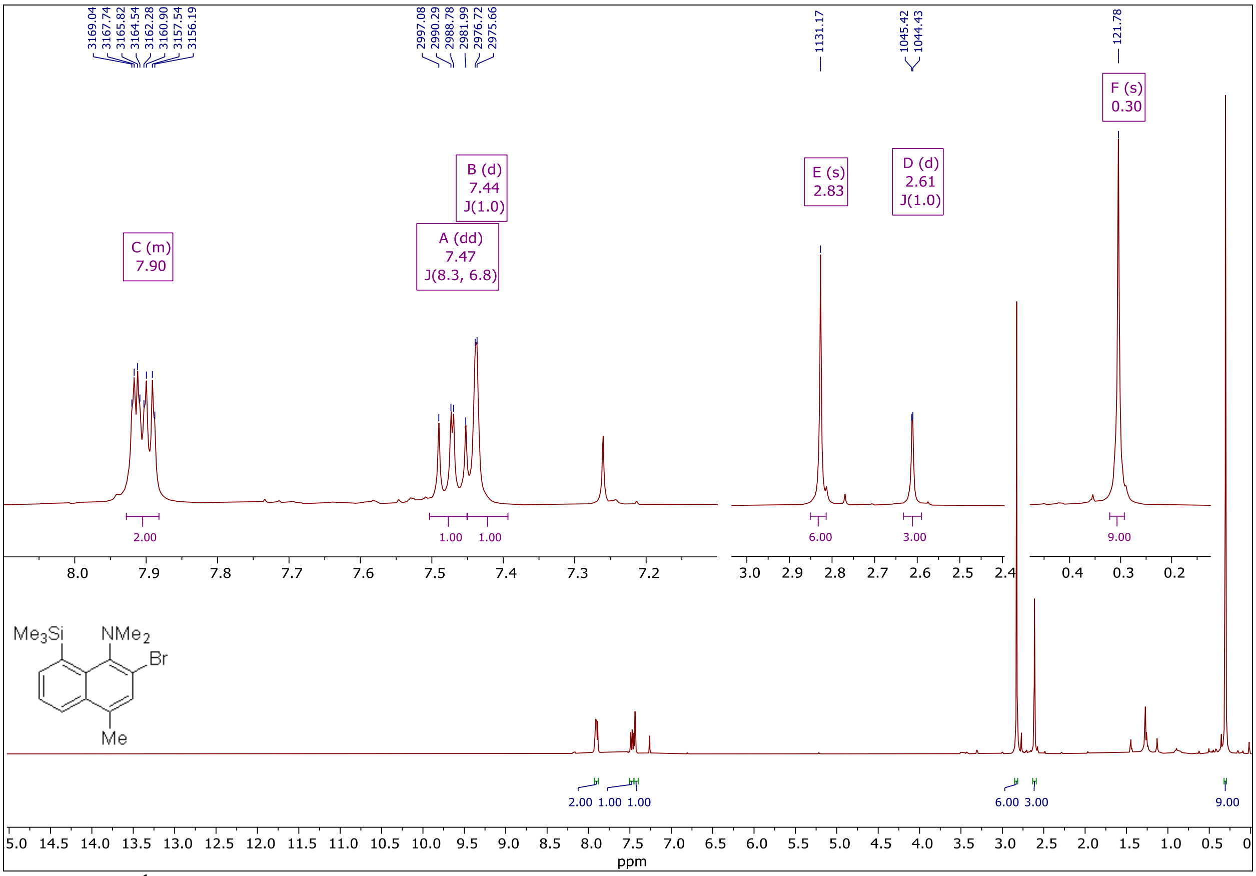

Fig. S98. ^{1}H NMR spectrum of 2-bromo-*N*,*N*,4-trimethyl-8-(trimethylsilyl)naphthalen-1-amine (400 MHz, $CDCl_3$).

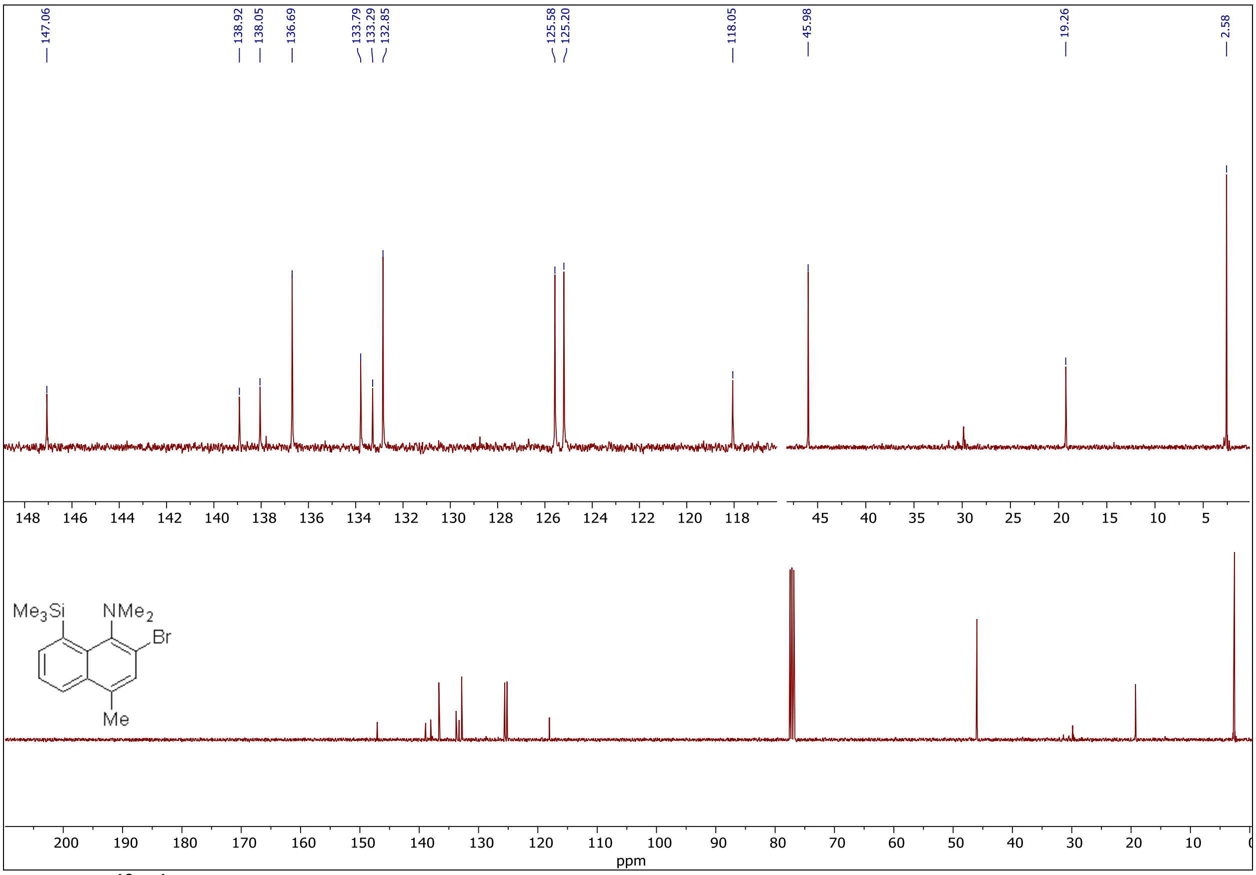

Fig. S99. $^{13}C\{^1H\}$ NMR spectrum of 2-bromo-*N*,*N*,4-trimethyl-8-(trimethylsilyl)naphthalen-1-amine (100 MHz, $CDCl_3$).

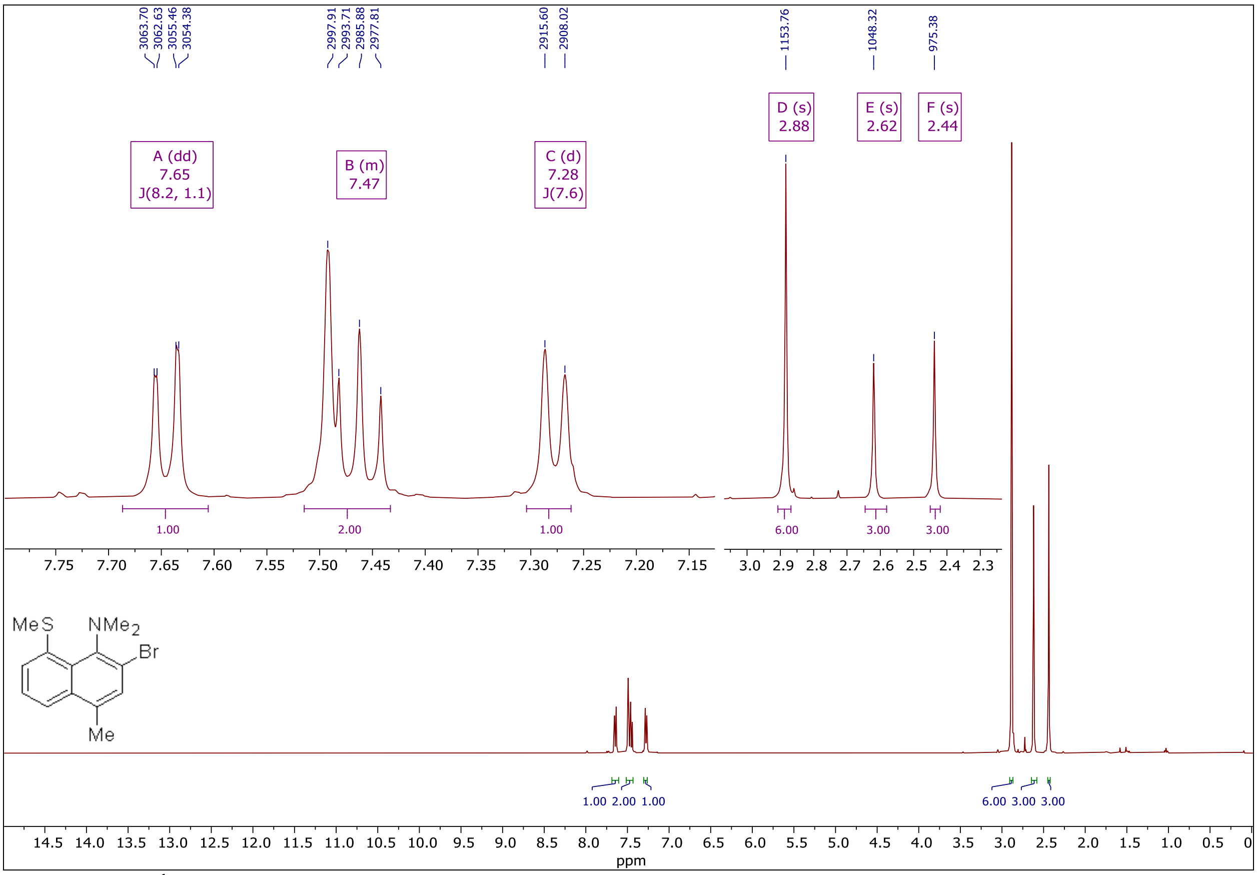

Fig. S100. ^1H NMR spectrum of 2-bromo-*N*,*N*,4-trimethyl-8-(methylthio)naphthalen-1-amine (400 MHz, $CDCl_3$).

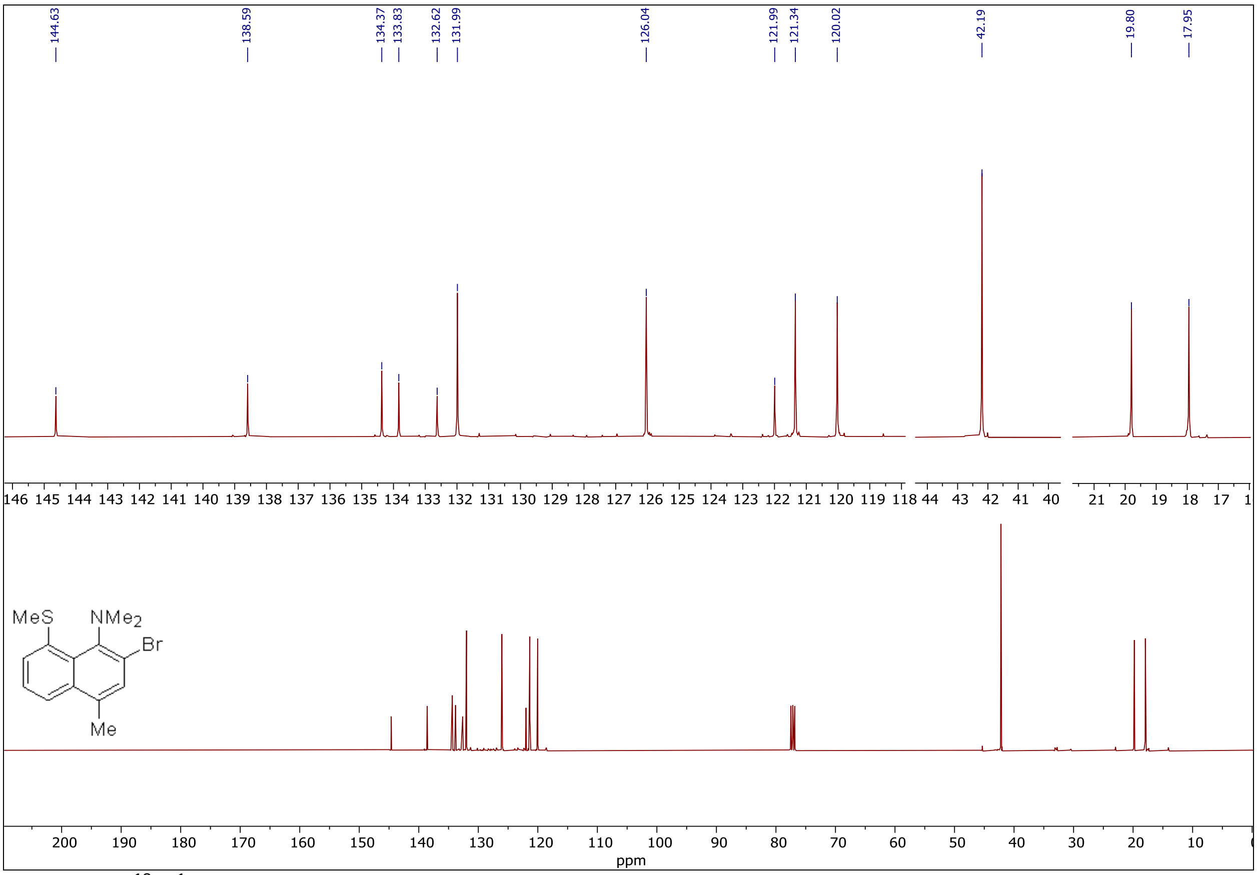

Fig. S101. $^{13}C\{^{1}H\}$ NMR spectrum of 2-bromo-*N*,*N*,4-trimethyl-8-(methylthio)naphthalen-1-amine (100 MHz, $CDCl_3$).

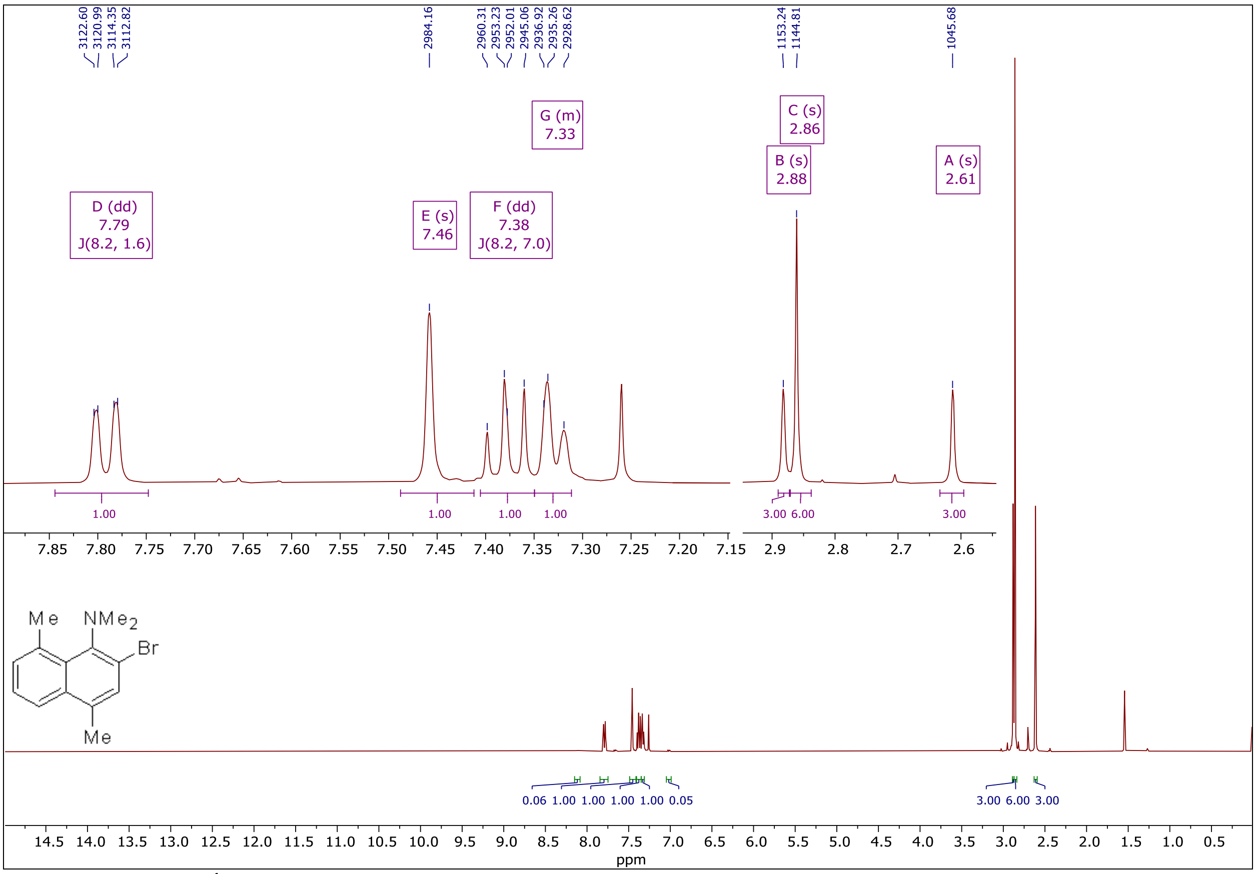

Fig. S102. ^{1}H NMR spectrum of 2-bromo-*N*,*N*,4,8-tetramethylnaphthalen-1-amine (400 MHz, $CDCl_3$).

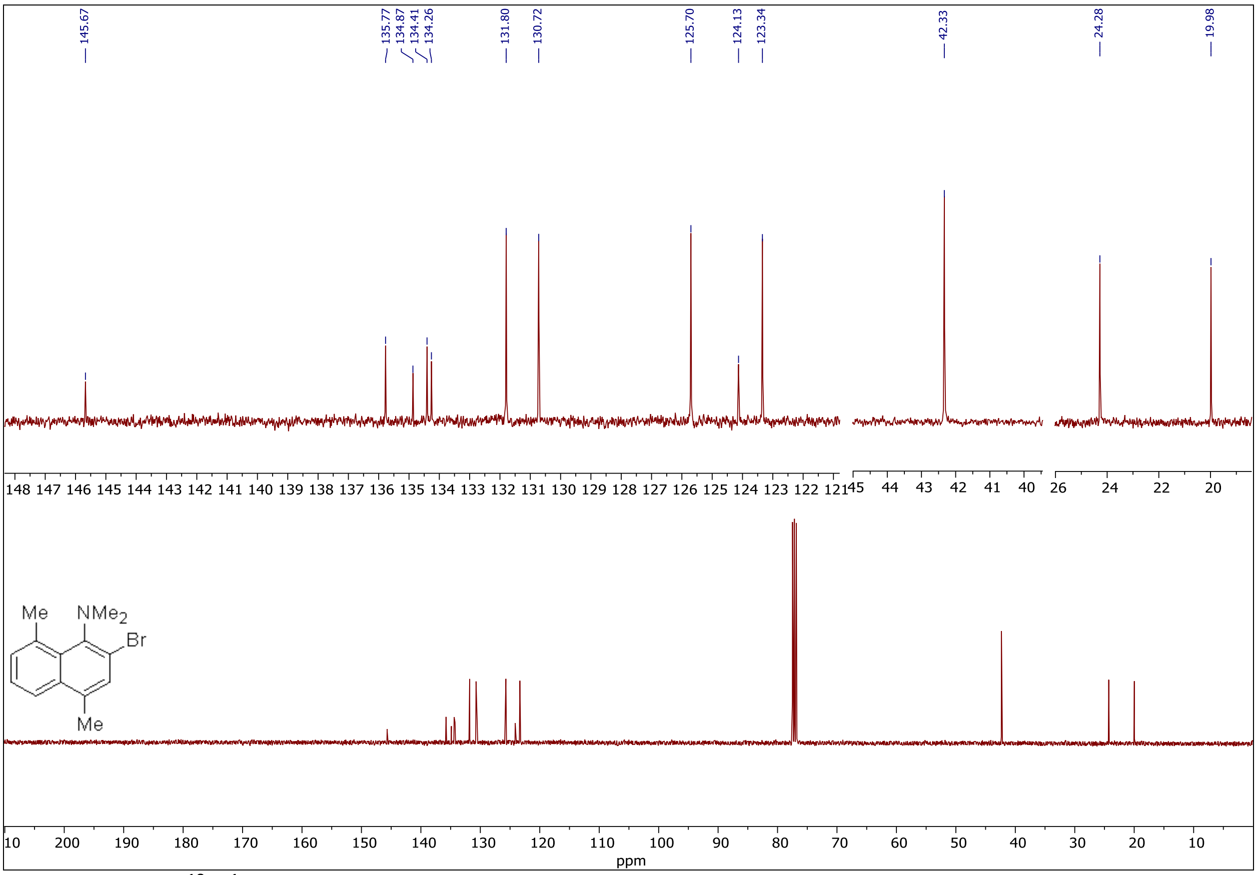

Fig. S103. $^{13}C\{^{1}H\}$ NMR spectrum of 2-bromo-*N*,*N*,4,8-tetramethylnaphthalen-1-amine (100 MHz, $CDCl_3$).

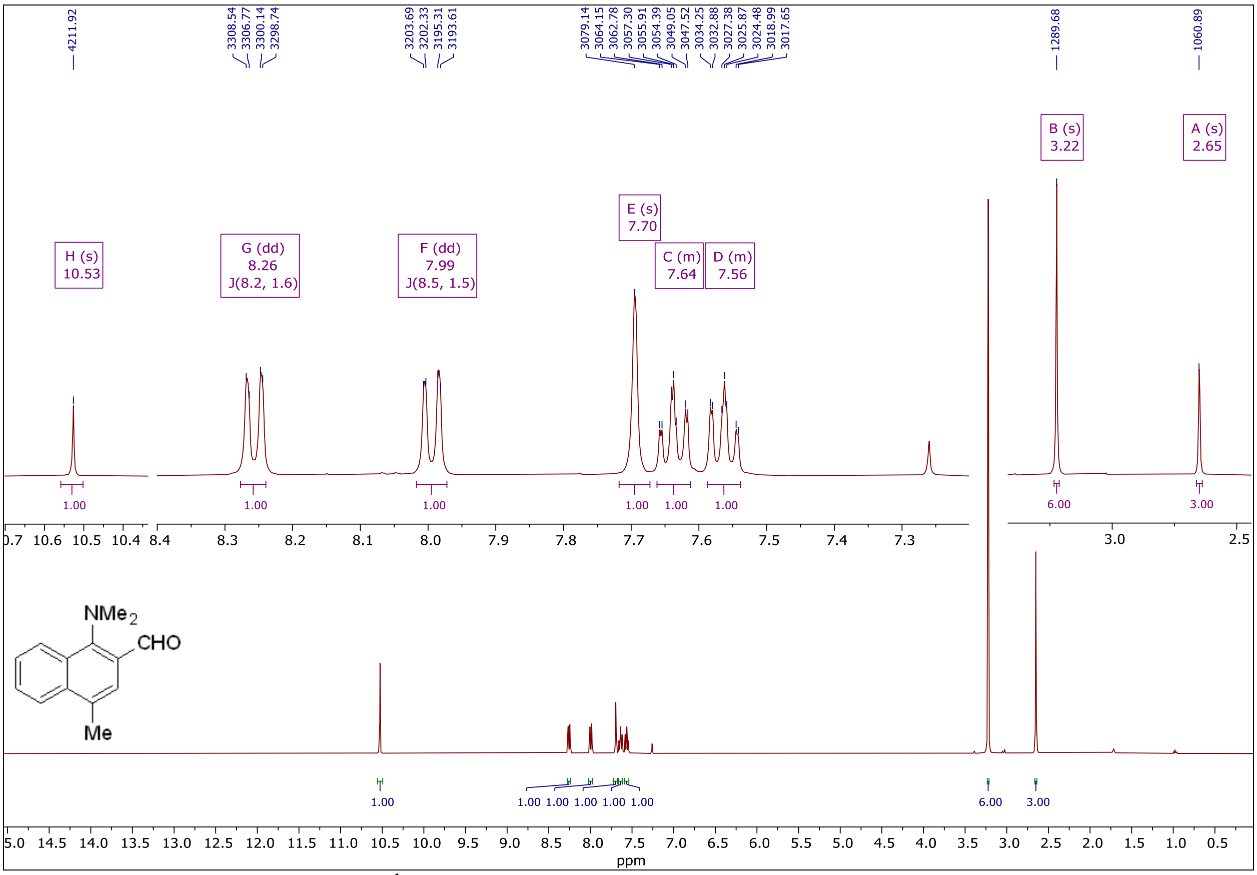

Fig. S104. ^{1}H NMR spectrum of compound **26a** (400 MHz, $CDCl_3$).

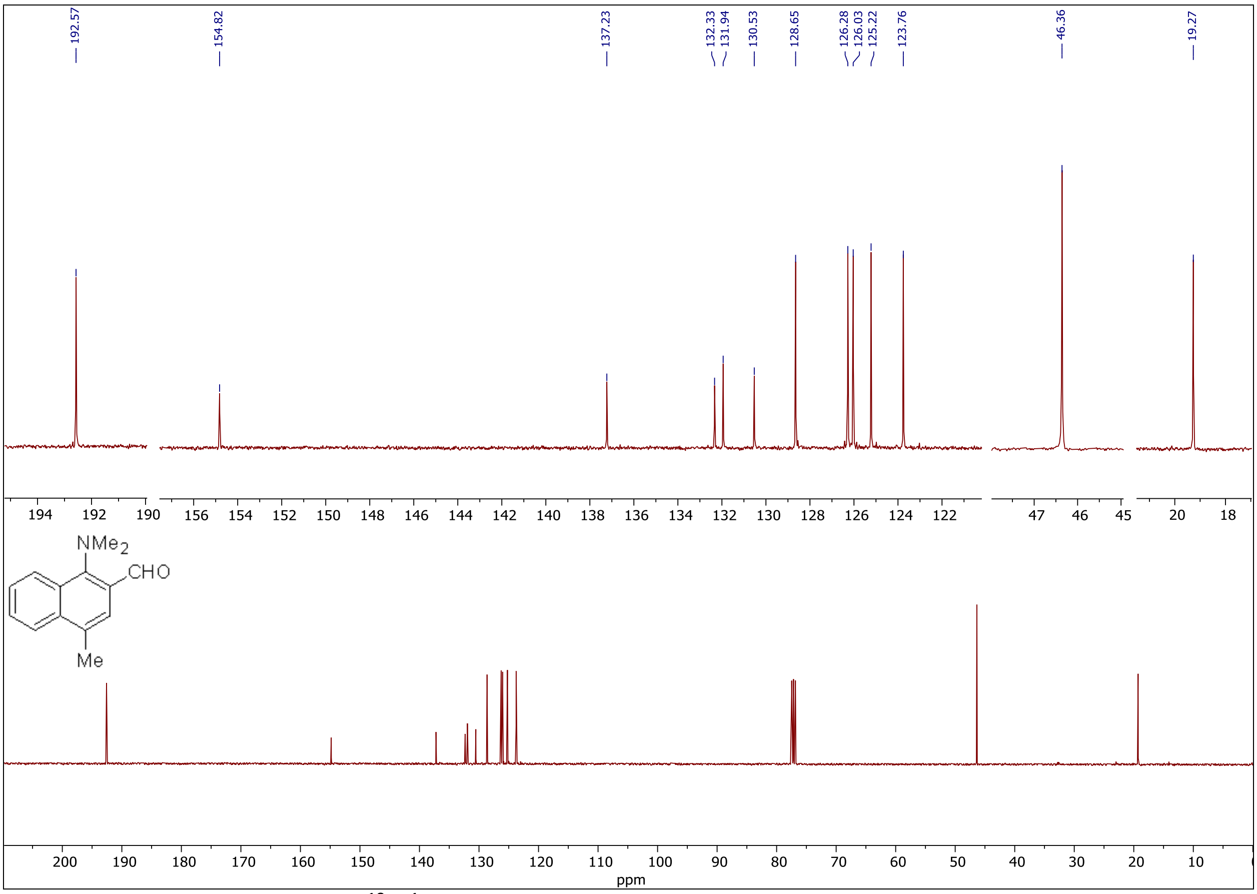

Fig. S105. $^{13}C\{^{1}H\}$ NMR spectrum of compound **26a** (100 MHz, $CDCl_3$).

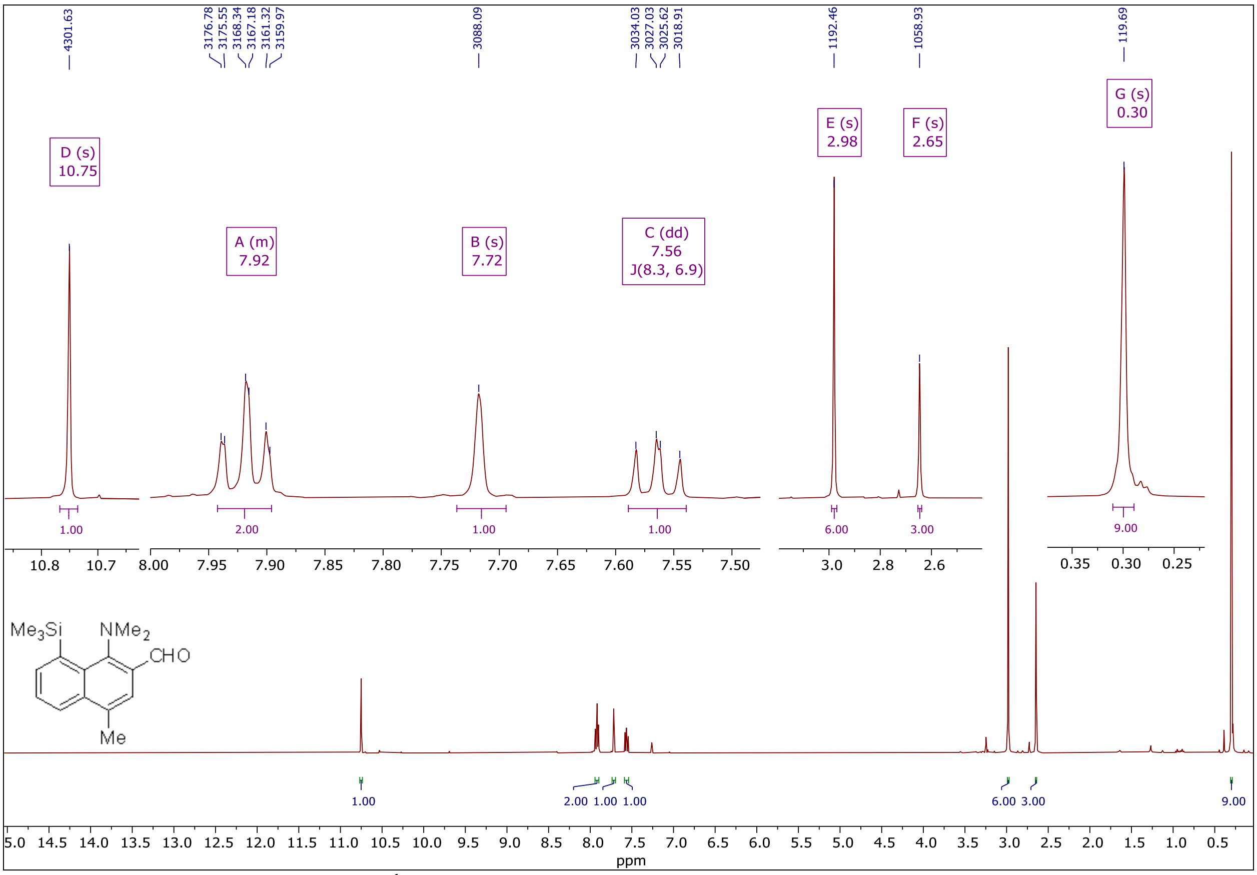

Fig. S106. ^{1}H NMR spectrum of compound **26b** (400 MHz, $CDCl_3$).

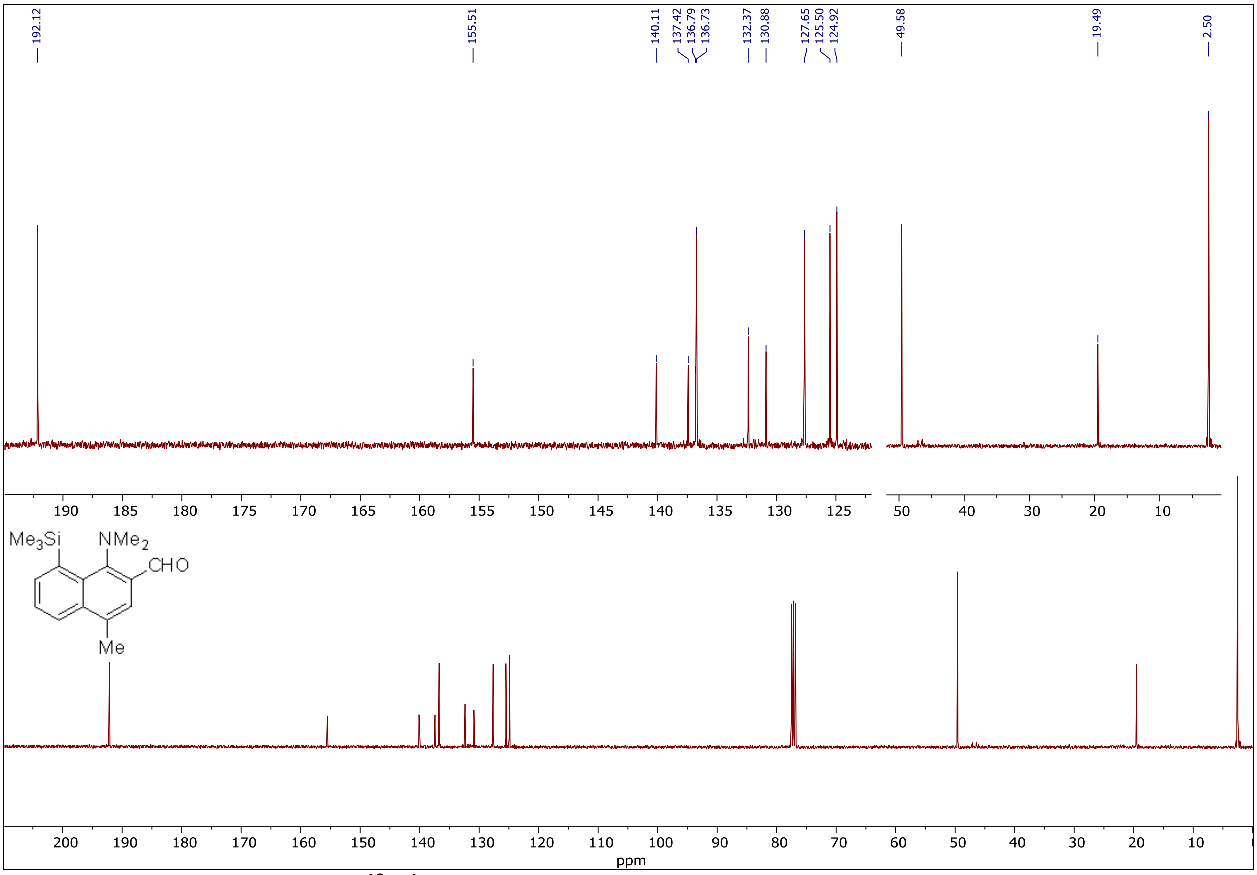

Fig. S107. $^{13}C\{^{1}H\}$ NMR spectrum of compound **26b** (100 MHz, $CDCl_3$).

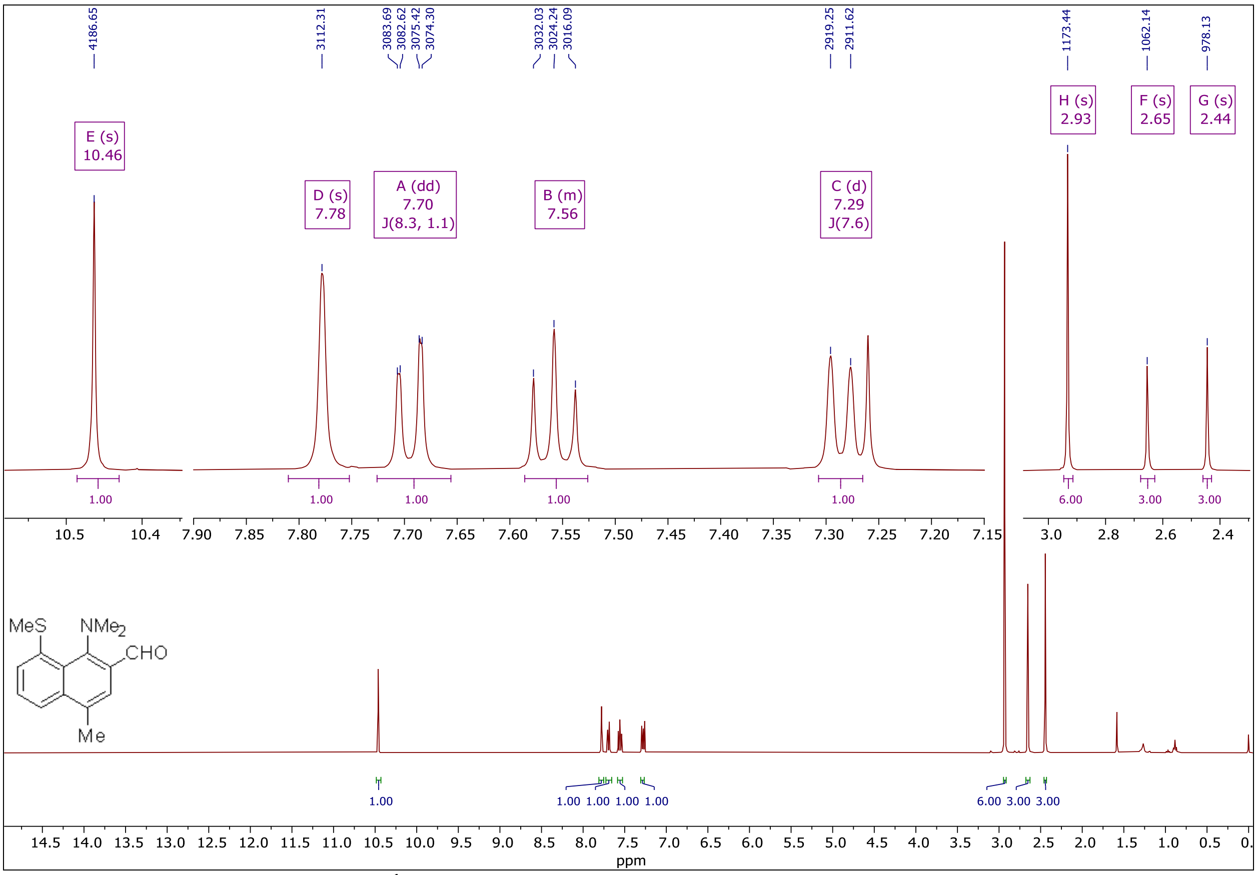

Fig. S108. ^{1}H NMR spectrum of compound **26d** (400 MHz, $CDCl_3$).

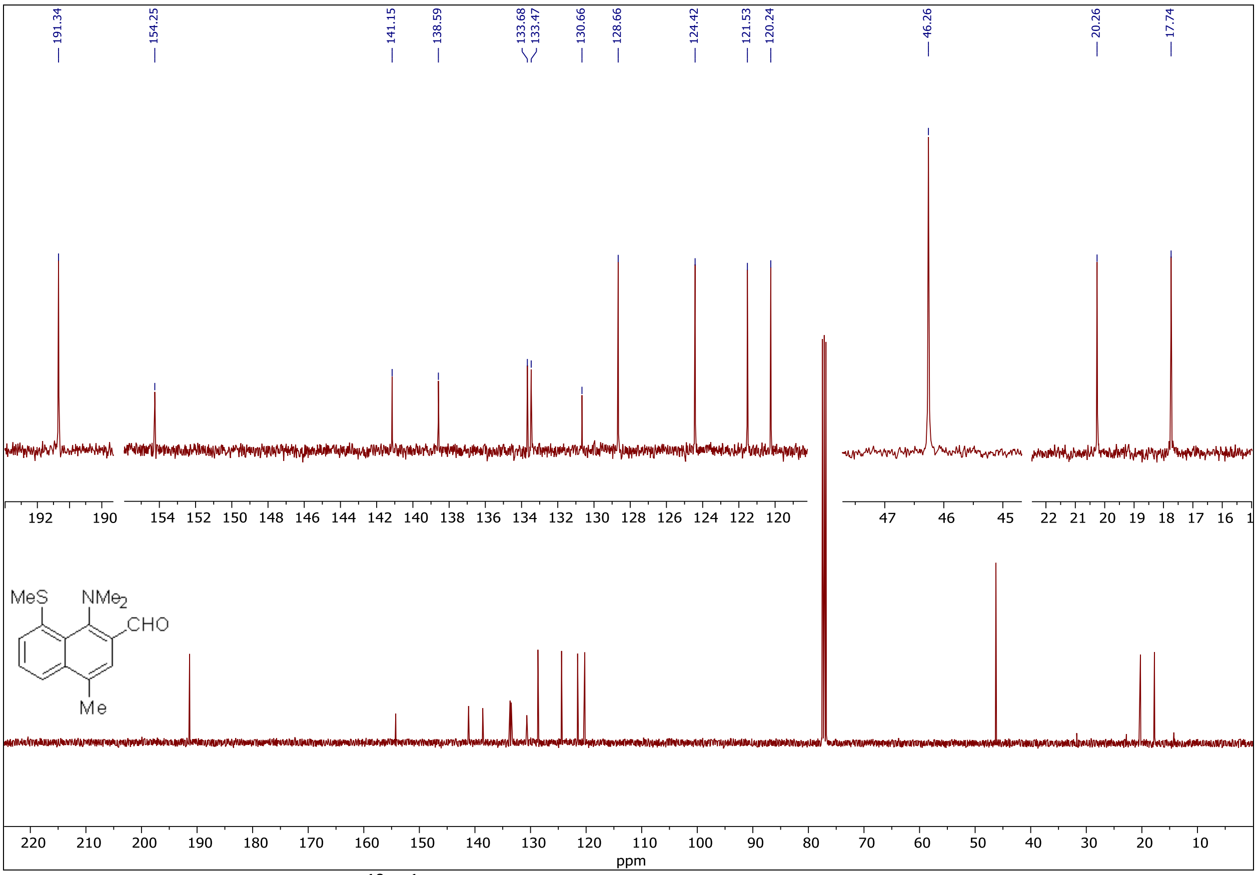

Fig. S109. $^{13}C\{^1H\}$ NMR spectrum of compound **26d** (100 MHz, $CDCl_3$).

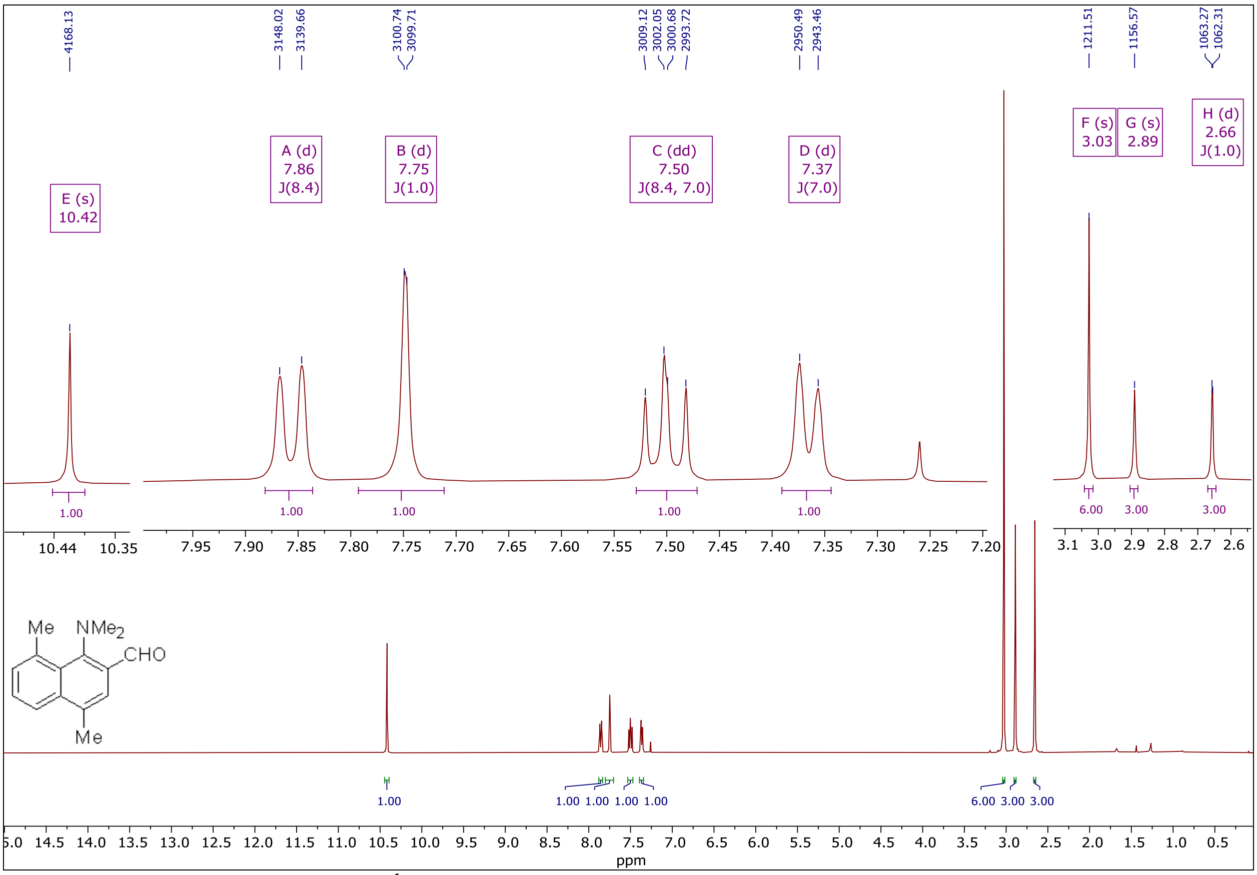

Fig. S110. ^1H NMR spectrum of compound **26e** (400 MHz, $CDCl_3$).

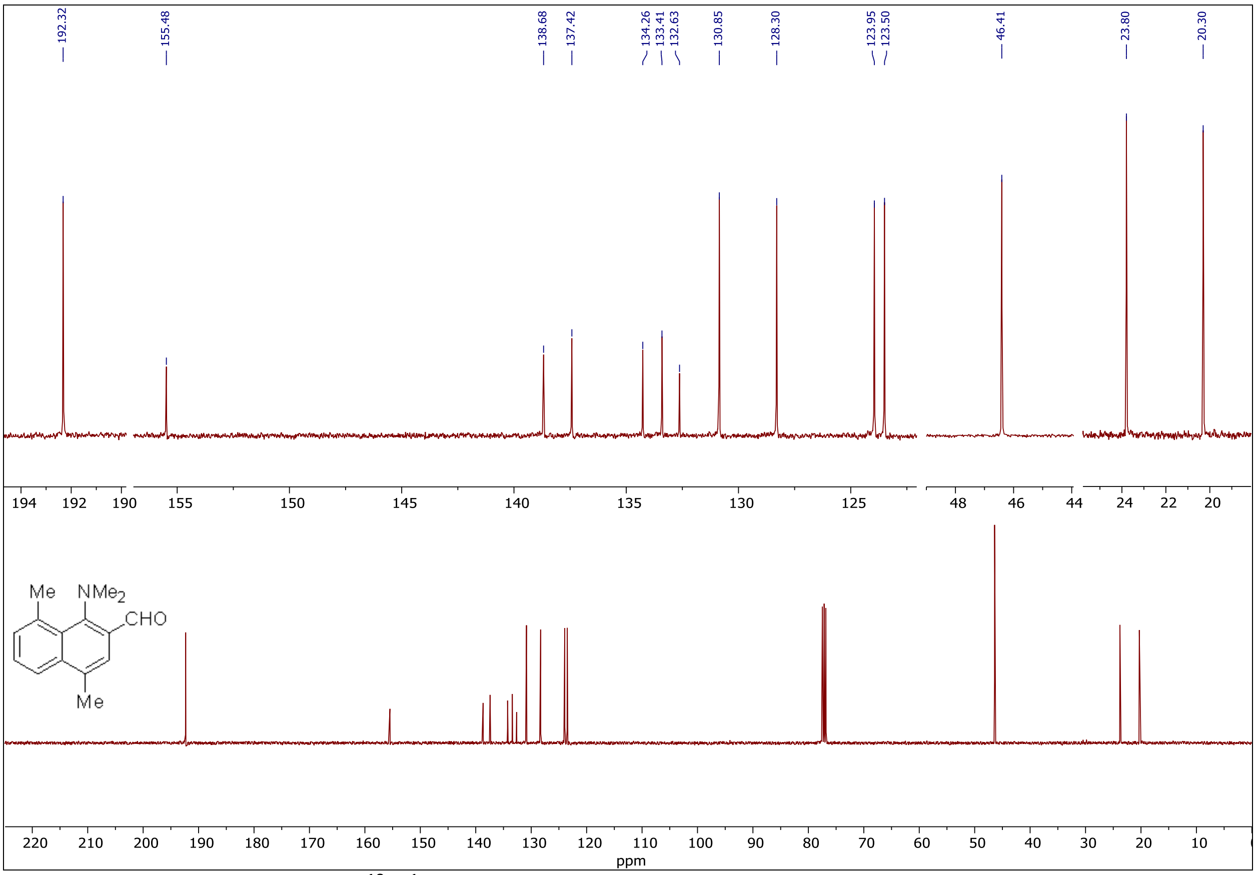

Fig. S111. $^{13}C\{^{1}H\}$ NMR spectrum of compound **26e** (100 MHz, $CDCl_3$).

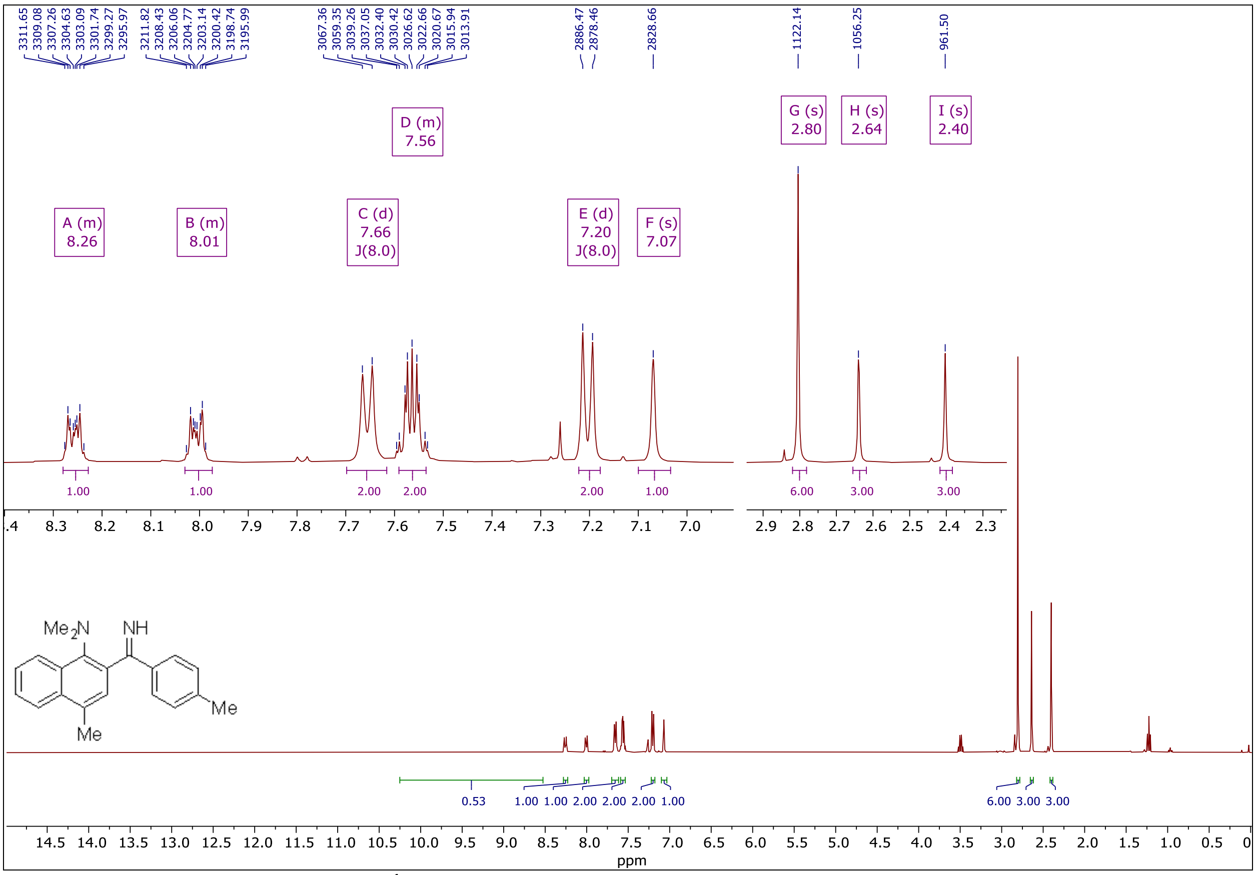

Fig. S112. ^{1}H NMR spectrum of compound **27a** (400 MHz, $CDCl_3$).

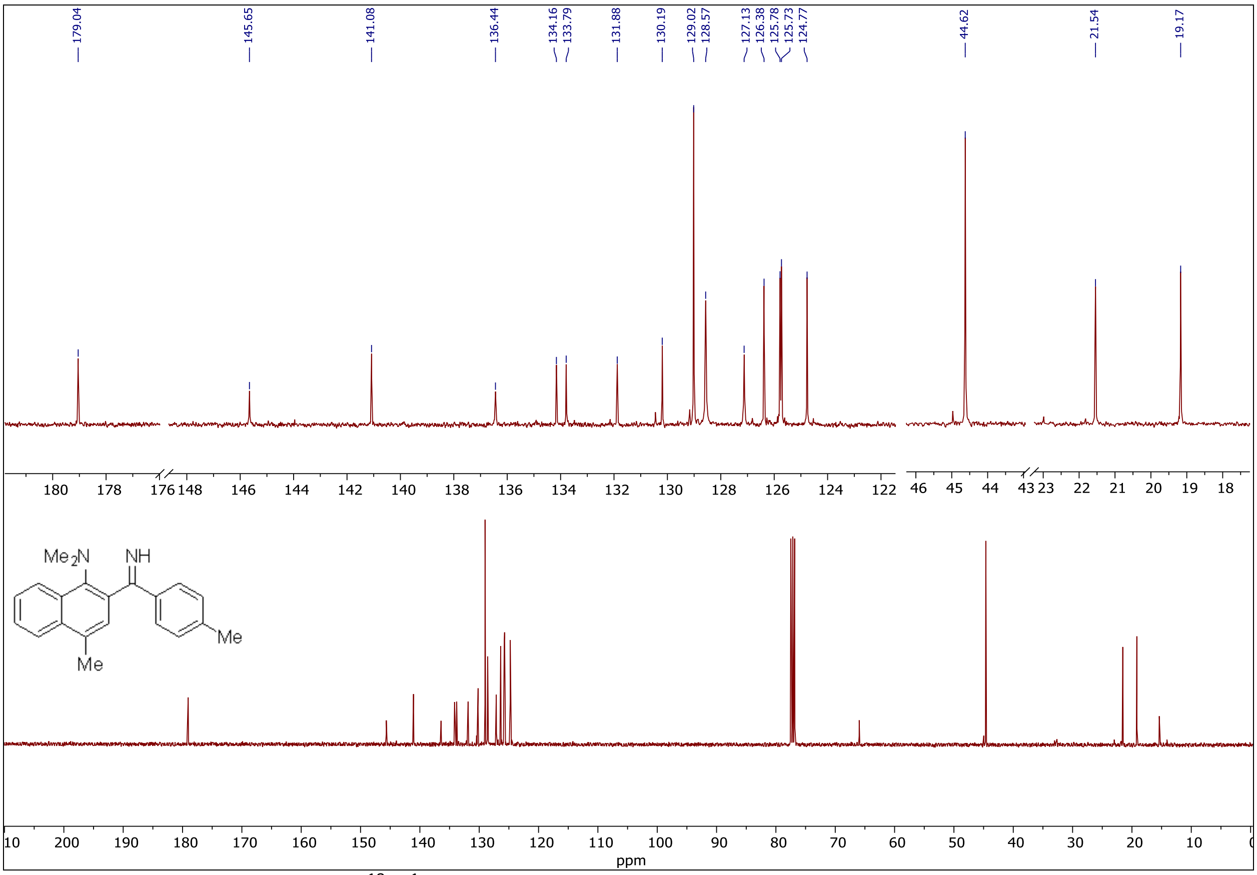

Fig. S113. $^{13}C\{^{1}H\}$ NMR spectrum of compound **27a** (100 MHz, $CDCl_3$).

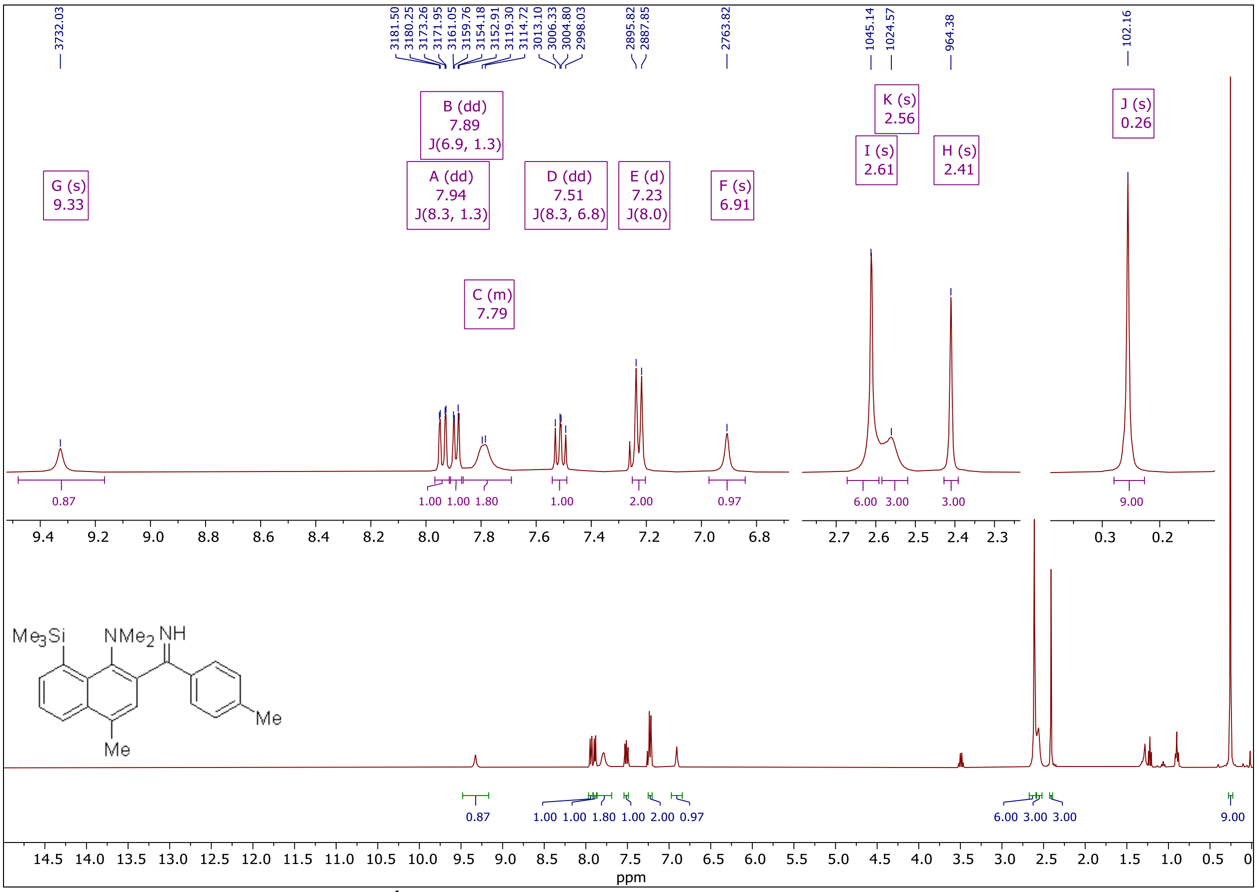

Fig. S114. ^{1}H NMR spectrum of compound **27e** (400 MHz, $CDCl_3$).

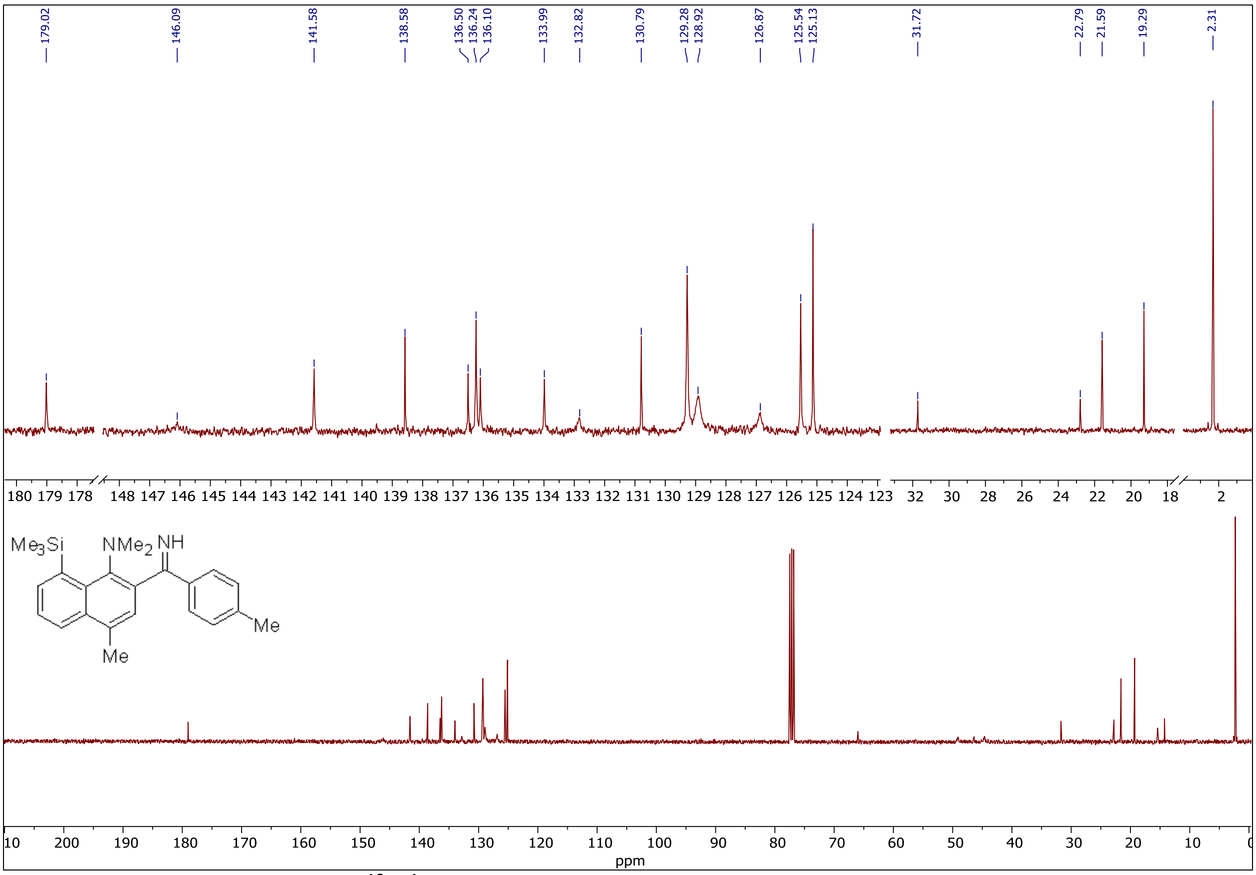

Fig. S115. $^{13}C\{^{1}H\}$ NMR spectrum of compound **27e** (100 MHz, $CDCl_3$).

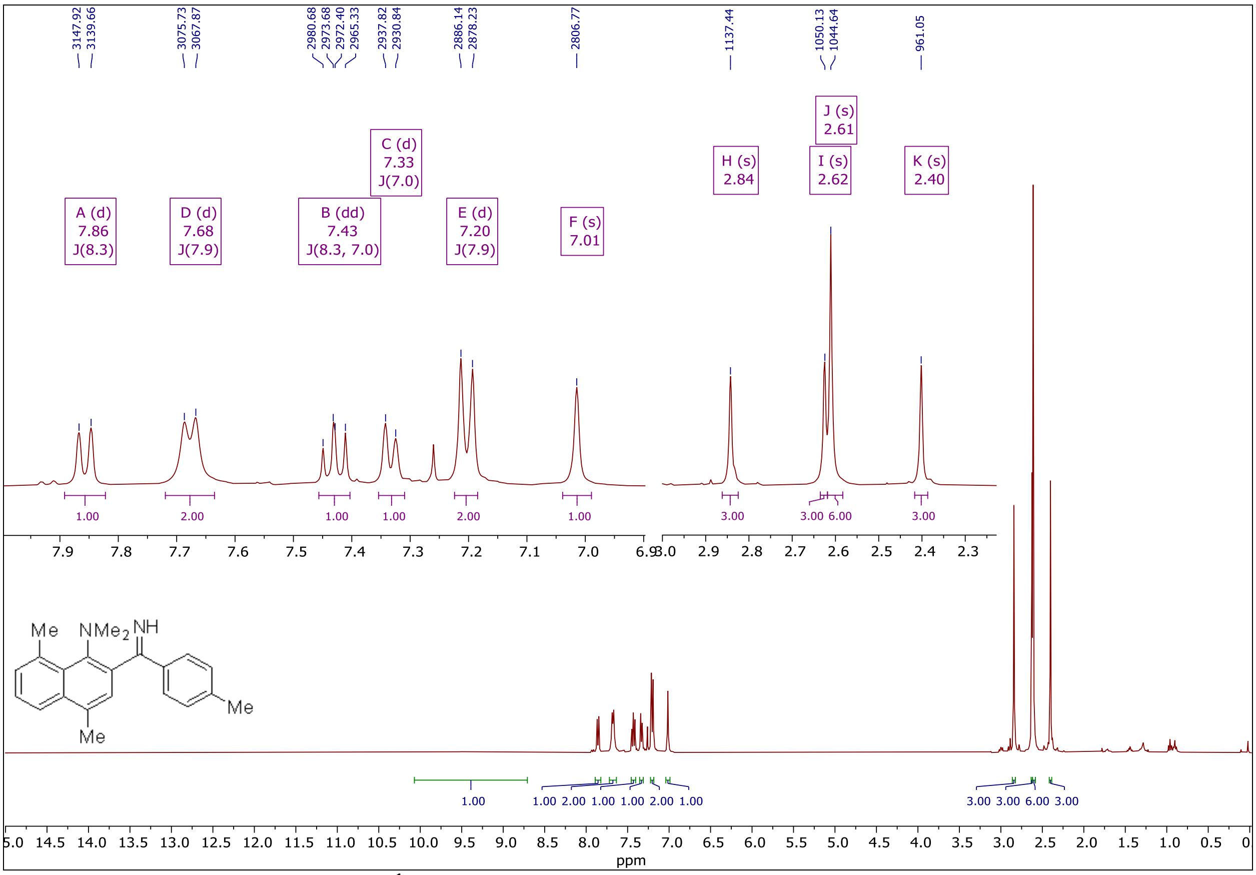

Fig. S116. ^{1}H NMR spectrum of compound **27f** (400 MHz, $CDCl_3$).

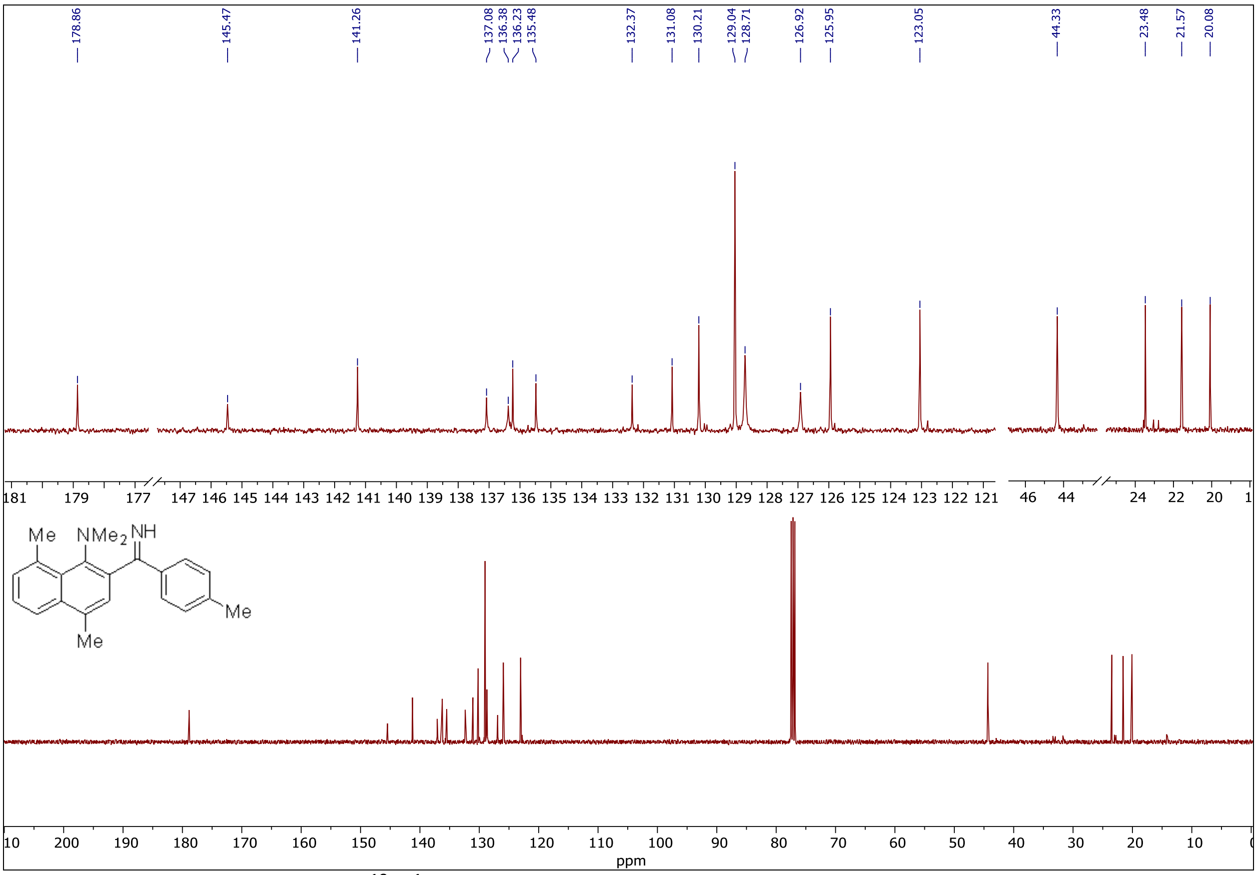

Fig. S117. $^{13}C\{^{1}H\}$ NMR spectrum of compound **27f** (100 MHz, $CDCl_3$).

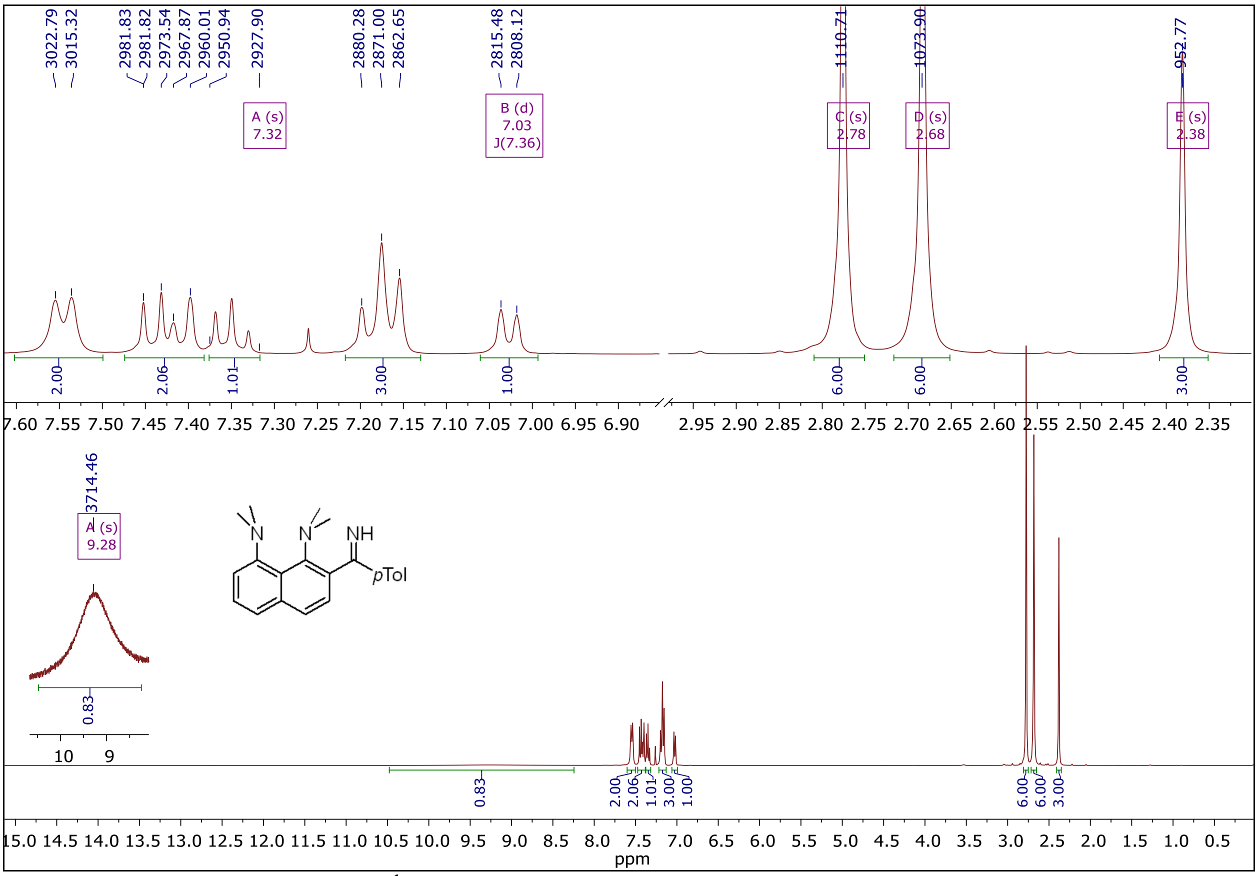

Fig. S118. ^{1}H NMR spectrum of compound **27h** (400 MHz, $CDCl_3$).

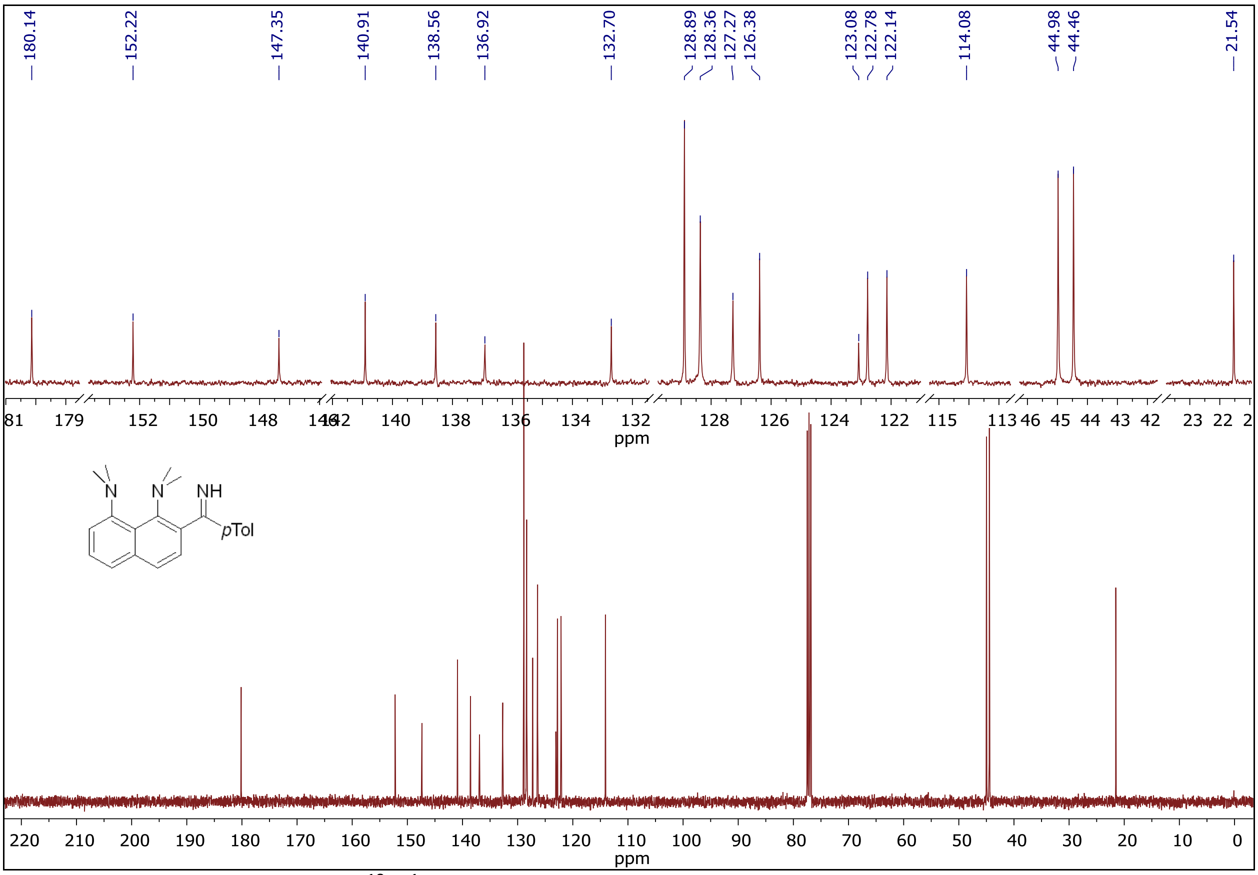

Fig. S119. $^{13}C\{^1H\}$ NMR spectrum of compound **27h** (100 MHz, $CDCl_3$).

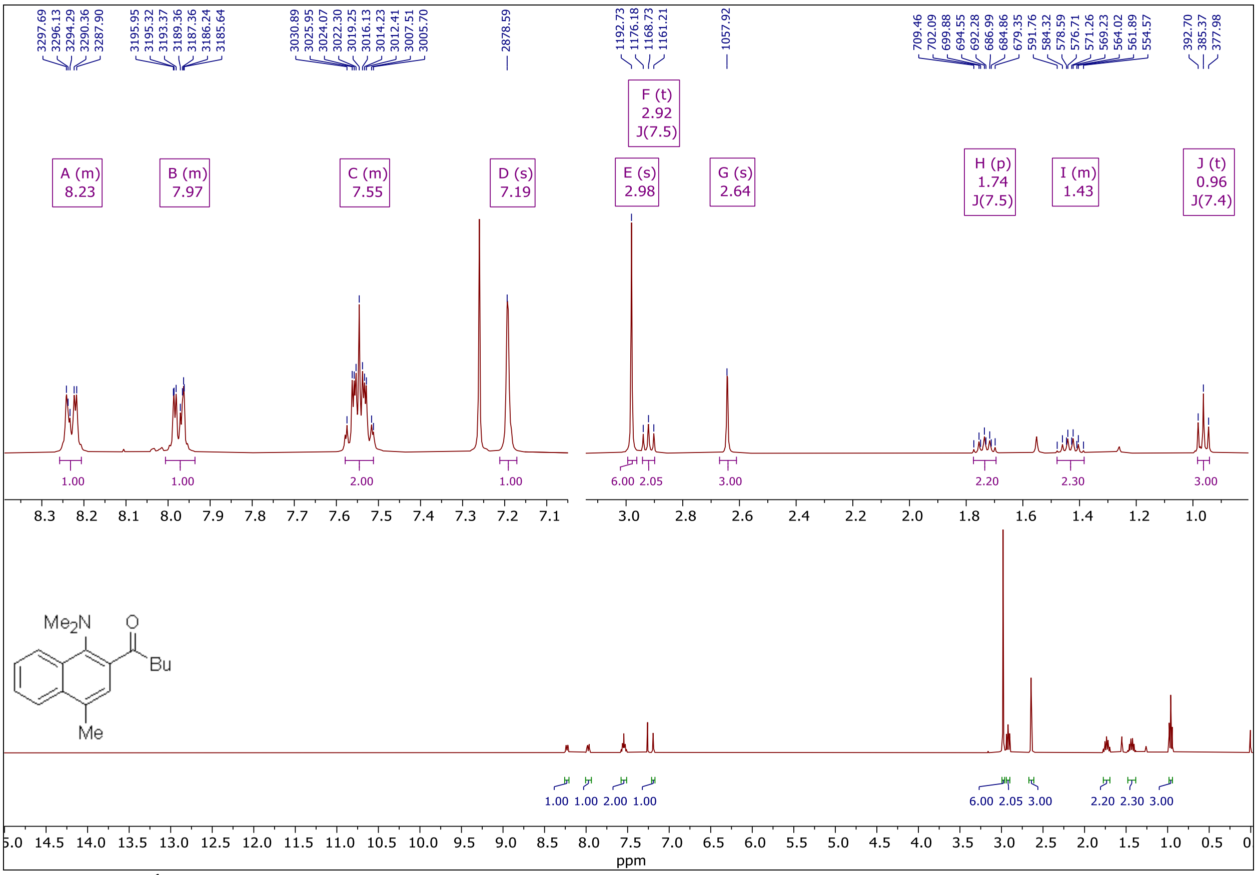

Fig. S120. ^1H NMR spectrum of 1-(1-(dimethylamino)-4-methylnaphthalen-2-yl)pentan-1-one (400 MHz, $CDCl_3$).

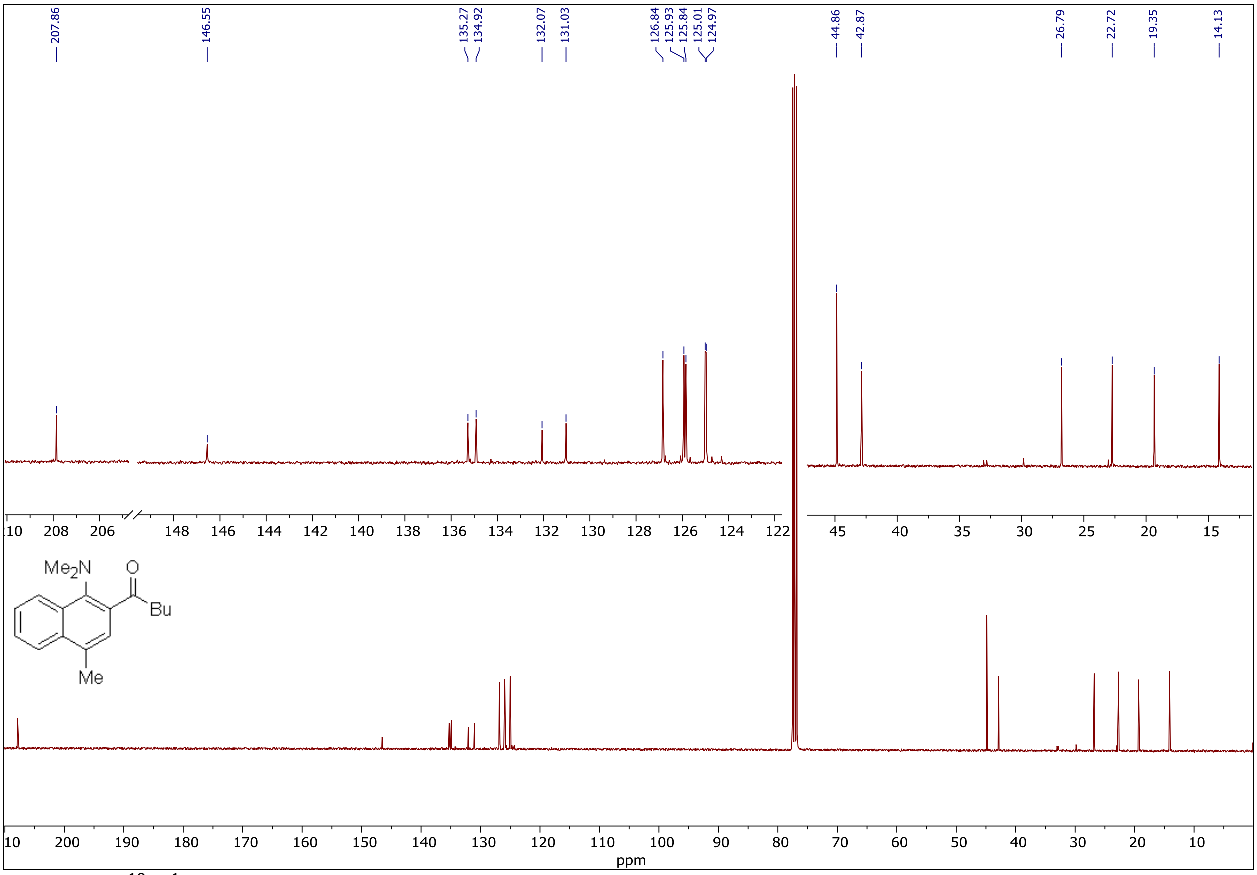

Fig. S121. $^{13}C\{^{1}H\}$ NMR spectrum of 1-(1-(dimethylamino)-4-methylnaphthalen-2-yl)pentan-1-one (100 MHz, $CDCl_3$).

X-ray studies

Table S5. Crystal data and structure refinement for **4d, 5e, 6e**.

Identification code	**4d**	**5e**	**6e**
CCDC code	2413769	2413771	2413772
Empirical formula	$C_{15}H_{18}N_2OS$	$C_{15}H_{16}N_2$	$C_{14}H_{14}N_2$
Formula weight	274.38	224.30	210.27
Temperature, K	120.00(10)	119.99(13)	120.4(9)
Crystal system	triclinic	orthorhombic	monoclinic
Space group	$P\overline{1}$	$P2_12_12_1$	$P2_1/n$
a, Å	7.1664(5)	7.14620(10)	8.3444(2)
b, Å	9.6849(8)	10.5169(2)	7.41260(10)
c, Å	10.7565(6)	16.4839(3)	17.7578(4)
α, °	85.552(6)	90	90
β, °	74.515(5)	90	97.405(2)
γ, °	85.032(6)	90	90
Volume, $Å^3$	715.61(9)	1238.86(4)	1089.22(4)
Z	2	4	4
ρ_{calc}, g/cm^3	1.273	1.203	1.282
μ, mm^{-1}	1.951	0.550	0.593
F(000)	292.0	480.0	448.0
Crystal size, mm^3	0.32 × 0.22 × 0.16	0.20 × 0.14 × 0.08	0.10 × 0.08 × 0.04
Radiation	Cu Kα (λ = 1.54184)	Cu Kα (λ = 1.54184)	Cu Kα (λ = 1.54184)
2Θ range for data collection, °	8.544 to 152.142	9.916 to 143.72	10.03 to 143.518
Index ranges	-8 ≤ h ≤ 8, -12 ≤ k ≤ 12, -15 ≤ l ≤ 15	-8 ≤ h ≤ 8, -12 ≤ k ≤ 12, -19 ≤ l ≤ 20	-10 ≤ h ≤ 10, -8 ≤ k ≤ 9, -21 ≤ l ≤ 21
Reflections collected	5151	7849	5767
Independent reflections	2895 [R_{int} = 0.0386, R_{sigma} = 0.0376]	2406 [R_{int} = 0.0401, R_{sigma} = 0.0383]	2125 [R_{int} = 0.0264, R_{sigma} = 0.0353]
Data/restraints/ parameters	2895/0/177	2406/0/158	2125/0/148
Goodness-of-fit on F^2	1.089	1.051	1.081
Final R indexes [I≥2σ (I)]	R_1 = 0.0480, wR_2 = 0.1312	R_1 = 0.0348, wR_2 = 0.0896	R_1 = 0.0410, wR_2 = 0.1078
Final R indexes [all data]	R_1 = 0.0544, wR_2 = 0.1358	R_1 = 0.0364, wR_2 = 0.0912	R_1 = 0.0471, wR_2 = 0.1127
Largest diff. peak/ hole, e·$Å^{-3}$	0.41/-0.33	0.11/-0.15	0.20/-0.22
Flack parameter	-	-	-

Table S6. Crystal data and structure refinement for **12a, 7d, 13a**.

Identification code	**12a**	**7d**	**13a**
CCDC code	2413775	2413770	2413773
Empirical formula	$C_{20}H_{18}N_2$	$C_{13}H_{11}NOS$	$C_{19}H_{15}NO$
Formula weight	286.37	229.29	273.32
Temperature, K	120.00(10)	119.97(13)	119.99(10)
Crystal system	monoclinic	monoclinic	monoclinic
Space group	$P2_1/n$	$P2_1/c$	$P2_1/n$
a, Å	14.9149(5)	7.4635(2)	11.8071(6)
b, Å	5.8595(2)	12.9592(3)	7.0202(2)
c, Å	17.7885(6)	11.4450(3)	17.6669(9)
α, °	90	90	90
β, °	107.473(4)	105.090(2)	106.300(5)
γ, °	90	90	90
Volume, $Å^3$	1482.87(9)	1068.80(5)	1405.52(11)
Z	4	4	4
ρ_{calc}, g/cm^3	1.283	1.425	1.292
μ, mm^{-1}	0.582	2.479	0.625
F(000)	608.0	480.0	576.0
Crystal size, mm^3	0.15 × 0.05 × 0.02	0.14 × 0.10 × 0.06	0.22 × 0.16 × 0.12
Radiation	Cu Kα (λ = 1.54184)	Cu Kα (λ = 1.54184)	Cu Kα (λ = 1.54184)
2Θ range for data collection, °	6.822 to 140.746	10.49 to 143.284	10.534 to 150.736
Index ranges	-18 ≤ h ≤ 17, -7 ≤ k ≤ 7, -21 ≤ l ≤ 19	-9 ≤ h ≤ 9, -8 ≤ k ≤ 15, -14 ≤ l ≤ 13	-14 ≤ h ≤ 13, -6 ≤ k ≤ 8, -20 ≤ l ≤ 21
Reflections collected	7893	5806	5426
Independent reflections	2793 [R_{int} = 0.0303, R_{sigma} = 0.0411]	2087 [R_{int} = 0.0395, R_{sigma} = 0.0426]	2649 [R_{int} = 0.0359, R_{sigma} = 0.0386]
Data/restraints/ parameters	2793/0/202	2087/0/147	2649/0/193
Goodness-of-fit on F^2	1.059	1.078	1.029
Final R indexes [I≥2σ (I)]	R_1 = 0.0487, wR_2 = 0.1145	R_1 = 0.0444, wR_2 = 0.1296	R_1 = 0.0505, wR_2 = 0.1358
Final R indexes [all data]	R_1 = 0.0587, wR_2 = 0.1195	R_1 = 0.0491, wR_2 = 0.1346	R_1 = 0.0607, wR_2 = 0.1486
Largest diff. peak/ hole, e·$Å^{-3}$	0.22/-0.23	0.34/-0.44	0.35/-0.25
Flack parameter	-	-	-

Table S7. Crystal data and structure refinement for **14a** and **14g**.

Identification code	**14a**	**14g**
CCDC code	2413776	2413781
Empirical formula	$C_{21}H_{19}N$	$C_{22}H_{22}N_2$
Formula weight	285.37	300.39
Temperature, K	100.00(10)	119.98(16)
Crystal system	orthorhombic	orthorhombic
Space group	Pbca	Pbcn
a, Å	13.9030(4)	11.2218(2)
b, Å	8.7955(2)	10.6592(2)
c, Å	24.6525(6)	26.5035(6)
α, °	90	90
β, °	90	90
γ, °	90	90
Volume, $Å^3$	3014.60(13)	3170.23(11)
Z	8	8
$ρ_{calc}$, g/cm^3	1.258	1.259
μ, mm^{-1}	550	0.567
F(000)	1216.0	1280.0
Crystal size, mm^3	0.10 × 0.06 × 0.04	0.11 × 0.07 × 0.03
Radiation	Cu Kα (λ = 1.54184)	Cu Kα (λ = 1.54184)
2Θ range for data collection, °	7.14 to 159.628	6.718 to 158.296
Index ranges	-17 ≤ h ≤ 17, -11 ≤ k ≤ 10, -31 ≤ l ≤ 26	-10 ≤ h ≤ 14, -13 ≤ k ≤ 13, -33 ≤ l ≤ 33
Reflections collected	22202	24594
Independent reflections	3201 [R_{int} = 0.0265, R_{sigma} = 0.0179]	3364 [R_{int} = 0.0391, R_{sigma} = 0.0219]
Data/restraints/ parameters	3201/0/213	3364/0/211
Goodness-of-fit on F^2	1.036	1.107
Final R indexes [I≥2σ (I)]	R_1 = 0.0383, wR_2 = 0.1056	R_1 = 0.0468, wR_2 = 0.1234
Final R indexes [all data]	R_1 = 0.0403, wR_2 = 0.1072	R_1 = 0.0493, wR_2 = 0.1250
Largest diff. peak/ hole, e·$Å^{-3}$	0.20/-0.24	0.18/-0.26
Flack parameter	-	-

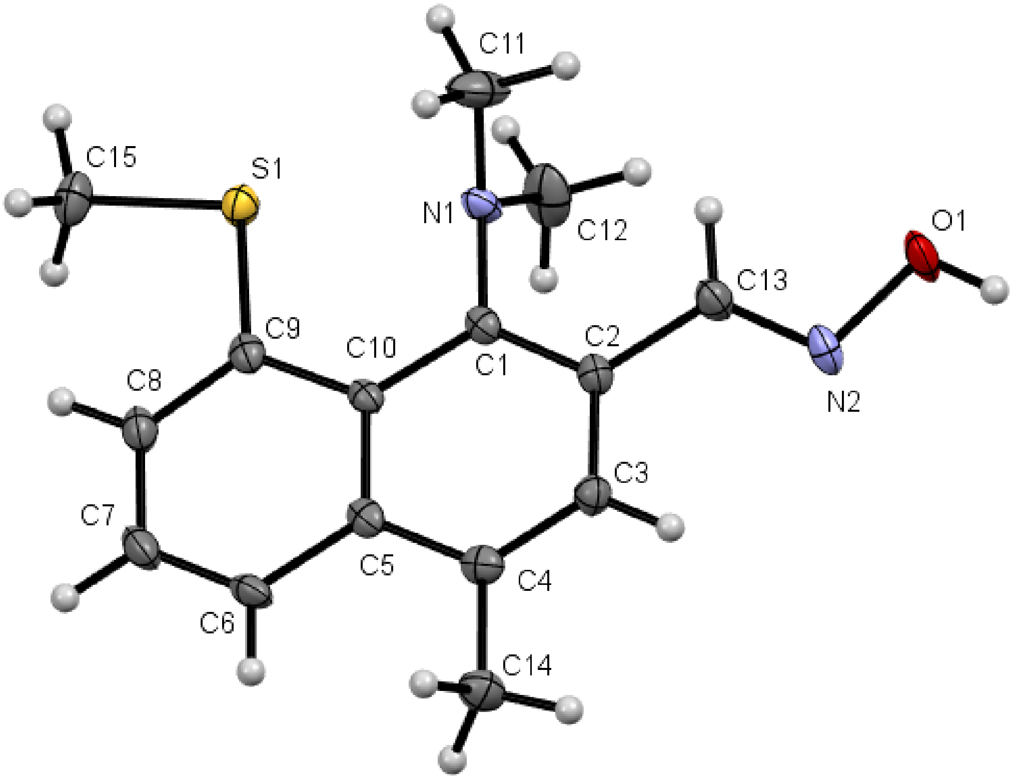

Fig. S122. Molecular structure of **4d** (by means of XRD) with an ellipsoid contour at the 50% probability level.

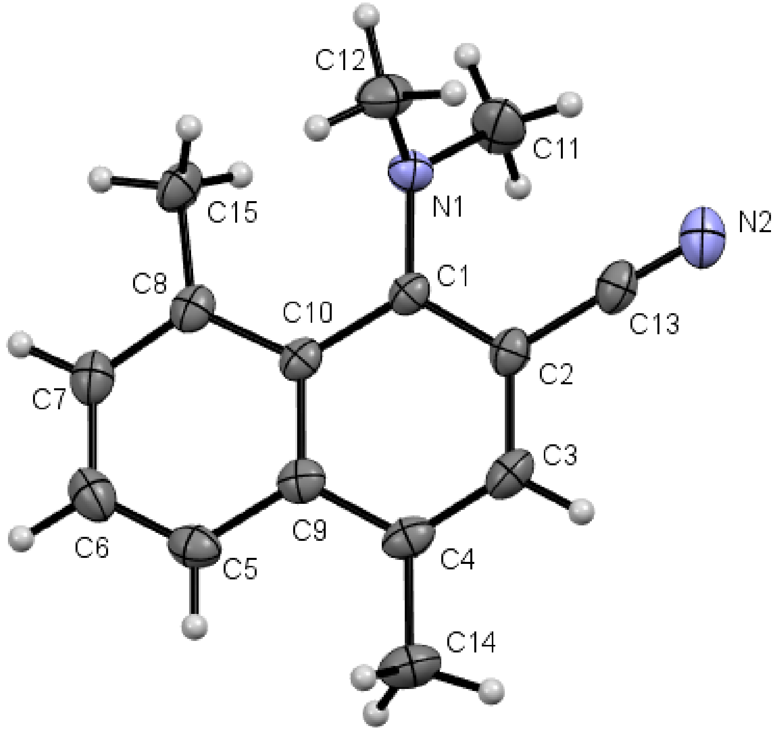

Fig. S123. Molecular structure of **5e** (by means of XRD) with an ellipsoid contour at the 50% probability level.

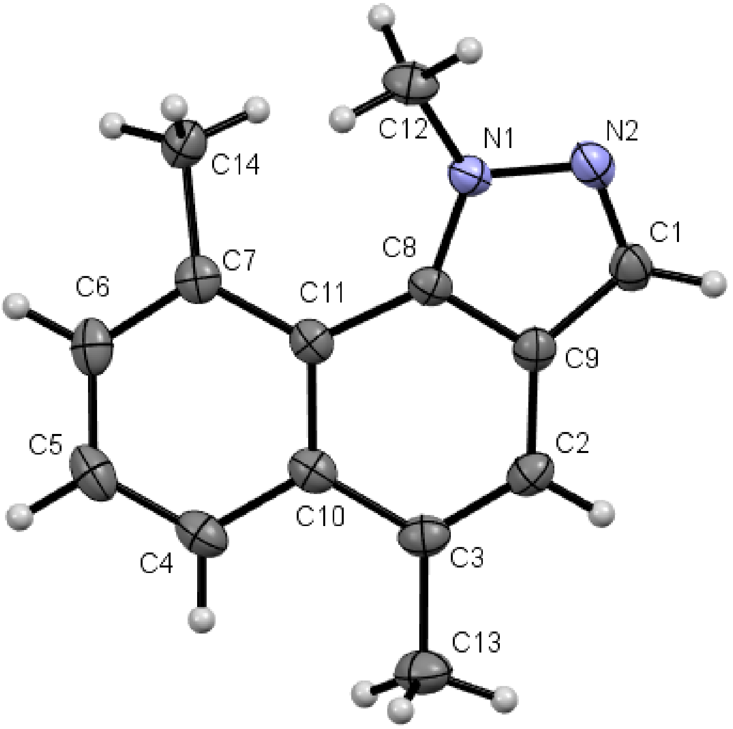

Fig. S124. Molecular structure of **6e** (by means of XRD) with an ellipsoid contour at the 50% probability level.

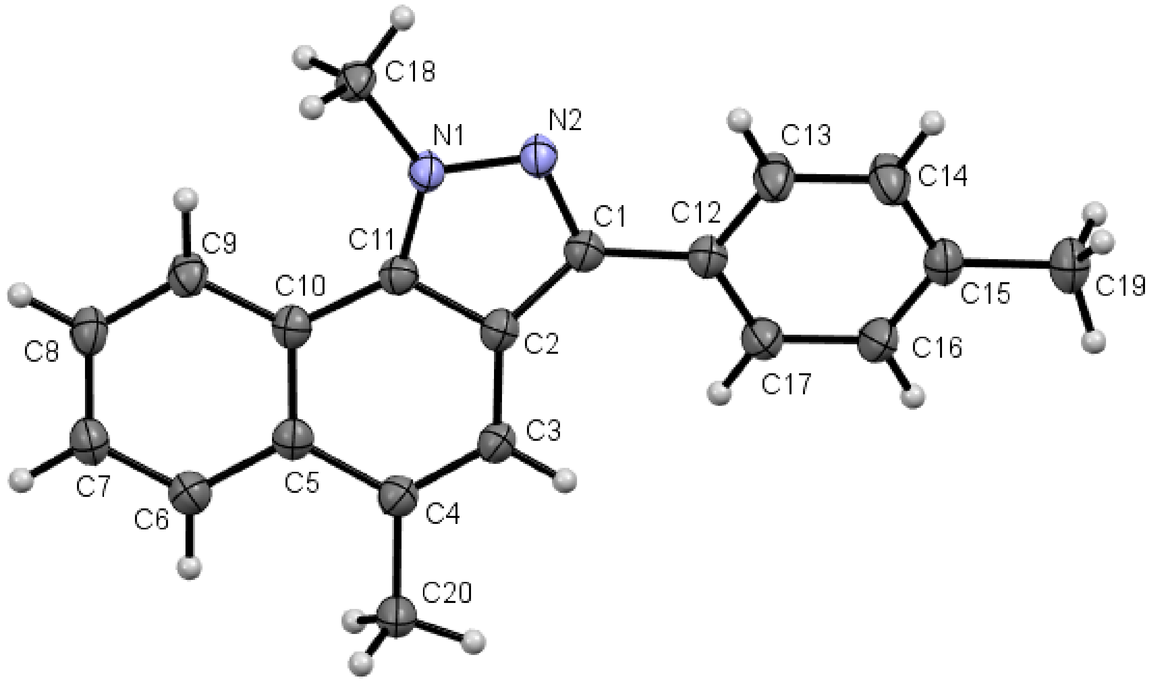

Fig. S125. Molecular structure of **12a** (by means of XRD) with an ellipsoid contour at the 50% probability level.

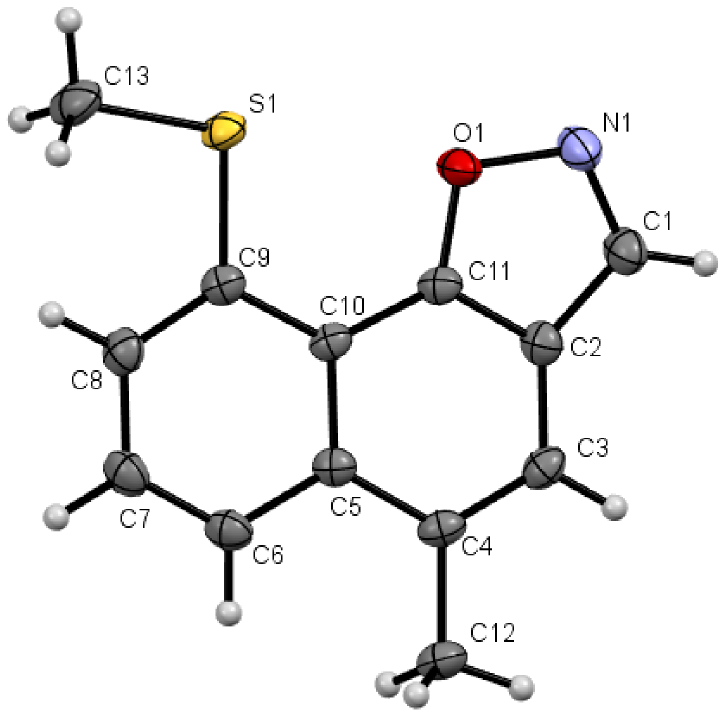

Fig. S126. Molecular structure of **7d** (by means of XRD) with an ellipsoid contour at the 50% probability level.

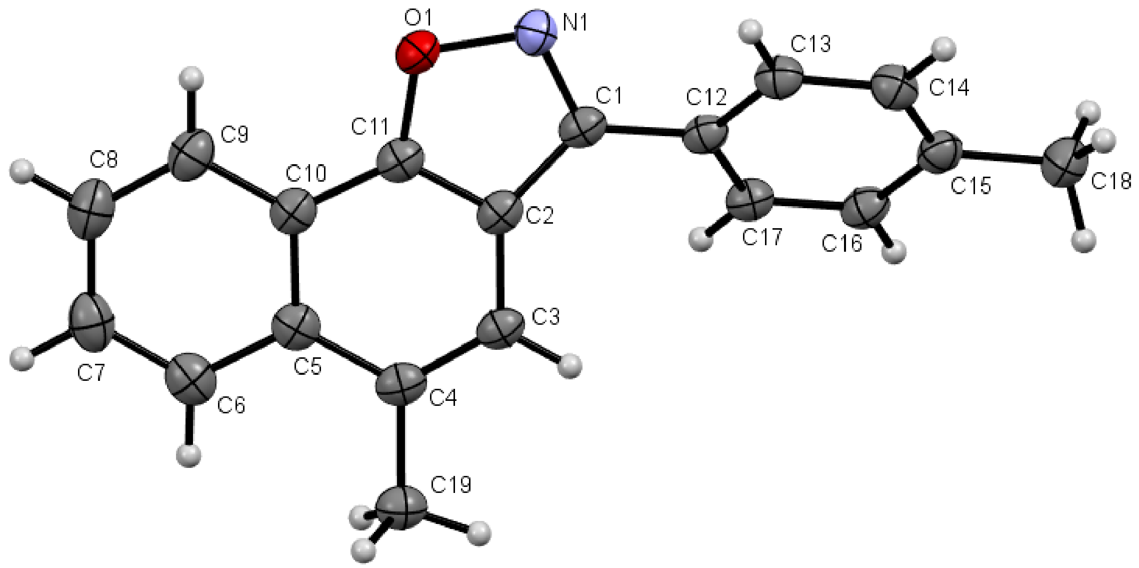

Fig. S127. Molecular structure of **13a** (by means of XRD) with an ellipsoid contour at the 50% probability level.

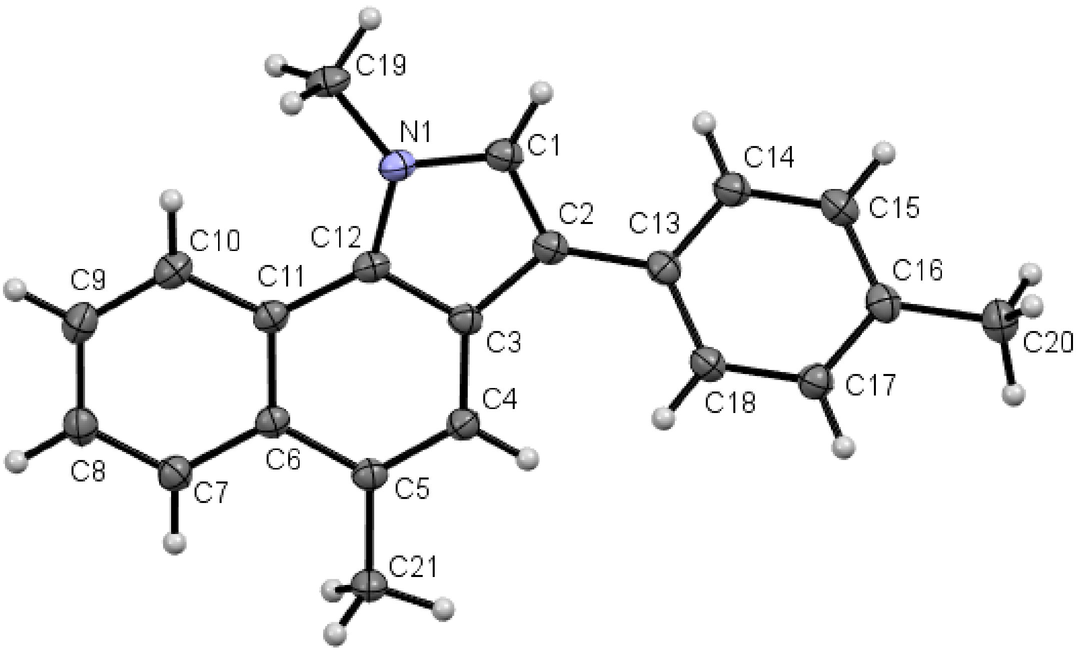

Fig. S128. Molecular structure of **14a** (by means of XRD) with an ellipsoid contour at the 50% probability level.

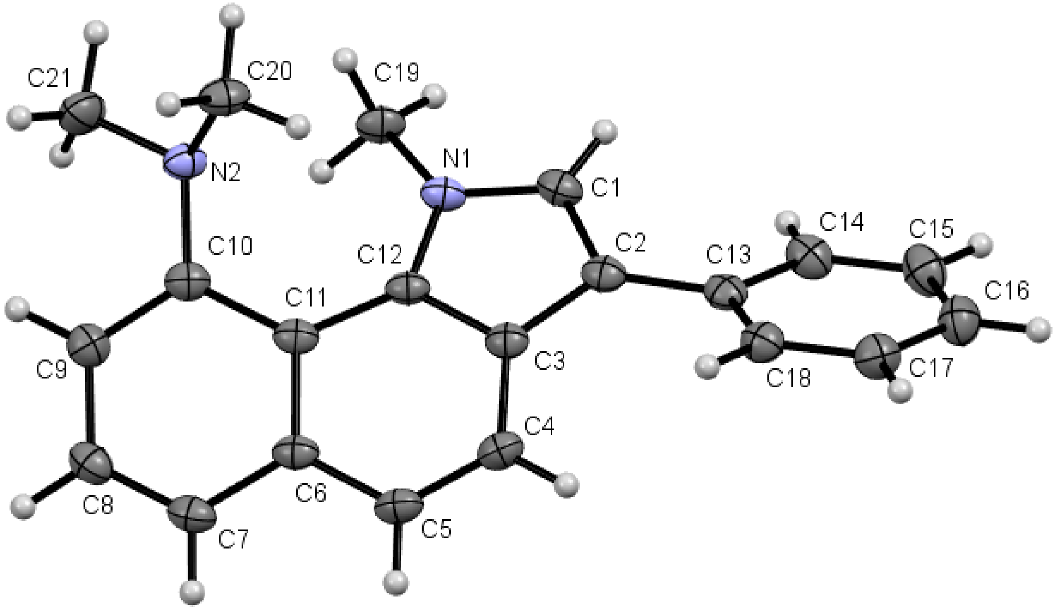

Fig. S129. Molecular structure of **14g** (by means of XRD) with an ellipsoid contour at the 50% probability level.

References

(1) Meshalkin, S. A.; Tsybulin, S. V.; Bardakov, V. G.; Tatarinov, I. A.; Shitov, D. A.; Tupikina, E. Y.; Efremova, M. M.; Antonov, A. S. “Buttressing Effect” in the Halogen-Lithium Exchange in *Ortho* -Bromo- *N,N* -dimethylanilines and Related Naphthalenes. *Chem. – A Eur. J.* **2024**, *30* (14). https://doi.org/10.1002/chem.202303956.

(2) Povalyakhina, M. A.; Antonov, A. S.; Dyablo, O. V.; Ozeryanskii, V. A.; Pozharskii, A. F. H-Bond-Assisted Intramolecular Nucleophilic Displacement of the 1-NMe 2 Group in 1,8-Bis(Dimethylamino)Naphthalenes as a Route to Multinuclear Heterocyclic Compounds and Strained Naphthalene Derivatives. *J. Org. Chem.* **2011**, *76* (17), 7157–7166. https://doi.org/10.1021/jo201171z.

(3) Pozharskii, A. F.; Ozeryanskii, V. A.; Filatova, E. A.; Dyablo, O. V.; Pogosova, O. G.; Borodkin, G. S.; Filarowski, A.; Steglenko, D. V. Neutral Pyrrole Nitrogen Atom as a π- and Mixed n,π-Donor in Hydrogen Bonding. *J. Org. Chem.* **2019**, *84* (2), 726–737. https://doi.org/10.1021/acs.joc.8b02562.

(4) Meshalkin, S. A.; Tsybulin, S. V.; Bardakov, V. G.; Tatarinov, I. A.; Shitov, D. A.; Tupikina, E. Y.; Efremova, M. M.; Antonov, A. S. “Buttressing Effect” in the Halogen-Lithium Exchange in Ortho-Bromo-N,N-Dimethylanilines and Related Naphthalenes. *Chem. - A Eur. J.* **2024**, *30* (14). https://doi.org/10.1002/chem.202303956.

(5) Vinogradova, O. V.; Filatova, E. A.; Vistorobskii, N. V.; Pozharskii, A. F.; Borovlev, I. V.; Starikova, Z. A. Resonance-Stabilized α-Naphthylmethyl Carbocations and Spiro Compounds Based Thereon: VII. Transformations of α-Naphthylmethyl Carbocations Stabilized by One Electron-Donor Group or Peri-Fused Heteroring. *Russ. J. Org. Chem.* **2006**, *42* (3), 338–348. https://doi.org/10.1134/S107042800603002X.

(6) Pozharskii, A. F.; Ozeryanskii, V. A. Peri-Naphthylenediamines. *Russ. Chem. Bull.* **1998**, *47* (1), 66–73. https://doi.org/10.1007/BF02495511.

(7) Pozharskii, A. F.; Degtyarev, A. V; Ryabtsova, O. V; Ozeryanskii, V. A.; Kletskii, M. E.; Starikova, Z. A.; Sobczyk, L.; Filarowski, A. 2-α-Hydroxyalkyl- and 2,7-Di(α-Hydroxyalkyl)-1,8-Bis(Dimethylamino)Naphthalenes: Stabilization of Nonconventional In/Out Conformers of “Proton Sponges” via N···H−O Intramolecular Hydrogen Bonding. A Remarkable Kind of Tandem Nitrogen Inversion. *J. Org. Chem.* **2007**, *72* (8), 3006–3019. https://doi.org/10.1021/jo062667v.
